# Chemoenzymatic Synthesis of Tenofovir

**DOI:** 10.1021/acs.joc.3c01005

**Published:** 2023-07-19

**Authors:** Beata Zdun, Tamara Reiter, Wolfgang Kroutil, Paweł Borowiecki

**Affiliations:** † Laboratory of Biocatalysis and Biotransformation, Department of Drugs Technology and Biotechnology, Faculty of Chemistry, Warsaw University of Technology, Koszykowa 75, 00-662 Warsaw, Poland; ‡ Institute of Chemistry, University of Graz, NAWI Graz, BioTechMed Graz, Field of Excellence BioHealth, Heinrichstrasse 28, 8010 Graz, Austria

## Abstract

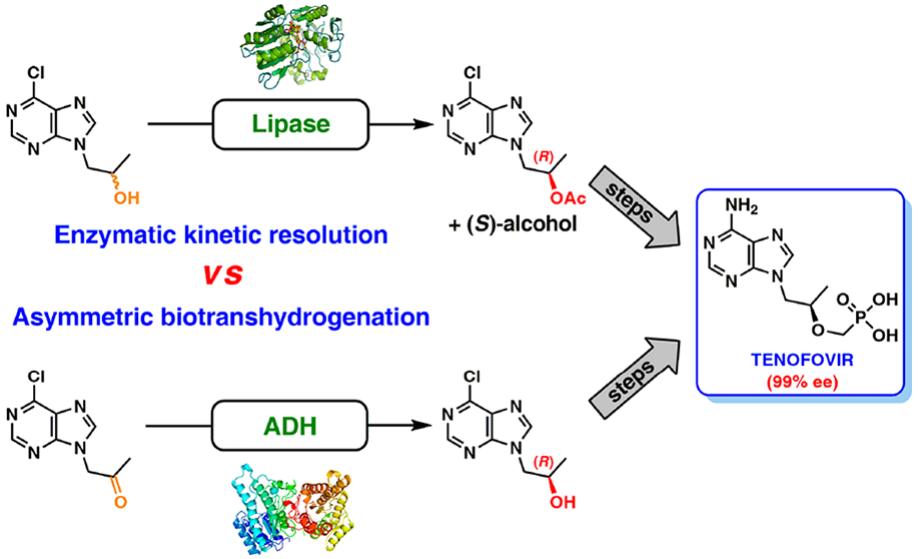

We report on novel chemoenzymatic routes toward tenofovir using low-cost starting materials and commercial or homemade enzyme preparations as biocatalysts. The biocatalytic key step was accomplished either via stereoselective reduction using an alcohol dehydrogenase or via kinetic resolution using a lipase. By employing a suspension of immobilized lipase from *Burkholderia cepacia* (Amano PS-IM) in a mixture of vinyl acetate and toluene, the desired (*R*)-ester (99% ee) was obtained on a 500 mg scale (60 mM) in 47% yield. Alternatively, stereoselective reduction of 1-(6-chloro-9*H*-purin-9-yl) propan-2-one (84 mg, 100 mM) catalyzed by lyophilized *E. coli* cells harboring recombinant alcohol dehydrogenase (ADH) from *Lactobacillus kefir* (*E. coli*/Lk-ADH Prince) allowed one to reach quantitative conversion, 86% yield and excellent optical purity (>99% ee) of the corresponding (*R*)-alcohol. The key (*R*)-intermediate was transformed into tenofovir through “one-pot” aminolysis–hydrolysis of (*R*)-acetate in NH_3_-saturated methanol, alkylation of the resulting (*R*)-alcohol with tosylated diethyl(hydroxymethyl) phosphonate, and bromotrimethylsilane (TMSBr)-mediated cleavage of the formed phosphonate ester into the free phosphonic acid. The elaborated enzymatic strategy could be applicable in the asymmetric synthesis of tenofovir prodrug derivatives, including 5′-disoproxil fumarate (TDF, Viread) and 5′-alafenamide (TAF, Vemlidy). The molecular basis of the stereoselectivity of the employed ADHs was revealed by molecular docking studies.

## 1 Introduction

Nowadays, developing cost-efficient and sustainable methodologies for the synthesis of enantiomerically pure active pharmaceutical ingredients (APIs), which often determine production cost, accessibility, and drug quality (i.e., therapeutic efficacy and patient safety), is of prime concern for both academia and industry.^1^ Moreover, chiral APIs are present in over 80% of drugs currently on the market, and therefore, their production in optically pure form is of paramount importance for target-oriented therapies.^2^

One of the sustainable and transition-metal-free industrial methods, which increases cost efficiency and reduces waste in small-molecule API synthesis by shortening synthetic routes mainly via bypassing protecting group requirements, relies on biocatalysis.^3^ Therefore, in a continuation of our interest in chemoenzymatic preparation of enantiomerically pure APIs,^4^ we present novel biocatalytic routes toward (*R*)-9-(2-phosphonomethoxypropyl) adenine [(*R*)-PMPA], namely, tenofovir (**TFV**).

**Figure 1:**
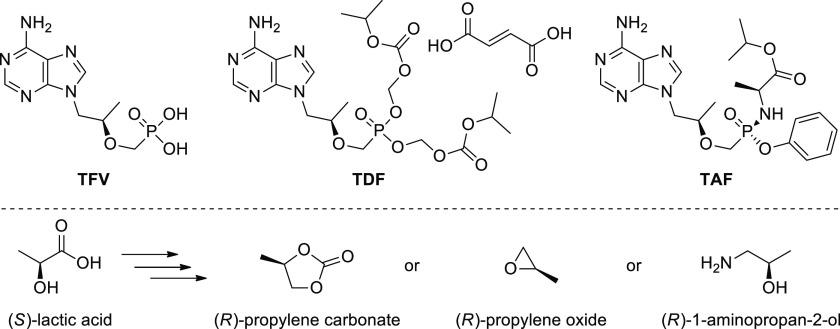
Chemical structures of tenofovir (**TFV**), tenofovir disoproxil fumarate (**TDF**), and tenofovir alafenamide (**TAF**), as well as chiral building blocks, derived from (*S*)-lactic acid, mostly employed in the manufacturing processes of the **TFV**, **TDF**, and **TAF**.

Tenofovir and its lipophilic prodrugs [i.e., tenofovir disoproxil fumarate (**TDF**) and tenofovir alafenamide (**TAF**)] (Figure 1) are powerful antiretroviral agents that already have gained the status of being “frontline drugs” for the treatment of human immunodeficiency virus (HIV) infection and chronic hepatitis B caused by HBV.^5^ The anti-HIV activity of parent tenofovir, resulting from competitive inhibition of HIV reverse transcriptase with respect to dATP, is strictly related to the absolute configuration of its stereogenic center. In this context, the (*R*)-configured title API is ca. 100-fold more active as a nucleoside reverse transcriptase inhibitor (NRTI) than its enantiomeric counterpart.^6^ Therefore, the synthesis and administration of pharmacologically relevant **TFV** single enantiomer by HIV-infected patients are required for highly active antiretroviral therapy (HAART).^7^

Most **TFV** manufacturing processes utilize adenine and (*R*)-propylene carbonate as starting materials.^8^ However, synthesis of (*R*)-propylene carbonate requires an asymmetric synthetic methodology, whereas *N*-alkylation of adenine with this chiral reagent generates ca. 10% of material loss due to undesired regioisomer formation.^9^ Recently, to reduce the costs of the production of **TFV**, an alternative synthetic strategy utilizing 5-amino-4-cyanoimidazole (HPI) as an intermediate was developed.^10^ This method starts with hydrogen cyanide, which is highly toxic and requires a well-ventilated fume hood equipped with a HCN detector. In turn, despite significant efforts already being made toward developing chemoenzymatic synthesis of many antiviral agents and their synthetic intermediates,^11^ tenofovir and its prodrugs lack a vast repertoire of such creative attempts. Until now, only two biocatalytic approaches have been reported and included a gram-scale *Candida antarctica* lipase B (CAL-B)-catalyzed kinetic resolution of racemic 1-(trityloxy)propan-2-ol^12^ or enzymatic reduction of prochiral 1-(6-amino-9*H*-purin-9-yl) propan-2-one by using the (*R*)-selective Codexis KRED P1B02.^13^ Both methods suffer from severe limitations that render them not ideal for potential upscaling. Thus, the first protocol elongates a routine synthesis of tenofovir in about six synthetic steps. In contrast, the second approach requires commercial enzymes of unknown composition, which enforces dependency on a particular supplier.

In this work, our ultimate goal was to elaborate practical and sustainable chemoenzymatic syntheses of enantiomerically pure tenofovir based on the conventional biocatalytic approaches via lipase-catalyzed kinetic resolution (KR) of the respective racemate and/or stereoselective biotranshydrogenation of the corresponding prochiral ketone using well-described engineered short-chain ADHs heterogeneously overexpressed in competent *E*. *coli* cells. In order to increase the applicability of these methods in industry and thus enhance supply chains worldwide to improve patient access, the key aim of the developed methods was to employ readily available, inexpensive starting materials and well-defined biocatalysts as well as proceed with a minimized number of steps prone to scalability in classical stirred-tank-type (bio)chemical reactors. Inspired by the synthesis reported by Zhang et al.,^14^ we envisioned (*R*)-1-(6-chloro-9*H*-purin-9-yl) propan-2-ol and/or (*R*)-1-(6-iodo-9*H*-purin-9-yl) propan-2-ol as pivotal building blocks to convey chirality into the title API.

## 2 Results and Discussion

**Scheme 1:**
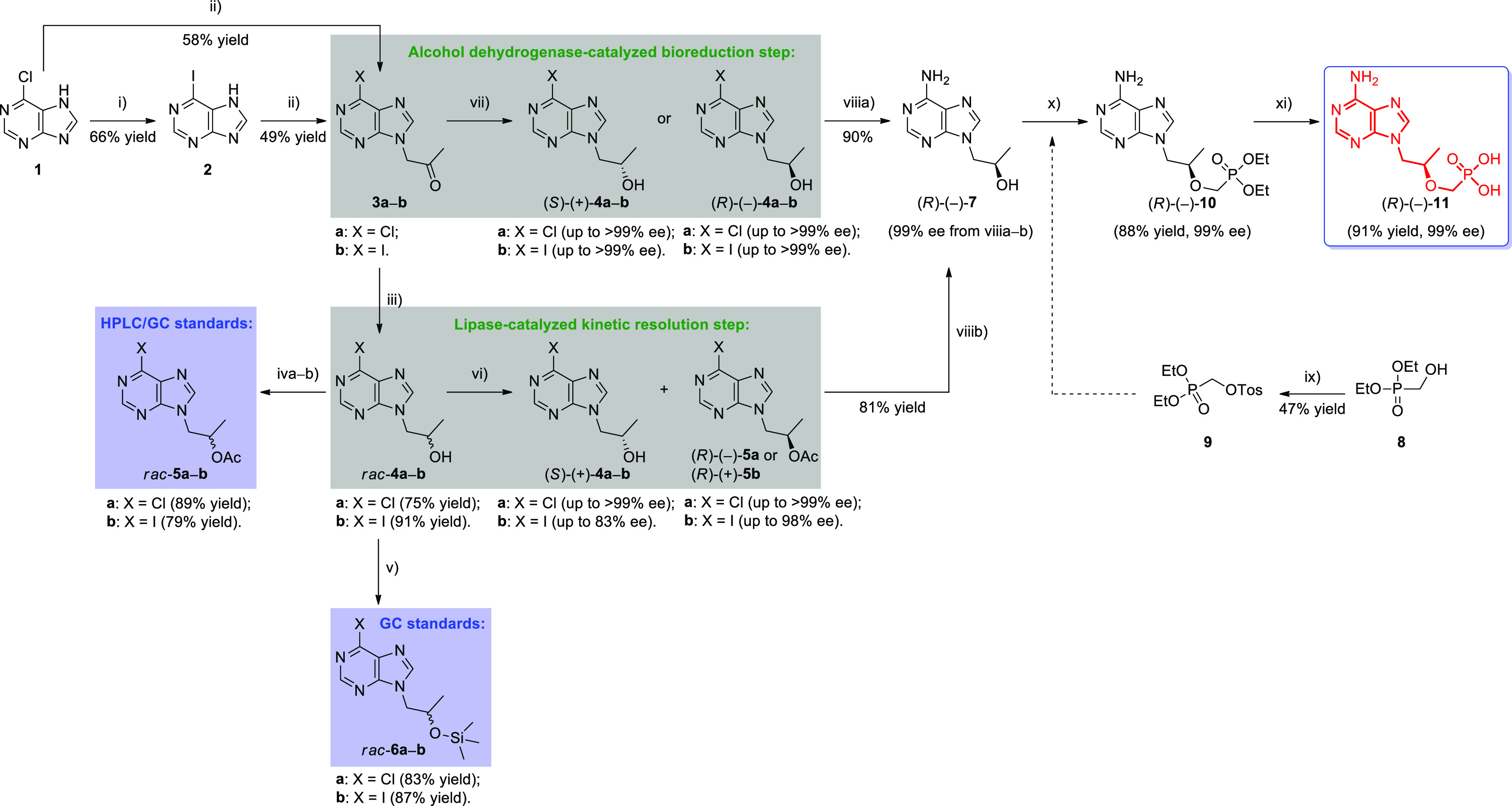
Chemoenzymatic Synthesis of Tenofovir [(*R*)-(−)-**11**] Reagents and conditions: (i) **1** (18.44 mmol), ice cold 57% HI_aq._ (10 equiv), 2 h at 0–5 °C with standing; (ii) **1** (12.9 mmol) or **2** (8.1 mmol), anhydrous K_2_CO_3_ (1 equiv), chloroacetone (1.1 equiv), dry DMF, 12 h at 25 °C; (iii) **3a** (7.5 mmol) or **3b** (3.3 mmol), NaBH_4_ (1.2 equiv), MeOH/CH_3_CN (2:1 v/v), 40 min at 0–5 °C; (iva) *rac*-**4a** (2.4 mmol), acetyl chloride (1.5 equiv), Et_3_N (1.5 equiv), DMAP (cat.), dry CH_2_Cl_2_, 15 min at 0–5 °C, then 48 h at 25 °C; (ivb) *rac*-**4b** (0.20 mmol), Ac_2_O (1.5 equiv), Et_3_N (1.5 equiv), DMAP (cat.), dry CH_2_Cl_2_, 15 min at 0–5 °C, then 24 h at 25 °C; (v) *rac*-**4a** (0.19 mmol) or *rac*-**4b** (0.13 mmol), *N,O*-bis(trimethylsilyl) acetamide (BSA, 4 equiv), CH_2_Cl_2_, 20 min at 25 °C; (vi) *rac*-**4a** (2.4 mmol, 60 mM final concentration), Amano PS-IM (42 mg/mmol), PhCH_3_/vinyl acetate (60 mL; 2:1 v/v), 26 h at 40 °C, 800 rpm (magnetic stirrer); (vii) **3a** (0.4 mmol, 100 mM final concentration), *E. coli*/(Lk-ADH-A or Lk-ADH Prince) (60 mg), NAD(P)H (1.0 mM final concentration), 50 mM Tris-HCl buffer (pH 7.5), 2-PrOH (10% v/v), DMSO (2.5% v/v), 4 mL (final volume), 24 h at 30 °C, 250 rpm (orbital shaker); (viiia–b) (*R*)-(−)-**4a** (0.47 mmol) or (*R*)-(−)-**5a** (0.40 mmol), NH_3_-saturated MeOH, 48 h at 150 °C (pressure tube); (ix) **8** (3.15 mmol), TsCl (1.2 equiv), Et_3_N (1.2 equiv), dry CH_2_Cl_2_, 15 min at 0–5 °C, then 24 h at 25 °C; (x) (*R*)-(−)-**7** (0.26 mmol), **9** (1.5 equiv), Mg(O^*t*^Bu)_2_ (1.5 equiv), dry DMF, 1 h at 65 °C, then 24 h at 75 °C; (xi) (*R*)-(−)-**10** (0.3 mmol, 99% ee), TMSBr (23 equiv), dry CH_2_Cl_2_, 48 h at 0–5 °C (pressure tube).

Herein, we report chemoenzymatic routes toward enantiomerically pure tenofovir (**11**). The synthetic pathway for the asymmetric synthesis of the nonracemic title API is outlined in Scheme 1 and involves two alternative biocatalytic approaches (i.e., lipase-catalyzed KR of *rac*-**4a** and -**4b** and ADH-catalyzed stereoselective bioreduction of **3a** and **3b**) to obtain the central key intermediates—(*R*)-1-(6-halo-9*H*-purin-9-yl) propan-2-ols [(*R*)-(−)-**4a** and -**4b**].

### 2.1 Lipase-Catalyzed Kinetic Resolution of Racemic Alcohols *rac*-**4a** and -**4b**

First, the syntheses of both ketones **3a** and **3b** and the corresponding racemic alcohols *rac*-**4a** and -**4b** were performed in analogy to the methods already reported in the literature^15^ (for details, see Supporting Information). In this regard, to obtain 1-(6-chloro-9*H*-purin-9-yl) propan-2-one (**3a**), alkylation of commercially available 6-chloropurine (**1**) with 1.1 equiv of chloroacetone in the presence of 1 equiv of anhydrous potassium carbonate (K_2_CO_3_) suspended in dry dimethylformamide (DMF) was performed, furnishing the carbonyl product **3a** in 58% yield. In turn, the synthesis of 1-(6-iodo-9*H*-purin-9-yl) propan-2-one (**3b**) was accomplished in a 2-step reaction sequence by the treatment of **1** with an aqueous solution of hydrogen iodide, followed by K_2_CO_3_-mediated alkylation of the isolated 6-iodopurine (**2**) with chloroacetone in the same manner as for **1**. In this case, the desired iodo-ketone **3b** was synthesized in 32% total yield after two steps. The resulting ketones **3a** and **3b** were then chemically reduced using a suspension of 1.2 equiv of sodium borohydride (NaBH_4_) in a mixture of methanol and acetonitrile (2:1 v/v), thus furnishing racemic alcohols *rac*-**4a** in 75% yield and *rac*-**4b** in 91% yield. Prior to developing the enzymatic kinetic resolution step, the syntheses of both racemic acetates *rac*-**5a** and -**5b** as analytical standards were performed using a conventional esterification protocol employing 1.5 equiv of acetic anhydride (Ac_2_O, in the case of *rac*-**5a**) or acetyl chloride (AcCl, in the case of *rac*-**5b**), 1.5 equiv of triethylamine (Et_3_N) as a base, and a catalytic amount of 4-(dimethylamino)pyridine (DMAP) in dry dichloromethane (CH_2_Cl_2_). In this case, the desired *rac*-**5a** was isolated in 89% yield, whereas *rac*-**5a** was isolated in 79% yield.

In the next step, the enzymatic KR of *rac*-**4a** and -**4b** was investigated by using a set of commonly used commercially available lipase preparations in the presence of an excess of vinyl acetate as an irreversible acyl donor in methyl *tert*-butyl ether (MTBE) as a model cosolvent at 40 °C (Table 1). Lipase-catalyzed KRs of *rac*-**4a** and -**4b** were carried out for either 16 or 24 h.

**Table 1:**
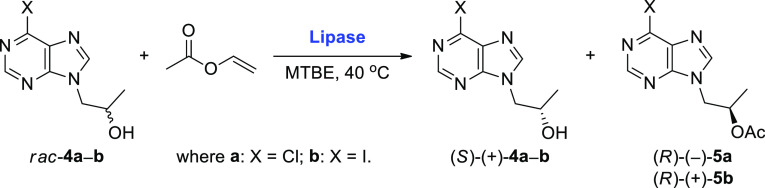
Lipase Screening for Enantioselective Transesterification of Racemic Alcohols *rac*-**4a** and -**4b**

entry	substrate^a^	lipase	*t* [h]	conv.^b^ [%]	ee_s_^c^ [%]	ee_p_^c^ [%]	*E*^d^
1	*rac*-**4a**	Novozym 435	16	64	59	33	3
2		Lipozyme 435	16	71	69	28	3
3		Chirazyme L-2, C-2	24	53	45	40	4
4		Chirazyme L-2, C-3	24	43	41	55	5
5		Novozym 435-STREM	16	72	75	29	4
6		CAL-B-Immobead 150	24	53	44	39	3
7		Chirazyme L-5	24	53	97	86	55
8		Amano PS-IM	24	51	>99	97	>200
9		Amano AK	24	27	37	98	142
10	*rac*-**4b**	Novozym 435	16	74	79	28	4
11		Lipozyme 435	16	65	76	41	5
12		Chirazyme L-2, C-2	24	58	59	42	4
13		Chirazyme L-2, C-3	24	36	38	67	7
14		Novozym 435-STREM	16	77	83	25	4
15		CAL-B-Immobead 150	24	60	65	43	5
16		Chirazyme L-5	24	20	24	95	49
17		Amano PS-IM	24	42	72	98	>200
18		Amano AK	24	11	12	95	44

a Conditions: *rac*-**4a** and -**4b** 0.12 mmol, lipase 5 mg (42 mg/mmol), MTBE 2 mL, vinyl acetate 1 mL (92 equiv), 40 °C, 800 rpm (magnetic stirrer).

b Conversion values (%) (i.e., consumption of substrate *rac*-**4a** and -**4b**) were determined by GC analyses after derivatization of crude mixture with BSA as a silylating reagent; for confirmation, the percent conversion was calculated from the enantiomeric excess of the unreacted alcohol (ee_s_) and the formed acetate (ee_p_) according to the formula conv. = ee_s_/(ee_s_ + ee_p_).

c Determined by HPLC analyses using columns packed with chiral stationary phases.

d Calculated according to Chen et al.^16^ using the equation: *E* = {ln[(1 – conv.)(1 – ee_s_)]}/{ln[(1 – conv.)(1 + ee_s_)]}.

The preliminary enzyme screening revealed that the best results in terms of the enantiomeric excess (ee) of the desired (*R*)-1-(6-halo-9*H*-purin-9-yl) propan-2-ols [(*R*)-(−)-**4a** and -**4b**] as well as the enantioselectivity (*E*) of the acetylation of *rac*-**4a** and -**4b** were obtained when the lipase from *Burkholderia cepacia* (BCL) immobilized on diatomaceous earth (Amano PS-IM) was employed as biocatalyst. This lipase preparation displayed *E* values of 348 in the case of 6-chloro derivative *rac*-**4a** and 214 in the case of 6-iodo derivative *rac*-**4b**, furnishing enantiomerically enriched acetates (*R*)-(−)-**5a** (97% ee) and (*R*)-(+)-**5b** (98% ee) with 51% and 42% conversion after 24 h (Table 1, entry 8 vs entry 17), respectively. Comparable optical purities were obtained with the native lipase from *Pseudomonas fluorescens* (Amano AK) (Table 1, entries 9 and 18), however with significantly lower conversion after 24 h. Hence, our attention was paid to Amano PS-IM. Furthermore, we decided to choose *rac*-**4a** for the subsequent optimization studies since the chloro substrate gave better results in terms of enantioselectivity and was obtained in one step less than iodo derivative *rac*-**4b**, thus simplifying the whole synthetic pathway toward tenofovir [(*R*)-(−)-**11**].

Once the suitable enzyme and substrate had been identified, optimization studies were continued to evaluate the effect of the organic cosolvents on Amano PS-IM as the rate and stereoselectivity of the enzymatic reactions as well as the thermal stability of the enzyme largely depend on the reaction medium^17^ (Table 2). For this purpose, 11 organic solvents of varying polarity (log *P* from −0.31 to 2.52) were investigated, including water-miscible polar solvents, such as 1,4-dioxane, acetonitrile (CH_3_CN), and acetone, or nonpolar solvents, such as toluene (PhCH_3_).

**Table 2:**
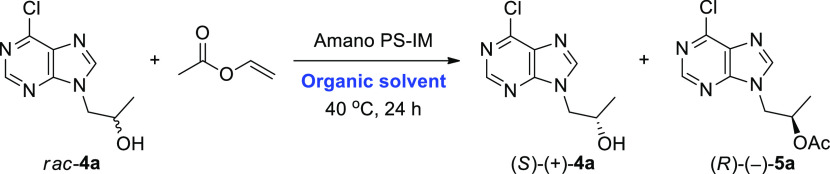
Cosolvent Screening for (Amano PS-IM)-Catalyzed KR of *rac*-**4a**

entry	cosolvent^a^ (log *P*)^b^	conv.^c^ [%]	ee_s_^d^ [%]	ee_p_^d^ [%]	*E*^e^
1	1,4-dioxane (−0.31)	20	25	99	>200
2	CH_3_CN (0.17)	12	14	>99	>200
3	acetone (0.20)	15	17	99	>200
4	EtOAc (0.29)	27	37	>99	>200
5	THF (0.40)	18	22	99	>200
6	MTBE (0.96)	47	87	98	>200
7	vinyl acetate (0.54)	38	60	99	>200
8	CH_2_Cl_2_ (1.01)	24	32	>99	>200
9	*tert*-amyl alcohol (1.09)	16	19	>99	>200
10	CHCl_3_ (1.67)	33	49	99	>200
11	PhCH_3_ (2.52)	45	81	99	>200

a Conditions: *rac*-**4a** 25 mg (0.12 mmol), Amano PS-IM 5 mg (42 mg/mmol), solvent 2 mL, vinyl acetate 1 mL (92 equiv), 24 h at 40 °C, 800 rpm (magnetic stirrer).

b Logarithm of the partition coefficient of a given solvent between *n*-octanol and water calculated using ChemBioDraw Ultra 13.0 software.

c Conversion values (%) (i.e., consumption of substrate *rac*-**4a**) were determined by GC analyses after derivatization of a crude mixture with BSA as a silylating reagent; for confirmation, the percent conversion was calculated from the enantiomeric excess of the unreacted alcohol (ee_s_) and the formed acetate (ee_p_) according to the formula conv. = ee_s_/(ee_s_ + ee_p_).

d Determined by HPLC analyses using columns packed with a chiral stationary phases.

e Calculated according to Chen et al.^16^ using the equation *E* = {ln[(1 – conv.)(1 – ee_s_)]}/{ln[(1 – conv.)(1 + ee_s_)]}.

The results showed that the enzymatic reaction gave higher conversions of the substrate (45–47%) in MTBE and PhCH_3_ than in other solvents (12–38%). In this regard, we observed the following rate trend: CH_3_CN ≈ acetone ≈ *tert*-amyl alcohol (2-methyl-2-butanol) < 1,4-dioxane ≈ THF < EtOAc ≈ CH_2_Cl_2_ < CHCl_3_ < vinyl acetate ≪ MTBE ≈ PhCH_3_. In turn, another order was observed for enantioselectivity: 1,4-dioxane ≈ CH_3_CN ≈ acetone ≈ EtOAc ≈ THF ≈ MTBE ≈ CH_2_Cl_2_ ≈ *tert*-amyl alcohol (*E* = 200–300) < CHCl_3_ ≈ vinyl acetate (*E* = 300–400) ≪ PhCH_3_ (*E* > 500). As can be noticed, no clear correlation between the log *P* value and the biocatalytic activity and the enantioselectivity was discerned. Considering the reaction rates and the values of the *E* factor, it was clear that the most promising results were obtained when Amano PS-IM was suspended in PhCH_3_, which is in line with the common rule that enzymes’ catalytic activity, stability, and enantioselectivity are generally improved in water-immiscible solvents with log *P* ≥ 2 due to negligible enzyme distortion in such media, suitable hydration of the protein molecule, and preservation of its active conformation.^18^ Therefore, toluene was chosen as a cosolvent for further optimization studies.

In the next step, the effect of the reaction time on the outcome of (Amano PS-IM)-catalyzed KR of *rac*-**4a** with vinyl acetate in PhCH_3_ was evaluated (for details, see Table S3 in Supporting Information). The KR assays were arbitrarily terminated at periodic time intervals between 4 and 30 h. Following the time course revealed that the most optimal compromise between the values of percent conversion and percent ee for the desired enantiomer (*R*)-(−)-**5a** is evident when the enzymatic reactions were terminated after 24 h. Although the elongation of time to 30 h led to improved conversion, the enantiomeric excess for the isolated acetate (*R*)-(−)-**5a** slightly decreased from >99% to 99%.

The temperature was studied to shorten the reaction times of the KRs as well as to investigate whether the immobilized enzyme can retain its enantioselectivity and stability at elevated temperatures (for details, see Table S4 in Supporting Information). The reactions were carried out at 40, 50, and 60 °C for 8 and/or 24 h for each temperature. When the lipase-catalyzed KR of *rac*-**4a** was performed at temperatures > 50 °C, the *E* values dropped to 226–371, which resulted in the isolation of the formed acetate (*R*)-(−)-**5a** in lower optical purities (96–98% ee).

The integration of the results obtained from the optimization studies showed that the most efficient biocatalyst was Amano PS-IM when used at 42 mg/mmol of *rac*-**4a** (2.5 mg/mL) and suspended in a solution of *rac*-**4a** (0.06 mmol/mL) in a mixture of PhCH_3_/vinyl acetate (3 mL, 2:1 v/v) as the reaction medium at 40 °C. In the next step, to evaluate the general applicability and versatility of the elaborated method on a preparative laboratory scale, enantioselective transesterification of *rac*-**4a** catalyzed by Amano PS-IM was scaled up to 500 mg of the racemic substrate (Table 3). Noteworthy, *rac*-**4a** was subjected to the optimized biocatalytic conditions with the preservation of the linear enlargement of all of the KR parameters, including substrate amount, enzyme concentration, volume of the acyl donor and solvent, etc. Arresting (Amano PS-IM)-catalyzed KR of *rac*-**4a** after 26 h (at 44% conversion) and purifying the crude reaction mixture using preparative silica-gel column chromatography afforded (*S*)-(−)-**4a** in 54% yield with 79% ee and (*R*)-(−)-**5a** in 47% yield with 99% ee (Table 3, entry 1).

**Table 3:**
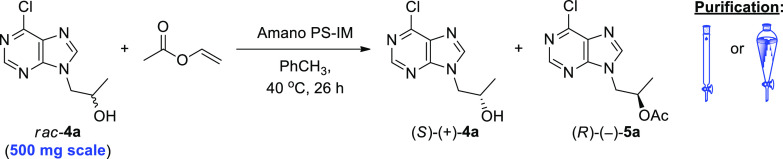
Preparative-Scale (Amano PS-IM)-Catalyzed KR of *rac*-**4a**

entry^a^	conv.^b^ [%]	ee_s_^c^ [%]/yield^d^ [%]/purity^e^ [%]	ee_p_^c^ [%]/yield^d^ [%]/purity^e^ [%]	*E*^f^
1	44^g^	79/54/>99	99/47/>99	>200
2	45^h^	82/48/92	99/31/>99	>200

a Conditions: *rac*-**4a** 500 mg, enzyme 100 mg, PhCH_3_ 40 mL, vinyl acetate 20 mL (92 equiv), 26 h at 40 °C, 800 rpm (magnetic stirrer).

b Conversion values (%) (i.e., consumption of substrate *rac*-**4a**) were determined by GC analyses after derivatization of crude mixture with BSA as a silylating reagent; for confirmation, the percent conversion was calculated from the enantiomeric excess of the unreacted alcohol (ee_s_) and the product (ee_p_) according to the formula conv. = ee_s_/(ee_s_ + ee_p_).

c Determined by HPLC analyses using columns packed with a chiral stationary phases.

d Isolated yield after column chromatography or liquid–liquid extractive workup.

e Chemical purity established using GC and/or HPLC methods.

f Calculated according to Chen et al.^16^ using the equation *E* = {ln[(1 – conv.)(1 – ee_s_)]}/{ln[(1 – conv.)(1 + ee_s_)]}.

g KR products were purified using SiO_2_-packed column chromatography.

h KR products were purified using liquid–liquid extractive workup.

Since we found that the KR products may conveniently be isolated by exploiting the solubility properties of the alcohol (*S*)-(−)-**4a** and acetate (*R*)-(−)-**5a** in the water–toluene biphasic mixture at room temperature, the enzymatic reaction was performed on a larger scale to obtain sufficient amount for handling (Table 3, entry 2). In this case, termination of KR of *rac*-**4a** after the same time as before furnished (*S*)-(−)-**4a** in 48% yield with 82% ee and (*R*)-(−)-**5a** in 31% yield with 99% ee. However, when employing liquid–liquid extractive (LLE) workup as a purification step, the yield of (*R*)-(−)-**5a** decreased due to a partial loss of the acetate, which was trapped in the water phase. Consequently, the recovered optically active alcohol (*S*)-(−)-**4a** contained 8% of acetate (*R*)-(−)-**5a**.

The absolute configuration of the KR products was determined by comparison with the reported optical rotations (for details, see Table S2 in Supporting Information).

### 2.2 *E. coli*/ADHs-Catalyzed Bioreduction of Ketones **3a** and **3b**

As the kinetic resolution methodology is limited by 50% reaction yields for the desired enantiomer and a cumbersome purification step, an alternative stereoselective transfer–biohydrogenation of prochiral ketones **3a** and **3b** was investigated to obtain (*R*)-(−)-**4a** and -**4b** in a more efficient manner (Table 4).

**Table 4:**
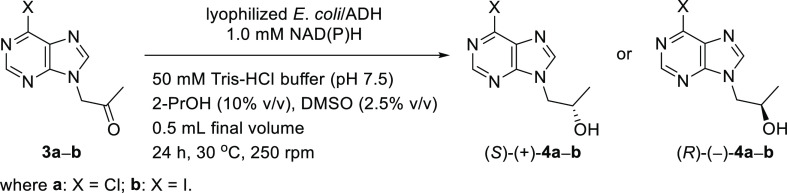
Analytical-Scale Studies on *E. coli*/ADHs-Catalyzed Stereoselective Reduction of Ketones **3a** and **3b**

			*nonrac*-**4a**		*nonrac*-**4b**	
entry	biocatalyst^a^	cofactor	conv.^b^ [%]	ee_p_^c^ [%] (config.^d^)	conv.^b^ [%]	ee_p_^c^ [%] (config.^d^)
1	*E. coli*/TeSADH	NADPH	N.D.^e^	N.A.^f^	N.D.^e^	N.A.^f^
2	*E. coli*/ADH-TH	NADPH	33	95/(*R*)	N.D.^e^	N.A.^f^
3	*E. coli*/ReADH	NADH	63	99/(*S*)	47	>99/(*S*)
4	*E. coli*/RasADH	NADPH	90	90/(*S*)	66	86/(*S*)
5	*E. coli*/SyADH	NADPH	54	41/(*S*)	26	50/(*S*)
6	*E. coli*/ADH-A	NADH	>99	>99/(*S*)	>99	>99/(*S*)
7	*E. coli*/LB-ADH	NADPH	21	74/(*R*)	N.D.^e^	N.A.^f^
8	*E. coli*/Lk-ADH-Lica	NADPH	>99	99/(*R*)	>99	>99/(*R*)
9	*E. coli*/Lk-ADH	NADPH	89	98/(*R*)	55	87/(*R*)
10	*E. coli*/Lk-ADH Prince	NADPH	>99	>99/(*R*)	>99	99/(*R*)
11	*E. coli*/PpADH	NADH	50	32/(*S*)	11	89/(*S*)

a Reaction conditions: **3a** or **3b** (10 mM final concentration), lyophilized biocatalyst (10 mg), NAD(P) H (1 mM final concentration), 50 mM Tris-HCl buffer (pH 7.5)/2-PrOH (500 μL, 90:10, v/v), DMSO (2.5% v/v), 24 h at 30 °C, 250 rpm (laboratory shaker).

b Conversion values (%) (i.e., consumption of substrates **3a** and **3b**) were determined by GC analyses after derivatization of crude mixture with BSA as a silylating reagent.

c Determined by HPLC analyses using columns packed with chiral stationary phases.

d Absolute configuration of optically active products (*nonrac*-**4a** and -**4b**) established by comparing HPLC picks elution order with enantiomeric standards. The major enantiomer is shown in parentheses.

e Not detected.

f Not applicable because of no detectable conversion.

Prior to alcohol dehydrogenase-catalyzed bioreduction studies, silylation of the polar hydroxyl groups in alcohols *rac*-**4a** and -**4b** using the standard derivatization protocol employing *N,O*-bis(trimethylsilyl) acetamide (BSA) in CH_2_Cl_2_ had to be applied to obtain more volatile compounds characterized by much lower values of the retention times (*t*_R_) than ketones **3a** and **3b**. As we needed trimethylsilyl ethers *rac*-**6a** and -**6b** in more significant amounts to proceed with calibration curves for GC analyses, the preparative scale for the silylation reaction was also performed, furnishing both derivatives in the range of 83–87% yield (after column chromatography) (Scheme 1).

An initial biocatalyst screening was attempted using lyophilized *Escherichia coli* cells harboring recombinantly overexpressed alcohol dehydrogenases (denoted as *E. coli*/ADHs) originating from various microorganisms, including *Thermoanaerobacter pseudoethanolicus* ATCC 33223 (*E. coli*/TeSADH^19^), *Thermoanaerobacter ethanolicus* (*E. coli*/ADH-TH^20^), *Rhodococcus erythropolis* DSM 43297 (*E. coli*/ReADH^21^), *Ralstonia* sp. DSM 6428 (*E*. *coli*/RasADH^22^), *Sphingobium yanoikuyae* DSM 6900 (*E*. *coli*/SyADH^23^), *Rhodococcus ruber* DSM 44541 (*E*. *coli*/ADH-A^24^), *Lactobacillus brevis* (*E*. *coli*/LB-ADH^25^), *Lactobacillus kefir* (*E*. *coli*/Lk-ADH-Lica,^26^*E*. *coli*/LkADH,^27^*E*. *coli*/Lk-ADH Prince^4b,4c^), and *Paracoccus pantotrophus* DSM 11072 (*E*. *coli*/PpADH^28^).

The *E. coli*/ADH-catalyzed bioreductions of both prochiral ketones **3a** and **3b** were carried out under standard biocatalytic conditions using a 10 mM concentration of the respective substrate and 10 mg of *E. coli* cells of each biocatalyst suspended in 50 mM Tris-HCl buffer (pH 7.5) in the presence of 1.0 mM of the external nicotinamide cofactor [NADH or its phosphate counterpart NADPH] for 24 h at 30 °C. In addition, to ensure complete solubility of the organic substrates **3a** and **3b** in the reaction media and enhance the in situ regeneration of the NAD(P)H cofactors, we have supplemented the reaction mixtures with 2.5% v/v of dimethyl sulfoxide (DMSO) and 10% v/v of propan-2-ol (2-PrOH), respectively. Notably, due to the high solubility of the products **4a** and **4b** in an aqueous medium, all of the enzymatic reactions were terminated by removal of the cells under suction and subsequent azeotropic evaporation of the water with toluene from permeate to eliminate an undesired drop in the product yield (for details, see Supporting Information).

To our delight, the biocatalytic reduction of **3a** and **3b** revealed that from the tested biocatalysts, those *E. coli* cells which contained the ADH variants derived from *L. kefir* (*E. coli*/Lk-ADH-Lica and *E. coli*/Lk-ADH Prince) allowed obtaining the desired optically pure (*R*)-(−)-**4a** and -**4b** (>99% ee) with >99% conversion. In turn, *E. coli*/ADH-A led to the formation of enantiomerically pure antipodes (*S*)-(+)-**4a** and **4b** (>99% ee) with quantitative conversions (>99%). Noteworthy, due to the opposite stereopreference of the examined whole-cell biocatalysts toward prochiral ketones **3a** and **3b**, both enantiomers of a key intermediate could be synthesized in simultaneous attempts.

**Scheme 2:**
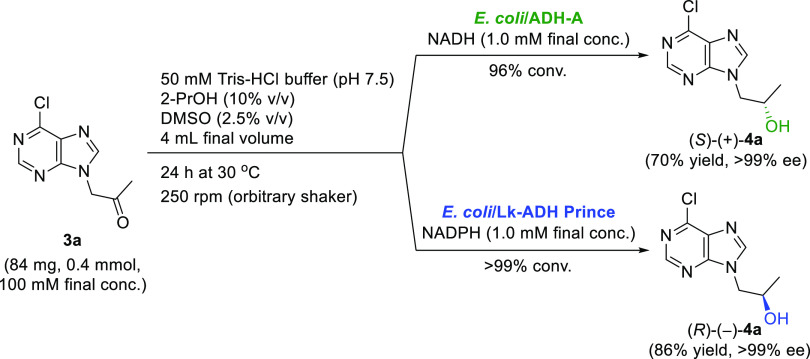
Stereocomplementary Bioreductions of **3a** Catalyzed by *E. coli*/ADH-A or *E. coli*/Lk-ADH Prince

After successfully screening the ADH biocatalysts, our next task was to scale up the asymmetric bioreduction of ketones **3a** and **3b** utilizing the stereocomplementary ADH preparations exhibiting either the Prelog selectivity (*E. coli*/ADH-A) or the anti-Prelog selectivity (*E. coli*/Lk-ADH Prince) (Scheme 2). Taking advantage of the chloro derivative **3a** over iodo derivative **3b** (i.e., the higher yield and simplicity of its synthesis due to a lesser number of steps and commercial availability of the reagents), we decided to use **3a** as the model substrate exclusively. The biotransformations were performed with 0.4 mmol of **3a** in 4 mL of the final volume of the reaction mixture, which resulted in a 10-fold higher substrate concentration (100 mM) than on an analytical scale. By utilizing *E. coli*/ADH-A, the asymmetric bioreduction of **3a** furnished (*S*)-(+)-**4a** in 70% yield and with >99% ee. In contrast, when *E*. *coli*/Lk-ADH Prince was used as a biocatalyst, (*R*)-(−)-**4a** was isolated in 86% yield and with >99% ee. Once again, to avoid drawbacks with the high solubility of the products (*R*)-(−)-**4a** and (*S*)-(+)-**4a** in an aqueous buffer solution, we employed an alternative workup procedure, which excluded direct liquid–liquid extraction of the water phase with organic solvents. This modification not only allowed us to obtain the appropriate products (*R*)-(−)-**4a** and (*S*)-(+)-**4a** in higher yields but also generated a more sustainable protocol for their isolation by decreasing the amount of the used solvents during the workup.

It was clear that the elaborated biocatalytic methodology can be amplified to a larger scale and thus outperformed the above-presented lipase-catalyzed KR of *rac*-**4a** and -**4b** (in terms of the product yield) as well as other literature reports^14^ (in terms of the enantiomeric purity of (*R*)-(−)-**4a**), requiring for asymmetric transfer hydrogenation of prochiral ketones **3a** and **3b** ruthenium catalysts. In this regard, ADH-catalyzed transformation, utilizing more benign catalysts and sustainable reaction conditions, might be relevant for industry, paving the way for more green technologies.

### 2.3 Molecular Docking

**Figure 2:**
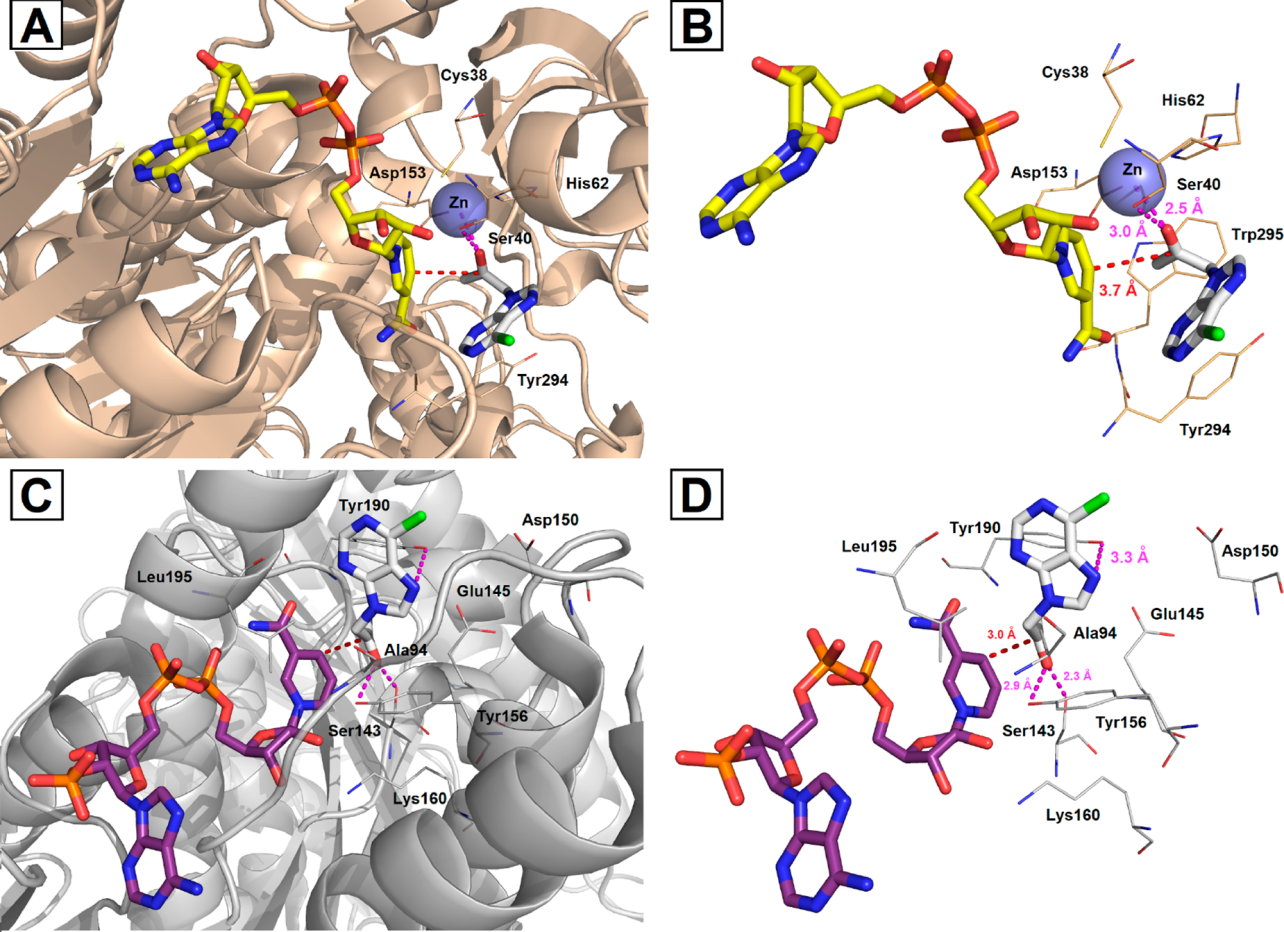
Representative three-dimensional (3D) binding modes of 1-(6-chloro-9*H*-purin-9-yl) propan-2-one (**3a**) with stereocomplementary alcohol dehydrogenases, namely, ADH-A (PDB code: 2XAA; A and B) and Lk-ADH (PDB code: 4RF2; C and D), with close contacts to amino acid residues and cofactors located in the active sites. The docked ligand **3a** and the cofactors are shown as sticks representation, where **3a** is white, NADH is yellow, and NADPH is violet. The overall receptor structures are shown as a semitransparent cartoon diagram (left; A and C), where ADH-A is wheat and Lk-ADH is gray. The most significant amino acid residues contributing to the stabilization of the ligand **3a** molecule in the complex with ADH-A or Lk-ADH by polar interactions, alkyl–alkyl (CH–CH), van der Waals (vdW,) and/or π–alkyl (CH−π) interactions are shown in lines representations. Nitrogen atoms are shown in blue, oxygen atoms in red, chlorine atoms in green, and phosphorus atoms in orange. All hydrogens were omitted for clarity. The zinc ion is presented as a semitransparent slate sphere. The formation of intermolecular hydrogen bonds is represented by magenta dashed lines, whereas the plausible trajectory of the hydride transfer from cofactors to a carbon atom of the carbonyl group is shown as red dashed lines. Mutual distances between the amino acid residues and the respective ligand’s atoms are given in Ångströms (right column; B and D). The figure was prepared using the program PyMOL (http://www.pymol.org/).

In order to rationalize the stereoselectivity of the ADH-catalyzed biotranshydrogenation of 1-(6-chloro-9*H*-purin-9-yl) propan-2-one (**3a**), comprehensive in silico enzyme–substrate docking calculations were performed. For this purpose, prochiral ketone **3a** was docked with receptor molecules prepared based on the crystal structures of both stereocomplementary ADHs, namely, ADH-A from *Rhodococcus ruber* DSM 44541 (PDB code: 2XAA)^29^ and Lk-ADH from *L. kefir* (PDB code: 4RF2),^30^ which were retrieved from the Protein Data Bank (PDB) database (http://www.rcsb.org/pdb/). As a result of molecular docking experiments, nine of the most energetically favorable binding modes for the ligand–protein complexes for ligand **3a** molecule and target ADH-A and Lk-ADH proteins were generated. The visualization of the representative docking poses of **3a** to ADH-A and Lk-ADH (taken from the lowest energy docking clusters) with close contacts to amino acid residues located in the corresponding active sites of the studied ADHs is presented in Figure 2.

Careful inspection of the productive pose of **3a** in ADH-A (Figure 2A and 2B) showed that this ketone forms strong 2.5 Å long metal–acceptor interactions with the catalytic zinc ion and a 3.0 Å long hydrogen bond with the hydroxyl group of the side chain of the Ser40 residue present in the active site. At the same time, the 6-chloro-9*H*-purine moiety of **3a** is located outside the small substrate-binding pocket, which allows ligand **3a** to avoid unfavorable steric clashes of the larger substituent with amino acid residues located more profoundly in the catalytic cavity (i.e., Tyr294, Trp295, Asp153, His62, Cys38, Ser40, etc.). In addition, the purine moiety is bound in the broader part of the active site cavity pointing toward the exterior amino acids (i.e., Phe43, Phe282, Ile271) within which **3a** forms π–π and π–alkyl (CH−π) interactions (see Figure S3A in Supporting Information). The second plausible explanation of the ligand accommodation is that the purine moiety introduces a steric hindrance due to the proximity of the catalytic zinc, which consequently pushes the substrate out of this region. Of note, the additional π–σ interactions between the methyl substituent of **3a** and the indole ring of Trp295 stabilize a hypothetical complex of **3a**–ADH-A such that the carbonyl oxygen atom of the substrate **3a** forces the location of the carbon atom of the carbonyl group to be in close proximity to the C4 atom of the NADH cofactor. As a consequence of this orientation, the hydride is transferred from the cofactor onto the *re* face of the carbonyl group of **3a** following the well-known (*S*)-stereoselectivity of ADH-A.

In turn, a complex of Lk-ADH and the selected top-scoring binding mode of **3a** (Figure 2C and 2D) confirmed that the ligand molecule is accommodated inside the binding cleft with a pose that promotes the (*R*)-stereoselective ketone reduction pathway. When analyzing in silico simulations, one can see that the confined environment of the Lk-ADH active site orients the 6-chloro-9*H*-purine moiety perpendicular to the cofactor, forcing the methyl group to locate into the smaller binding pocket in close proximity toward the catalytic triad (Ser143-Tyr156-Lys160). In addition, two strong 2.3–2.9 Å long hydrogen bonds formed between the oxygen atom of the prochiral carbonyl group of **3a** and the amino acid residues involved in the proton relay (Ser143 and Tyr156) favored the orientation of the ligand with the *si* face exposed toward the reactive hydride of the NADPH cofactor. Moreover, the docking of **3a** with Lk-ADH revealed that the imidazole ring of the purine moiety formed H-bond interactions with the phenol group of Tyr190. On the other hand, this functionality also establishes π–anion interactions with Glu145. Interestingly, the additional π–alkyl interactions between the purine ring and the side chains of the aliphatic amino acid residues (i.e., Ala202, Val196, Leu153) were found to be beneficial for the stabilization of the ligand–protein complex in such a way that anti-Prelog product (*R*)-(−)-**4a** could be obtained within this biocatalyst (see Figure S3B in Supporting Information). Noteworthy, all of the docking simulations are in line with the experimental results.

### 2.4 Synthesis of Enantioenriched Tenofovir [(*R*)-(−)-**11**]

**Scheme 3:**
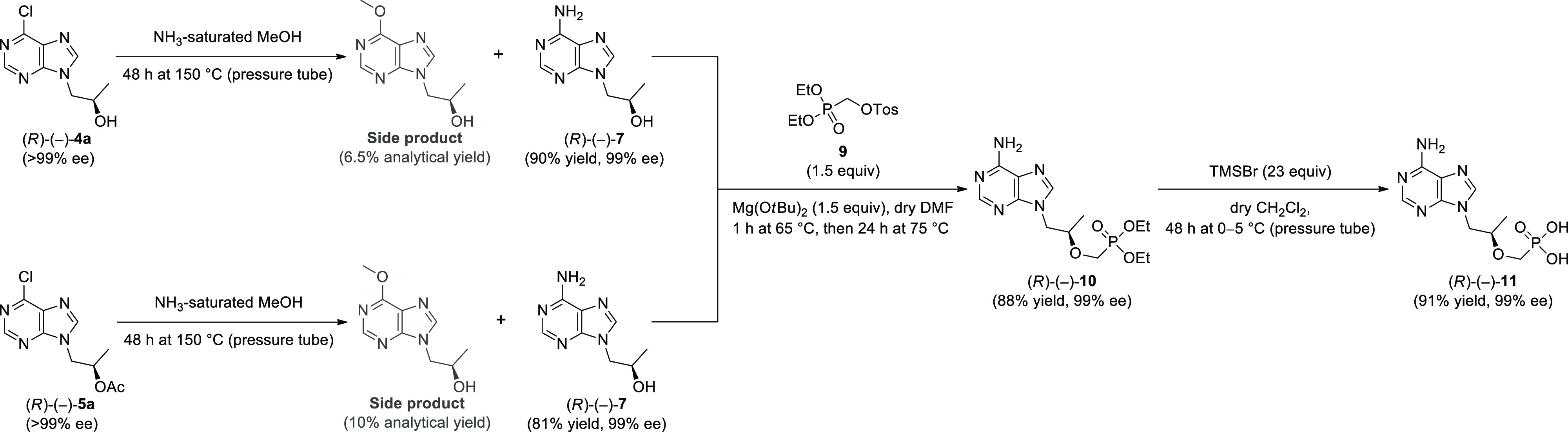
Transformation of (*R*)-1-(6-Chloro-9*H*-purin-9-yl) Propan-2-ol [(*R*)-(−)-**4a**] and/or (*R*)-1-(6-Chloro-9*H*-purin-9-yl) Propan-2-yl Acetate [(*R*)-(−)-**5a**] into Tenofovir [(*R*)-(−)-**11**]

The last task was to functionalize optically active alcohol (*R*)-(−)-**4a** into tenofovir [(*R*)-(−)-**11**] (Scheme 3). The primary goal of these studies was to develop synthetic procedures that would lead to (*R*)-(−)-**11** in high total yield and excellent enantiomeric excess. Besides the motivations mentioned above, the assumption of employing the shortest possible synthetic pathway leading to less waste generation was also considered.

Initially, we conducted experiments toward obtaining (2*R*)-1-(6-amino-9*H*-purin-9-yl) propan-2-ol [(*R*)-(−)-**7**]. To achieve this goal, direct amination of (*R*)-(−)-**4a** was performed using an ammonium-saturated solution of methanol. The reaction was carried out for 48 h at 150 °C in a pressure tube, furnishing (*R*)-(−)-**7** in 90% yield with 99% ee. Interestingly, the synthesis of (*R*)-(−)-**7** was also accomplished via one-pot two-step amination–hydrolysis of the acetate (*R*)-(−)-**5a** obtained from the lipase-catalyzed KR route. However, in this case, optically active 6-amino derivative (*R*)-(−)-**7** (99% ee) was isolated in a slightly lower yield (81%). Unfortunately, both variants of the investigated reaction generated undesired (2*R*)-1-(6-methoxy-9*H*-purin-9-yl) propan-2-ol. Nevertheless, the amounts of a 6-methoxy byproduct were negligible and did not exceed 6.5% analytical yield (determined by GC) in the case of the reaction utilizing (*R*)-(−)-**4a** as the substrate and 10% in the case of the reaction carried out with (*R*)-(−)-**5a**.

In a subsequent attempt, we performed Mg(O^*t*^Bu)_2_-mediated *O*-alkylation of the resulting (*R*)-(−)-**7** with 1.5 equiv of tosylated diethyl (hydroxymethyl) phosphonate (**9**) in analogy to the method reported by Barral et al.^31^ The reaction was carried out in dry dimethylformamide (DMF) for 24 h at 75 °C and led to obtaining the respective diethyl phosphonate ester (*R*)-(−)-**10** in 88% yield and with 99% ee. In the original procedure, the authors used room temperature and sodium base NaO^*t*^Bu instead of the magnesium one Mg(O^*t*^Bu)_2_ and obtained (*R*)-(−)-**10** in only a 29% yield. Our result proved the putative role of the Mg^2+^ counterion as well as an elevated temperature on the outcome of the alkylation reaction.

The final deprotection of phosphonate ester (*R*)-(−)-**10** was performed according to the procedure reported by Maghami et al.^15a^ In this regard, the treatment of (*R*)-(−)-**10** with trimethylsilyl bromide (TMSBr) in dry CH_2_Cl_2_ for 48 h at 0–5 °C (performed in a pressure tube) allowed us to afford free phosphonic acid (*R*)-(−)-**11** in 91% yield and without loss of optical purity (99% ee).

Taking into account both chemoenzymatic routes, the resulting tenofovir [(*R*)-(−)-**11**] was synthesized in 13.3% total yield when following the lipase-catalyzed KR methodology (6 steps) and in 35.9% total yield when following the *E. coli*/ADH-catalyzed bioreduction (5 steps) pathway. Compared with the reported methods,^8−12,14^ our approach is more straightforward and efficient in terms of the synthesis of optically active tenofovir [(*R*)-(−)-**11**]. In addition, since we have elaborated the lipase-catalyzed KR of tenofovir’s key intermediate as well as found efficient stereocomplementary ADHs [(*S*)-selective *E. coli*/ReADH and *E. coli*/ADH-A vs (*R*)-selective *E. coli*/Lk-ADH-Lica and *E. coli*/Lk-ADH Prince) toward prochiral ketones, which allow obtaining the (*S*)-enantiomer; this method can be used in the synthesis of tenofovir enantiomeric counterpart, potentially useful as an impurity standard for pharmacological investigations.

## 3 Conclusions

Here, we report straightforward chemoenzymatic synthesis routes for the facile preparation of tenofovir in an optically pure form, starting from simple and inexpensive commercially available reagents as well as biodegradable enzyme preparations. The elaborated methodology involves a highly stereoselective bioreduction of prochiral 1-(6-chloro-9*H*-purin-9-yl) propan-2-one catalyzed by a (*R*)-specific ADH variant from *L. kefir* (*E. coli*/Lk-ADH Prince) and/or enantioselective transesterification of racemic 1-(6-chloro-9*H*-purin-9-yl) propan-2-ol with vinyl acetate in the presence of immobilized lipase from *B. cepacia* (Amano PS-IM) carried out under kinetically controlled conditions. The ADH-based methodology furnished (*R*)-1-(6-chloro-9*H*-purin-9-yl) propan-2-ol in high isolated yield (86%) and excellent enantiomeric purity (>99% ee). In contrast, when following the lipase-catalyzed KR strategy, the desired optically active (*R*)-precursor (99% ee) was obtained in 47% yield after preparative chromatography or in 31% yield after liquid–liquid extractive workup without employing chromatographic purification. The key biocatalytic steps were combined with a convenient “aminolysis–hydrolysis–alkylation–deprotection” reaction sequence to convert the enzymatically generated (*R*)-intermediate into the desired tenofovir active agent in good overall isolated yield (up to 72%) and excellent optical purity (99% ee) after 3 steps. Comparing both investigated chemoenzymatic routes, the one which utilized *E. coli*/ADH-catalyzed bioreduction of 1-(6-chloro-9*H*-purin-9-yl) propan-2-one turned out to be more efficient since tenofovir has been synthesized in 35.9% total yield after 5 steps. This approach offers an attractive alternative to the already reported catalytic methods and can potentially serve in tenofovir manufacturing, as well as in the synthesis of other pharmaceutically relevant acyclic nucleoside phosphonates.

## 4 Experimental Section

### 4.1 Representative Biocatalytic Procedures

#### 4.1.1 General Procedure for Preparative Scale (Amano PS-IM)-Catalyzed KR of *rac*-**4a**

##### 4.1.1.1 Method A (Ended with Purification via Silica-Gel Column Chromatography)

The reaction mixture containing racemic 1-(6-chloro-9*H*-purin-9-yl) propan-2-ol (*rac*-**4a**, 500 mg, 2.4 mmol, 60 mM final concentration), PhCH_3_ (40 mL), vinyl acetate (18.62 g, 216 mmol, 20 mL), and Amano PS-IM (100 mg) was stirred (800 rpm, IKA RCT basic) in an Ace round-bottom pressure flask with an Ace-Thred 15 PTFE front-seal plug (capacity 250 mL; Sigma-Aldrich no. Z567191) for 26 h at 40 °C. Next, the enzymatic reaction was stopped by filtering off the lipase on a Schott funnel under a vacuum and by washing the biocatalyst preparation with a portion of PhCH_3_ (20 mL). After evaporation of the volatiles, the crude oil was purified by silica gel column chromatography using a mixture of CH_2_Cl_2_/MeOH (95:5, v/v) to provide the corresponding EKR products as follows: (*S*)-(+)-**4a** (269 mg, 1.3 mmol, 54% yield, 79% ee, >99% purity) and (*R*)-(−)-**5a** (282 mg, 1.1 mmol, 47% yield, 99% ee, >99% purity) as a white solids. For details, see Table 3.

##### 4.1.1.2 Method B (Ended with Purification via Liquid–Liquid Extraction)

All of the manipulations, except the workup and purification procedures, were carried out by analogy with the above protocol. To isolate the EKR products, the crude reaction mixture was filtered off from the lipase preparation, and the filtrate cake was rinsed with a portion of PhCH_3_ (20 mL). After evaporation of the volatiles, the crude oil was dissolved in PhCH_3_ (40 mL) and washed with H_2_O (3 × 20 mL). The combined organic layer was dried over MgSO_4_, the drying agent was filtered off, and the permeate was concentrated under reduced pressure to provide (*R*)-(−)-**5a** (187 mg, 0.7 mmol, 31% yield, 99% ee, >99% purity) as a white solid. The combined aqueous phase was back-extracted with PhCH_3_ (3 × 40 mL) to remove traces of (*R*)-(−)-**5a**. Afterward, an aqueous layer was azeotropically condensed with PhCH_3_ (100 mL). The resulting oil residue was diluted with AcOEt (40 mL) and dried over MgSO_4_, and after filtering off the drying agent and evaporation of the volatile solvents, the desired (*S*)-(+)-**4a** (240 mg, 1.1 mmol, 48% yield, 82% ee, 92% purity) was obtained as a white solid. For details, see Table 3.

#### 4.1.2 General Procedure for Preparative-Scale Bioreduction of **3a** Using *E. coli*/ADH-A or *E. coli*/Lk-ADH Prince

*E. coli*/Lk-ADH-A or *E. coli*/Lk-ADH Prince (60 mg) was suspended in 50 mM Tris–HCl buffer (3.5 mL; pH 7.5) containing NADH (2.86 mg, 1.0 mM final concentration in the case of *E. coli*/ADH-A) or NADPH (3.33 mg, 1.0 mM final concentration in the case of *E. coli*/Lk-ADH Prince) and preincubated for 30 min at 30 °C with gentle shaking. Then, 1-(6-chloro-9*H*-purin-9-yl) propan-2-one (**3a**, 84 mg, 0.4 mmol, 100 mM final concentration) and 2-PrOH (400 μL, 10% v/v) supplemented with DMSO (100 μL, 2.5% v/v) were added to the mixture. The reaction was shaken at 30 °C and 250 rpm for 24 h. After incubation, the enzymatic reaction was stopped by filtering off the cells under a vacuum and rinsing the filtrate cake with PhCH_3_ (15 mL). Next, the water was azeotropically evaporated from the permeate, and the crude oil residue was purified by silica gel column chromatography using a mixture of CH_2_Cl_2_/MeOH (95:5, v/v), thus obtaining the desired optically active product (*S*)-(+)-**4a** (60 mg, 0.28 mmol, 70% yield, >99% ee, in the case of *E. coli*/ADH-A) or (*R*)-(−)-**4a** (73 mg, 0.34 mmol, 86% yield, >99% ee, in the case of *E. coli*/Lk-ADH Prince) as a white solid.

## Data Availability

The data underlying this study are available in the published article and its Supporting Information.

## Supporting Information Available

Supplementary tables and figures; general experimental methods and synthetic procedures; compound characterization data; molecular docking data; GC and HPLC analytical separation conditions, copies of chiral HPLC chromatograms, and ^1^H NMR, ^13^C{^1^H} NMR, ^31^P{^1^H} NMR, IR, and FTMS spectra (PDF)
FAIR data, including the primary NMR FID files, for compounds **2, 3a**, **3b**, *rac*-**4a**, *rac*-**4b**, *rac*-**5a**, *rac*-**5b**, *rac*-**6a**, *rac*-**6b**, (*R*)-(−)-**7**, **9**, (*R*)-(−)-**10**, and (*R*)-(−)-**11** (ZIP)

## Dedication

Dedicated to Czesław Wawrzeńczyk on the occasion of his 80th birthday.
