# Supplementary material for: Chemoenzymatic Synthesis of Tenofovir
Source: J Org Chem. 2023 Jul 19;88(15):11045–55. doi: 10.1021/acs.joc.3c01005 (PMC10407936; doi:10.1021/acs.joc.3c01005)
Supplement: Supplementary file 1 — jo3c01005_si_001.pdf [file jo3c01005_si_001.pdf]

Supporting Information

for

Chemoenzymatic Synthesis of Tenofovir

Beata Zdun,[a] Tamara Reiter,[b] Wolfgang Kroutil,[b] and Paweł Borowiecki[a,]*

[a] Laboratory of Biocatalysis and Biotransformation, Department of Drugs Technology and Biotechnology, Faculty of Chemistry, Warsaw University of Technology, Faculty of Chemistry, Koszykowa St. 75, 00-662 Warsaw, Poland.

[b] Institute of Chemistry, University of Graz, NAWI Graz, BioTechMed Graz, Field of Excellence BioHealth, Heinrichstrasse 28, 8010 Graz, Austria.

*Corresponding author. Dr. Paweł Borowiecki (Email: pawel.borowiecki@pw.edu.pl; Website: http://lbb-wut-borowiecki.ch.pw.edu.pl/)

Table of contents

1. General experimental methods ………………………………………..……………….. S2–S3

2. Synthetic procedures and analytical data for the obtained compounds ……………… S4–S15

3. **Table S1**. List of commercial enzyme preparations employed in these studies …..……... S16

4. **Table S2**. The results of specific rotation values for the optically active products …..….. S17

5. **Table S3**. Studies on the reaction time-course of (Amano PS-IM)-catalyzed KR of *rac*-**4a** with vinyl acetate in $PhCH_3$ at 40 °C …………………………………………..………... S18

6. **Table S4**. Temperature effect on (Amano PS-IM)-catalyzed KR of *rac*-**4a** with vinyl acetate in $PhCH_3$ after 8 h and 24 h ………………………………………………………..….. S18

7. Molecular docking ………..……………………………………………………..….. S19–S24

8. **Table S5.** Docking scoring of 1-(6-chloro-9*H*-purin-9-yl)propan-2-one (**3a**) complexed with alcohol dehydrogenases ……………………………………………………………………. S24

9. **Table S6**. Analytical separation conditions of racemic compounds by GC column …….. S25

10. **Table S7**. HPLC analytical separation conditions of purine derivatives by chiral columns – Chiralcel OD-H or Chiralcel OJ-H or Chiralpak AD-H …………..…………………….…... S26

11. Analytical data (copies of HPLC chromatograms) ……………...................…… S27–S97

12. Spectral data (copies of NMR, IR, and FTMS spectra) ……................................ S98–S126

13. References …..…………………………………………………...………..……...... S127

1. General experimental methods.

β-Nicotinamide adenine dinucleotide, disodium salt, hydrate, 95+%, reduced form (NADH) was purchased from Across Organics (Cat. No.: 271100010); β-nicotinamide adenine dinucleotide 2′-phosphate reduced tetrasodium salt (NADPH) was purchased from AmBeed (Cat. No.: A341469); 6-chloropurine was purchased from Fluorochem Ltd (Cat. No.: 032518); diethyl (hydroxymethyl)phosphonate was purchased from TCI (Cat. No.: D3813). Chromatography grade *n*-hexane and 2-propanol (2-PrOH) used in high-performance liquid chromatography (HPLC) were purchased from Avantor Performance Materials Poland S.A. (formerly POCH Polish Chemicals Reagents). All other commercially available reagents [purchased from Merck KGaA, (Darmstadt, Germany), TCI (Tokyo Chemical Industry), Thermo Fisher (Kandel) GmbH (Kandel, Germany), and Fluorochem Ltd (Hadfield Derbyshire, United Kingdom)] were used without further purification.

The commercial enzyme preparations were purchased from Novozymes A/S (Bagsvaerd, Denmark), STREM Chemicals, Inc. (Newburyport, MA, USA), Amano Pharmaceutical Co., Ltd. (Nagoya, Japan), Sigma-Aldrich (currently Merck) (Darmstadt, Germany), Roche (Basel, Switzerland), Boehringer Mannheim (currently Roche Diagnostics) (Basel, Switzerland), and were used without pre-treatment (for details, see **Table S1** appended in Supporting Information).

Analytical scale enzymatic reactions were performed in thermo-stated glass vials (V = 4 mL) placed in Chemglass CG-1991-04 GOD Anodized Aluminum Reaction Block, 48 Position, 19 mm Hole Depth, For Circular Top Hot Plate Stirrer. Preparative-scale lipase kinetic resolutions were carried out in an Ace round-bottom pressure flask with Ace-Thred 15 PTFE front-seal plug (capacity 250 mL; Sigma Aldrich No.: Z567191). All non-aqueous reactions were carried out under oxygen-free (argon-protective) conditions using over-dried glassware.

Analytical thin-layer chromatography was carried out on TLC aluminum plates with silica gel Kieselgel 60 F_{254} (Merck, Germany) (0.2 mm thickness film containing a fluorescence indicator green 254 nm (F254) using UV light as a visualizing agent.

Preparative separations were carried out by (i) column chromatography using Merck silica gel 60 (230–400 mesh), with grain size 40–63 µm, or by (ii) PLC PSC-Fertigplatten Kieselgel 60 F_{254} (20 × 20 cm with 2 mm thickness layer) glass plates purchased from Merck, (Darmstadt, Germany).

The solvent residues were evaporated at reduced pressure using a Büchi rotary evaporator and a high-vacuum oil pump at p = 0.05 mmHg.

Melting point (mp) ranges, uncorrected, were determined with a commercial apparatus (Thomas-Hoover "UNI-MELT" capillary melting point apparatus) on samples contained in rotating capillary glass tubes open on one side (1.35 mm inner diam. and 80 mm length).

The gas chromatography (GC) analyses were performed with an Agilent Technologies 6890N instrument (Maryland, United States) equipped with a flame ionization detector (FID) and fitted with HP-50+ (30 m) semi-polar column (50 % phenyl – 50 % methylpolysiloxane); the GC injector was maintained at 250 °C; Helium (2 mL/min) was used as carrier gas; retention times (t_R) are given in minutes under these conditions; column temperature programs are given in **Table S6** appended in Supporting Information.

The enantiomeric excesses (% ee) of optically active compounds were determined by high performance liquid chromatography (HPLC) analyses performed on Shimadzu Nexera-*i* (LC-2040C 3D) equipped with a photodiode array detector (PAD) using Chiralpak AD-H, Chiralcel OD-H or Chiralcel OJ-H (4.6 mm × 250 mm, coated on 5 µm silica gel grain size) chiral columns (Daicel Chemical Industries Ltd., Japan) equipped with dedicated pre-columns (4 mm × 10 mm, 5 µm); the respective mixtures of *n*-hexane/2-PrOH or *n*-hexane/2-PrOH/diethylamine (DEA) were used as mobile phases in the appropriate ratios; the HPLC analyses were executed in an isocratic and isothermal (30 °C) manner; flow (*f*) is given in mL/min; racemic compounds were used as standards; HPLC conditions and retention times (t_R) are given in **Table S7** appended in Supporting Information.

Optical rotations ([α]) were measured with a PolAAr 32 polarimeter in a 2 dm long cuvette using the sodium D line (589 nm) at 30 °C; $[\alpha]_D$ are given in units of deg dm^{-1} cm^3 g^{-1}; the concentration *c* is in g/100 mL (for details, see **Table S2** appended in Supporting Information).

^1H NMR, $^{13}C\{^1H\}$ NMR, and $^{31}P\{^1H\}$ spectra were recorded on a Spectrometr Varian NMR System 500 MHz (Varian, Inc., Palo Alto, CA, USA)]; chemical shifts (δ) are given in parts per million (ppm) on the delta scale related to the solvent peak used as reference value; signal multiplicity assignment: s, singlet; d, doublet; t, triplet; q, quartet; m, multiplet; coupling constant (J) are given in hertz (Hz); all samples were recorded as solutions in fully deuterated chloroform ($CDCl_3$), dimethylsulfoxide (DMSO-d_6), and deuterium oxide (D_2O), respectively. All NMR reports for Supporting Information were created by ACD/NMR Processor Academic Edition 12.0. (Freeware software provided by ACD/Labs, USA & Canada).

Fourier Transform Mass Spectrometry (FTMS) was recorded on Q Exactive Hybrid Quadrupole-Orbitrap Mass Spectrometer, ESI source: electrospray with spray voltage 4.00 kV; all samples were prepared by dilution with MeOH (0.5 mL) and addition of a mixture of CH_3CN/MeOH/H_2O (50:25:25, v/v/v) + 0.5% formic acid (HCOOH) each.

IR spectra were recorded on Specord M80 from Carl Zeiss (Jena, Germany) in transmittance mode in the 300–4000 cm^{-1} range, in ambient air at room temperature, with 2 cm^{-1} resolution and accumulation of 32 scans; wavenumber (frequency, ν) is given in cm^{-1}; samples were prepared as Nujol suspensions.

2. Synthetic procedures and analytical data for the obtained compounds.

2.1. General procedure for the synthesis of 6-iodopurine (2)

6-Chloropurine (**1**, 2.85 g, 18.44 mmol) was slowly added with stirring into an ice-cold 57% aqueous solution of HI (25 mL, ca. 10 equiv). The reaction mixture was allowed to stand at 0–5 °C for 2 h, and then filtered through a sintered glass funnel. The remaining precipitate was suspended in a cold H_2O (20 mL) and adjusted to pH 7.5 by adding 25% $NH_{3(aq)}$. After thorough chilling, the precipitate was filtered, washed with cold H_2O, and dried in a vacuum desiccator over anhydrous P_2O_5. Next, the remaining crude solid was purified by adding H_2O (35 mL) and 25% $NH_{3(aq)}$ (2.5 mL) and precipitation at pH 5 with AcOH (2 mL). After drying the solid at 110 °C, the desired 6-iodopurine (**2**, 3.0 g, 12.2 mmol, 66% yield) was obtained as a light beige solid.

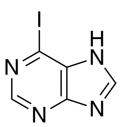

Mp 160 °C (25% $NH_{3(aq)}$/AcOH, decomp.) {lit.[1] 167 °C (decomp.) (25% $NH_{3(aq)}$/AcOH)}; R_f [$CHCl_3$/MeOH (90:10, v/v)] 0.47; ^{1}H NMR (500 MHz, DMSO-*d*$_6$): δ 8.74 (s, 1H), 8.68 (s, 1H), 8.63 (s, 0.1H), 8.58 (s, 0.1H); ^{13}C{^{1}H} NMR (126 MHz, DMSO-*d*$_6$): δ 151.8, 151.5, 144.7, 137.6, 122.0; IR (nujol): ν_{max} = 2924, 2340, 1592, 1556, 1464, 1320, 1224, 916, 836, 640 cm^{-1}; FTMS (ESI-TOF) *m/z*: [M+H]$^+$ Calcd for $C_5H_4IN_4^+$ m/z: 246.9475, Found 246.9475; GC [260 (const.)]: Not Found.

2.2. General procedure for the synthesis of 1-(6-chloro-9H-purin-9-yl)propan-2-one (3a)

To a solution of 6-chloropurine (**1**, 2.0 g, 12.9 mmol) in dry DMF (60 mL), anhydrous K_2CO_3 (1.79 g, 12.9 mmol) was added. Next, the mixture was stirred for 30 min at 25 °C, followed by the addition of a solution of chloroacetone (1.14 mL, 14.2 mmol) in dry DMF (20 mL) over 2.5 h. The reaction mixture was continuously stirred overnight at 25 °C, then filtered through Celite, and the filtrate was evaporated to dryness. The residue was dissolved in $CHCl_3$ (300 mL) and washed with saturated $NaHCO_3$ (4 × 50 mL) and brine (40 mL). The combined organic layer was dried over Na_2SO_4, and the solvent was evaporated. Afterward, the crude product was purified by column chromatography using CH_2Cl_2/MeOH (97:3, v/v) mixture as eluent, thus affording **3a** (1.58 g, 7.51 mmol, 58% yield) as a white solid.

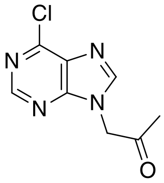

Mp 168–170 °C (CH_2Cl_2/MeOH) [lit.[2] 168–171 °C (no data)]; R_f [CH_2Cl_2/MeOH (97:3, v/v)] 0.27; ^{1}H NMR (500 MHz, $CDCl_3$): δ 8.71 (s, 1 H), 8.12 (s, 1H), 5.15 (s, 2H), 2.37 (s, 3H); ^{13}C{^{1}H} NMR (126 MHz, $CDCl_3$): δ 198.7, 152.3, 151.9, 151.4, 145.7, 110.1, 52.4, 27.4; IR (nujol): ν_{max} =1720,

1560, 1172, 948 cm^{-1}; FTMS (ESI-TOF) *m/z*: $[M+H]^+$ Calcd for $C_8H_8ClN_4O^+$ m/z: 211.0381, Found 211.0380; GC [260 (const.)]: t_R = 2.67 min.

2.3. General procedure for the synthesis of 1-(6-iodo-9H-purin-9-yl)propan-2-one (3b)

To a solution of 6-iodopurine (**2**, 2 g, 8.1 mmol) in dry DMF (60 mL), anhydrous K_2CO_3 (1.12 g, 8.1 mmol) was added. Next, the mixture was stirred for 30 min at 25 °C, followed by the addition of a solution of chloroacetone (720 μL, 8.9 mmol) in dry DMF (20 mL) over 2.5 h. The reaction mixture was continuously stirred overnight at 25 °C, then filtered through Celite, and the filtrate was evaporated to dryness. The residue was dissolved in $CHCl_3$ (300 mL) and washed with saturated $NaHCO_3$ (4 × 50 mL) and brine (40 mL). The combined organic layer was dried over Na_2SO_4, and the solvent was evaporated. The crude product was purified by recrystallization from 2-PrOH (20 mL), affording **3b** (1.21 g, 4.0 mmol, 49% yield) as a white solid.

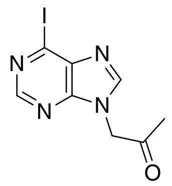

Mp 160–161 °C (2-PrOH) [lit.[3] 167–169 °C (no data)]; R_f [$CHCl_3$/MeOH (90:10, v/v)] 0.69; ^{1}H NMR (500 MHz, $CDCl_3$): δ 8.59 (s, 1H), 8.12 (s, 1H), 5.11 (s, 2H), 2.36 (s, 3H); $^{13}C\{^1H\}$ NMR (126 MHz, $CDCl_3$): δ 198.7, 152.2, 148.2, 145.1, 138.3, 122.3, 52.4, 27.4; IR (nujol): ν_{max} = 2866, 2332, 1728, 1552, 1464, 1332, 1208, 1168, 920 cm^{-1}; FTMS (ESI-TOF) *m/z*: $[M+H]^+$ Calcd for $C_8H_8IN_4O^+$ m/z: 302.9737, Found 302.9735; GC [260 (const.)]: 5.18 min.

2.4. General procedure for the synthesis of 1-(6-chloro-9H-purin-9-yl)propan-2-ol (rac-4a)

To a solution of 1-(6-chloro-9*H*-purin-9-yl)propan-2-one (**3a**, 1.58 g, 7.50 mmol) in MeOH/CH_3CN (80 mL, 2:1 v/v) $NaBH_4$ (341 mg, 9.0 mmol) was added portion-wise over 20 min. at 0–5 °C. The reaction mixture was stirred for an additional 20 min at 0–5 °C until TLC confirmed the complete consumption of the substrate. An excess of $NaBH_4$ reagent was decomposed by adding a saturated aqueous solution of NH_4Cl (40 mL), and the reaction volume was concentrated to 50 mL under vacuum. Next, the reaction mixture was extracted with $CHCl_3$ (4 × 100 mL). The combined organic layers were washed with a saturated aqueous solution of NH_4Cl (60 mL) and brine (60 mL), dried over Na_2SO_4, and concentrated under reduced pressure. The crude product was purified by column chromatography using a gradient of CH_2Cl_2/MeOH (from 97:3 to 95:5, v/v) mixture as eluent to afford the desired *rac*-**4a** (1.20 g, 5.64 mmol, 75% yield) as a white solid.

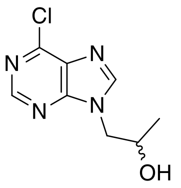

Mp 105–107 °C (CH_2Cl_2/MeOH) [lit.[4] 96–97 °C (no data)]; R_f [CH_2Cl_2/MeOH (95:5, v/v)] 0.27; ^{1}H NMR (500 MHz, $CDCl_3$): δ 8.67 (s, 1H), 8.18 (s, 1H), 4.42 (dd, J=14.2, 2.7 Hz, 1H), 4.27–4.35 (m, 1H), 4.11 (dd, J=14.1, 8.2 Hz, 1H), 3.65 (br. s., 1H), 1.31 (d, J=6.4 Hz, 3H); ^{13}C{^{1}H} NMR (126 MHz, $CDCl_3$): δ 152.0, 151.9, 150.8, 146.6, 131.2, 66.1, 51.7, 21.0; IR (nujol): ν_{max} = 3256, 2924, 2356, 1596, 1460, 1336, 1076, 948 cm^{-1}; FTMS (ESI-TOF) *m/z*: [M+H]$^+$ Calcd for $C_8H_{10}ClN_4O^+$ m/z: 213.0538, Found 213.0538; GC [260 (const.)]: t_R = 2.81 min; HPLC [*n*-hexane-2-PrOH (90:10, v/v); f=1.0 mL/min; λ=264 nm; *T*=30 °C (Chiralcel OJ-H)]: t_R = 16.210 (*S*-isomer) and 17.868 min (*R*-isomer). For (*S*)-(+)-**4a**: $[\alpha]_D^{30.0}$ = +26.59 (*c* 0.86, MeOH, >99% ee) [lit.[5] $[\alpha]_D^{20.0}$ = +39 (*c* 0.32, MeOH, 97% ee)]; for (*R*)-(–)-**4a**: $[\alpha]_D^{30.0}$ = –25.10 (*c* 1.17, MeOH, >99% ee) [lit.[5] $[\alpha]_D^{20.0}$ = –50 (*c* 0.14, MeOH, 97% ee)].

2.5. General procedure for the synthesis of 1-(6-iodo-9H-purin-9-yl)propan-2-ol (rac-4b)

To a solution of 1-(6-iodo-9*H*-purin-9-yl)propan-2-one (**3b**, 1.0 g, 3.3 mmol) in MeOH/CH_3CN (60 mL, 2:1 v/v) $NaBH_4$ (150 mg, 4.0 mmol) was added portion-wise over 20 min. at 0–5 °C. The reaction mixture was stirred for an additional 20 min at 0–5 °C until TLC confirmed the complete consumption of the substrate. An excess of $NaBH_4$ reagent was decomposed by the addition of a saturated aqueous solution of NH_4Cl (30 mL), and the reaction volume was concentrated to 40 mL under vacuum. The reaction mixture was extracted with $CHCl_3$ (4 × 80 mL). The combined organic layers were washed with a saturated aqueous solution of NH_4Cl (50 mL) and brine (50 mL), dried over Na_2SO_4, and concentrated under reduced pressure. The crude product was purified by column chromatography using $CHCl_3$/MeOH (95:5, v/v) mixture as eluent to afford the desired product *rac*-**4b** (915 mg, 3.0 mmol, 91% yield) as a white solid.

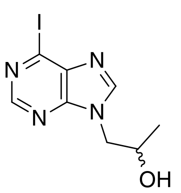

Mp 111–113 °C ($CHCl_3$/MeOH) [lit.[5] 112–114 °C (no data)]; R_f [$CHCl_3$/MeOH (95:5, v/v)] 0.27; ^{1}H NMR (500 MHz, $CDCl_3$): δ 8.57 (s, 1H), 8.21 (s, 1H), 4.39 (dd, J=14.1, 2.6 Hz, 1H), 4.27–4.35 (m, 1H), 4.11 (dd, J=14.2, 7.8 Hz, 1H), 2.94 (br. s., 1H), 1.30 (d, J=6.4 Hz, 3H); ^{13}C{^{1}H} NMR (126 MHz, $CDCl_3$): δ 152.0, 148.2, 145.9, 138.4, 122.0, 66.2, 51.6, 21.0; IR (nujol): ν_{max} = 2852, 1716, 1552, 1332, 1132, 1068, 924, 832 cm^{-1}; FTMS (ESI-TOF) *m/z*: [M+H]$^+$ Calcd for $C_8H_{10}IN_4O^+$ m/z: 304.9894, Found 304.9894; GC [260 (const.)]: t_R = 5.50 min; HPLC [*n*-hexane-2-PrOH (90:10, v/v); f=1.0 mL/min; λ=272 nm; *T*=30 °C (Chiralcel OJ-H)]: t_R = 26.792 (*S*-isomer) and 32.605 min (*R*-isomer). For (*S*)-(+)-**4b**: $[\alpha]_D^{28.0}$ = +18.64 (*c* 0.59, MeOH, 91% ee) [lit.[5] $[\alpha]_D^{20.0}$ = +26 (*c* 0.17, MeOH, 96% ee)].

2.6. *General procedure for the synthesis of 1-(6-chloro-9H-purin-9-yl)propan-2-yl acetate (rac-5a)*

To a solution of 1-(6-chloro-9*H*-purin-9-yl)propan-2-ol (*rac*-**4a**, 500 mg, 2.4 mmol) in CH_2Cl_2 (20 mL), Et_3N (357 mg, 3.5 mmol, 350 μL) and DMAP (10 mg) were added. The mixture was cooled to 0–5 °C in an ice bath. Next, acetyl chloride (277 mg, 3.5 mmol, 252 μL) was dissolved in dry CH_2Cl_2 (10 mL) and added dropwise to the reaction mixture using a syringe. Afterward, the resulting mixture was continuously stirred at cooling bath temperature and left to warm at room temperature (ca. 25 °C) for 48 h. The crude mixture was diluted with CH_2Cl_2 (25 mL), subsequently quenched with H_2O (50 mL), and the water phase was extracted with CH_2Cl_2 (3 × 25 mL). The combined organic layer was washed with a saturated aqueous solution of $NaHCO_3$ (100 mL), brine (100 mL), and dried over anhydrous $MgSO_4$. After filtration of the drying agent under suction and subsequent evaporation of the residuals of solvent under reduced pressure, the crude product was purified by column chromatography on silica gel using a gradient of CH_2Cl_2/MeOH (95:5 v/v) mixture as eluent, thus obtaining the desired acetate *rac*-**5a** (533 mg, 2.0 mmol, 89% yield) as a white solid.

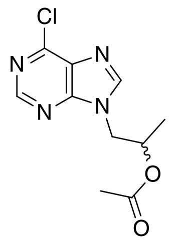

Mp 93–95 °C (CH_2Cl_2/MeOH); R_f [CH_2Cl_2/MeOH (95:5, v/v)] 0.45; ^{1}H NMR (500 MHz, $CDCl_3$): δ 8.74 (s, 1H), 8.13 (s, 1H), 5.27 (td, *J*=6.7, 3.3 Hz, 1H), 4.51 (dd, *J*=14.7, 3.4 Hz, 1H), 4.34 (dd, *J*=14.7, 7.1 Hz, 1H), 1.98 (s, 3H), 1.29 (d, *J*=6.4 Hz, 3H); ^{13}C{^{1}H} NMR (126 MHz, $CDCl_3$): δ 170.0, 152.4, 152.1, 151.3, 145.7, 131.0, 68.6, 48.2, 21.2, 7.6; IR (nujol): ν_{max} = 2908, 2340, 1740, 1592, 1564, 1228, 1068, 948, 636 cm^{-1}; FTMS (ESI-TOF) *m/z*: $[M+H]^+$ Calcd for $C_{10}H_{12}ClN_4O_2^+$ m/z: 255.0643, Found 255.0644; GC [260 (const.)]: t_R = 2.72 min; HPLC [*n*-hexane-2-PrOH (95:5, v/v); f=0.9 mL/min; λ=263 nm; *T*=30 °C (Chiralcel OD-H)]: t_R = 37.732 (*R*-isomer) and 42.878 min (*S*-isomer). For (*R*)-(–)-**5a**: $[\alpha]_D^{30.0}$ = –4.24 (*c* 0.59, MeOH, 99% ee) or $[\alpha]_D^{28.0}$ = –10.00 (*c* 0.95, $CHCl_3$, 99% ee).

2.7. *General procedure for the synthesis of 1-(6-iodo-9H-purin-9-yl)propan-2-yl acetate (rac-5b)*

To a solution of 1-(6-iodo-9*H*-purin-9-yl)propan-2-ol (*rac*-**4b**, 60 mg, 0.20 mmol) in CH_2Cl_2 (3 mL), Et_3N (30 mg, 0.30 mmol, 30 μL) and DMAP (10 mg) were added. The mixture was cooled to 0–5 °C in an ice bath. Next, acetic anhydride (30 mg, 0.30 mmol, 28 μL) was dissolved in dry CH_2Cl_2 (2 mL) and dropped portion-wise into the reaction mixture using a syringe. Afterward, the resulting mixture was continuously stirred at cooling bath temperature

and left to warm at room temperature (ca. 25 °C) for the next 24 h. The crude mixture was diluted with CH_2Cl_2 (5 mL) and subsequently quenched with H_2O (10 mL). The water phase was extracted with CH_2Cl_2 (3 × 5 mL), and the combined organic layer was washed with a saturated aqueous solution of $NaHCO_3$ (20 mL), brine (20 mL), and dried over anhydrous $MgSO_4$. After filtration of the drying agent under suction and subsequent evaporation of the residuals of solvent under reduced pressure, the crude product was purified by column chromatography on silica gel, using a gradient of $CHCl_3$/MeOH (95:5 v/v) mixture, thus obtaining the desired acetate *rac*-**5b** (54 mg, 0.16 mmol, 79% yield) as a white solid.

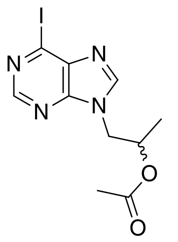

Mp 130–132 °C ($CHCl_3$/MeOH); R_f [$CHCl_3$/MeOH (95:5, v/v)] 0.69; ^{1}H NMR (500 MHz, $CDCl_3$): δ 8.62 (s, 1H), 8.12 (s, 1H), 5.26 (td, *J*=6.7, 3.3 Hz, 1H), 4.48 (dd, *J*=14.7, 3.4 Hz, 1H), 4.30 (dd, *J*=14.7, 7.1 Hz, 1H), 1.99 (s, 3H), 1.29 (d, *J*=6.6, 3H); ^{13}C{^{1}H} NMR (126 MHz, $CDCl_3$): δ 170.0, 152.3, 148.4, 144.9, 138.4, 122.4, 68.7, 48.1, 21.1, 17.5; IR (nujol): ν_{max} = 1736, 1556, 1336, 1232, 1084, 924, 836 cm^{-1}; FTMS (ESI-TOF) *m/z*: [M+H]$^+$ Calcd for $C_{10}H_{12}IN_4O_2^+$ m/z: 347.0000, Found 346.9997; GC [260 (const.)]: t_R = 5.20 min; HPLC [*n*-hexane-2-PrOH (90:10, v/v); f=0.8 mL/min; λ=272 nm; *T*=30 °C (Chiralpak AD-H)]: t_R = 20.671 (*R*-isomer) and 25.799 min (*S*-isomer). For (*R*)-(+)-**5b**: $[\alpha]_D^{28.0}$ = +5.00 (*c* 1.00, $CHCl_3$, 98% ee).

2.8. General procedure for the synthesis of trimethylsilyl 1-(6-chloro-9H-purin-9-yl)propan-2-ol (rac-6a)

To a solution of 1-(6-chloro-9*H*-purin-9-yl)propan-2-ol (*rac*-**4a**, 40 mg, 0.19 mmol) in CH_2Cl_2 (1 mL) *N*,*O*-bis(trimethylsilyl)acetamide (BSA, 153 mg, 0.75 mmol, 186 µL) was added in one portion, and the reaction mixture was stirred for 20 min at ambient temperature (ca. 25 °C). Next, the volatile compounds were evaporated under a vacuum, and the crude product was purified on SiO_2 column chromatography using a mixture of CH_2Cl_2/MeOH (95:5, v/v) as eluent. The resulting oil was additionally treated with MeOH (0.5 mL). The undissolved solid was filtered off. The filtrate was concentrated under a vacuum to afford the desired silylated alcohol *rac*-**6a** (44.5 mg, 0.16 mmol, 83% yield) as a white solid.

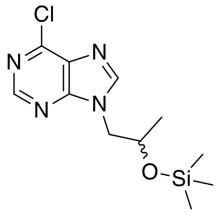

Mp 89–91 °C (MeOH) [lit.[5] 94–96 °C (no data)]; R_f [CH_2Cl_2/MeOH (95:5, v/v)] 0.73; ^{1}H NMR (500 MHz, $CDCl_3$): δ 8.73 (s, 1H), 8.17 (s, 1H), 4.37 (dd, *J*=13.8, 2.8 Hz, 1H), 4.11–4.20 (m, 1H), 4.01–4.10 (m, 1H), 1.23 (dd, *J*=6.1, 1.0 Hz, 3H), –0.12 (s, 9H); ^{13}C{^{1}H} NMR (126 MHz, $CDCl_3$): δ

152.0, 151.9, 151.0, 146.6, 131.5, 66.6, 51.5, 21.3, –0.2; IR (nujol): ν_{max} = 2940, 1668, 1560, 1336, 1252, 1144, 1076, 940, 840, 756 cm^{-1}; FTMS (ESI-TOF) *m/z*: $[M+H]^+$ Calcd for $C_{11}H_{18}ClN_4OSi^+$ m/z: 285.0933, Found 285.0933; GC [260 (const.)]: t_R = 2.05 min.

2.9. General procedure for the synthesis of trimethylsilyl 1-(6-iodo-9H-purin-9-yl)propan-2-ol (rac-6b)

To a solution of 1-(6-iodo-9*H*-purin-9-yl)propan-2-ol (*rac*-**4b**, 40 mg, 0.13 mmol) in CH_2Cl_2 (1 mL) *N,O*-bis(trimethylsilyl)acetamide (BSA, 107 mg, 0.53 mmol, 130 μL) was added in one portion, and the reaction mixture was stirred for 20 min at ambient temperature (ca. 25 °C). Next, the volatile compounds were evaporated under a vacuum, and the crude product was purified on SiO_2 column chromatography using a mixture of $CHCl_3$/MeOH (95:5, v/v) as eluent. The resulting oil was additionally treated with MeOH (0.5 mL). The undissolved solid was filtered off. The filtrate was concentrated under a vacuum to afford the desired silylated alcohol *rac*-**6b** (43 mg, 0.11 mmol, 87% yield) as a white solid.

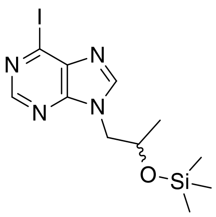

Mp 84–86 °C (MeOH); R_f [$CHCl_3$/MeOH (95:5, v/v)] 0.75; ^{1}H NMR (500 MHz, $CDCl_3$): δ 8.60 (s, 1H), 8.18 (s, 1H), 4.33 (dd, *J*=13.9, 2.9 Hz, 1H), 4.10–4.18 (m, 1H), 4.02 (dd, *J*=13.8, 8.2 Hz, 1H), 1.22 (d, *J*=6.4 Hz, 3H), –0.13 (m, 9H); $^{13}C\{^1H\}$ NMR (126 MHz, $CDCl_3$): δ 152.0, 148.2, 146.0, 138.5, 122.0, 66.6, 51.5, 21.3, –0.2; IR (nujol): ν_{max} = 2924, 1552, 1332, 1100, 1004, 840 cm^{-1}; FTMS (ESI-TOF) *m/z*: $[M+H]^+$ Calcd for $C_{11}H_{18}IN_4OSi^+$ m/z: 377.0289, Found 377.0289; GC [260 (const.)]: t_R = 3.43 min.

2.10. General procedure for the EKR of rac-4a–b – Enzyme screening

To a solution of *rac*-**4a–b** (0.12 mmol) in MTBE (2 mL), vinyl acetate (934 mg, 10.8 mmol, 1 mL) and the respective lipase preparation (5 mg) were added. The reaction mixture was stirred in a thermo-stated screw-capped glass vial (*V* = 4 mL) placed in an anodized aluminum reaction block at 40 °C and 800 rpm for the time indicated in **Table 1**. Next, the reaction was stopped by cooling the mixture, filtering off the enzyme on a Schott funnel under a vacuum, and washing the enzyme with a portion of MTBE (2 mL). After evaporation of the volatiles from the permeate, a small portion of the resulting crude oil was withdrawn to determine % conv. by using GC analysis after derivatization of the crude mixture with BSA (see protocols **2.8.** and **2.9.** above). The rest of the resulting crude oil was purified by silica gel column chromatography using a mixture of CH_2Cl_2/MeOH (95:5, v/v) as an eluent to afford (*S*)-(+)-**4a** and (*R*)-(–)-**5a**

or $CHCl_3$/MeOH (95:5, v/v) as an eluent to afford (*S*)-(+)-**4b** and (*R*)-(+)-**5b**. In order to obtain data concerning %-conversions, % ee as well as *E*-values, the HPLC analyses were performed for both EKR products. For HPLC analysis, the representative samples (2–3 mg) were diluted with HPLC-grade 2-PrOH (1.5 mL). For additional data, see **Table 1** in the main manuscript.

2.11. General procedure for (Amano PS-IM)-catalyzed KR of rac-4a – Solvent screening

The reaction mixture containing *rac*-**4a** (25 mg, 0.12 mmol), the appropriate organic solvent (2 mL), vinyl acetate (934 mg, 10.8 mmol, 1 mL), and Amano PS-IM (5 mg) was stirred using a magnetic stirrer (800 rpm IKA RCT basic) in a thermo-stated sealed glass vial (*V* = 4 mL) at 40 °C for 24 h. Further manipulations were carried out by analogy with the previous procedures for enzyme screening. For additional data, see **Table 2** in the main manuscript.

2.12. General procedure for (Amano PS-IM)-catalyzed KR of rac-4a – Kinetic

The reaction mixture composed of *rac*-**4a** (25 mg, 0.12 mmol), $PhCH_3$ (2 mL), vinyl acetate (934 mg, 10.8 mmol, 1 mL), and Amano PS-IM (5 mg) was stirred in a thermo-stated sealed glass vial (*V* = 4 mL) placed in anodized aluminum reaction block at 40 °C and 800 rpm for the subsequent time-scale: 4 h, 8 h, 16 h, 20 h, 24 h, and 30 h. Each of the enzymatic reactions was stopped by filtering off the enzyme on the Schott funnel under a vacuum, washing it with a portion of $PhCH_3$ (2 mL). After evaporation of the volatiles from the filtrate, the crude oil was purified by column chromatography packed with a silica gel using CH_2Cl_2/MeOH (95:5 v/v) mixture as eluent, thus yielding the desired optically active products (*S*)-(+)-**4a** and (*R*)-(–)-**5a**. For additional data, see **Table S3** appended into Supporting Information.

2.13. General procedure for (Amano PS-IM)-catalyzed KR of rac-4a – Effect of temperature

The reaction mixture containing *rac*-**4a** (25 mg, 0.12 mmol), $PhCH_3$ (2 mL), vinyl acetate (934 mg, 10.8 mmol, 1 mL), and Amano PS-IM (5 mg) was stirred (800 rpm, IKA RCT basic) in a thermo-stated sealed glass vial (*V* = 4 mL) for 8 h and/or 24 h at 40 °C, 50 °C, and 60 °C, respectively. After this time, the reaction was stopped by filtering off the enzyme on a Schott funnel under a vacuum and washing it with a portion of $PhCH_3$ (2 mL). The crude oil was purified by column chromatography packed with a silica gel using CH_2Cl_2/MeOH (95:5 v/v) mixture as eluent, thus yielding the desired optically active products (*S*)-(+)-**4a** and (*R*)-(–)-**5a**. For additional data, see **Table S4** appended into Supporting Information.

2.14. General procedure for preparative scale (Amano PS-IM)-catalyzed KR of rac-4a

2.14.1. Method A (ended with purification via column chromatography): The reaction mixture containing racemic 1-(6-chloro-9*H*-purin-9-yl)propan-2-ol (*rac*-**4a**, 500 mg, 2.4 mmol, 60 mM final conc.), $PhCH_3$ (40 mL), vinyl acetate (18.62 g, 216 mmol, 20 mL), and Amano PS-IM (100 mg) was stirred (800 rpm, IKA RCT basic) in an Ace round-bottom pressure flask with Ace-Thred 15 PTFE front-seal plug (capacity 250 mL; Sigma Aldrich No.: Z567191) for 26 h at 40 °C. Next, the enzymatic reaction was stopped by filtering off the lipase on a Schott funnel under a vacuum and by washing the biocatalyst preparation with a portion of $PhCH_3$ (20 mL). After evaporation of the volatiles, the crude oil was purified by silica gel column chromatography using a mixture of CH_2Cl_2/MeOH (95:5, v/v) to provide the corresponding EKR products as follows: (*S*)-(+)-**4a** (269 mg, 1.3 mmol, 54% yield, 79% ee, >99% purity) and (*R*)-(–)-**5a** (282 mg, 1.1 mmol, 47% yield, 99% ee, >99% purity) as a white solids. For details, see **Table 3** in the main manuscript.

2.14.2. Method B (ended with purification via liquid-liquid extraction): All the manipulations, except the workup and purification procedures, were carried out by analogy with the above protocol. To isolate EKR products, the crude reaction mixture was filtered off from the lipase preparation, and the filtrate cake was rinsed with a portion of $PhCH_3$ (20 mL). After evaporation of the volatiles, the crude oil was dissolved in $PhCH_3$ (40 mL) and washed with H_2O (3 × 20 mL). The combined organic layer was dried over $MgSO_4$, the drying agent was filtered off, and the permeate was concentrated under reduced pressure to provide (*R*)-(–)-**5a** (187 mg, 0.7 mmol, 31% yield, 99% ee, >99% purity) as a white solid. The combined aqueous phase was back-extracted with $PhCH_3$ (3 × 40 mL) to remove traces of (*R*)-(–)-**5a**. Afterward, an aqueous layer was azeotropically condensed with $PhCH_3$ (100 mL). The resulting oil residue was diluted with AcOEt (40 mL), dried over $MgSO_4$, and after filtering off the drying agent and evaporation of the volatile solvents, the desired (*S*)-(+)-**4a** (240 mg, 1.1 mmol, 48% yield, 82% ee, 92% purity) was obtained as a white solid. For details, see **Table 3** in the main manuscript.

2.15. General procedure for the stereoselective bioreduction of 3a–b – biocatalysts screening

Each of the *E. coli* cells containing the appropriate overexpressed recombinant ADH (10 mg) was suspended in 50 mM Tris–HCl buffer (400 μL; pH 7.5) with an additional portion of NAD(P)H [50 μL was taken from the 10 mM stock solution prepared in Tris–HCl buffer (1 mL) to obtain 1 mM final conc. of cofactor], and preincubated for 30 min at 30 °C. Then, the respective ketone **3a**–**b** [50 μL was taken from the 100 mM stock solution prepared in 2-PrOH

(1 mL) supplemented with DMSO (2.5% v/v) to obtain 10 mM final conc. of the substrate] was added, and the reaction mixture was shaken at 30 °C and 250 rpm for 24 h. After incubation, the enzymatic reaction was stopped by filtering off the cells using a syringe equipped with a hydrophobic PTFE filter (0.45 μM). Next, a portion of $PhCH_3$ (5 mL) was added to the permeate, and the water was azeotropically evaporated using a rotavap. The crude oil residue was diluted with HPLC-grade 2-PrOH and additionally passed through short-pad column chromatography (*vide* Pasteur pipette terminated with cotton wool and filled with SiO_2 gel) using HPLC-grade 2-PrOH (100%) as eluent, thus obtaining the desired optically active bioreduction products, i.e., (*S*)-(+)-**4a**–**b** or (*R*)-(–)-**4a**–**b**, depending on the employed biocatalyst.

2.16. General procedure for preparative-scale bioreduction of 3a using E. coli/ADH-A or E. coli/Lk-ADH Prince

E. coli/Lk-ADH-A or *E. coli*/Lk-ADH Prince (60 mg) was suspended in 50 mM Tris–HCl buffer (3.5 mL; pH 7.5) containing NADH (2.86 mg, 1.0 mM final concentration in the case of *E. coli*/ADH-A) or NADPH (3.33 mg, 1.0 mM final concentration in the case of *E. coli*/Lk-ADH Prince) and preincubated for 30 min at 30 °C with gentle shaking. Then, 1-(6-chloro-9*H*-purin-9-yl)propan-2-one (**3a**, 84 mg, 0.4 mmol, 100 mM final conc.) and 2-PrOH (400 μL, 10% v/v) supplemented with DMSO (100 μL, 2.5% v/v) were added to the mixture. The reaction was shaken at 30 °C and 250 rpm for 24 h. After incubation, the enzymatic reaction was stopped by filtering off the cells under a vacuum and rinsing the filtrate cake with $PhCH_3$ (15 mL). Next, the water was azeotropically evaporated from the permeate, and the crude oil residue was purified by silica gel column chromatography using a mixture of CH_2Cl_2/MeOH (95:5, v/v), thus obtaining the desired optically active product (*S*)-(+)-**4a** (60 mg, 0.28 mmol, 70% yield, >99% ee, in the case of *E. coli*/ADH-A) or (*R*)-(–)-**4a** (73 mg, 0.34 mmol, 86% yield, >99% ee, in the case of *E. coli*/Lk-ADH Prince) as a white solid.

2.17. General procedure for the synthesis of (2R)-1-(6-amino-9H-purin-9-yl)propan-2-ol [(R)-(–)-7]

2.17.1. Method A [direct amination of alcohol (R)-(–)-4a]: A solution of (2*R*)-1-(6-chloro-9*H*-purin-9-yl)propan-2-ol [(*R*)-(–)-**4a**, 100 mg, 0.47 mmol, >99% ee] in NH_3-saturated MeOH (10 mL was stirred for 48 h at 150 °C in a pressure tube. Next, the volatile compounds were evaporated under a vacuum, and the crude product was purified by column chromatography on

silica gel using a mixture of CH_2Cl_2/MeOH (95:5 v/v) as an eluent to afford (*R*)-(–)-**7** (81.5 mg, 0.42 mmol, 90% yield, 99% ee) as a white solid.

2.17.2. Method B [one-pot two-step amination-hydrolysis of acetate (R)-(–)-5a]: A solution of (2*R*)-1-(6-chloro-9*H*-purin-9-yl)propan-2-yl acetate [(*R*)-(–)-**5a**, 100 mg, 0.40 mmol, >99% ee] in NH_3-saturated MeOH (10 mL) was stirred for 48 h at 150 °C in a pressure tube. Next, the volatile compounds were evaporated under a vacuum, and the crude product was purified by column chromatography on silica gel using a mixture of CH_2Cl_2/MeOH (95:5 v/v) as an eluent to afford (*R*)-(–)-**7** (61.15 mg, 0.32 mmol, 81% yield, 99% ee) as a white solid.

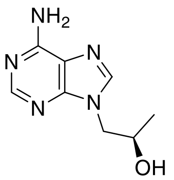

Mp 186–187 °C (decomp.) (CH_2Cl_2/MeOH) [lit.[6] 187–189 °C (no data)]; R_f [CH_2Cl_2/MeOH (90:10, v/v)] 0.18; ^{1}H NMR (500 MHz, DMSO-d_6): δ 8.13 (s, 1H), 8.04 (s, 1H), 7.19 (br s, 2H), 5.03 (d, *J*=4.7 Hz, 1H), 3.90–4.16 (m, 3H), 1.05 (d, *J*=6.2 Hz, 3H); ^{13}C{^{1}H} NMR (126 MHz, DMSO-d_6): δ 155.9, 152.3, 149.7, 141.5, 118.5, 64.6, 50.1, 20.9; IR (nujol): ν_{max} = 2924, 1676, 1604, 1456, 1072, 724 cm^{-1}; FTMS (ESI-TOF) *m/z*: [M+H]$^+$ Calcd for $C_8H_{12}N_5O^+$ m/z: 194.1036, Found 194.1035; GC [260 (const.)]: t_R = 2.956 min; HPLC [*n*-hexane-2-PrOH-DEA (95:5:0.1, v/v); f=0.8 mL/min; λ=261 nm; *T*=30 °C (Chiralpak OJ-H)]: t_R = 44.070 (*R*-isomer) and 48.060 min (*S*-isomer). For (*R*)-(–)-**7**: $[\alpha]_D^{30.0}$ = –39.42 (*c* 1.04, MeOH, 99% ee) [lit.[5] $[\alpha]_D^{20.0}$ = –39 (*c* 0.32, MeOH, 93% ee)].

General procedure for the synthesis of (diethoxyphosphoryl)methyl 4-methylbenzenesulfonate (9)

To a solution of diethyl (hydroxymethyl)phosphonate (**8**, 529 mg, 400 µL, 3.15 mmol) in dry CH_2Cl_2 (2 mL), Et_3N (382 mg, 3.78 mmol, 375 µL) was added. The mixture was cooled to 0–5 °C in an ice bath. Next, tosyl chloride (719 mg, 3.78 mmol) was dissolved in dry CH_2Cl_2 (3 mL) and added dropwise to the reaction mixture using a syringe. Afterward, the resulting mixture was continuously stirred at cooling bath temperature and left to warm at room temperature for the next 24 h. The crude mixture was diluted with CH_2Cl_2 (5 mL) and washed with H_2O (3 × 10 mL) and brine (3 × 10 mL). The combined organic layer was dried over anhydrous $MgSO_4$, and after filtering the drying agent, the permeate was concentrated under reduced pressure. The crude product was purified by column chromatography using a mixture of *n*-hexane/AcOEt (1:2, v/v) as eluent to afford the desired **9** (476 mg, 1.48 mmol, 47% yield) as a colorless oil.

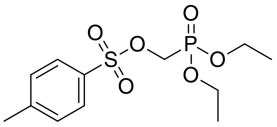

R_f [*n*-hexane/AcOEt (1:2, v/v)] 0.31; ^{1}H NMR (500 MHz, DMSO-d_6): δ 11.18 (t, J=7.0 Hz, 6H), 7.79–7.87 (m, 2H), 7.48–7.55 (m, 2H), 4.36 (d, J=9.5 Hz, 2H), 3.96–4.07 (m, 4H), 2.43 (s, 3H); ^{13}C{^{1}H} NMR (126 MHz, DMSO-d_6): δ 145.6, 131.3, 130.3, 128.0, 62.8 (d, $J_{C–P}$=6.9 Hz), 61.5 (d, $J_{C–P}$=164.9 Hz), 21.2, 16.1 (d, $J_{C–P}$=5.9 Hz); ^{31}P{^{1}H} (202 MHz, DMSO-d_6): δ = +15.8; IR (nujol): ν_{max} = 2984, 1596, 1368, 1260, 1180, 1028, 776, 664 cm^{-1}; FTMS (ESI-TOF) *m/z*: [M+H]$^+$ Calcd for $C_{12}H_{20}O_6PS^+$ m/z: 323.0713, Found 323.0711; GC [260 (const.)]: t_R = 4.18 min.

General procedure for the synthesis of diethyl ({[(2R)-1-(6-amino-9H-purin-9-yl)propan-2-yl]oxy}methyl)phosphonate [(R)-(–)-10]

To a solution of (2*R*)-1-(6-amino-9*H*-purin-9-yl)propan-2-ol [(*R*)-(–)-**7**, 50 mg, 0.26 mmol, 99% ee] in anhydrous DMF (2 mL) magnesium *tert*-butoxide (66 mg, 0.39 mmol) was added in one portion. The mixture was stirred under argon protective conditions at 65 °C for 1 h. Next, the temperature was raised to 75 °C, and a solution of diethyl methylsulfonyloxymethyl phosphonate (**9**, 125 mg, 0.39 mmol) in anhydrous DMF (0.5 mL) was added dropwise to the reaction mixture using a syringe. Afterward, the resulting mixture was continuously stirred at 75 °C for the next 24 h. Next, the volatile compounds were evaporated under a vacuum, and the crude product was purified by column chromatography on silica gel using a gradient of CH_2Cl_2/MeOH (98:2, 97:3, 95:5 v/v) mixture as an eluent to afford (*R*)-(–)-**10** (78.4 mg, 0.2 mmol, 88% yield, 99% ee) as a white solid.

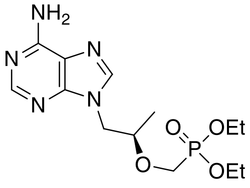

Mp 149–150 °C (CH_2Cl_2/MeOH) (no literature data); R_f [CH_2Cl_2/MeOH (90:10, v/v)] 0.33; ^{1}H NMR (500 MHz, DMSO-d_6): δ 8.13 (s, 1H), 8.05 (s, 1H), 7.19 (br s, 2H), 4.22–4.31 (m, 1H), 4.11–4.19 (m, 1H), 3.83–3.98 (m, 7H), 3.75 (dd, J=13.9, 9.5 Hz, 1H), 1.07–1.19 (m, 9H); ^{13}C{^{1}H} NMR (126 MHz, DMSO-d_6): δ 155.9, 152.4, 149.8, 141.4, 118.4, 75.5 (d, J=12.9 Hz), 61.8 (d, $J_{C–P}$=164.6 Hz), 60.7 (d, $J_{C–P}$=21.7 Hz), 46.8, 16.7, 16.2 (d, $J_{C–P}$=12.2 Hz); ^{31}P{^{1}H} (202 MHz, DMSO-d_6): δ = +21.2; IR (nujol): ν_{max} = 3120, 2368, 1676, 1604, 1248, 1144, 1028, 976, 724 cm^{-1}; FTMS (ESI-TOF) *m/z*: [M+H]$^+$ Calcd for $C_{13}H_{23}N_5O_4P^+$ m/z: 344.1482, Found 344.1481; GC [260 (const.)]: t_R = 15.57 min; HPLC [*n*-hexane-2-PrOH (85:15, v/v); f=0.8 mL/min; λ=260 nm; *T*=30 °C (Chiralpak AD-H)]: t_R = 26.194 (*S*-isomer) and 34.152 min (*R*-isomer). For (*R*)-(–)-**10**: $[\alpha]_D^{30.0}$ = –23.41 (*c* 1.02, MeOH, 99% ee) [lit.[5] $[\alpha]_D^{20.0}$ = –28.2 (*c* 0.86, MeOH, 97% ee)].

General procedure for the synthesis of ({[(2R)-1-(6-amino-9H-purin-9-yl)propan-2-yl]oxy}methyl)phosphonic acid [(R)-(–)-11, tenofovir]

To a solution diethyl ({[(2*R*)-1-(6-amino-9*H*-purin-9-yl)propan-2-yl]oxy}methyl)phosphonate [(*R*)-(–)-**10**, 100 mg, 0.3 mmol, 99% ee) in dry CH_2Cl_2 (6 mL) TMSBr (0.92 mL, 7.0 mmol) was added in one portion at 0–5 °C. The reaction mixture was stirred under argon for 48 h at 0–5 °C. Next, the solvent was evaporated, and the resulting yellowish oil was co-evaporated with MeOH (20 mL) followed by Et_2O (20 mL). The residue was dissolved in H_2O (20 mL) and extracted with CH_2Cl_2 (4 × 3 mL). The aqueous fraction was co-evaporated with $PhCH_3$ (100 mL), and the crude product was purified by preparative-layer chromatography (PLC) using a mixture of 2-PrOH/H_2O/25% NH_3 (70:20:10, v/v/v) to afford the desired product (*R*)-(–)-**11** (76.2 mg, 0.26 mmol, 91% yield, 99% ee) as a white solid.

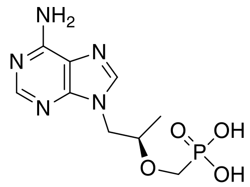

Mp 283–285 °C (decomp.) (2-PrOH/H_2O/25% NH_3) [lit.[7] 279 °C (H_2O/EtOH)]; R_f [2-PrOH/H_2O/25% NH_3 (70:20:10, v/v/v)] 0.16; ^1H NMR (500 MHz, D_2O): δ 8.41 (s, 1H), 8.40 (s, 1H), 4.50 (dd, *J*=14.7, 3.2 Hz, 1H), 4.32 (dd, *J*=14.9, 6.9 Hz, 1H), 3.97–4.07 (m, 1H), 3.71 (dd, *J*=13.2, 9.3 Hz, 1H), 3.51 (dd, *J*=13.2, 9.5 Hz, 1H), 1.20 (d, *J*=6.4 Hz, 3H); $^{13}C\{^1H\}$ NMR (126 MHz, DMSO-d_6): δ 155.8, 152.2, 149.8, 141.7, 118.2, 75.3 (d, *J*=11.7 Hz), 64.5 (d, *J*=161.2 Hz), 46.5, 17.0; $^{31}P\{^1H\}$ (202 MHz, DMSO-d_6): δ = +16.6; IR (nujol): ν_{max} = 3212, 1700, 1412, 1236, 1076, 936, 752 cm^{-1}; FTMS (ESI-TOF) *m/z*: $[M+H]^+$ Calcd for $C_9H_{15}N_5O_4P^+$ m/z: 288.0856, Found 288.0855. For (*R*)-(–)-**11**: $[\alpha]_D^{30.0}$ = –18.91 (*c* 1.00, 0.1 M HCl, 99% ee) [lit.[7] $[\alpha]_D^{(no\ data)}$ = –21.2 (*c* 0.5, aq HCl, not reported)].

3. Table S1. List of commercial enzyme preparations employed in these studies.

Enzyme and its origin (microorganism/tissue)	Enzyme preparation[a] (brand name)	Usage form of enzyme preparation	Enzyme specified activity	Commercial supplier (Cat. No.)
**Lipase from *Candida antarctica B* (CAL-B)**	**Novozym 435**	immobilized on the macroporous acrylic resin [poly (methyl methacrylate-co-butyl methacrylate)]	>10000 U/g or 10 PLU/mg, water content 1.4%	Novozymes A/S (Bagsvaerd, Denmark)
	**Lipozyme 435**	immobilized on Lewatit VP OC 1600	unspecified	Novozymes A/S (Bagsvaerd, Denmark)
	**Chirazyme L-2, c.-f., C2, Lyo.**	immobilized on the carrier-fixed (carrier 2)	150 kU	Roche
	**Chirazyme L-2, c.-f., C3, Lyo.**	immobilized on the carrier-fixed (carrier 3)	150 kU	Roche
	**Novozym 435-STREM**	immobilized on the macroporous acrylic resin	10000 PLU/g	STREM Chemicals, INC. (cat. nr.: 06-3123)
	**CAL-B-Immobead 150**	immobilized on Immobead 150	≥1800 U/g	Sigma Aldrich (cat. nr.: 54326)
**Lipase from *Candida antarctica A* (CAL-A)**	**Chirazyme L-5**	native	unspecified	Boehringer Mannheim[b]
**Lipase from *Burkholderia cepacia* (BCL)**	**PS-Immobead 150**	immobilized on Immobead 150	≥900 U/g	Sigma Aldrich (cat. nr.: 54327)
	**Amano PS**	native	>23.000 U/g	Amano Pharmaceutical Co., Ltd.
	**Amano PS-IM**	immobilized on diatomite	500 U/g	Amano Pharmaceutical Co., Ltd.
**Lipase from *Pseudomonas fluorescens* (PFL)**	**Amano AK**	native	>20.000 U/g	Amano Pharmaceutical Co., Ltd.
**Lipase from *Thermomyces lanuginosus* (TLL)**	**TL-Immobead 150**	immobilized on Immobead 150	≥3000 U/g	Sigma Aldrich (cat. nr.: 76546)
	**Lipozyme TL IM**	immobilized on a non-compressible silica gel carrier into an immobilized granulate (a silica granulated)	170 IUN/g	Novozymes A/S (Bagsvaerd, Denmark)
**Lipase from *Rhizomucor miehei* (RML)**	**Lipozyme RM IM**	immobilized (unspecified carrier)	150 IU/g	Novozymes A/S (Bagsvaerds, Denmark)
**Lipase from *Mucor javanicus***	**Amano Lipase M**	native	>10.000 U/g	Sigma Aldrich (cat. nr.: 534803)
**Lipase from *Rhizopus oryzae***	**Amano Lipase F-AP15**	native	≥150.000 U/g	Sigma Aldrich (cat. nr.: 534811)
**Lipase from *Candida rugosa***	**Lipase AY Amano 30**	native	>30.000 U/g	Amano Pharmaceutical Co., Ltd.
**Esterase from porcine liver**	**PLE**	native	≥50 U/mg	Sigma Aldrich (cat. nr.: 46058)

[a] All commercial formulations of enzymes studied herein were used without pre-treatment.

[b] Currently: Roche Diagnostics.

4. Table S2. The results of specific rotation values for the optically active products.

Compound	ee [%]	Measured specific rotation [α]$_D$	Literature specific rotation [α]$_D$	Ref.
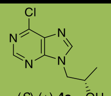 (*S*)-(+)-**4a**	>99	$[\alpha]_D^{30.0}$ = +26.59 (*c* 0.86, MeOH)	$[\alpha]_D^{20.0}$ = +39 (*c* 0.32, MeOH, 97% ee)	[5]
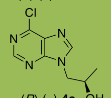 (*R*)-(–)-**4a**	>99	$[\alpha]_D^{30.0}$ = –25.10 (*c* 1.17, MeOH)	$[\alpha]_D^{20.0}$ = –50 (*c* 0.14, MeOH, 97% ee)	[5]
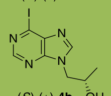 (*S*)-(+)-**4b**	91	$[\alpha]_D^{28.0}$ = +18.64 (*c* 0.59, MeOH)	$[\alpha]_D^{20.0}$ = +26 (*c* 0.17, MeOH, 96% ee)	[5]
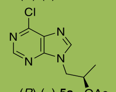 (*R*)-(–)-**5a**	99	$[\alpha]_D^{30.0}$ = –4.24 (*c* 0.59, MeOH);<br>$[\alpha]_D^{28.0}$ = –10.00 (*c* 0.95, $CHCl_3$);	–	[–]
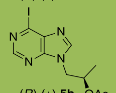 (*R*)-(+)-**5b**	98	$[\alpha]_D^{28.0}$ = +5.00 (*c* 1.00, $CHCl_3$)	–	[–]
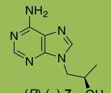 (*R*)-(–)-**7**	99	$[\alpha]_D^{30.0}$ = –39.42 (*c* 1.04, MeOH)	$[\alpha]_D^{20.0}$ = –39 (*c* 0.32, MeOH, 93% ee)	[5]
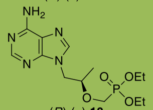 (*R*)-(–)-**10**	99	$[\alpha]_D^{30.0}$ = –23.41 (*c* 1.02, MeOH)	$[\alpha]_D^{20.0}$ = –28.2 (*c* 0.86, MeOH, 97% ee)	[5]
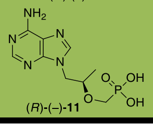 (*R*)-(–)-**11**	99	$[\alpha]_D^{30.0}$ = –18.91 (*c* 1.00, 0.1 M HCl)	$[\alpha]_D^{(no\ data)}$ = –21.2 (*c* 0.5, aq HCl, not reported)	[7]

5. Table S3. Studies on the reaction time-course of (Amano PS-IM)-catalyzed KR of *rac*-4a with vinyl acetate in $PhCH_3$ at 40 °C.

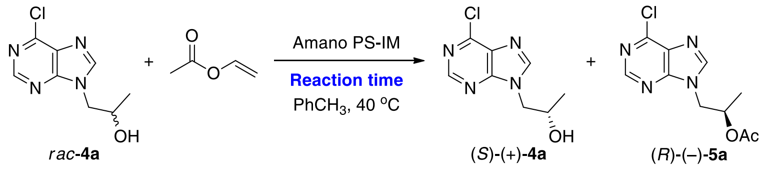

Entry	Time[a] [h]	Conv.[b] [%]	ee_s[c] [%]	ee_p[c] [%]	E[d]
1	4	12	13	>99	>200
2	8	23	30	>99	>200
3	16	40	65	>99	>200
4	20	42	71	>99	>200
5	24	46	85	>99	>200
6	30	47	88	99	>200

[a] Conditions: *rac*-**4a** (0.12 mmol), Amano PS-IM 5 mg (42 mg/mmol), $PhCH_3$ 2 mL, vinyl acetate 1 mL (92 equiv), 40 °C, 800 rpm (magnetic stirrer).

[b] Conversion values (%) (i.e., consumption of substrate *rac*-**4a**) were determined by GC analyses after derivatization of crude mixture with BSA as a silylating reagent; for confirmation, the % conversion was calculated from the enantiomeric excess of the unreacted alcohol (ee_s) and the formed acetate (ee_p) according to the formula conv. = $ee_s/(ee_s + ee_p)$.

[c] Determined by HPLC analyses using columns packed with chiral stationary phases.

[d] Calculated according to Chen *et al.*[8], using the equation: $E = \{\ln[(1 - \text{conv.})(1 - ee_s)]\}/\{\ln[(1 - \text{conv.})(1 + ee_s)]\}$.

6. Table S4. Temperature effect on (Amano PS-IM)-catalyzed KR of *rac*-4a with vinyl acetate in $PhCH_3$ after 8 h and 24 h.

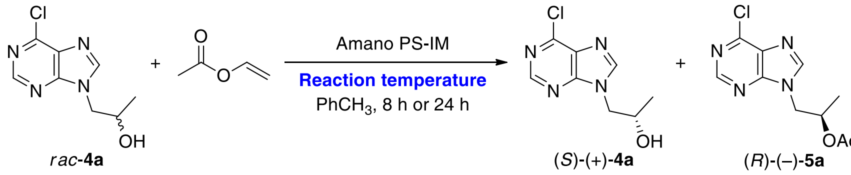

Entry	Temp.[a] [°C]	t [h]	Conv.[b] [%]	ee_s[c] [%]	ee_p[c] [%]	E[d]
1	40	8	23	30	>99	>200
2	40	24	46	85	>99	>200
3	50	8	29	41	>99	>200
4	50	24	49	95	98	>200
5	60	8	32	47	>99	>200
6	60	24	51	98	96	>200

[a] Conditions: *rac*-**4a** (0.12 mmol), Amano PS-IM 5 mg (42 mg/mmol), $PhCH_3$ 2 mL, vinyl acetate 1 mL (92 equiv), 800 rpm (magnetic stirrer).

[b] Conversion values (%) (i.e., consumption of substrate *rac*-**4a**) were determined by GC analyses after derivatization of crude mixture with BSA as a silylating reagent; for confirmation, the % conversion was calculated from the enantiomeric excess of the unreacted alcohol (ee_s) and the formed acetate (ee_p) according to the formula conv. = $ee_s/(ee_s + ee_p)$.

[c] Determined by HPLC analyses using columns packed with chiral stationary phases.

[d] Calculated according to Chen *et al.*[8], using the equation: $E = \{\ln[(1 - \text{conv.})(1 - ee_s)]\}/\{\ln[(1 - \text{conv.})(1 + ee_s)]\}$.

7. Molecular docking.

7.1. Molecular docking preparation

Molecular docking simulations to establish favorable ligand binding geometries for the studied ligand, namely 1-(6-chloro-9*H*-purin-9-yl)propan-2-one (**3a**), were carried out on a 24 CPUs-based desktop PC computer equipped with AMD Ryzen™ 9 3900X 12-Core Processor 3800 MHz and 32 GB of RAM on a Microsoft Windows 11 Professional 64-bit operating system using AutoDock Vina vs. 1.1.2 program for Windows (http://autodock.scripps.edu/)[9]. At first, the respective ligand molecule **3a** in non-ionizable form was prepared with ChemAxon MarvinSketch vs. 14.9.1.0 (http://www.chemaxon.com/marvin/) using the general 'Cleaning in 3D' option to assign with proper 3D orientation and then calculating conformers with MMFF94 force field parameters and saved as .pdb file. To obtain the minimum energy conformation for docking studies, the initial geometries of the afore-pretreated ligand were additionally optimized in Avogadro vs. 1.2.0. (http://avogadro.cc/) using MMFF94 force field with 500 steps and Steepest Descent Algorithm, after adding all the hydrogens to the structure, and saved as .mol2 files. The energy of the ligand molecules was minimized using the built-in feature of Avogadro, including the General Amber Force Field (GAFF)[10] with the Steepest Descent Algorithm (100 steps). The minimum conformation energy obtained for ligand **3a** was $E_{calc.}$ = 100.983 kJ/mol. The visualization of the optimized geometry of **3a** was performed using the molecular visualization software POV-Ray for Windows vs. 3.7.0.msvc10.win64 licensed under the terms of the GNU Affero General Public License (AGPL3) (**Figure S1**).

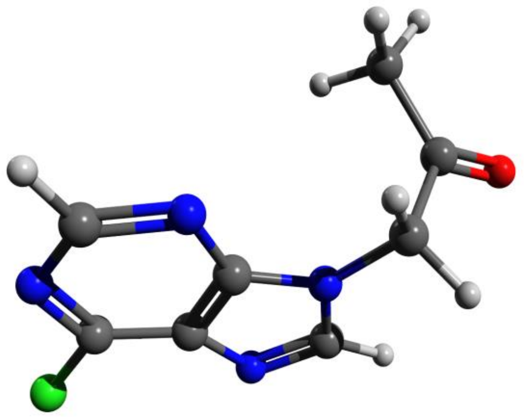

Figure S1. The geometry of 1-(6-chloro-9*H*-purin-9-yl)propan-2-one (**3a**) optimized in Avogadro – Version 1.2.0. The figure was prepared by rendering them using molecular visualization software POV-Ray – Version 3.7.0. Nitrogen atoms are presented with blue color, oxygen atoms with red color, chlorine atom with green color, whereas hydrogen atoms are expressed as light-grey balls.

The Gaussian Input file for geometry optimization of 1-(6-chloro-9*H*-purin-9-yl)propan-2-one (**3a**) was performed using the standard set of parameters (theory: B3LYP, basis: 6-31G(d), charge: 0, and multiplicity: 1). The list of the Cartesian coordinates for the optimized structure of the ligand molecule **3a** is as follows:

%NProcShared=2
#n B3LYP/6-31G(d) Opt

Cartesian coordinates for the optimized structure of 1-(6-chloro-9*H*-purin-9-yl)propan-2-one (**3a**):

0 1

N	-0.20750	-2.39510	0.64830
C	0.72310	-1.49120	0.42350
N	0.19810	-0.22850	0.37190
C	-1.14900	-0.32860	0.57450
N	-2.05120	0.66750	0.59720
C	-3.29560	0.19920	0.83590
N	-3.68220	-1.07560	1.02640
C	-2.71480	-2.01150	0.97910
C	-1.38630	-1.68430	0.74990
Cl	-3.13920	-3.63600	1.20750
C	0.93930	0.99120	0.15780
C	1.38240	1.12130	-1.27660
C	0.33660	1.48050	-2.29910
O	2.55100	0.90840	-1.60330
H	1.77950	-1.69470	0.28800
H	-4.08460	0.94560	0.87980
H	0.32970	1.85520	0.43850
H	1.81510	0.97330	0.81520
H	0.81930	1.68110	-3.25980
H	-0.19390	2.38200	-1.98310
H	-0.36440	0.65160	-2.42020

Afterward, the Gasteiger partial charges were calculated with AutoDock Tools vs. 1.5.6 (ADT, S3 http://mgltools.scripps.edu/). In contrast, all torsion angles for ligand **3a** were considered flexible, and all the possible rotatable bonds and non-polar hydrogens were determined (the number of rotatable bonds found in **3a** were 2 out of 32). The final ligand file was saved as PDBQT files (.pdbqt format) and was ready for the docking procedure disclosed in section *7.2. Molecular docking procedure*.

The crystal structures of the studied alcohol dehydrogenases (ADHs), namely alcohol dehydrogenase ADH-A from *Rhodococcus ruber* DSM 44541 (PDB code: 2XAA)[11] with the 2.80 Å resolution and alcohol dehydrogenase Lk-ADH from *Lactobacillus kefir* (PDB code: 4RF2)[12] with the 2.09 Å resolution, were downloaded from Brookhaven RCSB Protein Data Bank (PDB database, http://www.rcsb.org/pdb/). To avoid steric clashes within the model, the crude target proteins .pdb files were prepared by UCSF Chimera vs. 1.11.2 package (http://www.cgl.ucsf.edu/chimera/)[13] by removing all nonstandard molecules, including 1,4-butanediol (BU1) in the case of 2XAA as well as crystal waters (HOH) in case of 2XAA and 4RF2, respectively. The remaining ligands, including nicotinamide-adenine-dinucleotide (NAD^+, denoted as NAD) and zinc ion (ZN) in the case of 2XAA, as well as nicotinamide-adenine-dinucleotide phosphate (NADP+, denoted as NAP) and magnesium ion (MG), have been left to determine the relevant interactions between ligand-receptor. Next, the polar hydrogen atoms were added, and Gasteiger charges were calculated with AutoDock Tools 1.5.6 package using its standard utility scripts, and then the final protein files were saved as PDBQT files (.pdbqt). Next, a searching 'grid box' was set by using the AutoGrid function to perform docking in a (40 × 40 × 40 Å)-unit grid box (final size space dimension: x = 40 Å, y = 40 Å, z = 40 Å), centered on catalytic cavity located in ADH-A (PDB code: 2XAA) (center_x = 21.644; center_y = −24.639; center_z = 24.583) or Lk-ADH (PDB code: 4RF2) (center_x = 25.365; center_y = −31.938; center_z = 29.345) as target coordinate with a grid spacing of 0.325 Å, respectively.

7.2. Molecular docking procedure

Docking was performed into a rigid protein as well as using advanced protein flexibility by specifying flexible sidechains. Each docking was performed with an exhaustiveness level of 96 concerning global search. For each ligand molecule, 100 independent runs were performed using the Lamarckian Genetic Algorithm (GA) with at most 106 energy evaluations and a maximum number of generations of >27 000 Å^3 (the search space volume). The rest of the docking parameters, including the remaining Lamarckian GA parameters, were set as default using the standard values for genetic Vina algorithms (the posed dockings were below 5.00 Å rmsd). The docking modes of the studied ligand **3a** were clustered and ranked based on a mutual ligand–protein affinity expressed as absolute free binding energies [ΔG_{calc} (kcal/mol)] as well as the values of root mean square deviation (rmsd) in both modes regarding rmsd lower bound (l.b.), and rmsd upper bound (u.b.), respectively (**Table S5**). The rmsd-values were computed referring to the input structure submitted to docking simulations. For ADH-A (PDB code: 2XAA), the used random seed amounted to –271090392; whereas for Lk-ADH (PDB code: 4RF2), the used random seed amounted to –1983867596. The best nine poses (modes) for **3a**-ADH-A complex and five for **3a**-Lk-ADH complex were selected according to AutoDock Vina scoring functions mainly based on binding energies and showed mutual ligand–protein affinity (kcal/mol). The results generated by AutoDock Vina, including optimized binding poses of **3a** in hypothetical complexes with ADH-A (PDB code: 2XAA) and Lk-ADH (PDB code: 4RF2) proteins as well as critical polar contacts between the respective atoms of the ligand and receptor molecules (2XAA and 4RF2), were visualized using The PyMOL Molecular Graphics System software, vs. 1.3, Schrödinger, LLC (https://www.pymol.org/) (**Figure S2**). Two-dimensional (2D) visualization of the ADH-A (PDB code: 2XAA) and Lk-ADH (PDB code: 4RF2) binding interfaces for top-scoring poses of the ligand **3a** molecule was computed by BIOVIA Discovery Studio Visualizer 20.1.0.19295 software (**Figure S3**).

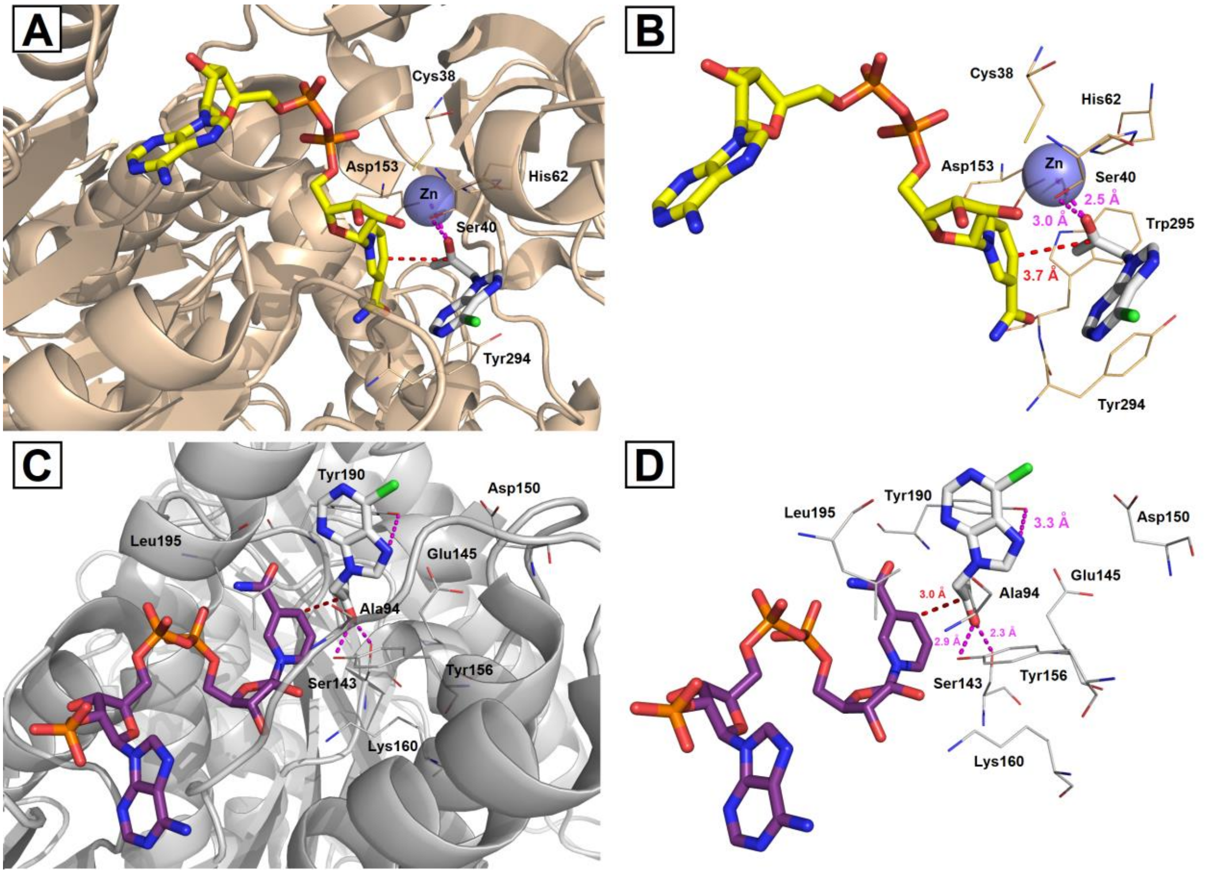

Figure S2. Representative three-dimensional (3D) binding modes of 1-(6-chloro-9*H*-purin-9-yl)propan-2-one (**3a**) with stereocomplemantary alcohol dehydrogenases, namely ADH-A (PDB code: 2XAA; panel **A**–**B**) and Lk-ADH (PDB code: 4RF2; panel **C**–**D**), with close contacts to amino acid residues and cofactors located in the active sites. The docked ligand **3a** and the cofactors are shown as *sticks* representation, where **3a** is white, NADH is yellow, and NADPH is violet. The overall receptor structures are shown as a semi-transparent *cartoon* diagram (left column; panels **A** and **C**), where ADH-A is wheat and Lk-ADH is grey, respectively. The most significant amino acid residues contributing to the stabilization of the ligand **3a** molecule in the complex with ADH-A or Lk-ADH by polar interactions, alkyl−alkyl (CH–CH) van der Waals (vdW) and/or π−alkyl (CH–π) interactions are shown in *lines* representations. Nitrogen atoms are presented with blue color, oxygen atoms with red color, chlorine atoms with green color, whereas phosphorus atoms with orange color. All the hydrogens were omitted for clarity. The zinc ion is presented as a semi-transparent slate *sphere*. The formation of intermolecular hydrogen bonds is represented by magenta dashed lines, whereas the plausible trajectory of the hydride transfer from cofactors to a carbon atom of the carbonyl group is shown as red dashed lines. Mutual distances between the amino acid residues and the respective ligand's atoms are given in Ångström (right column; panels **B** and **D**). The figure was prepared using the program PyMOL (http://www.pymol.org/).

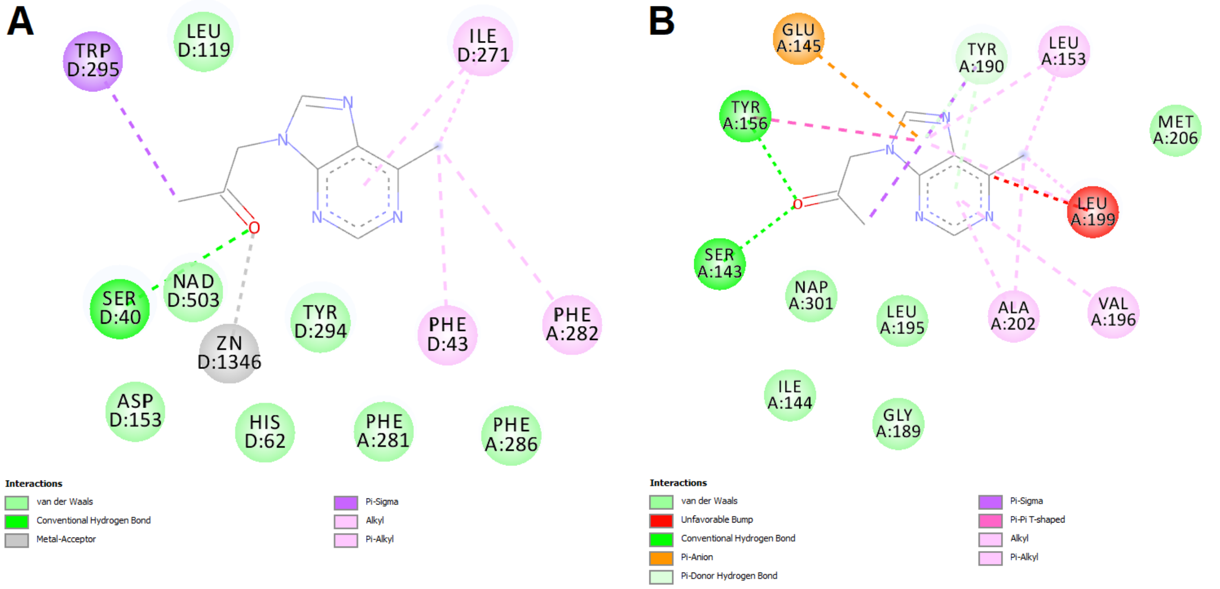

Figure S3. Complexes of ADH-A (PDB code: 2XAA) with NAD$^+$ cofactor (**A**) and Lk-ADH (PDB code: 4RF2) with NADP$^+$ cofactor (**B**) and the selected top-scoring poses of **3a** with interacting amino acid residues. The 2D protein-ligand interactions maps were generated by using freeware for academia BIOVIA Discovery Studio Visualizer 20.1.0.19295 software (Dassault Systèmes Biovia Corp.; https://www.3ds.com). Polar contacts between receptor-ligand including conventional hydrogen bonds (green), π−donor hydrogen bonds (light green), π−anion (orange), metal-acceptor (grey), unfavorable acceptor−acceptor bump clashes (red), as well as non-polar contacts between receptor-ligand including π–σ interactions (purple/violet), π–alkyl (light pink), and π–π T-shaped (pink) are represented by dashed lines. Intermolecular Van der Waals forces are displayed in light-green spoked arcs.

8. Table S5. Docking scoring of 1-(6-chloro-9*H*-purin-9-yl)propan-2-one (3a) complexed with alcohol dehydrogenases.

Entry	Ligand	Enzyme	Pose[a]	Affinity (kcal/mol)	Distance from best mode[b] rmsd l.b.	rmsd u.b.
1	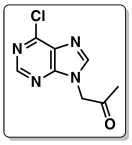	ADH-A from *Rhodococcus ruber* DSM 44541 (PDB code: 2XAA)	S1	–6.2	0.000	0.000
2			S2	–6.1	1.699	2.733
3			S3	–6.0	3.048	4.526
4			S4	–6.0	1.041	2.483
5			S5	–5.9	2.993	5.236
6			S6	–5.8	20.228	21.379
7			S7	–5.7	1.815	2.216
8			S8	–5.7	2.900	4.820
9			S9	–5.7	18.969	20.164
10		Lk-ADH from *Lactobacillus kefir* (PDB code: 4RF2)	S1	8.8	0.000	0.000
11			S2	11.2	3.210	4.612
12			S3	11.3	2.421	3.689
13			S4	11.3	3.368	4.884
14			S5	11.7	1.089	1.095

[a] The pose S1 represents the lowest value of ΔG_{calc} (kcal/mol), which means that ligand-binding affinity to a receptor is the highest, and on the contrary, the S9 mode (in the case of ADH-A) or S5 mode (in the case of Lk-ADH) represents the lowest ligand-binding affinity.
[b] The values <2.000 rmsd represent the closest distance between the ligand and the opioid receptor binding site.

9. Table S6. Analytical separation conditions of racemic compounds by GC column.

Compound	Temperature program	Retention time [min]
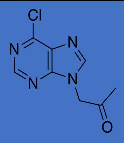	260 (isothermal)	2.67
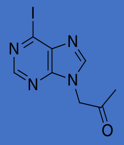		5.18
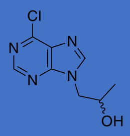	260 (isothermal)	2.81
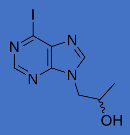		5.50
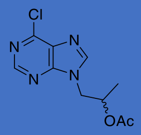	260 (isothermal)	2.72
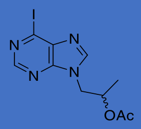		5.20
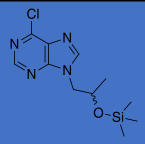	260 (isothermal)	2.05
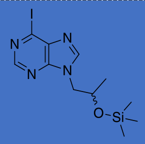		3.43
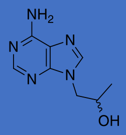	260 (isothermal)	2.96
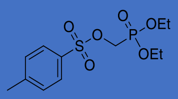	260 (isothermal)	4.18
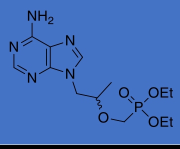	260 (isothermal)	15.57

10. Table S7. HPLC analytical separation conditions of purine derivatives by chiral columns – Chiralcel OD-H or Chiralcel OJ-H or Chiralpak AD-H.[a]

Compound	HPLC Column	Mobile Phase<br>*n*-Hexane/IPA/DEA [v/v/v]	Flow Rate [mL/min] (Pressure [MPa])	Detection [nm]	Retention Time [min]
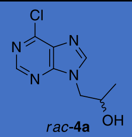 *rac*-**4a**	Chiralcel OJ-H	90:10:0	1.0 (4.5)	264	16.210 (*S*) and 17.868 (*R*)
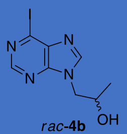 *rac*-**4b**	Chiralcel OJ-H	90:10:0	1.0	272	26.792 (*S*) and 32.605 (*R*)
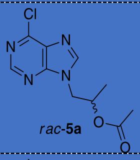 *rac*-**5a**	Chiralcel OD-H	95:5:0	0.9	263	37.732 (*R*) and 42.878 (*S*)
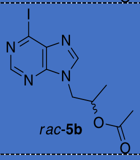 *rac*-**5b**	Chiralpak AD-H	90:10:0	0.8	272	20.671 (*R*) and 25.799 (*S*)
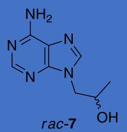 *rac*-**7**	Chiralcel OJ-H	95:5:0.1	0.8	261	44.070 (*R*) and 48.060 (*S*)
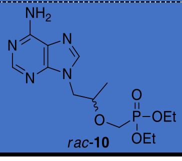 *rac*-**10**	Chiralpak AD-H	85:15:0	0.8	260	26.194 (*S*) and 34.152 (*R*)

[a] Performed on a Shimadzu Nexera-*i* (LC-2040C 3D) equipped with a photodiode array detector (PAD).

11. Analytical data (copies of HPLC chromatograms)

HPLC of *rac*-4a on Chiralcel OJ-H at 30 °C

Conditions: *n*-hexane-2-PrOH (90:10, v/v); f=1.0 mL/min; λ=264 nm; *p*=4.5 MPa

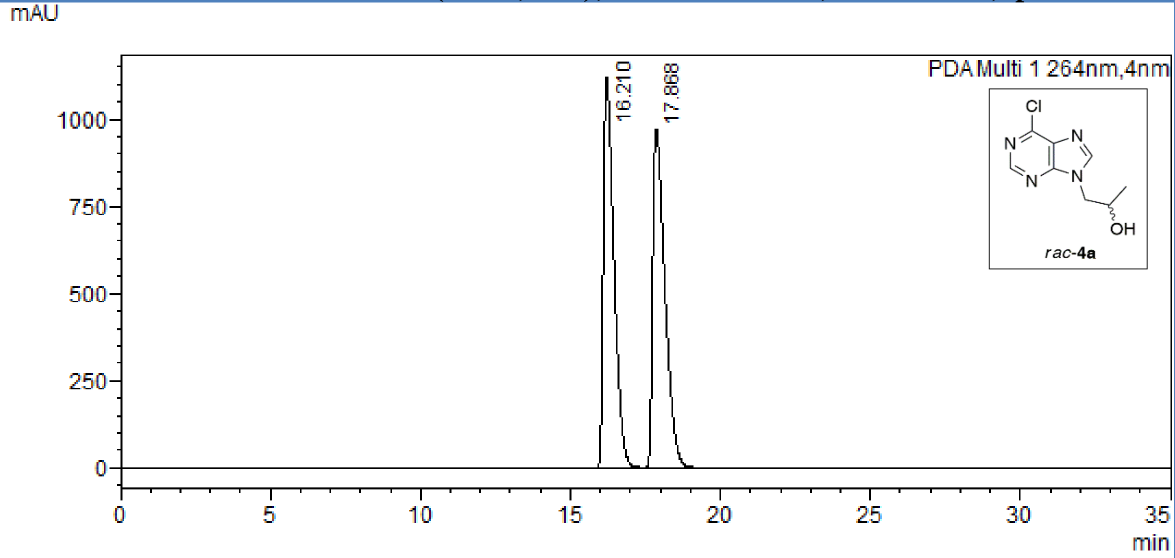

Peak Table

PDA Ch1 264nm

Peak#	Ret. Time	Area	Height	Height%	Area%
1	16.210	27661596	1119207	53.492	49.959
2	17.868	27706959	973091	46.508	50.041
Total		55368556	2092298	100.000	100.000

HPLC of (*S*)-(+)-4a on Chiralcel OJ-H at 30 °C

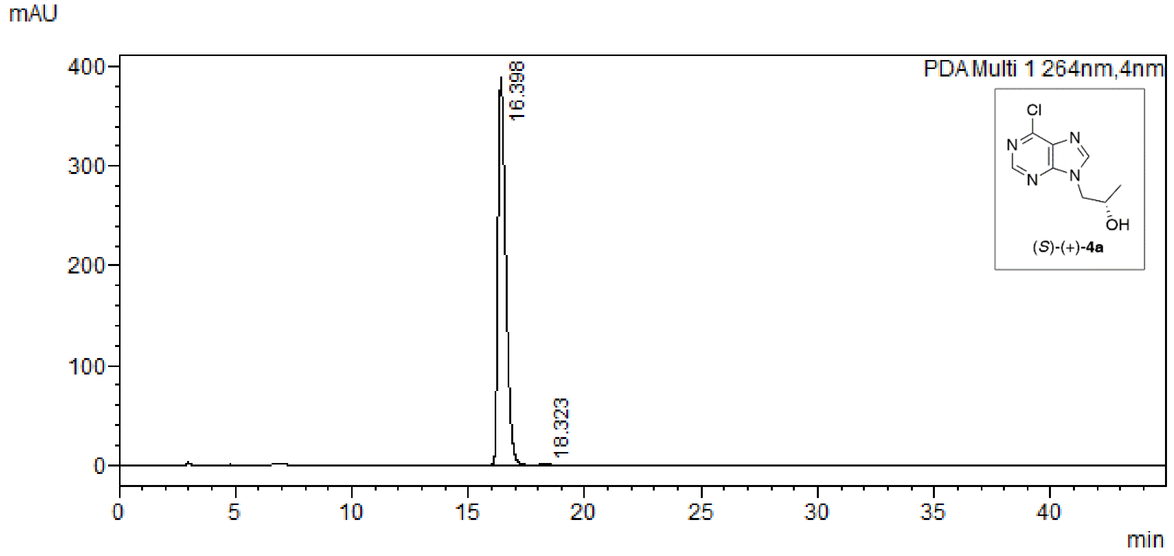

Peak Table

PDA Ch1 264nm

Peak#	Ret. Time	Area	Height	Height%	Area%
1	16.398	8948482	388616	99.751	99.766
2	18.323	21020	969	0.249	0.234
Total		8969502	389585	100.000	100.000

HPLC of (*R*)-(–)-4a on Chiralcel OJ-H at 30 °C

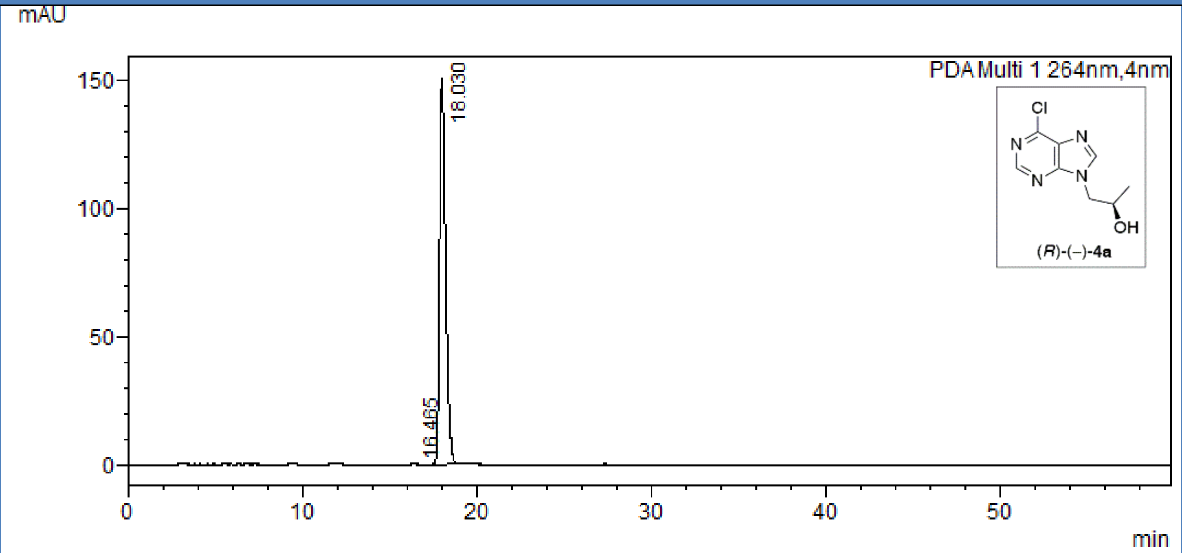

Peak Table

PDA Ch1 264nm

Peak#	Ret. Time	Area	Height	Height%	Area%
1	16.465	3481	221	0.147	0.095
2	18.030	3656845	150479	99.853	99.905
Total		3660325	150700	100.000	100.000

HPLC of *rac*-5a on Chiralcel OD-H at 30 °C

Conditions: *n*-hexane-2-PrOH (95:5, v/v); f=0.9 mL/min; λ=263 nm; *p*=4.0 MPa

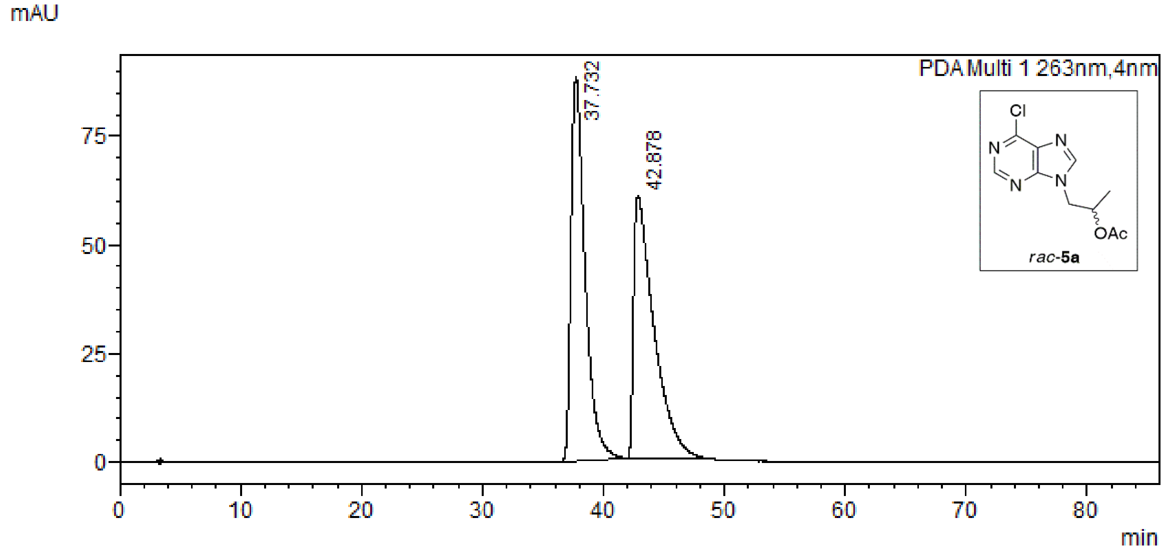

Peak Table

PDA Ch1 263nm

Peak#	Ret. Time	Area	Height	Height%	Area%
1	37.732	7122396	88275	59.346	50.274
2	42.878	7044861	60472	40.654	49.726
Total		14167258	148747	100.000	100.000

HPLC of (*R*)-(–)-5a on Chiralcel OD-H at 30 °C

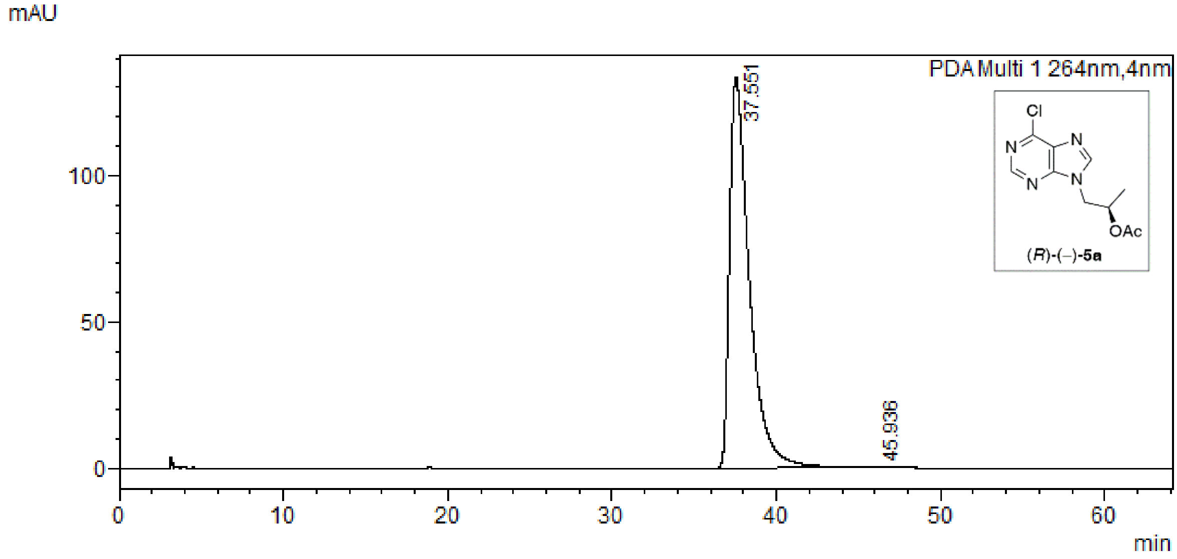

Peak Table

PDA Ch1 264nm

Peak#	Ret. Time	Area	Height	Height%	Area%
1	37.551	11165114	133288	99.763	99.784
2	45.936	24159	316	0.237	0.216
Total		11189273	133604	100.000	100.000

HPLC of *rac*-4b on Chiralcel OJ-H at 30 °C

Conditions: *n*-hexane-2-PrOH (90:10, v/v); f=1.0 mL/min; λ=272 nm; *p*=4.5 MPa

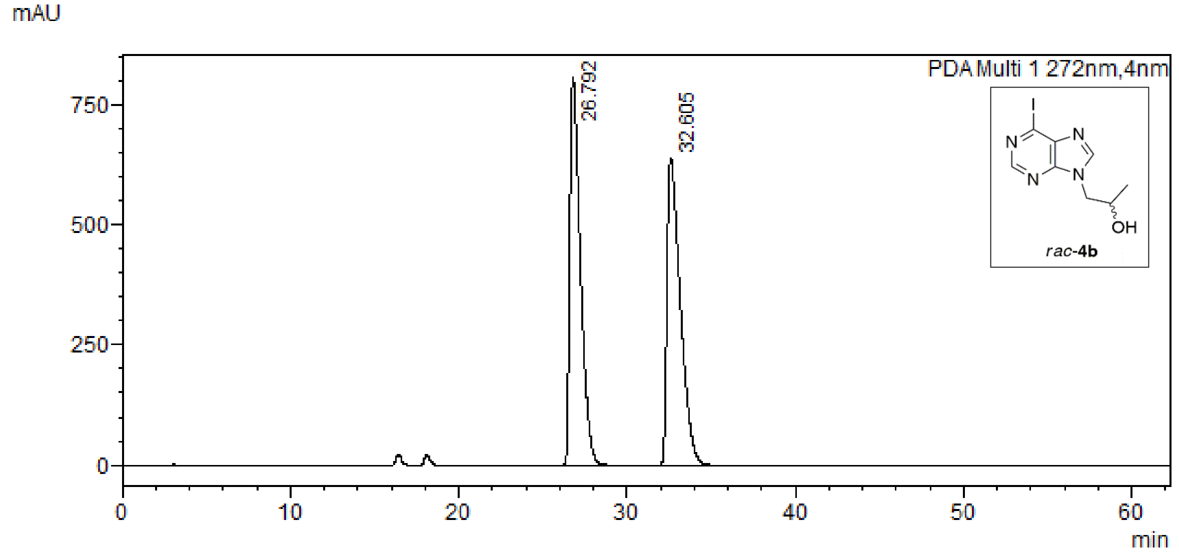

Peak Table

PDA Ch1 272nm

Peak#	Ret. Time	Area	Height	Height%	Area%
1	26.792	35355828	806301	55.753	50.025
2	32.605	35321195	639892	44.247	49.975
Total		70677022	1446193	100.000	100.000

HPLC of (*S*)-(+)-4b on Chiralcel OJ-H at 30 °C

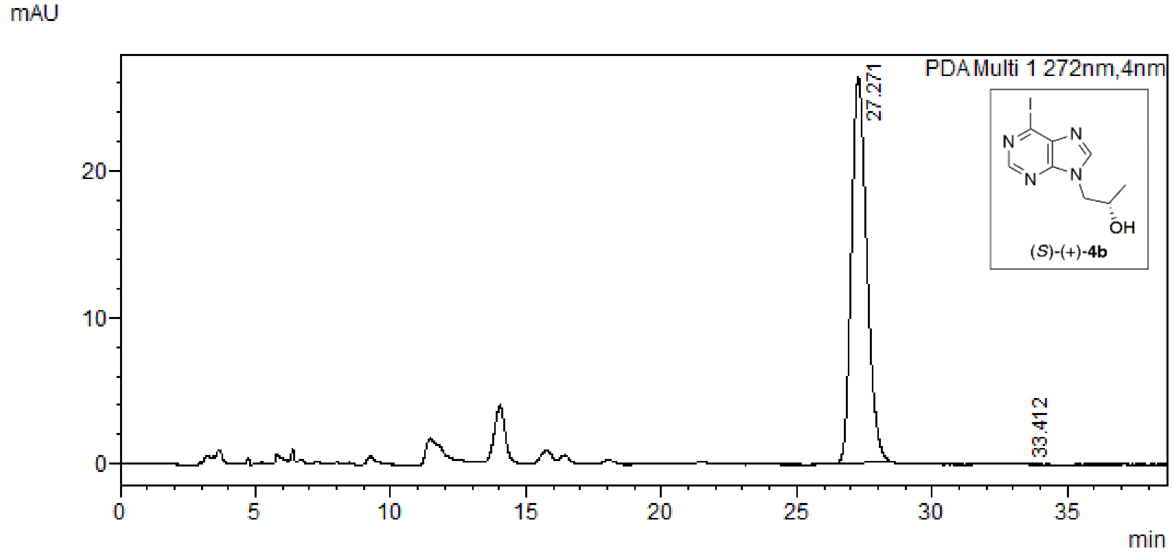

Peak Table

PDA Ch1 272nm

Peak#	Ret. Time	Area	Height	Height%	Area%
1	27.271	1035649	26228	99.746	99.777
2	33.412	2316	67	0.254	0.223
Total		1037965	26295	100.000	100.000

HPLC of (*R*)-(–)-4b on Chiralcel OJ-H at 30 °C

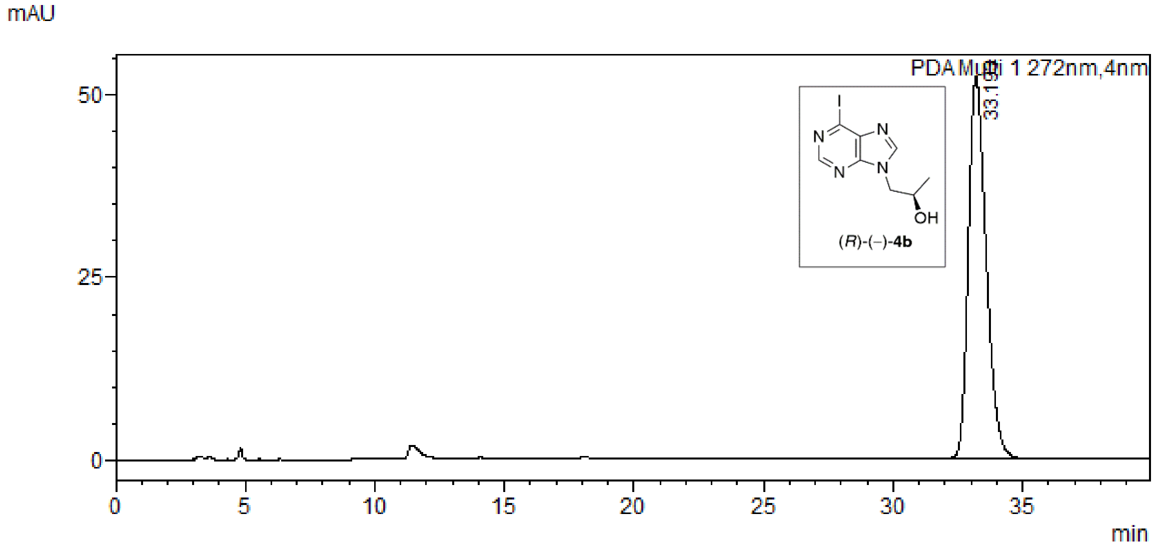

Peak Table

PDA Ch1 272nm

Peak#	Ret. Time	Area	Height	Height%	Area%
1	33.193	2497958	52270	100.000	100.000
Total		2497958	52270	100.000	100.000

HPLC of *rac*-5b on Chiralpak AD-H at 30 °C

Conditions: *n*-hexane-2-PrOH (90:10, v/v); f=0.8 mL/min; λ=272 nm; *p*=3.5 MPa

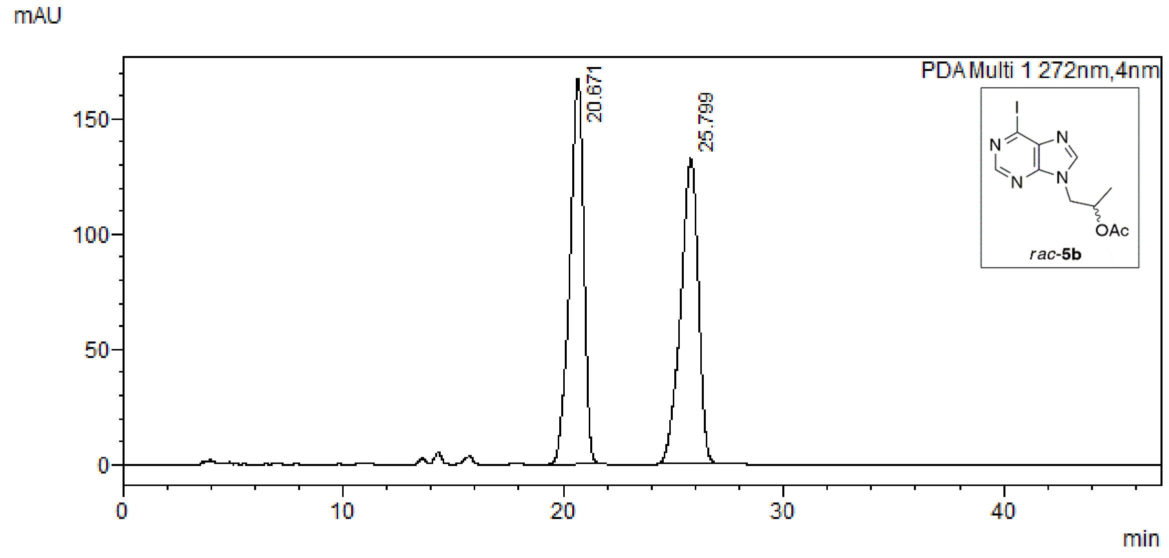

Peak Table

PDA Ch1 272nm

Peak#	Ret. Time	Area	Height	Height%	Area%
1	20.671	7441887	166940	55.753	50.097
2	25.799	7412942	132487	44.247	49.903
Total		14854829	299427	100.000	100.000

HPLC of (*R*)-(+)-5b on Chiralpak AD-H at 30 °C

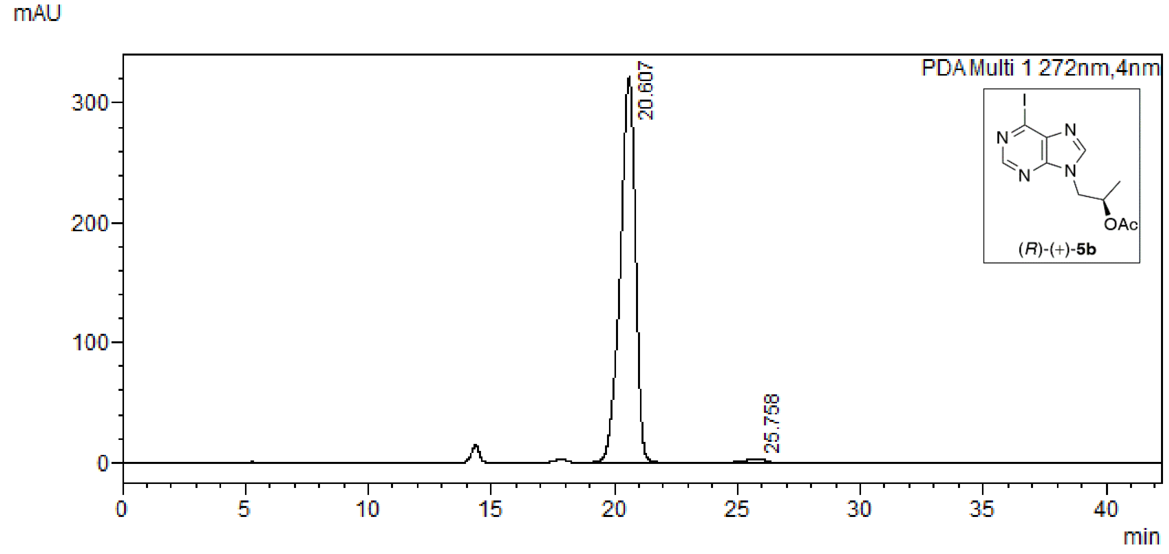

Peak Table

PDA Ch1 272nm

Peak#	Ret. Time	Area	Height	Height%	Area%
1	20.607	13999093	321743	99.009	98.852
2	25.758	162512	3220	0.991	1.148
Total		14161605	324964	100.000	100.000

HPLC analysis for the subsequent biocatalytic reaction:

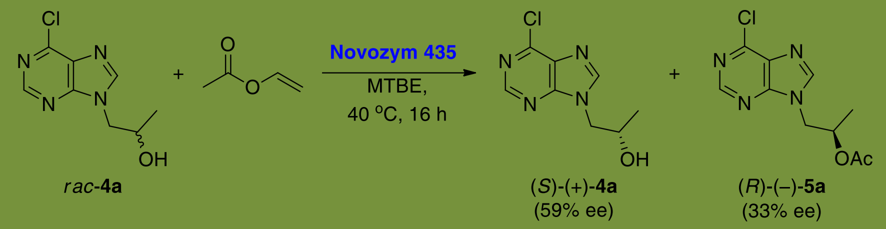

HPLC conditions [for (*S*)-(+)-4a]: *n*-hexane-*i*-PrOH (90:10, v/v); f=1.0 mL/min; λ=264 nm; Chiralcel OJ-H

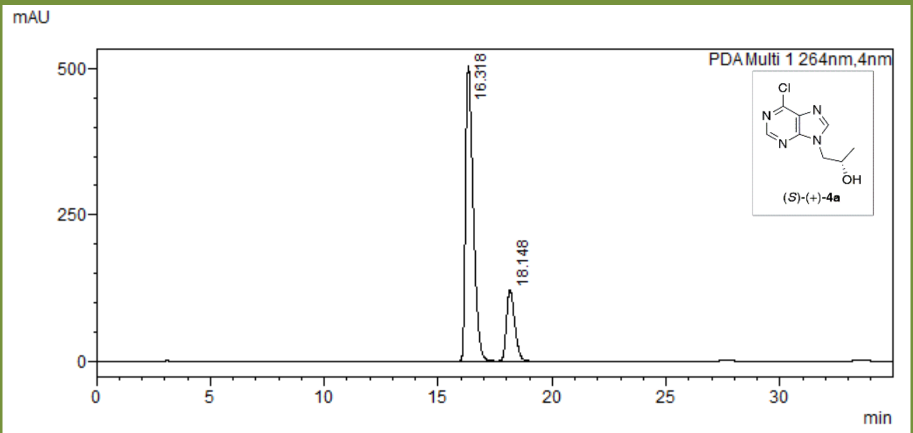

Peak Table

PDA Ch1 264nm

Peak#	Ret. Time	Area	Height	Height%	Area%
1	16.318	11913357	503842	80.540	79.556
2	18.148	3061368	121734	19.460	20.444
Total		14974725	625576	100.000	100.000

HPLC conditions [for (*R*)-(–)-5a]: *n*-hexane-*i*-PrOH (95:5, v/v); f=0.9 mL/min; λ=263 nm; Chiralcel OD-H

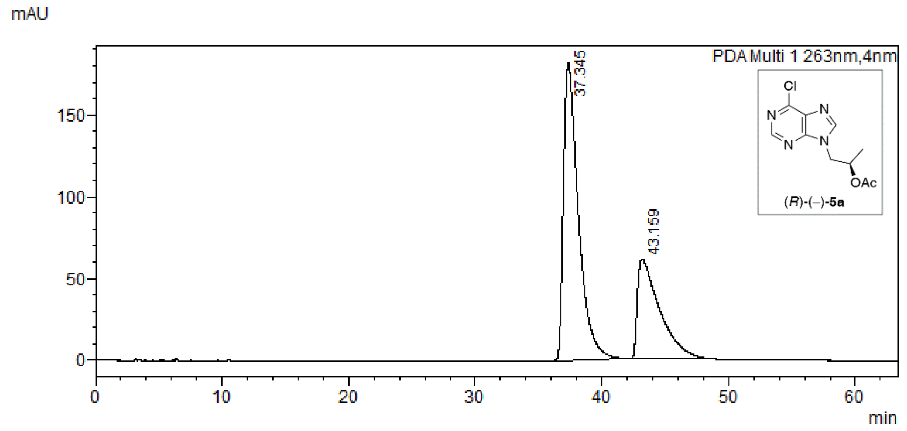

Peak Table

PDA Ch1 263nm

Peak#	Ret. Time	Area	Height	Height%	Area%
1	37.345	14828419	181406	74.794	66.559
2	43.159	7450337	61136	25.206	33.441
Total		22278756	242542	100.000	100.000

HPLC analysis for the subsequent biocatalytic reaction:

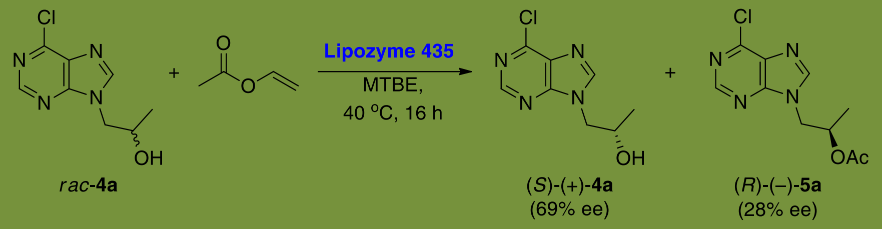

HPLC conditions [for (*S*)-(+)-4a]: *n*-hexane-*i*-PrOH (90:10, v/v); f=1.0 mL/min; λ=264 nm; Chiralcel OJ-H

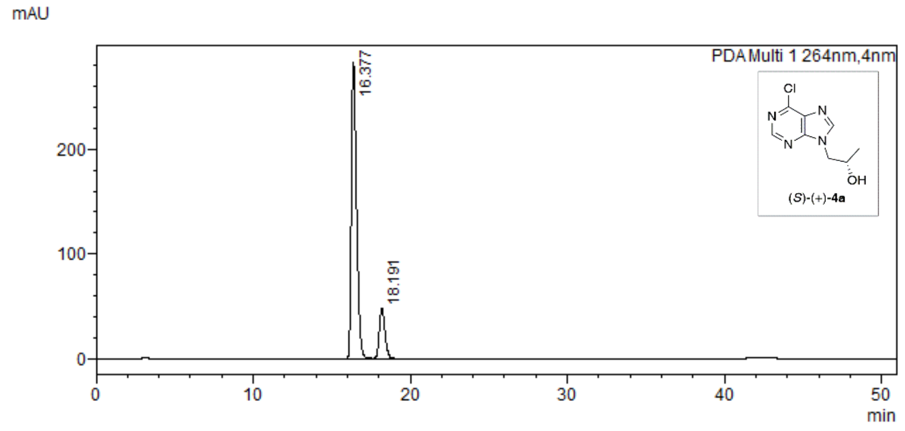

Peak Table

PDA Ch1 264nm

Peak#	Ret. Time	Area	Height	Height%	Area%
1	16.377	6521770	282557	85.512	84.586
2	18.191	1188446	47873	14.488	15.414
Total		7710216	330430	100.000	100.000

HPLC conditions [for (*R*)-(–)-5a]: *n*-hexane-*i*-PrOH (95:5, v/v); f=0.9 mL/min; λ=263 nm; Chiralcel OD-H

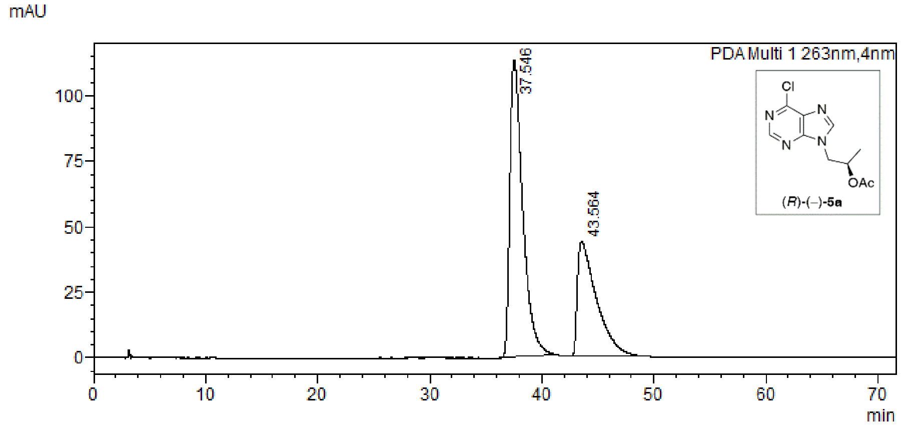

Peak Table

PDA Ch1 263nm

Peak#	Ret. Time	Area	Height	Height%	Area%
1	37.546	9075599	113057	72.120	63.870
2	43.564	5133987	43706	27.880	36.130
Total		14209586	156762	100.000	100.000

HPLC analysis for the subsequent biocatalytic reaction:

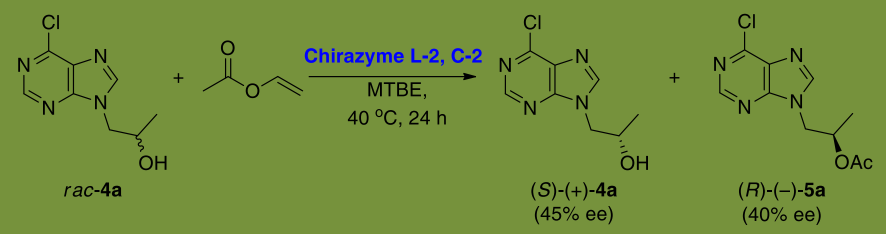

HPLC conditions [for (*S*)-(+)-4a]: *n*-hexane-*i*-PrOH (90:10, v/v); f=1.0 mL/min; λ=264 nm; Chiralcel OJ-H

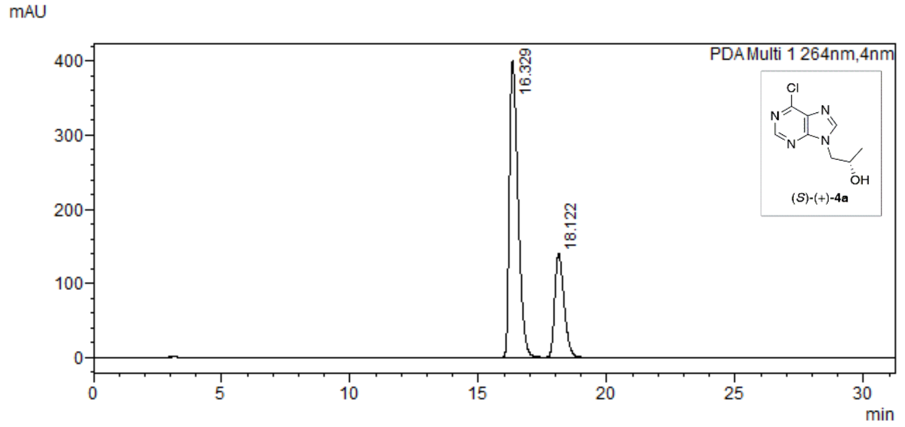

Peak Table

PDA Ch1 264nm

Peak#	Ret. Time	Area	Height	Height%	Area%
1	16.329	9371398	400110	74.110	72.660
2	18.122	3526245	139773	25.890	27.340
Total		12897643	539883	100.000	100.000

HPLC conditions [for (*R*)-(–)-5a]: *n*-hexane-*i*-PrOH (95:5, v/v); f=0.9 mL/min; λ=263 nm; Chiralcel OD-H

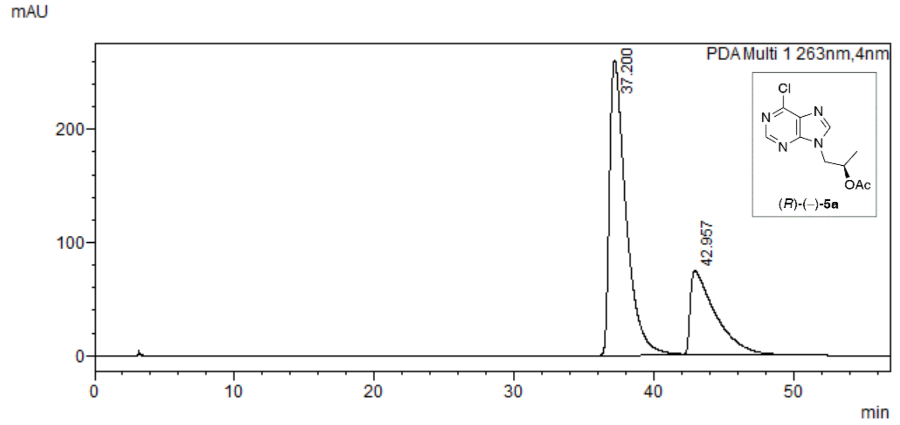

Peak Table

PDA Ch1 263nm

Peak#	Ret. Time	Area	Height	Height%	Area%
1	37.200	21273857	260324	78.051	70.127
2	42.957	9062500	73205	21.949	29.873
Total		30336357	333529	100.000	100.000

HPLC analysis for the subsequent biocatalytic reaction:

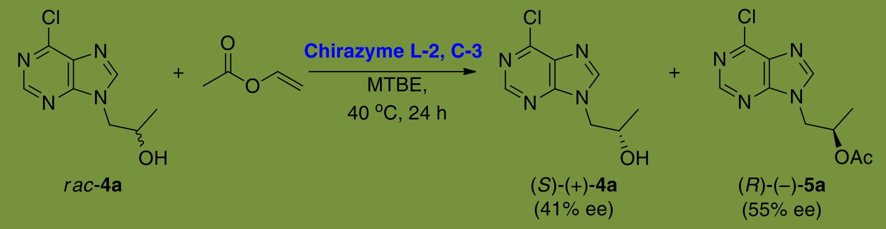

HPLC conditions [for (*S*)-(+)-4a]: *n*-hexane-*i*-PrOH (90:10, v/v); f=1.0 mL/min; λ=264 nm; Chiralcel OJ-H

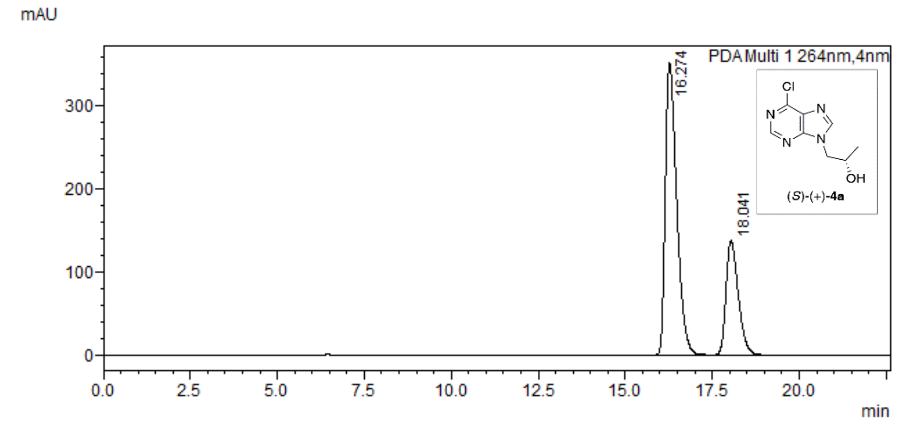

Peak Table

PDA Ch1 264nm

Peak#	Ret. Time	Area	Height	Height%	Area%
1	16.274	8053278	352216	71.877	70.427
2	18.041	3381615	137811	28.123	29.573
Total		11434893	490027	100.000	100.000

HPLC conditions [for (*R*)-(–)-5a]: *n*-hexane-*i*-PrOH (95:5, v/v); f=0.9 mL/min; λ=263 nm; Chiralcel OD-H

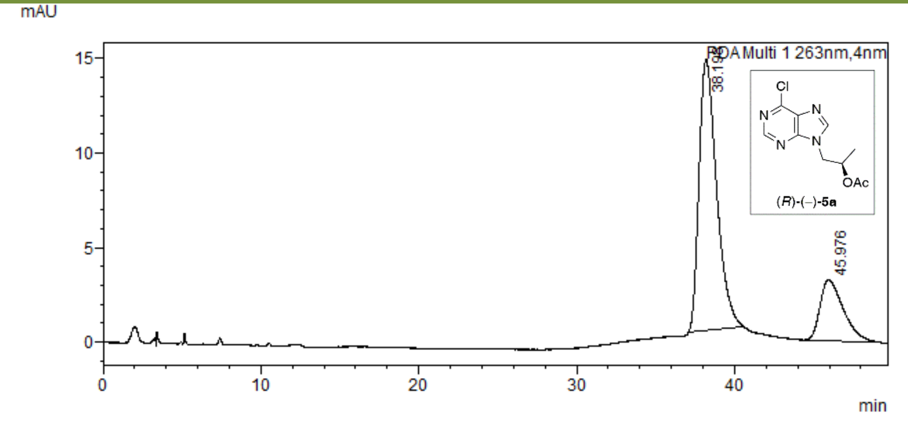

Peak Table

PDA Ch1 263nm

Peak#	Ret. Time	Area	Height	Height%	Area%
1	38.198	1114351	14342	81.698	77.315
2	45.976	326969	3213	18.302	22.685
Total		1441320	17555	100.000	100.000

HPLC analysis for the subsequent biocatalytic reaction:

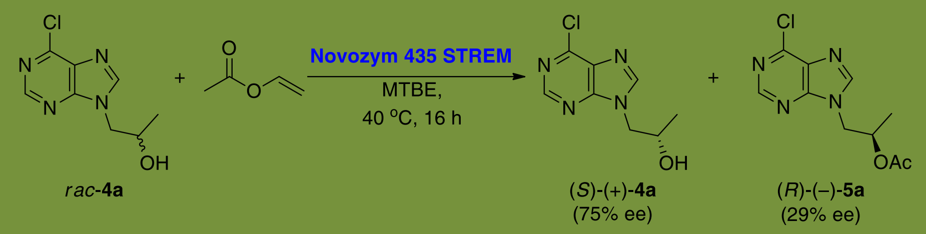

HPLC conditions [for (*S*)-(+)-4a]: *n*-hexane-*i*-PrOH (90:10, v/v); f=1.0 mL/min; λ=264 nm; Chiralcel OJ-H

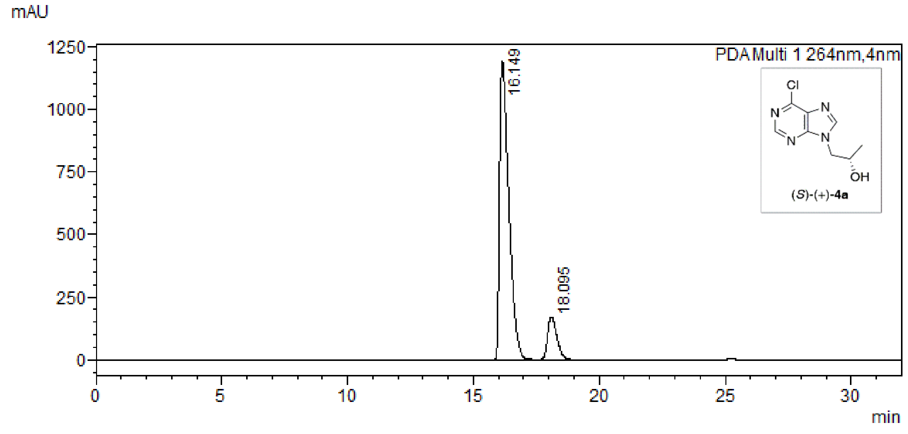

Peak Table

PDA Ch1 264nm

Peak#	Ret. Time	Area	Height	Height%	Area%
1	16.149	30603767	1191693	87.468	87.418
2	18.095	4404657	170748	12.532	12.582
Total		35008424	1362441	100.000	100.000

HPLC conditions [for (*R*)-(–)-5a]: *n*-hexane-*i*-PrOH (95:5, v/v); f=0.9 mL/min; λ=263 nm; Chiralcel OD-H

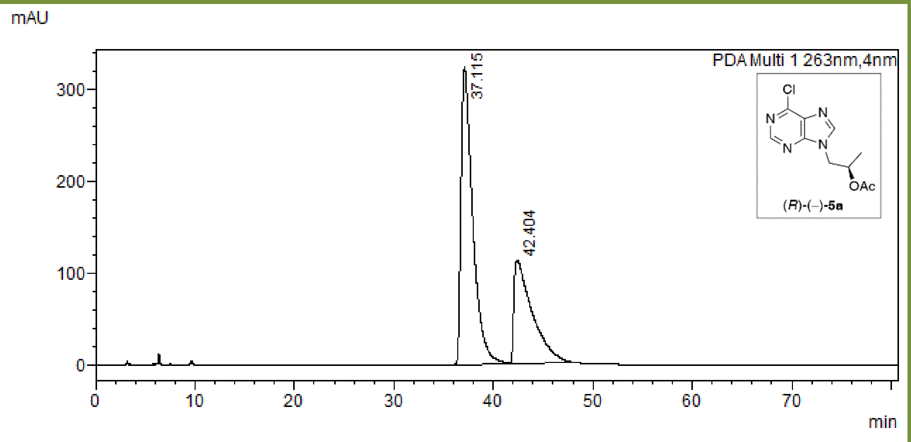

Peak Table

PDA Ch1 263nm

Peak#	Ret. Time	Area	Height	Height%	Area%
1	37.115	26439390	323469	74.157	64.310
2	42.404	14672738	112724	25.843	35.690
Total		41112128	436193	100.000	100.000

HPLC analysis for the subsequent biocatalytic reaction:

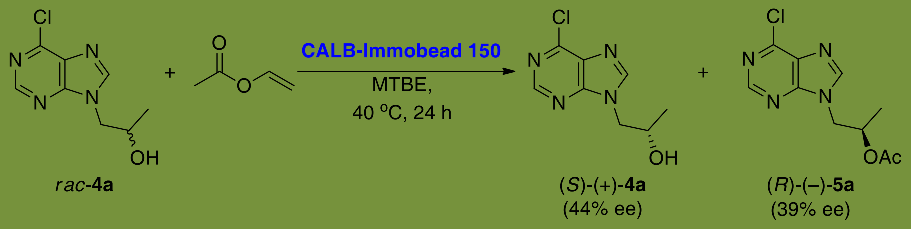

HPLC conditions [for (*S*)-(+)-4a]: *n*-hexane-*i*-PrOH (90:10, v/v); f=1.0 mL/min; λ=264 nm; Chiralcel OJ-H

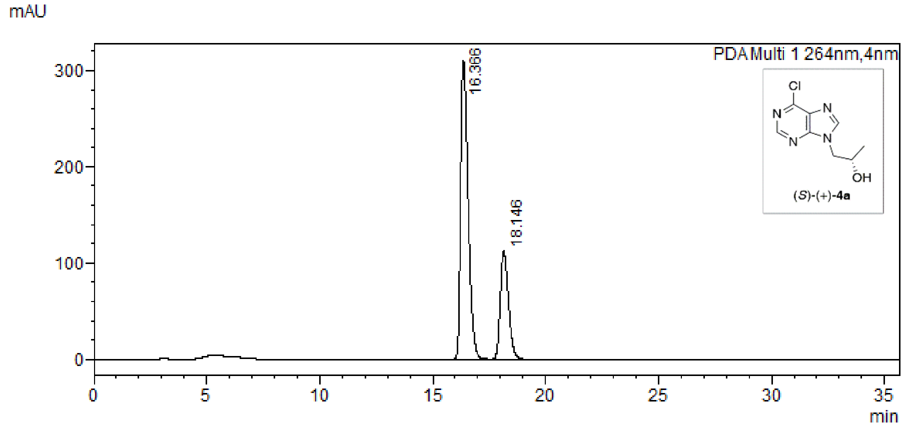

Peak Table

PDA Ch1 264nm

Peak#	Ret. Time	Area	Height	Height%	Area%
1	16.366	7156759	309854	73.402	71.810
2	18.146	2809512	112276	26.598	28.190
Total		9966270	422131	100.000	100.000

HPLC conditions [for (*R*)-(–)-5a]: *n*-hexane-*i*-PrOH (95:5, v/v); f=0.9 mL/min; λ=263 nm; Chiralcel OD-H

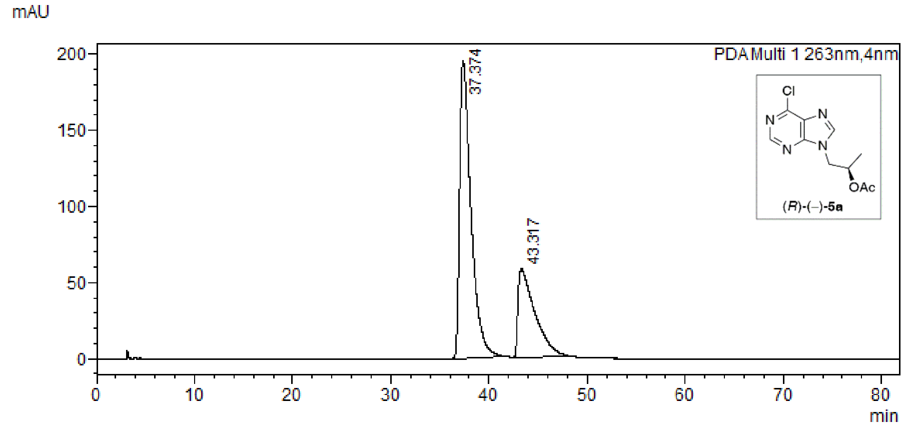

Peak Table

PDA Ch1 263nm

Peak#	Ret. Time	Area	Height	Height%	Area%
1	37.374	15887952	195241	77.121	69.456
2	43.317	6986843	57922	22.879	30.544
Total		22874795	253163	100.000	100.000

HPLC analysis for the subsequent biocatalytic reaction:

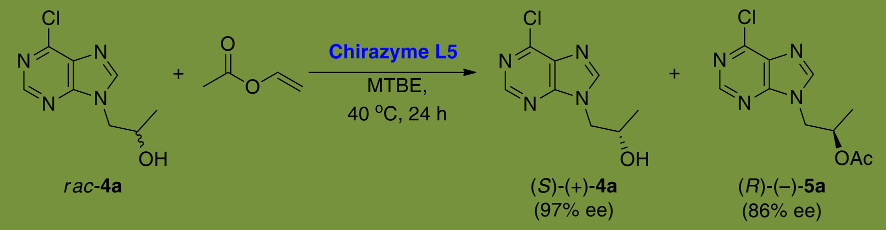

HPLC conditions [for (*S*)-(+)-4a]: *n*-hexane-*i*-PrOH (90:10, v/v); f=1.0 mL/min; λ=264 nm; Chiralcel OJ-H

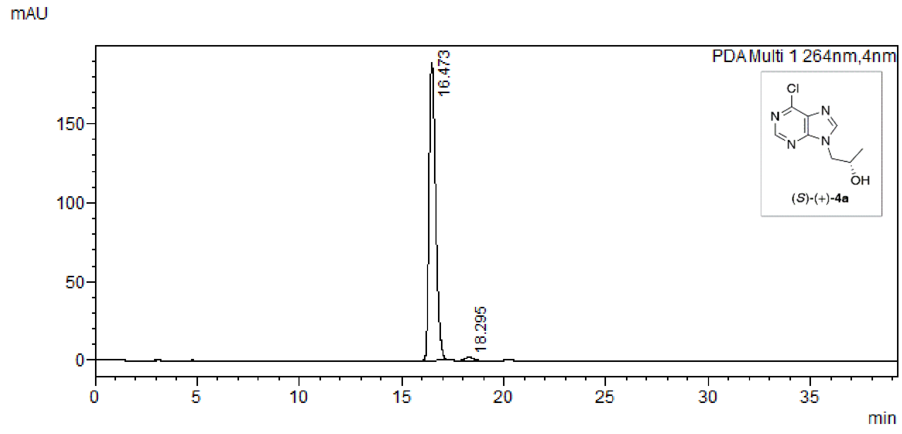

Peak Table

PDA Ch1 264nm

Peak#	Ret. Time	Area	Height	Height%	Area%
1	16.473	4217728	188725	98.651	98.603
2	18.295	59772	2581	1.349	1.397
Total		4277501	191306	100.000	100.000

HPLC conditions [for (*R*)-(–)-5a]: *n*-hexane-*i*-PrOH (95:5, v/v); f=0.9 mL/min; λ=263 nm; Chiralcel OD-H

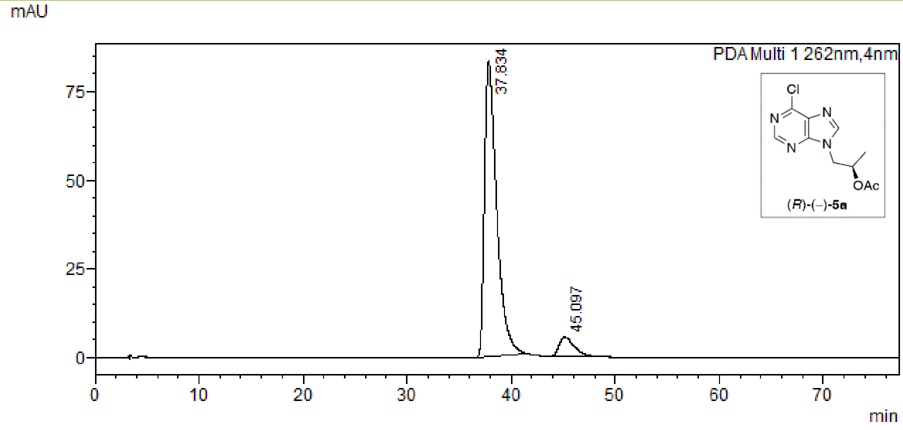

Peak Table

PDA Ch1 262nm

Peak#	Ret. Time	Area	Height	Height%	Area%
1	37.834	6782515	83362	94.032	93.098
2	45.097	502811	5291	5.968	6.902
Total		7285326	88653	100.000	100.000

HPLC analysis for the subsequent biocatalytic reaction:

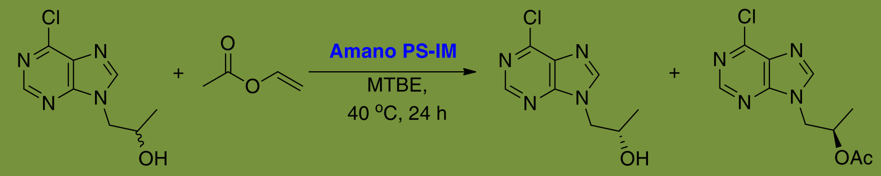

rac-**4a** (*S*)-(+)-**4a** (>99% ee) (*R*)-(–)-**5a** (97% ee)

HPLC conditions [for (*S*)-(+)-4a]: *n*-hexane-*i*-PrOH (90:10, v/v); f=1.0 mL/min; λ=264 nm; Chiralcel OJ-H

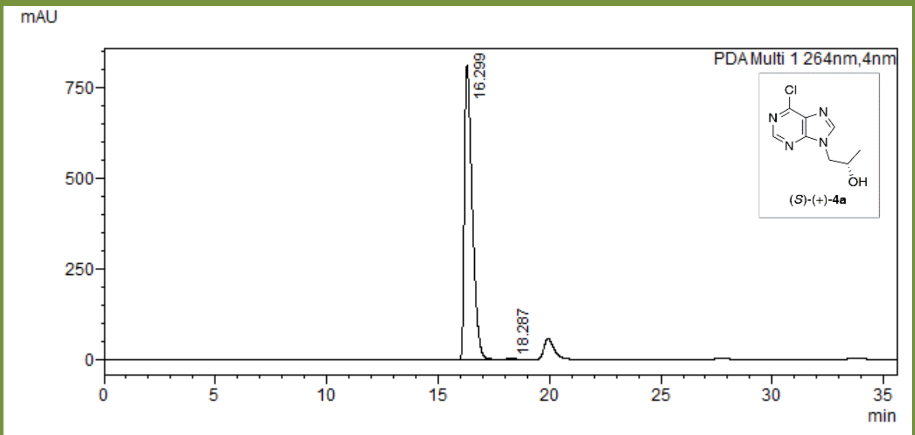

Peak Table

PDA Ch1 264nm

Peak#	Ret. Time	Area	Height	Height%	Area%
1	16.299	19787046	809856	99.749	99.762
2	18.287	47207	2037	0.251	0.238
Total		19834253	811893	100.000	100.000

HPLC conditions [for (*R*)-(–)-5a]: *n*-hexane-*i*-PrOH (95:5, v/v); f=0.9 mL/min; λ=263 nm; Chiralcel OD-H

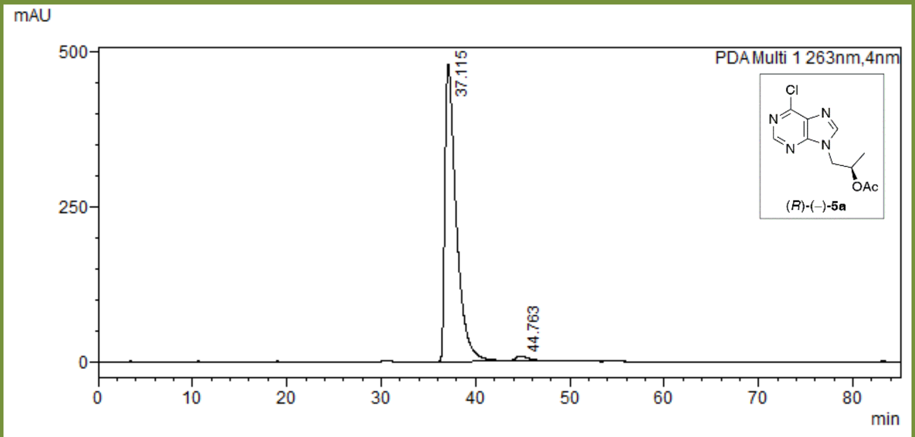

Peak Table

PDA Ch1 263nm

Peak#	Ret. Time	Area	Height	Height%	Area%
1	37.115	40144147	478988	98.465	98.254
2	44.763	713267	7465	1.535	1.746
Total		40857414	486453	100.000	100.000

HPLC analysis for the subsequent biocatalytic reaction:

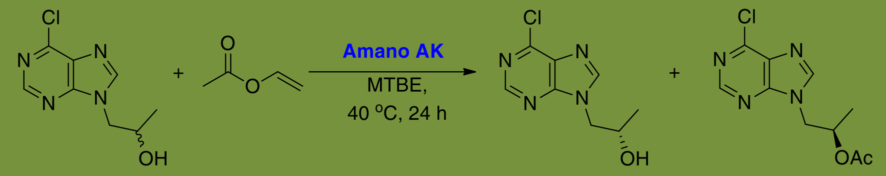

rac-**4a** (*S*)-(+)-**4a** (37% ee) (*R*)-(–)-**5a** (98% ee)

HPLC conditions [for (*S*)-(+)-4a]: *n*-hexane-*i*-PrOH (90:10, v/v); f=1.0 mL/min; λ=264 nm; Chiralcel OJ-H

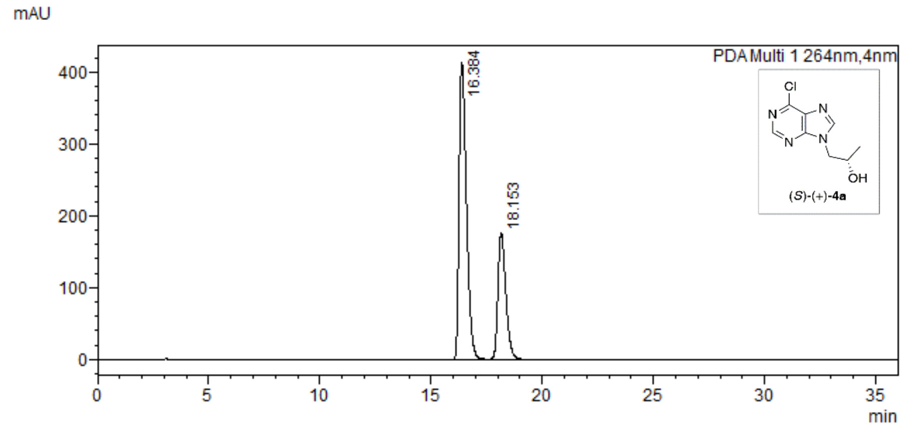

Peak Table

PDA Ch1 264nm

Peak#	Ret. Time	Area	Height	Height%	Area%
1	16.384	9482304	412827	70.159	68.674
2	18.153	4325414	175588	29.841	31.326
Total		13807718	588415	100.000	100.000

HPLC conditions [for (*R*)-(–)-5a]: *n*-hexane-*i*-PrOH (95:5, v/v); f=0.9 mL/min; λ=263 nm; Chiralcel OD-H

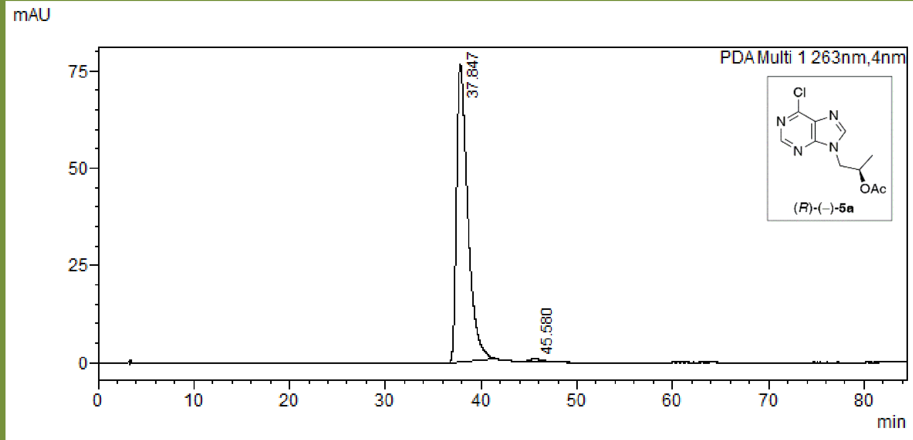

Peak Table

PDA Ch1 263nm

Peak#	Ret. Time	Area	Height	Height%	Area%
1	37.847	6204204	76330	99.146	99.057
2	45.580	59070	657	0.854	0.943
Total		6263274	76987	100.000	100.000

HPLC analysis for the subsequent biocatalytic reaction:

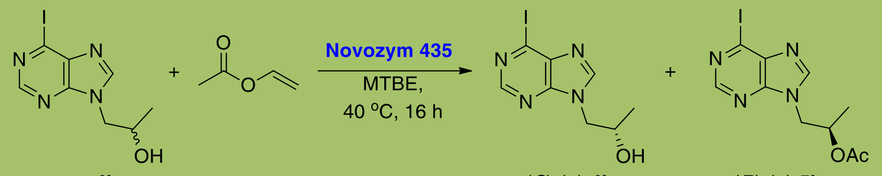

rac-**4b** (*S*)-(+)-**4b** (79% ee) (*R*)-(–)-**5b** (28% ee)

HPLC conditions [for (*S*)-(+)-4b]: *n*-hexane-*i*-PrOH (90:10, v/v); f=1.0 mL/min; λ=272 nm; Chiralcel OJ-H

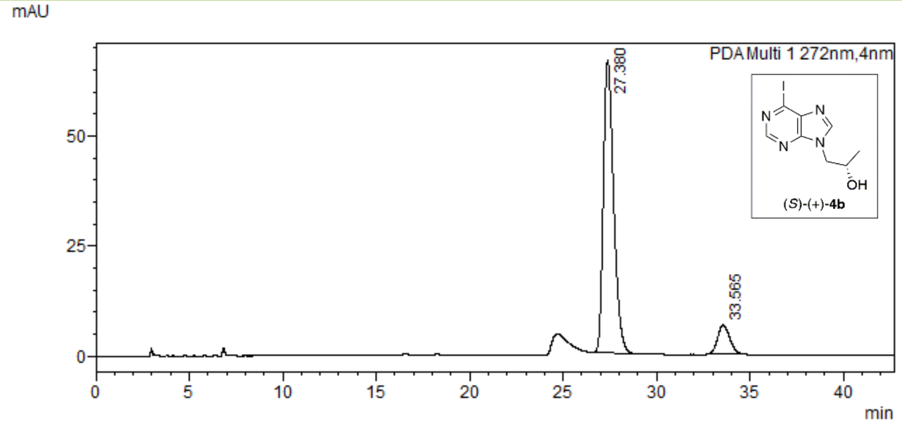

Peak Table

PDA Ch1 272nm

Peak#	Ret. Time	Area	Height	Height%	Area%
1	27.380	2546709	66432	90.928	89.542
2	33.565	297440	6628	9.072	10.458
Total		2844149	73059	100.000	100.000

HPLC conditions [for (*R*)-(+)-5b]: *n*-hexane-*i*-PrOH (90:10, v/v); f=0.8 mL/min; λ=272 nm; Chiralpak AD-H

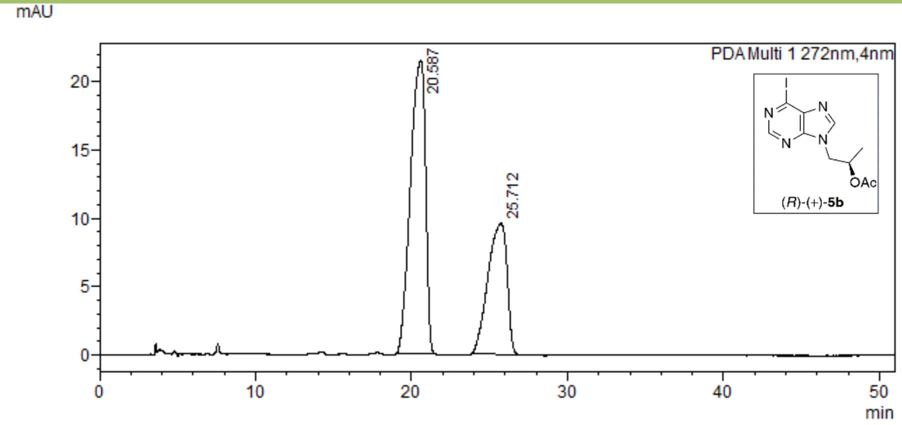

Peak Table

PDA Ch1 272nm

Peak#	Ret. Time	Area	Height	Height%	Area%
1	20.587	1450422	21459	69.223	64.175
2	25.712	809665	9541	30.777	35.825
Total		2260088	31000	100.000	100.000

HPLC analysis for the subsequent biocatalytic reaction:

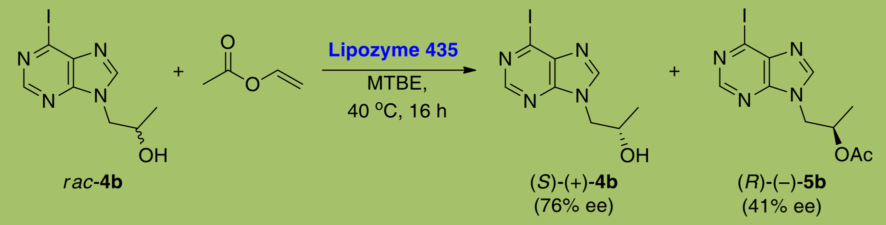

HPLC conditions [for (*S*)-(+)-4b]: *n*-hexane-*i*-PrOH (90:10, v/v); f=1.0 mL/min; λ=272 nm; Chiralcel OJ-H

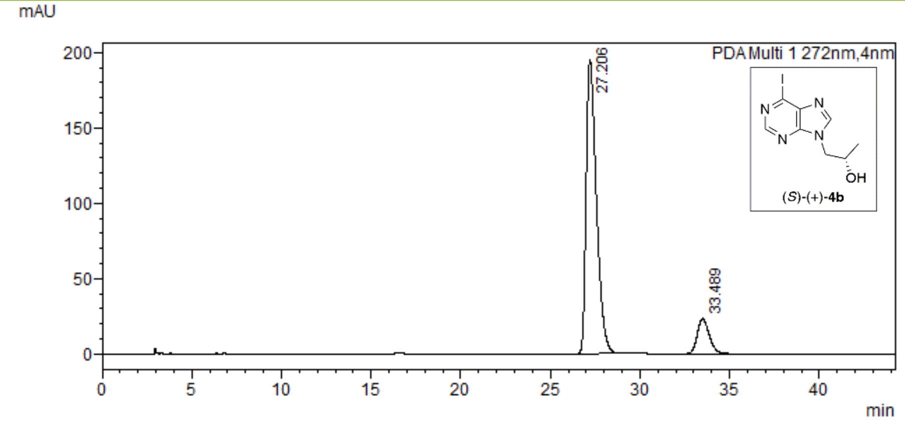

Peak Table

PDA Ch1 272nm

Peak#	Ret. Time	Area	Height	Height%	Area%
1	27.206	7762617	194839	89.346	87.935
2	33.489	1065036	23233	10.654	12.065
Total		8827654	218072	100.000	100.000

HPLC conditions [for (*R*)-(+)-5b]: *n*-hexane-*i*-PrOH (90:10, v/v); f=0.8 mL/min; λ=272 nm; Chiralpak AD-H

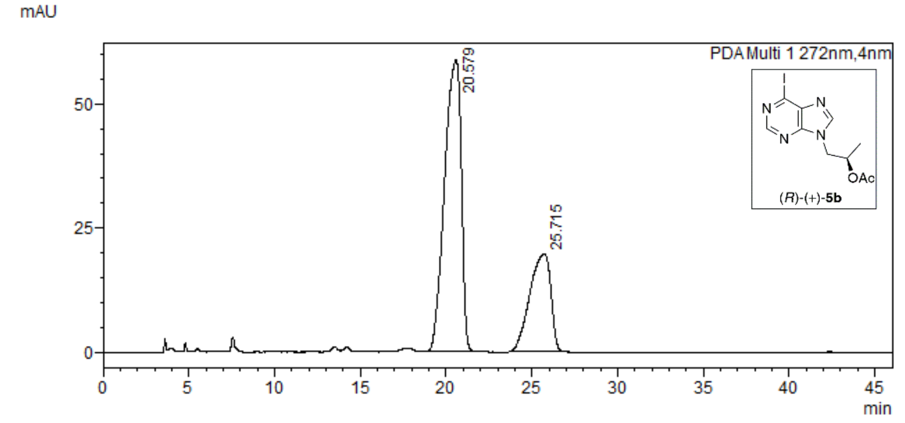

Peak Table

PDA Ch1 272nm

Peak#	Ret. Time	Area	Height	Height%	Area%
1	20.579	3981330	58668	74.927	70.365
2	25.715	1676769	19632	25.073	29.635
Total		5658099	78300	100.000	100.000

HPLC analysis for the subsequent biocatalytic reaction:

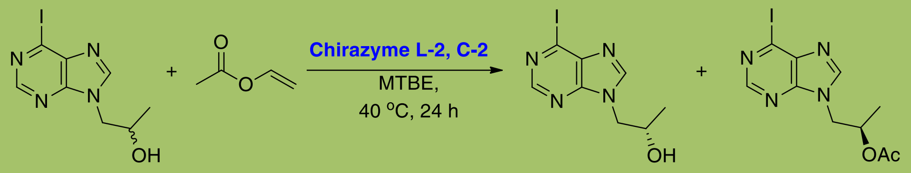

rac-**4b** (*S*)-(+)-**4b** (59% ee) (*R*)-(–)-**5b** (42% ee)

HPLC conditions [for (*S*)-(+)-4b]: *n*-hexane-*i*-PrOH (90:10, v/v); f=1.0 mL/min; λ=272 nm; Chiralcel OJ-H

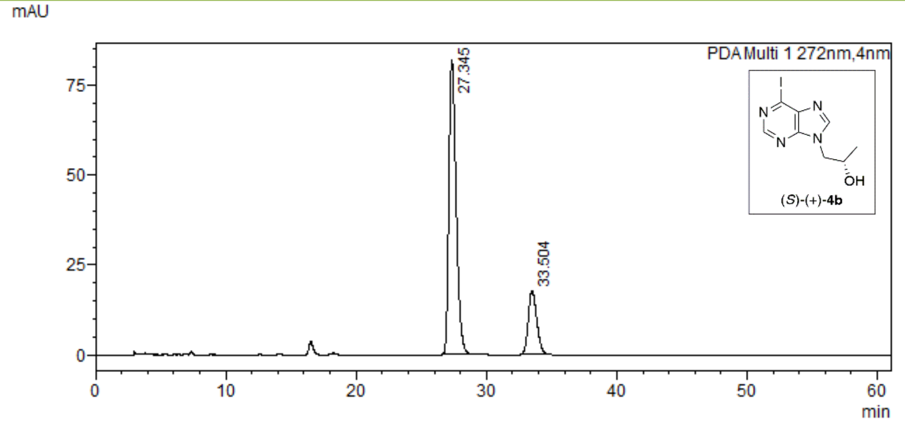

Peak Table

PDA Ch1 272nm

Peak#	Ret. Time	Area	Height	Height%	Area%
1	27.345	3162073	81845	82.159	79.622
2	33.504	809296	17773	17.841	20.378
Total		3971369	99617	100.000	100.000

HPLC conditions [for (*R*)-(+)-5b]: *n*-hexane-*i*-PrOH (90:10, v/v); f=0.8 mL/min; λ=272 nm; Chiralpak AD-H

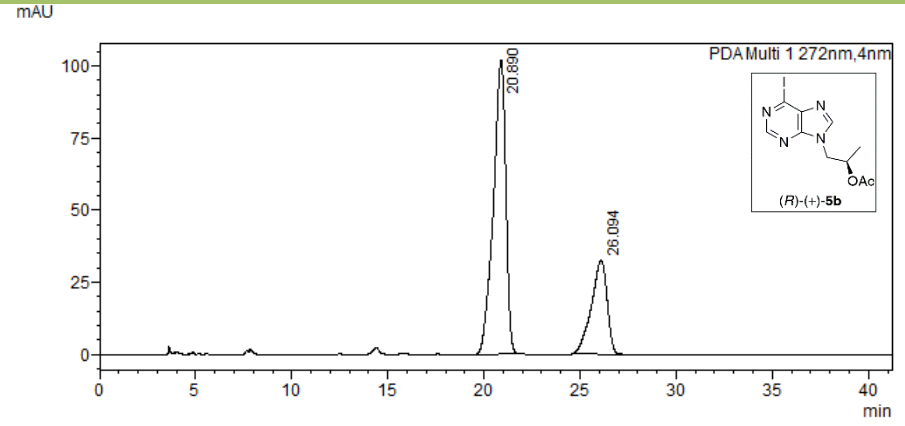

Peak Table

PDA Ch1 272nm

Peak#	Ret. Time	Area	Height	Height%	Area%
1	20.890	4595757	101677	75.749	71.239
2	26.094	1855382	32552	24.251	28.761
Total		6451139	134229	100.000	100.000

HPLC analysis for the subsequent biocatalytic reaction:

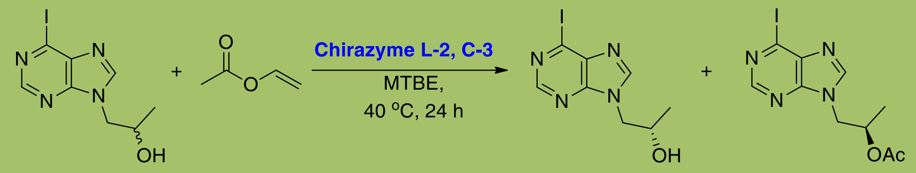

rac-**4b** (*S*)-(+)-**4b** (38% ee) (*R*)-(–)-**5b** (67% ee)

HPLC conditions [for (*S*)-(+)-4b]: *n*-hexane-*i*-PrOH (90:10, v/v); f=1.0 mL/min; λ=272 nm; Chiralcel OJ-H

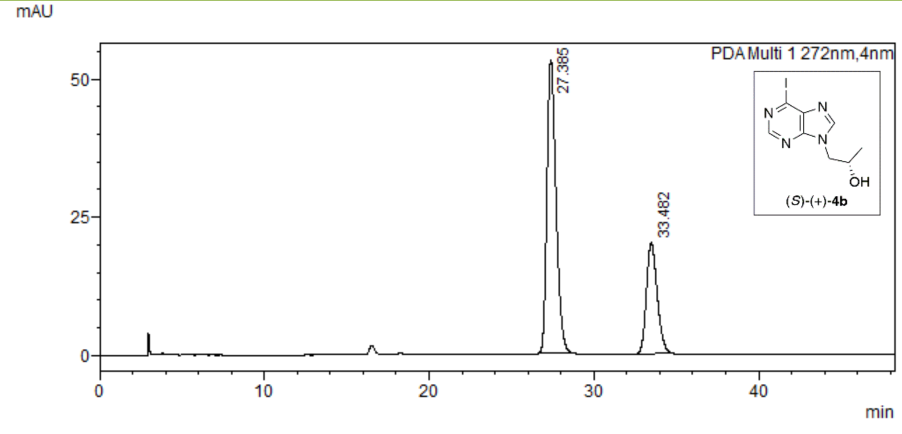

Peak Table

PDA Ch1 272nm

Peak#	Ret. Time	Area	Height	Height%	Area%
1	27.385	2033072	53080	72.526	68.872
2	33.482	918891	20107	27.474	31.128
Total		2951963	73187	100.000	100.000

HPLC conditions [for (*R*)-(+)-5b]: *n*-hexane-*i*-PrOH (90:10, v/v); f=0.8 mL/min; λ=272 nm; Chiralpak AD-H

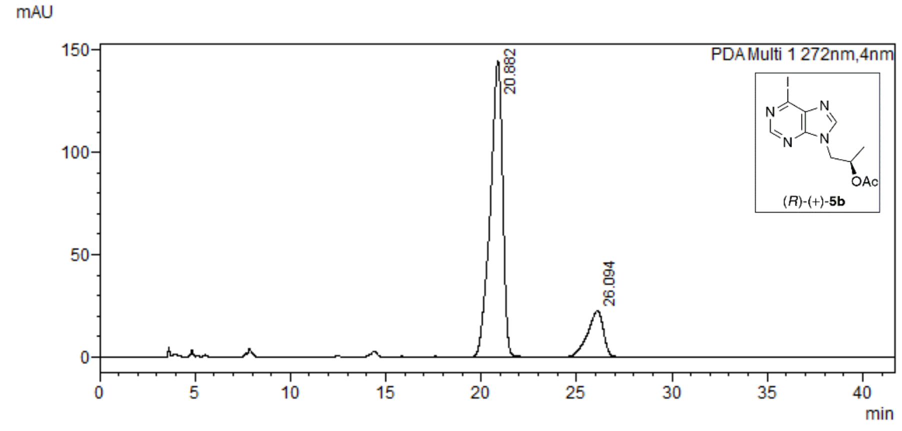

Peak Table

PDA Ch1 272nm

Peak#	Ret. Time	Area	Height	Height%	Area%
1	20.882	6533471	144495	86.625	83.723
2	26.094	1270191	22310	13.375	16.277
Total		7803662	166805	100.000	100.000

HPLC analysis for the subsequent biocatalytic reaction:

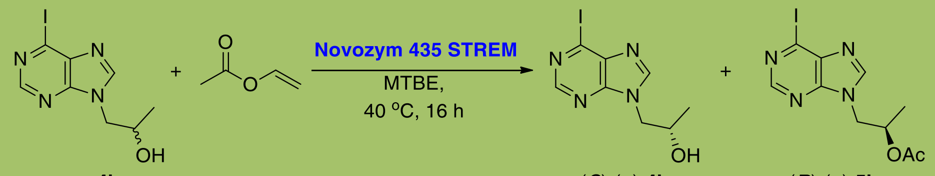

rac-**4b** (*S*)-(+)-**4b** (83% ee) (*R*)-(–)-**5b** (25% ee)

HPLC conditions [for (*S*)-(+)-4b]: *n*-hexane-*i*-PrOH (90:10, v/v); f=1.0 mL/min; λ=272 nm; Chiralcel OJ-H

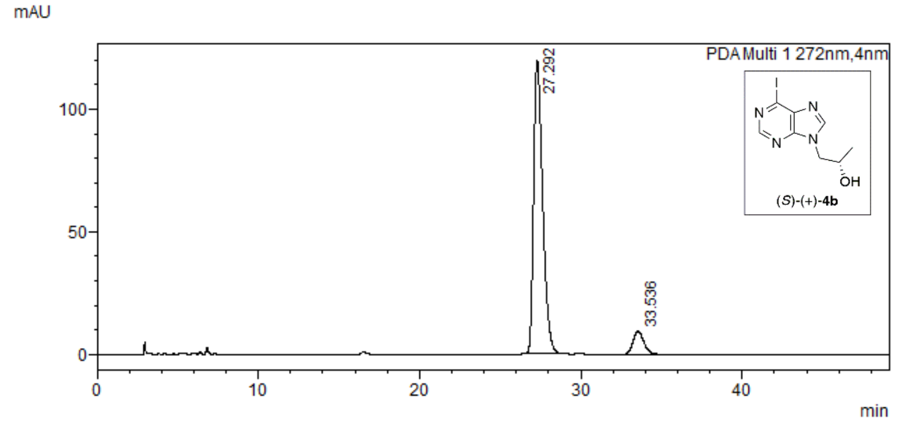

Peak Table

PDA Ch1 272nm

Peak#	Ret. Time	Area	Height	Height%	Area%
1	27.292	4662763	119522	92.624	91.526
2	33.536	431707	9518	7.376	8.474
Total		5094470	129040	100.000	100.000

HPLC conditions [for (*R*)-(+)-5b]: *n*-hexane-*i*-PrOH (90:10, v/v); f=0.8 mL/min; λ=272 nm; Chiralpak AD-H

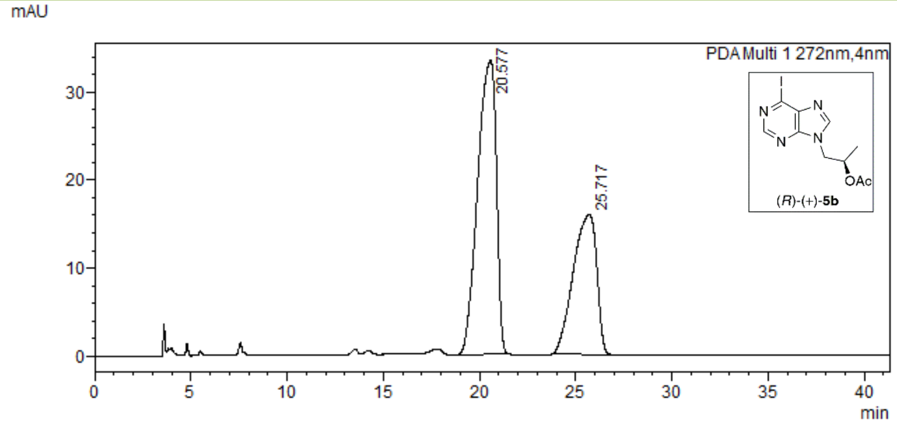

Peak Table

PDA Ch1 272nm

Peak#	Ret. Time	Area	Height	Height%	Area%
1	20.577	2261341	33405	67.821	62.678
2	25.717	1346531	15850	32.179	37.322
Total		3607872	49256	100.000	100.000

HPLC analysis for the subsequent biocatalytic reaction:

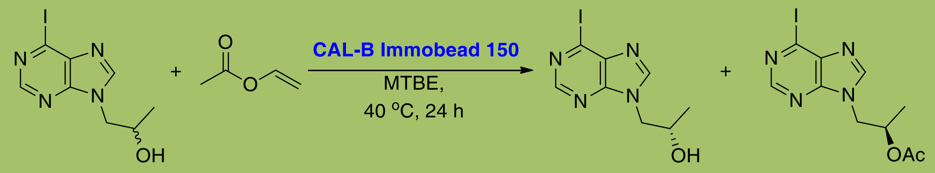

rac-**4b** (*S*)-(+)-**4b** (65% ee) (*R*)-(–)-**5b** (43% ee)

HPLC conditions [for (*S*)-(+)-4b]: *n*-hexane-*i*-PrOH (90:10, v/v); f=1.0 mL/min; λ=272 nm; Chiralcel OJ-H

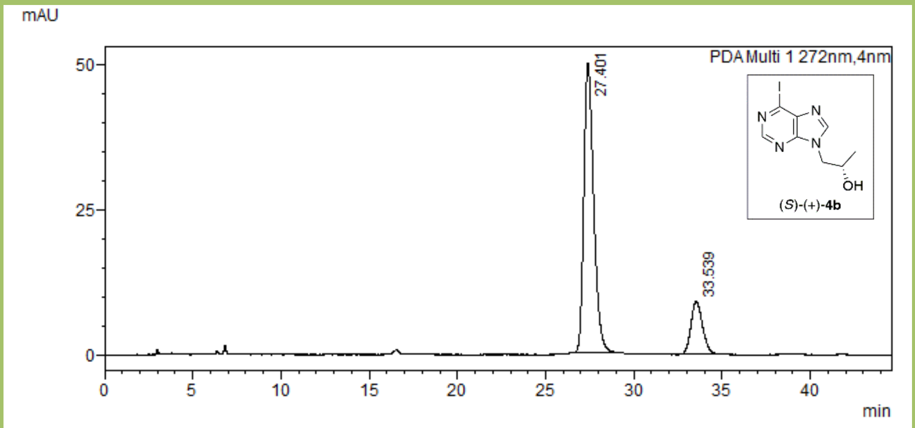

Peak Table

PDA Ch1 272nm

Peak#	Ret. Time	Area	Height	Height%	Area%
1	27.401	1927345	49878	84.532	82.372
2	33.539	412467	9127	15.468	17.628
Total		2339812	59005	100.000	100.000

HPLC conditions [for (*R*)-(+)-5b]: *n*-hexane-*i*-PrOH (90:10, v/v); f=0.8 mL/min; λ=272 nm; Chiralpak AD-H

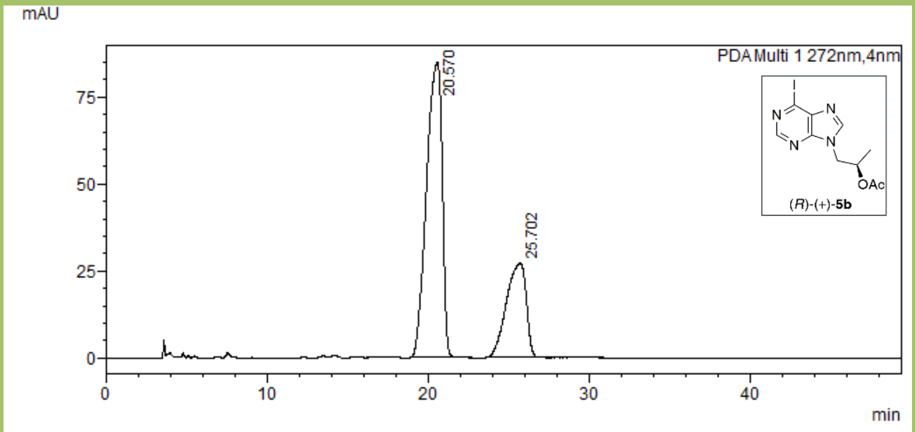

Peak Table

PDA Ch1 272nm

Peak#	Ret. Time	Area	Height	Height%	Area%
1	20.570	5743481	84731	75.817	71.313
2	25.702	2310463	27026	24.183	28.687
Total		8053945	111757	100.000	100.000

HPLC analysis for the subsequent biocatalytic reaction:

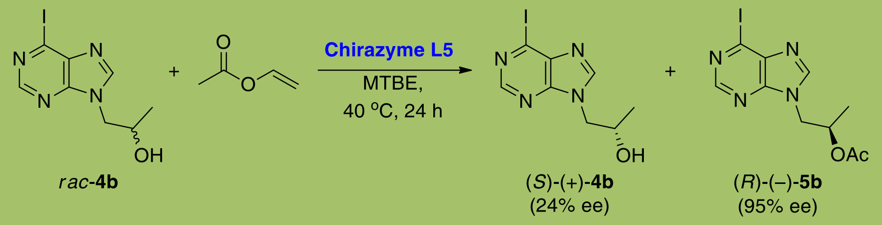

HPLC conditions [for (*S*)-(+)-4b]: *n*-hexane-*i*-PrOH (90:10, v/v); f=1.0 mL/min; λ=272 nm; Chiralcel OJ-H

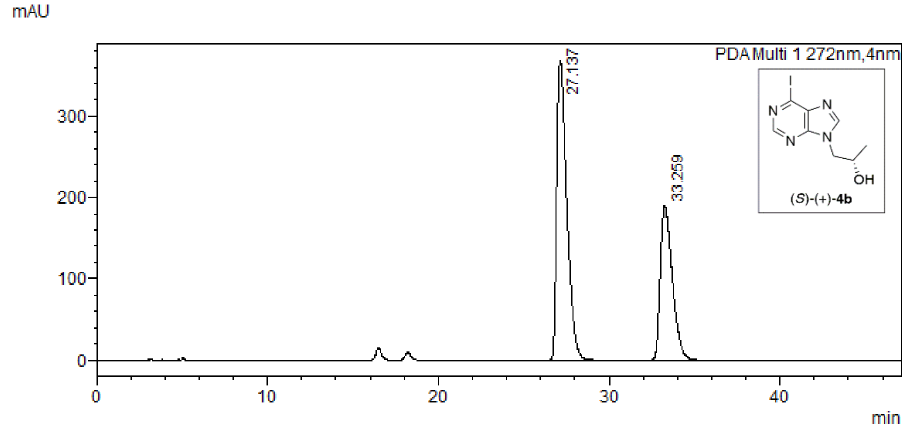

Peak Table

PDA Ch1 272nm

Peak#	Ret. Time	Area	Height	Height%	Area%
1	27.137	15104648	367507	65.907	61.913
2	33.259	9292023	190111	34.093	38.087
Total		24396671	557619	100.000	100.000

HPLC conditions [for (*R*)-(+)-5b]: *n*-hexane-*i*-PrOH (90:10, v/v); f=0.8 mL/min; λ=272 nm; Chiralpak AD-H

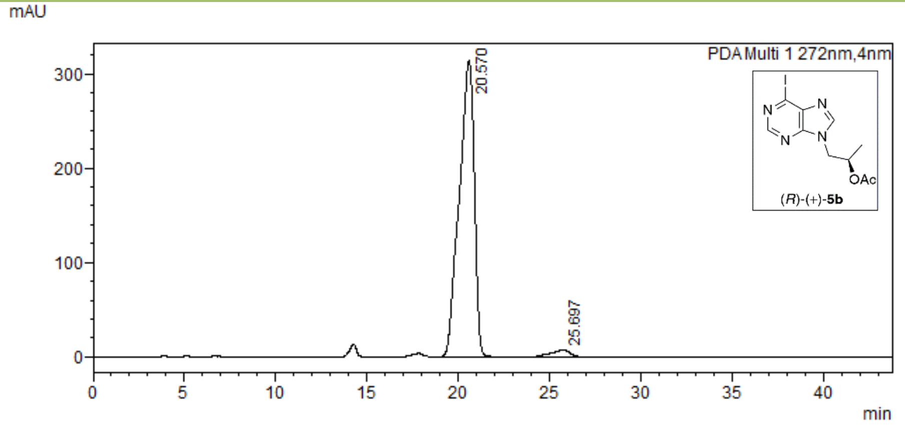

Peak Table

PDA Ch1 272nm

Peak#	Ret. Time	Area	Height	Height%	Area%
1	20.570	17990826	313715	97.902	97.440
2	25.697	472615	6724	2.098	2.560
Total		18463441	320438	100.000	100.000

HPLC analysis for the subsequent biocatalytic reaction:

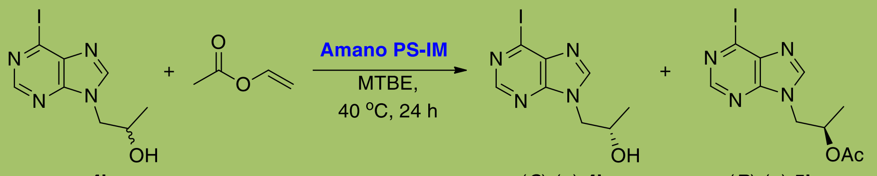

HPLC conditions [for (*S*)-(+)-4b]: *n*-hexane-*i*-PrOH (90:10, v/v); f=1.0 mL/min; λ=272 nm; Chiralcel OJ-H

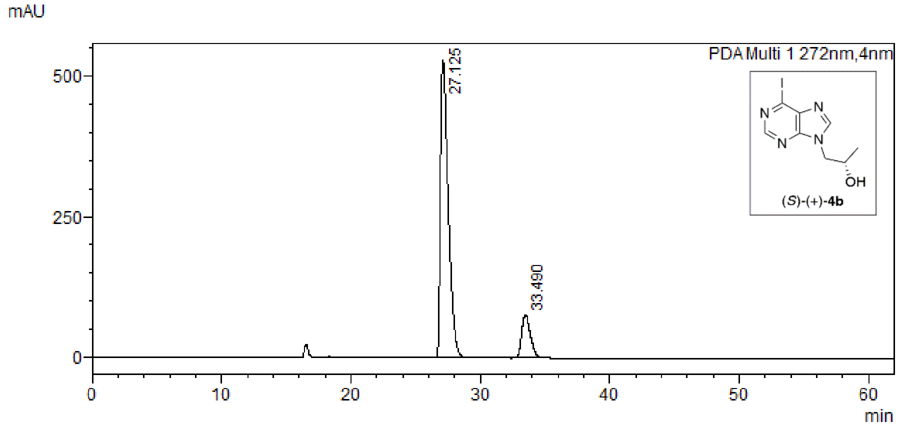

Peak Table

PDA Ch1 272nm

Peak#	Ret. Time	Area	Height	Height%	Area%
1	27.125	22463428	528717	87.220	86.060
2	33.490	3638739	77474	12.780	13.940
Total		26102166	606190	100.000	100.000

HPLC conditions [for (*R*)-(+)-5b]: *n*-hexane-*i*-PrOH (90:10, v/v); f=0.8 mL/min; λ=272 nm; Chiralpak AD-H

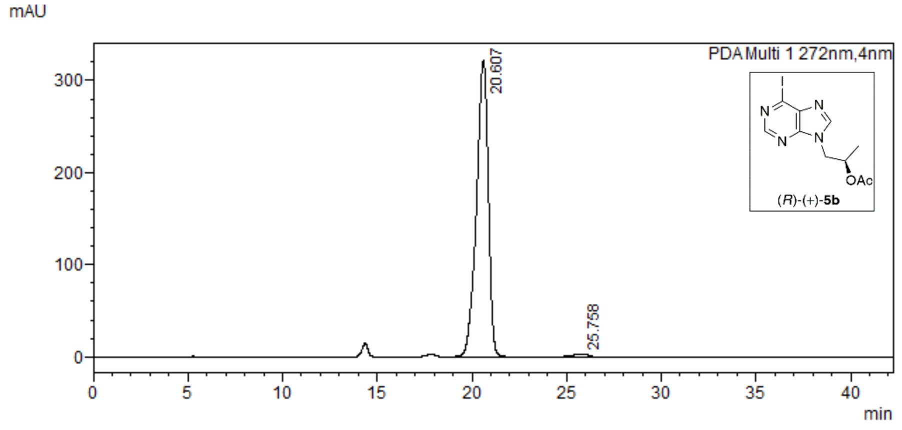

Peak Table

PDA Ch1 272nm

Peak#	Ret. Time	Area	Height	Height%	Area%
1	20.607	13999093	321743	99.009	98.852
2	25.758	162512	3220	0.991	1.148
Total		14161605	324964	100.000	100.000

HPLC analysis for the subsequent biocatalytic reaction:

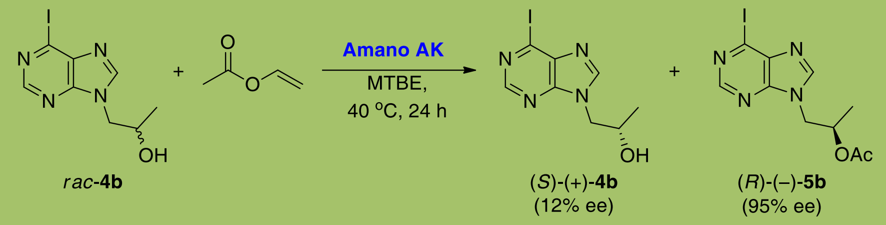

HPLC conditions [for (*S*)-(+)-4b]: *n*-hexane-*i*-PrOH (90:10, v/v); f=1.0 mL/min; λ=272 nm; Chiralcel OJ-H

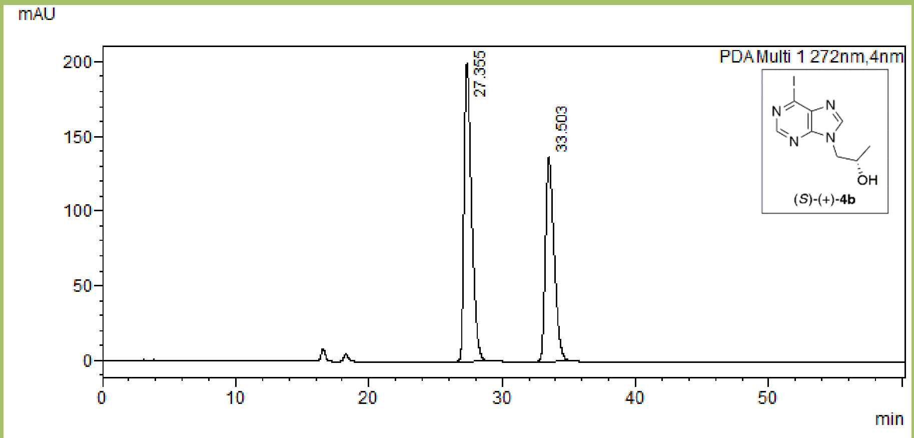

Peak Table

PDA Ch1 272nm

Peak#	Ret. Time	Area	Height	Height%	Area%
1	27.355	7867971	199359	59.338	56.093
2	33.503	6158583	136616	40.662	43.907
Total		14026554	335975	100.000	100.000

HPLC conditions [for (*R*)-(+)-5b]: *n*-hexane-*i*-PrOH (90:10, v/v); f=0.8 mL/min; λ=272 nm; Chiralpak AD-H

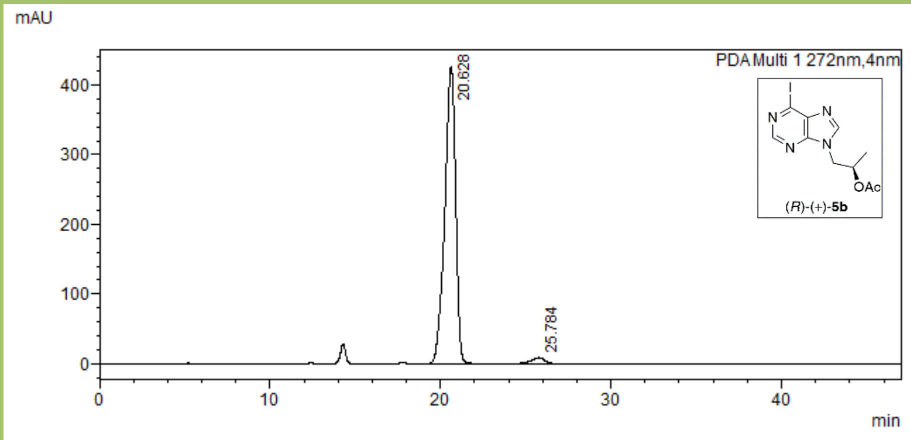

Peak Table

PDA Ch1 272nm

Peak#	Ret. Time	Area	Height	Height%	Area%
1	20.628	18823175	425802	98.056	97.663
2	25.784	450370	8442	1.944	2.337
Total		19273545	434245	100.000	100.000

HPLC analysis for the subsequent biocatalytic reaction:

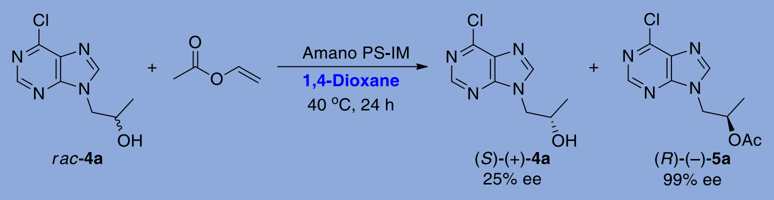

HPLC conditions [for (*S*)-(+)-4a]: *n*-hexane-*i*-PrOH (90:10, v/v); f=1.0 mL/min; λ=264 nm; Chiralcel OJ-H

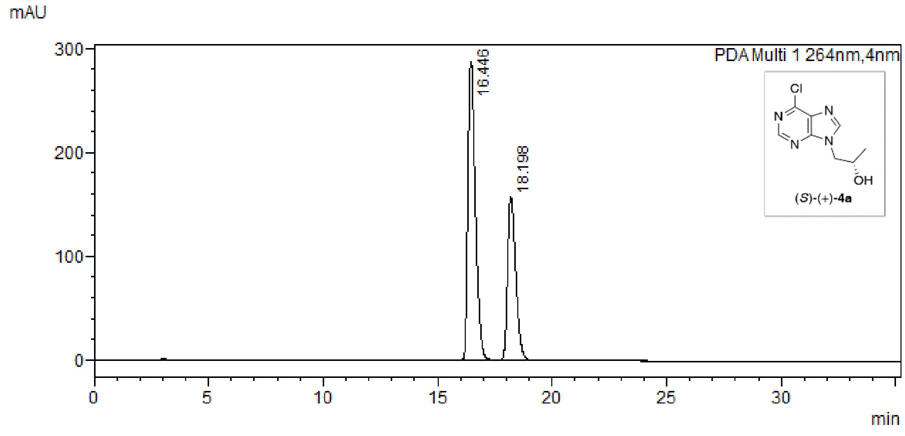

Peak Table

PDA Ch1 264nm

Peak#	Ret. Time	Area	Height	Height%	Area%
1	16.446	6522083	287712	64.533	62.619
2	18.198	3893359	158128	35.467	37.381
Total		10415441	445840	100.000	100.000

HPLC conditions [for (*R*)-(–)-5a]: *n*-hexane-*i*-PrOH (95:5, v/v); f=0.9 mL/min; λ=263 nm; Chiralcel OD-H

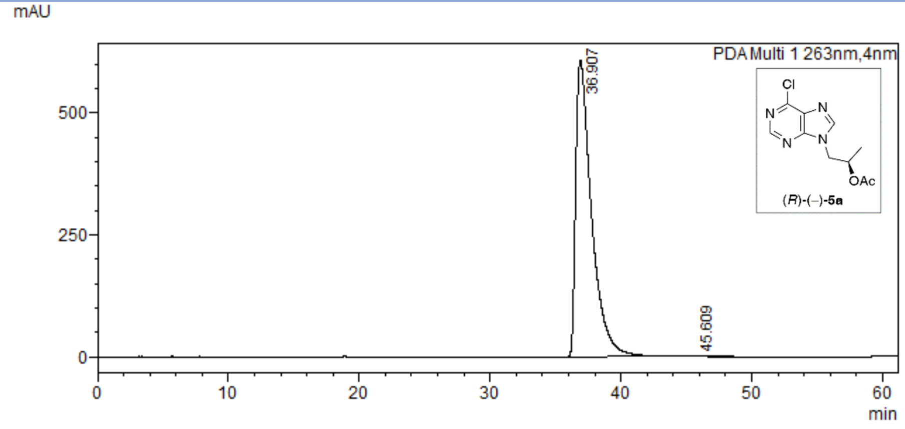

Peak Table

PDA Ch1 263nm

Peak#	Ret. Time	Area	Height	Height%	Area%
1	36.907	50884259	606947	99.666	99.479
2	45.609	266293	2035	0.334	0.521
Total		51150552	608982	100.000	100.000

HPLC analysis for the subsequent biocatalytic reaction:

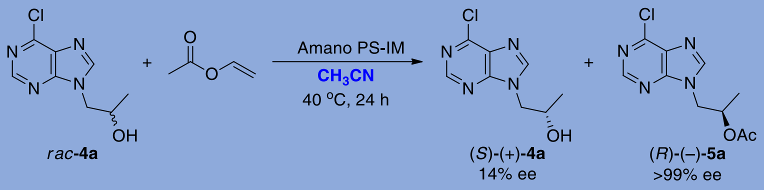

HPLC conditions [for (*S*)-(+)-4a]: *n*-hexane-*i*-PrOH (90:10, v/v); f=1.0 mL/min; λ=264 nm; Chiralcel OJ-H

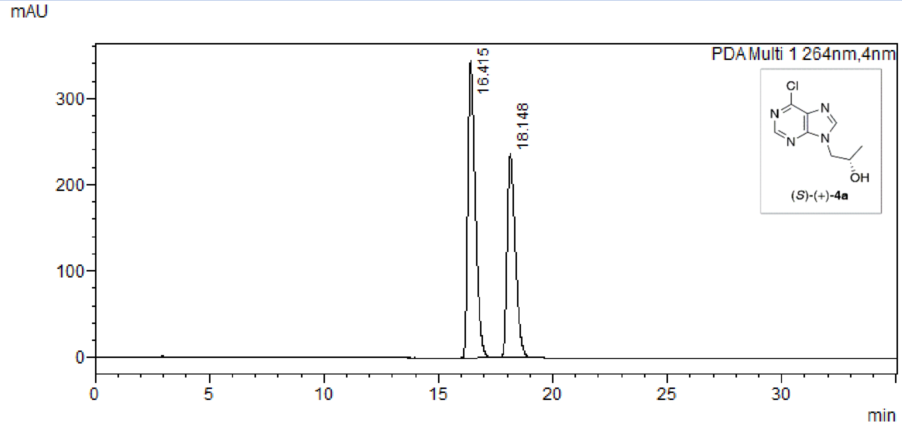

Peak Table

PDA Ch1 264nm

Peak#	Ret. Time	Area	Height	Height%	Area%
1	16.415	7816280	343734	59.254	56.980
2	18.148	5901368	236369	40.746	43.020
Total		13717648	580102	100.000	100.000

HPLC conditions [for (*R*)-(–)-5a]: *n*-hexane-*i*-PrOH (95:5, v/v); f=0.9 mL/min; λ=263 nm; Chiralcel OD-H

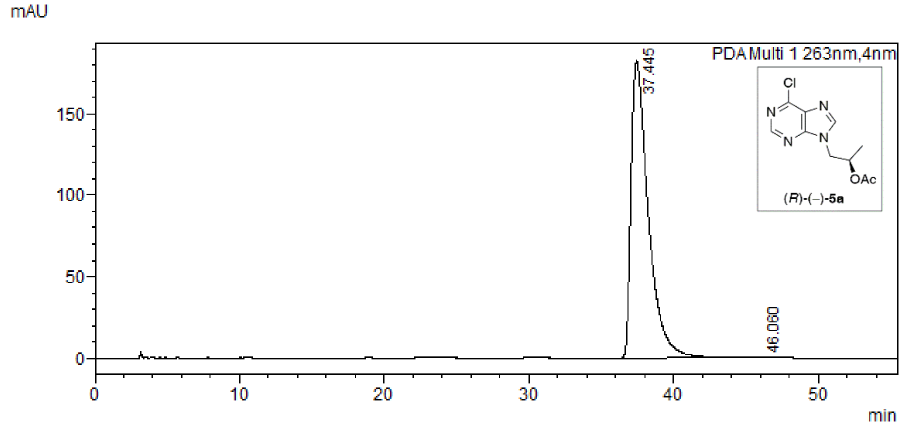

Peak Table

PDA Ch1 263nm

Peak#	Ret. Time	Area	Height	Height%	Area%
1	37.445	15112296	182517	99.809	99.861
2	46.060	21016	349	0.191	0.139
Total		15133313	182866	100.000	100.000

HPLC analysis for the subsequent biocatalytic reaction:

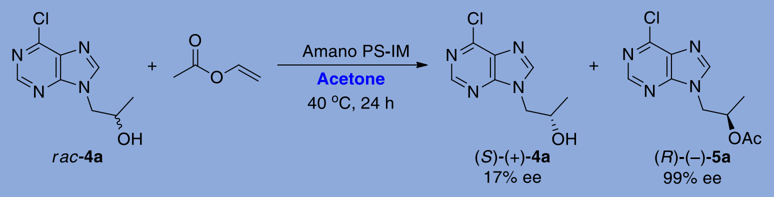

HPLC conditions [for (*S*)-(+)-4a]: *n*-hexane-*i*-PrOH (90:10, v/v); f=1.0 mL/min; λ=264 nm; Chiralcel OJ-H

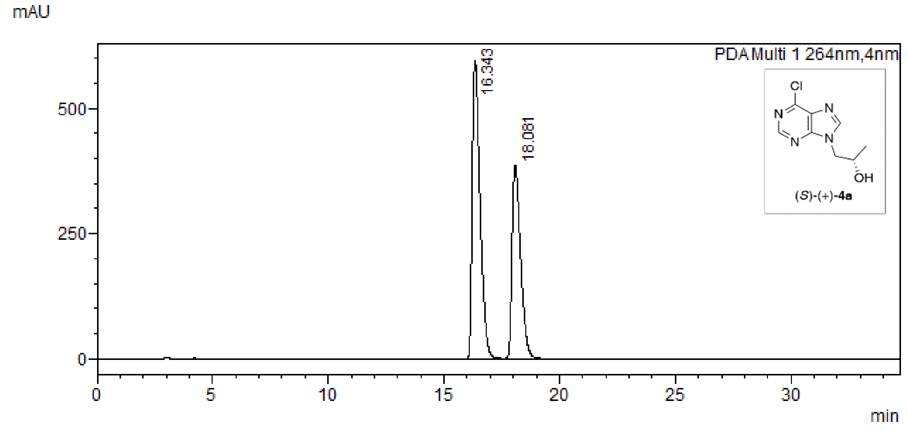

Peak Table

PDA Ch1 264nm

Peak#	Ret. Time	Area	Height	Height%	Area%
1	16.343	13997196	594978	60.687	58.638
2	18.081	9873417	385432	39.313	41.362
Total		23870613	980411	100.000	100.000

HPLC conditions [for (*R*)-(–)-5a]: *n*-hexane-*i*-PrOH (95:5, v/v); f=0.9 mL/min; λ=263 nm; Chiralcel OD-H

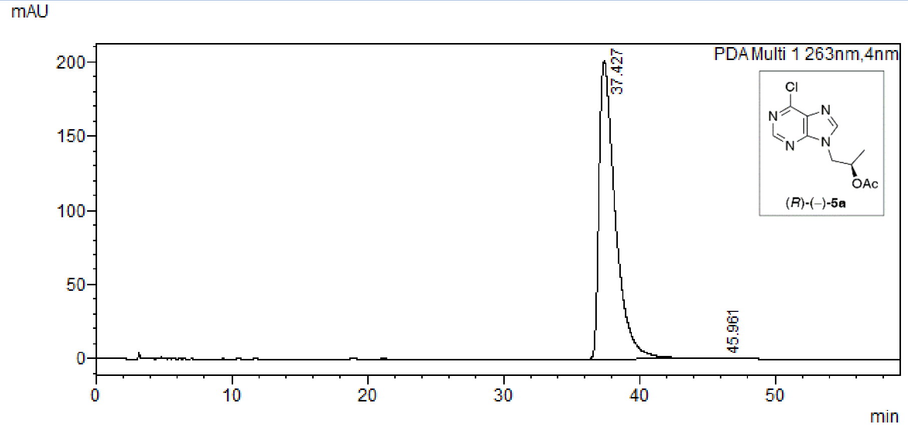

Peak Table

PDA Ch1 263nm

Peak#	Ret. Time	Area	Height	Height%	Area%
1	37.427	16676017	200597	99.669	99.608
2	45.961	65654	666	0.331	0.392
Total		16741671	201264	100.000	100.000

HPLC analysis for the subsequent biocatalytic reaction:

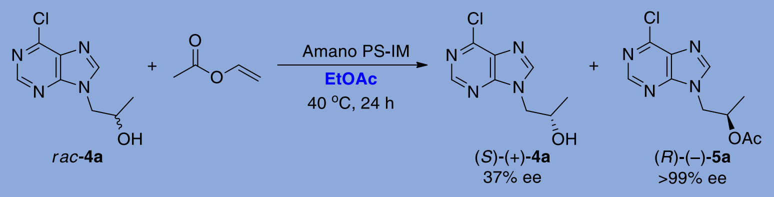

HPLC conditions [for (*S*)-(+)-4a]: *n*-hexane-*i*-PrOH (90:10, v/v); f=1.0 mL/min; λ=264 nm; Chiralcel OJ-H

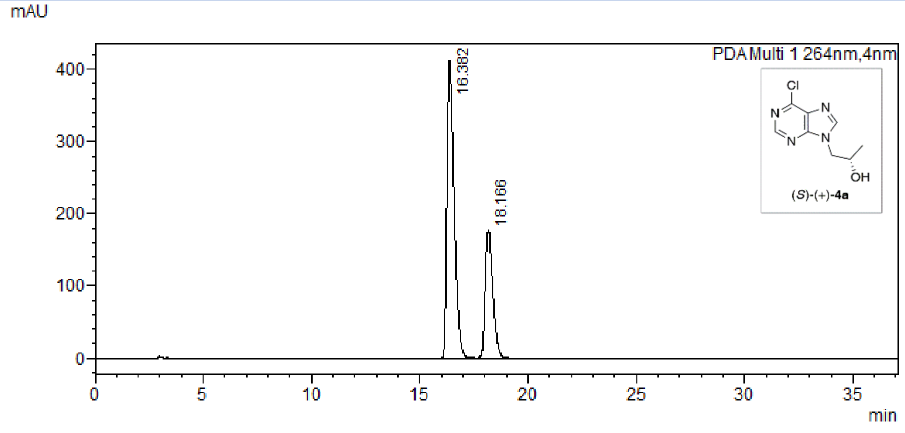

Peak Table

PDA Ch1 264nm

Peak#	Ret. Time	Area	Height	Height%	Area%
1	16.382	9471489	411846	69.929	68.488
2	18.166	4358015	177106	30.071	31.512
Total		13829504	588952	100.000	100.000

HPLC conditions [for (*R*)-(–)-5a]: *n*-hexane-*i*-PrOH (95:5, v/v); f=0.9 mL/min; λ=263 nm; Chiralcel OD-H

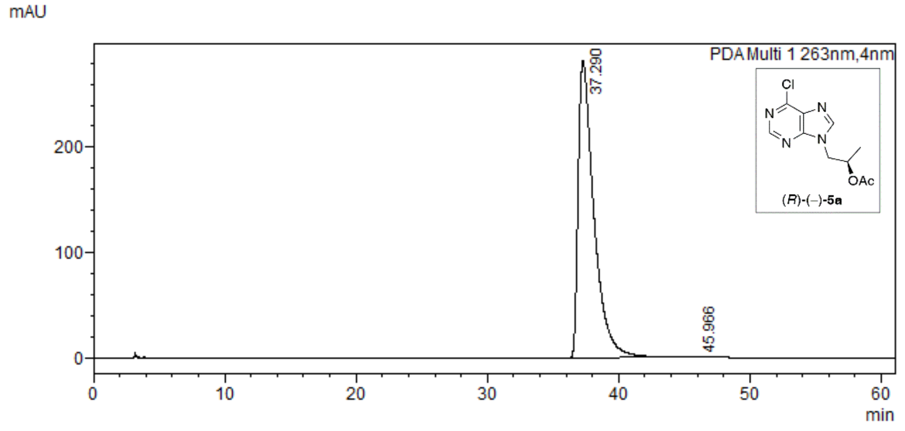

Peak Table

PDA Ch1 263nm

Peak#	Ret. Time	Area	Height	Height%	Area%
1	37.290	23515195	281808	99.802	99.776
2	45.966	52846	560	0.198	0.224
Total		23568041	282368	100.000	100.000

HPLC analysis for the subsequent biocatalytic reaction:

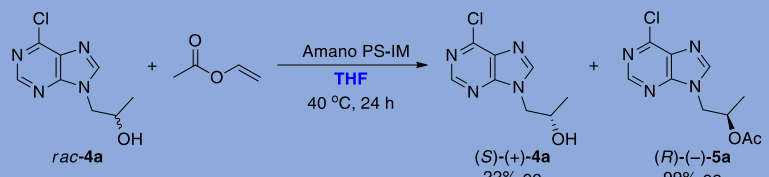

HPLC conditions [for (*S*)-(+)-4a]: *n*-hexane-*i*-PrOH (90:10, v/v); f=1.0 mL/min; λ=264 nm; Chiralcel OJ-H

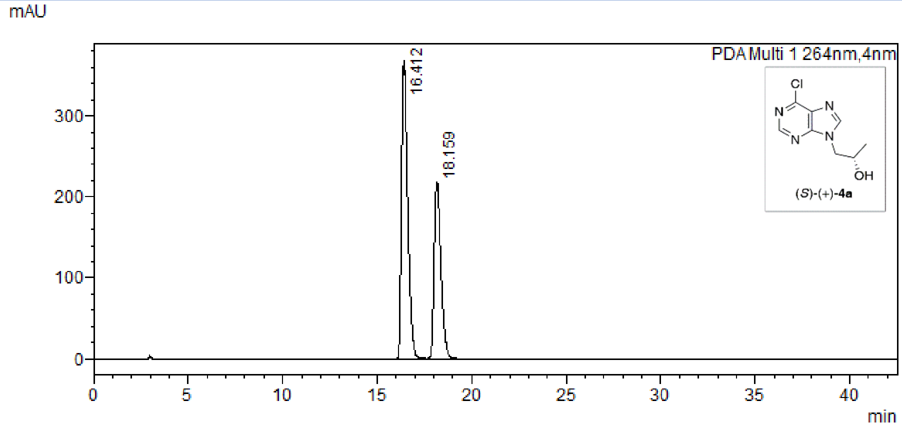

Peak Table

PDA Ch1 264nm

Peak#	Ret. Time	Area	Height	Height%	Area%
1	16.412	8392529	368118	62.744	60.762
2	18.159	5419550	218576	37.256	39.238
Total		13812079	586694	100.000	100.000

HPLC conditions [for (*R*)-(–)-5a]: *n*-hexane-*i*-PrOH (95:5, v/v); f=0.9 mL/min; λ=263 nm; Chiralcel OD-H

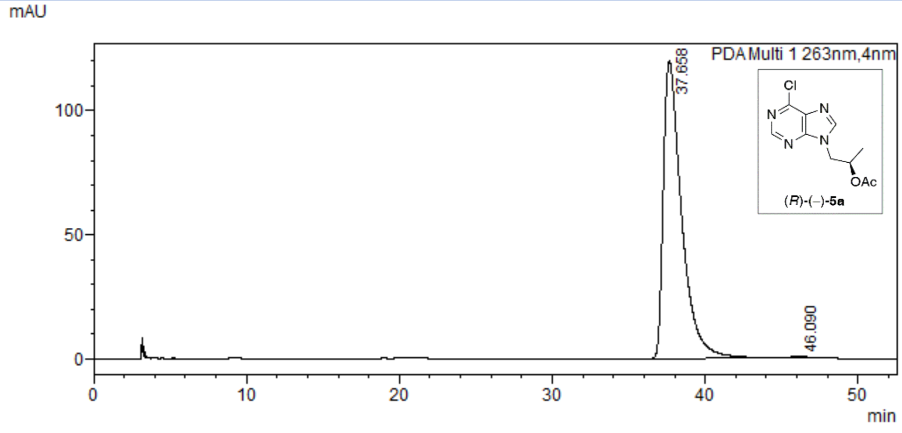

Peak Table

PDA Ch1 263nm

Peak#	Ret. Time	Area	Height	Height%	Area%
1	37.658	9963467	120080	99.537	99.526
2	46.090	47453	559	0.463	0.474
Total		10010920	120639	100.000	100.000

HPLC analysis for the subsequent biocatalytic reaction:

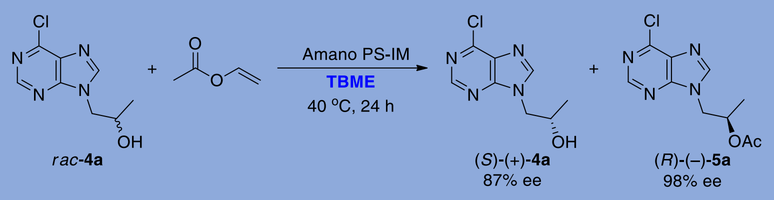

HPLC conditions [for (*S*)-(+)-4a]: *n*-hexane-*i*-PrOH (90:10, v/v); f=1.0 mL/min; λ=264 nm; Chiralcel OJ-H

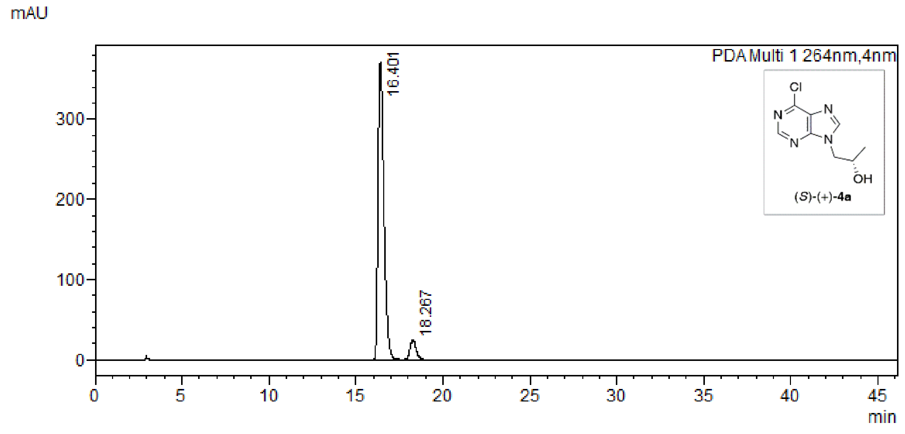

Peak Table

PDA Ch1 264nm

Peak#	Ret. Time	Area	Height	Height%	Area%
1	16.401	8485721	370858	93.776	93.524
2	18.267	587573	24616	6.224	6.476
Total		9073294	395473	100.000	100.000

HPLC conditions [for (*R*)-(–)-5a]: *n*-hexane-*i*-PrOH (95:5, v/v); f=0.9 mL/min; λ=263 nm; Chiralcel OD-H

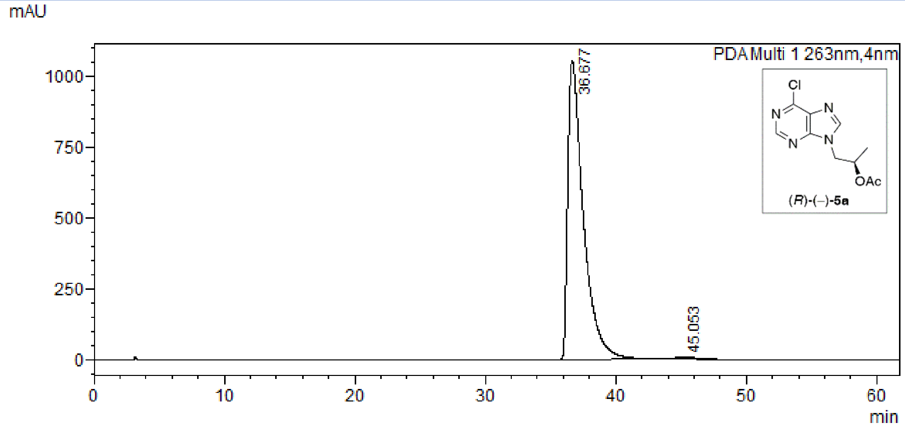

Peak Table

PDA Ch1 263nm

Peak#	Ret. Time	Area	Height	Height%	Area%
1	36.677	87847592	1054250	99.196	99.023
2	45.053	867154	8546	0.804	0.977
Total		88714746	1062796	100.000	100.000

HPLC analysis for the subsequent biocatalytic reaction:

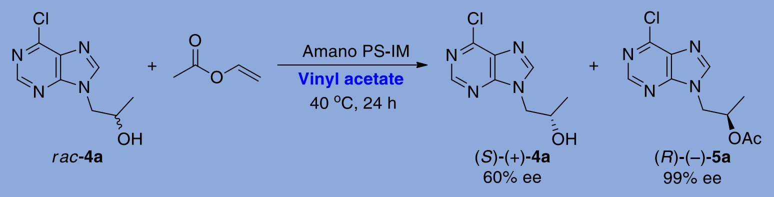

HPLC conditions [for (*S*)-(+)-4a]: *n*-hexane-*i*-PrOH (90:10, v/v); f=1.0 mL/min; λ=264 nm; Chiralcel OJ-H

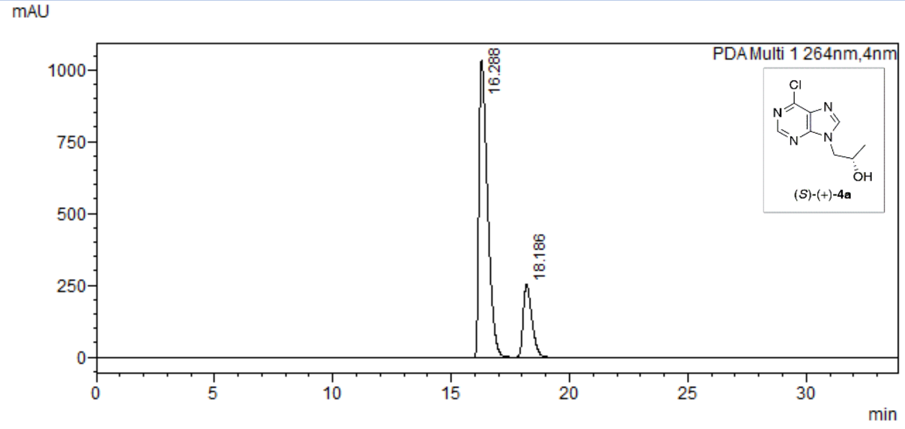

Peak Table

PDA Ch1 264nm

Peak#	Ret. Time	Area	Height	Height%	Area%
1	16.288	26281120	1032380	80.396	80.188
2	18.186	6493200	251734	19.604	19.812
Total		32774320	1284114	100.000	100.000

HPLC conditions [for (*R*)-(–)-5a]: *n*-hexane-*i*-PrOH (95:5, v/v); f=0.9 mL/min; λ=263 nm; Chiralcel OD-H

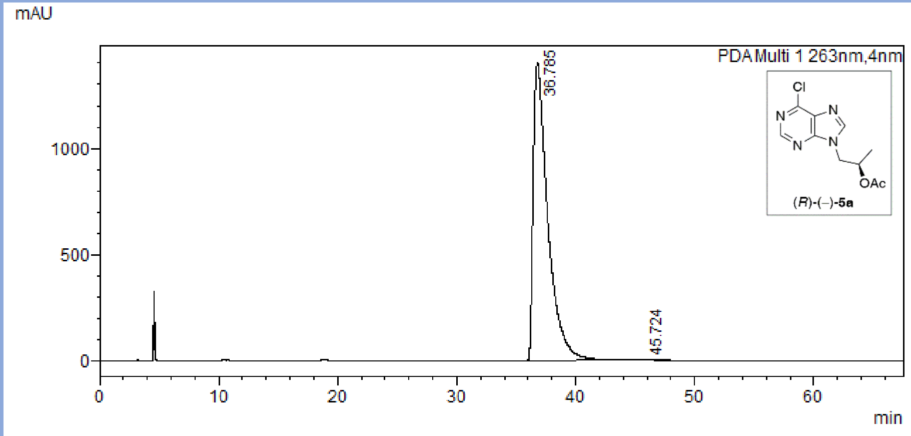

Peak Table

PDA Ch1 263nm

Peak#	Ret. Time	Area	Height	Height%	Area%
1	36.785	120240464	1401577	99.762	99.732
2	45.724	323058	3343	0.238	0.268
Total		120563521	1404920	100.000	100.000

HPLC analysis for the subsequent biocatalytic reaction:

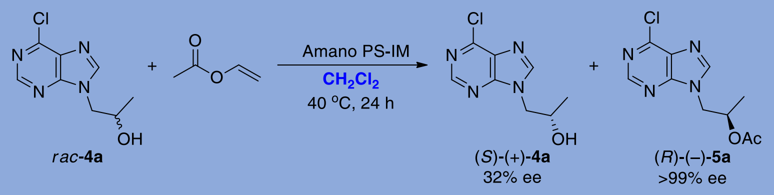

HPLC conditions [for (*S*)-(+)-4a]: *n*-hexane-*i*-PrOH (90:10, v/v); f=1.0 mL/min; λ=264 nm; Chiralcel OJ-H

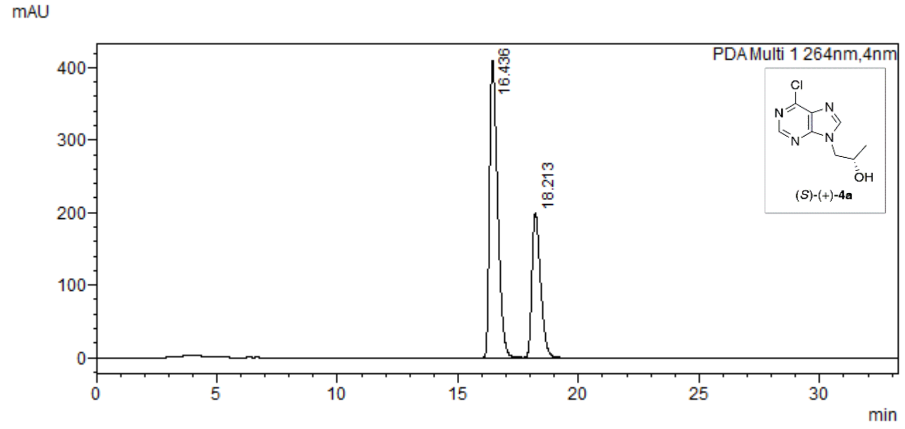

Peak Table

PDA Ch1 264nm

Peak#	Ret. Time	Area	Height	Height%	Area%
1	16.436	9506199	409275	67.233	65.786
2	18.213	4943990	199466	32.767	34.214
Total		14450189	608741	100.000	100.000

HPLC conditions [for (*R*)-(–)-5a]: *n*-hexane-*i*-PrOH (95:5, v/v); f=0.9 mL/min; λ=263 nm; Chiralcel OD-H

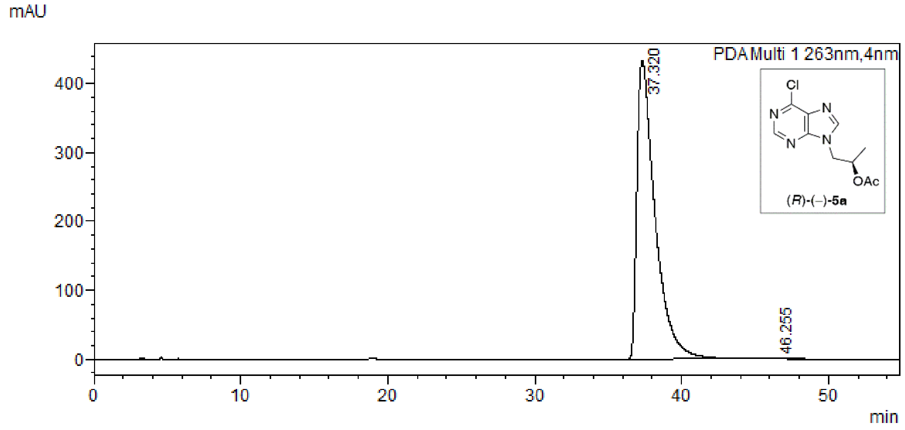

Peak Table

PDA Ch1 263nm

Peak#	Ret. Time	Area	Height	Height%	Area%
1	37.320	36931745	432470	99.798	99.816
2	46.255	67992	873	0.202	0.184
Total		36999738	433344	100.000	100.000

HPLC analysis for the subsequent biocatalytic reaction:

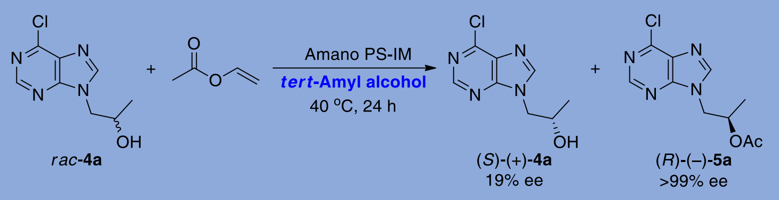

HPLC conditions [for (*S*)-(+)-4a]: *n*-hexane-*i*-PrOH (90:10, v/v); f=1.0 mL/min; λ=264 nm; Chiralcel OJ-H

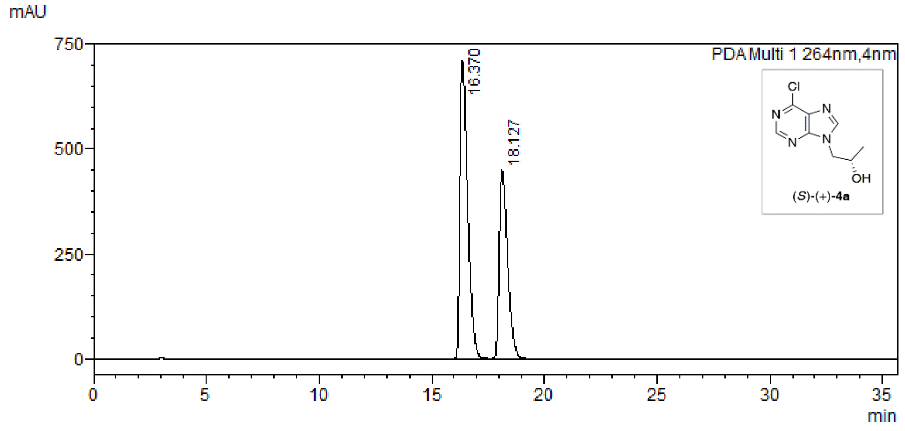

Peak Table

PDA Ch1 264nm

Peak#	Ret. Time	Area	Height	Height%	Area%
1	16.370	17142063	710331	61.213	59.414
2	18.127	11709751	450102	38.787	40.586
Total		28851814	1160433	100.000	100.000

HPLC conditions [for (*R*)-(–)-5a]: *n*-hexane-*i*-PrOH (95:5, v/v); f=0.9 mL/min; λ=263 nm; Chiralcel OD-H

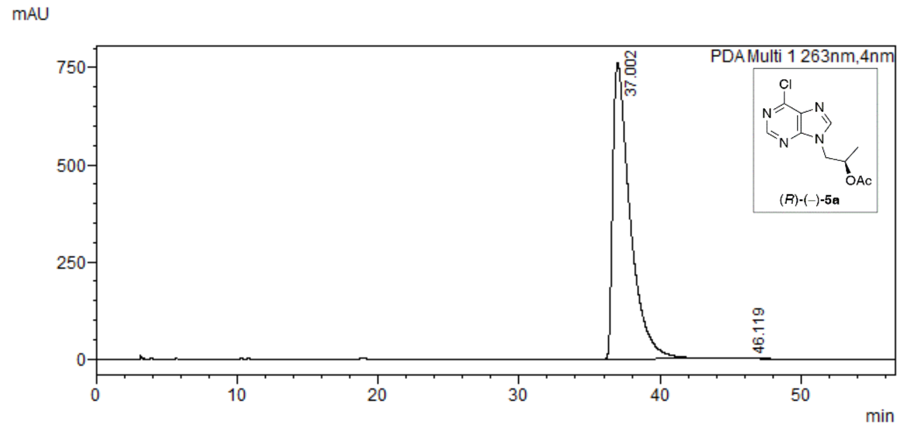

Peak Table

PDA Ch1 263nm

Peak#	Ret. Time	Area	Height	Height%	Area%
1	37.002	65238169	761145	99.818	99.826
2	46.119	113547	1386	0.182	0.174
Total		65351716	762531	100.000	100.000

HPLC analysis for the subsequent biocatalytic reaction:

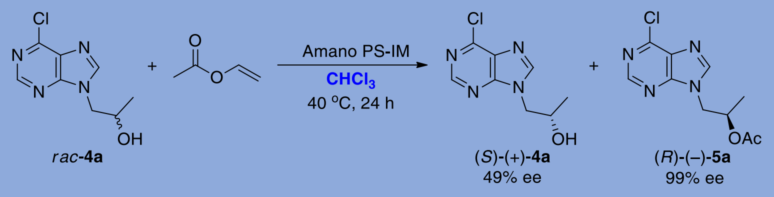

HPLC conditions [for (*S*)-(+)-4a]: *n*-hexane-*i*-PrOH (90:10, v/v); f=1.0 mL/min; λ=264 nm; Chiralcel OJ-H

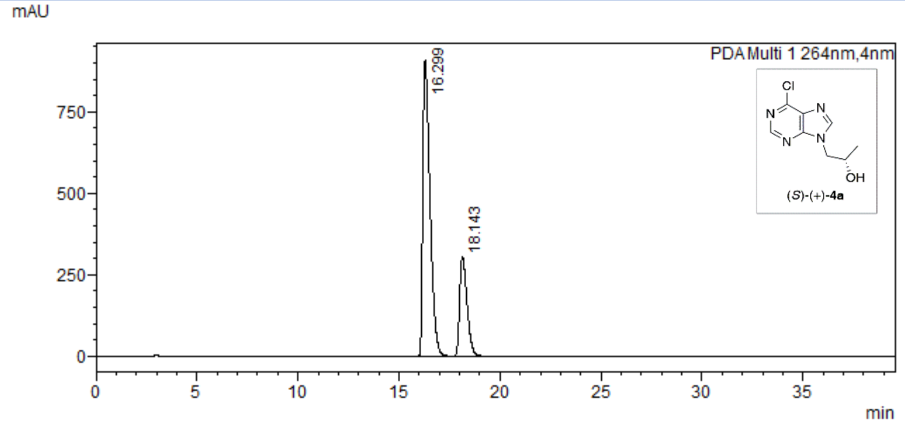

Peak Table

PDA Ch1 264nm

Peak#	Ret. Time	Area	Height	Height%	Area%
1	16.299	22422394	907423	74.855	74.307
2	18.143	7752787	304815	25.145	25.693
Total		30175181	1212237	100.000	100.000

HPLC conditions [for (*R*)-(–)-5a]: *n*-hexane-*i*-PrOH (95:5, v/v); f=0.9 mL/min; λ=263 nm; Chiralcel OD-H

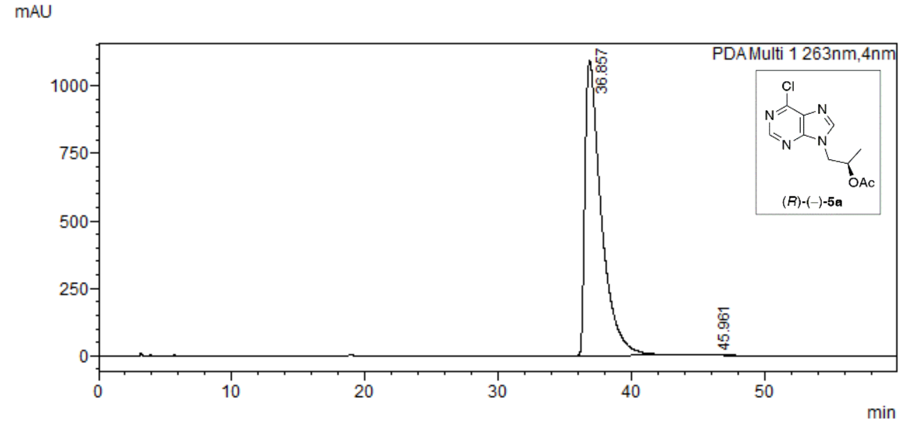

Peak Table

PDA Ch1 263nm

Peak#	Ret. Time	Area	Height	Height%	Area%
1	36.857	93485379	1093236	99.759	99.712
2	45.961	270163	2642	0.241	0.288
Total		93755542	1095878	100.000	100.000

HPLC analysis for the subsequent biocatalytic reaction:

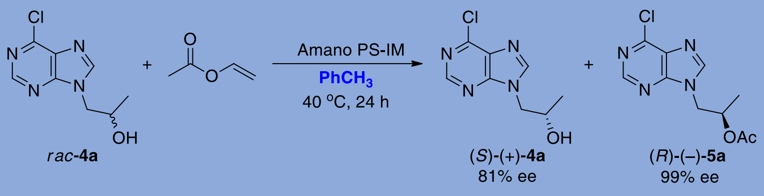

HPLC conditions [for (*S*)-(+)-4a]: *n*-hexane-*i*-PrOH (90:10, v/v); f=1.0 mL/min; λ=264 nm; Chiralcel OJ-H

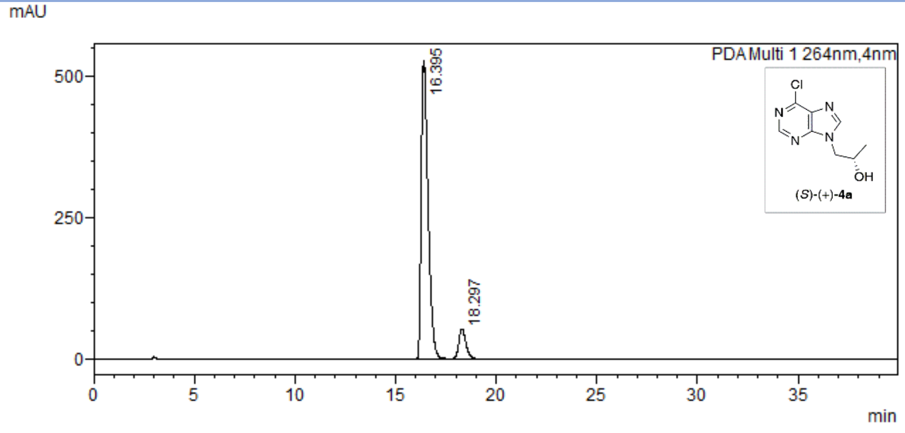

Peak Table

PDA Ch1 264nm

Peak#	Ret. Time	Area	Height	Height%	Area%
1	16.395	12437752	527679	90.869	90.641
2	18.297	1284185	53024	9.131	9.359
Total		13721937	580703	100.000	100.000

HPLC conditions [for (*R*)-(–)-5a]: *n*-hexane-*i*-PrOH (95:5, v/v); f=0.9 mL/min; λ=263 nm; Chiralcel OD-H

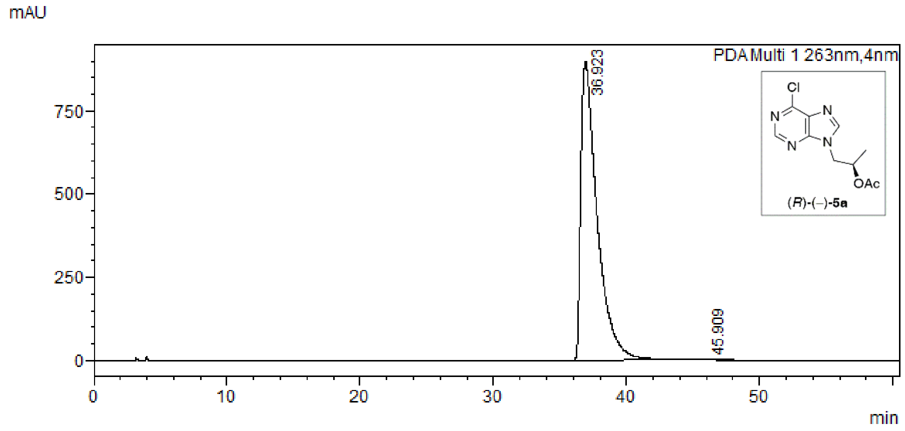

Peak Table

PDA Ch1 263nm

Peak#	Ret. Time	Area	Height	Height%	Area%
1	36.923	76983178	897137	99.596	99.541
2	45.909	354768	3643	0.404	0.459
Total		77337946	900780	100.000	100.000

HPLC analysis for the subsequent biocatalytic reaction:

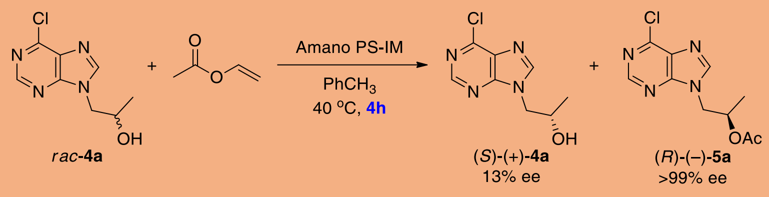

HPLC conditions [for (*S*)-(+)-4a]: *n*-hexane-*i*-PrOH (90:10, v/v); f=1.0 mL/min; λ=264 nm; Chiralcel OJ-H

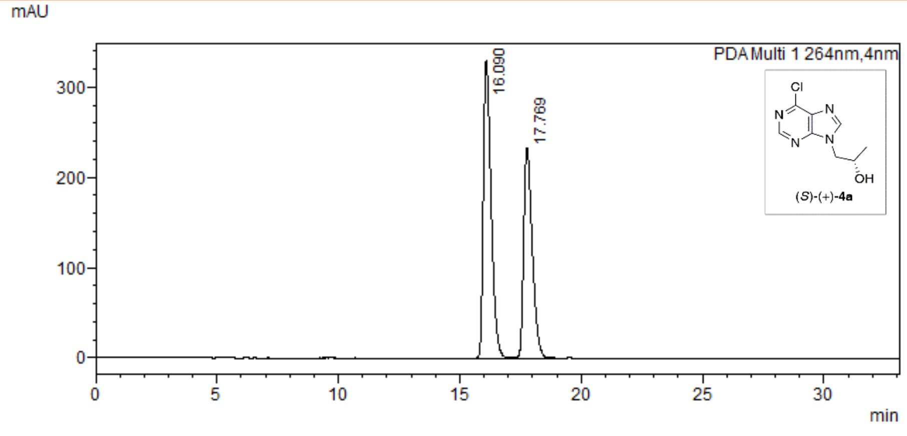

Peak Table

PDA Ch1 264nm

Peak#	Ret. Time	Area	Height	Height%	Area%
1	16.090	7387281	329415	58.637	56.348
2	17.769	5722770	232372	41.363	43.652
Total		13110051	561786	100.000	100.000

HPLC conditions [for (*R*)-(–)-5a]: *n*-hexane-*i*-PrOH (95:5, v/v); f=0.9 mL/min; λ=263 nm; Chiralcel OD-H

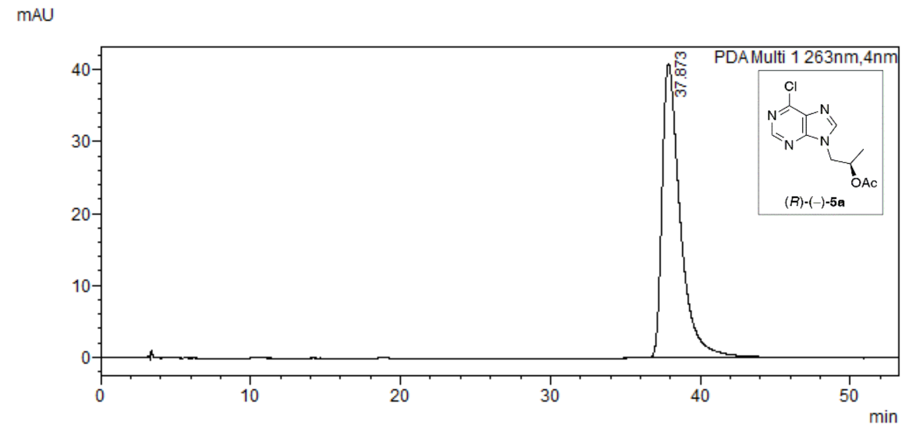

Peak Table

PDA Ch1 263nm

Peak#	Ret. Time	Area	Height	Height%	Area%
1	37.873	3435464	40834	100.000	100.000
Total		3435464	40834	100.000	100.000

HPLC analysis for the subsequent biocatalytic reaction:

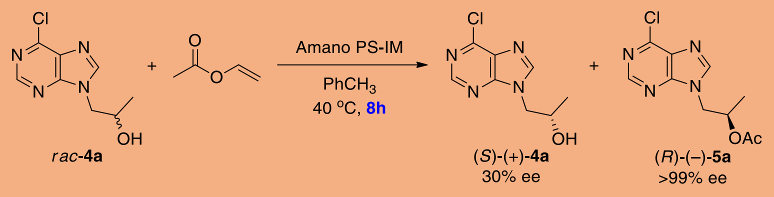

HPLC conditions [for (*S*)-(+)-4a]: *n*-hexane-*i*-PrOH (90:10, v/v); f=1.0 mL/min; λ=264 nm; Chiralcel OJ-H

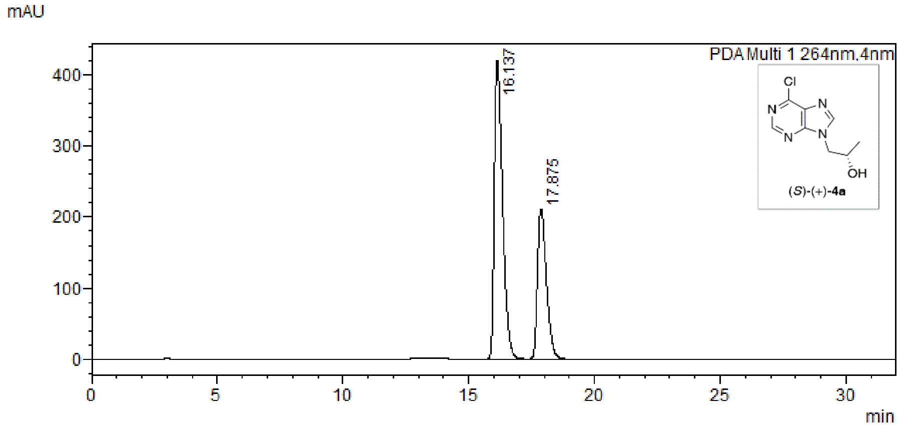

Peak Table

PDA Ch1 264nm

Peak#	Ret. Time	Area	Height	Height%	Area%
1	16.137	9602256	419505	66.494	64.784
2	17.875	5219805	211390	33.506	35.216
Total		14822061	630895	100.000	100.000

HPLC conditions [for (*R*)-(–)-5a]: *n*-hexane-*i*-PrOH (95:5, v/v); f=0.9 mL/min; λ=263 nm; Chiralcel OD-H

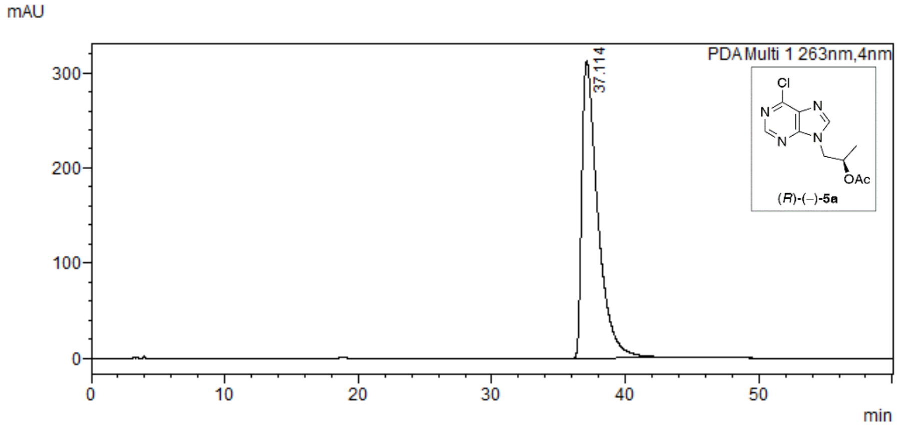

Peak Table

PDA Ch1 263nm

Peak#	Ret. Time	Area	Height	Height%	Area%
1	37.114	25904261	312163	100.000	100.000
Total		25904261	312163	100.000	100.000

HPLC analysis for the subsequent biocatalytic reaction:

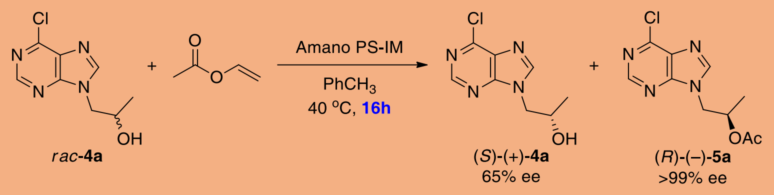

HPLC conditions [for (*S*)-(+)-4a]: *n*-hexane-*i*-PrOH (90:10, v/v); f=1.0 mL/min; λ=264 nm; Chiralcel OJ-H

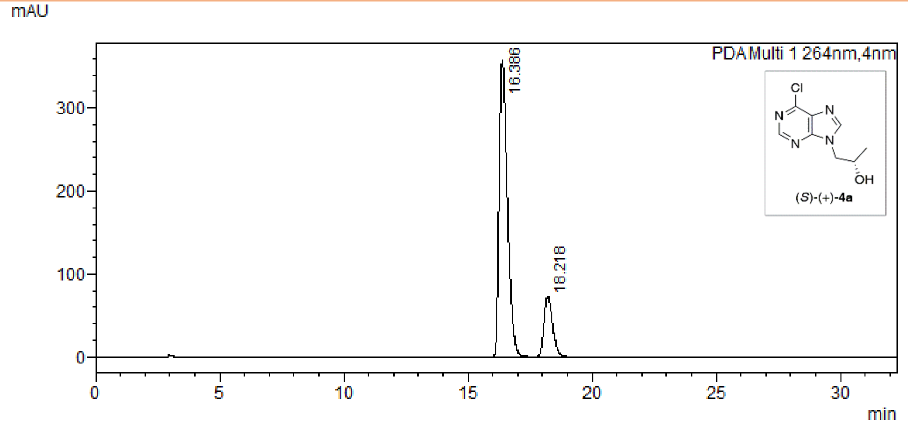

Peak Table

PDA Ch1 264nm

Peak#	Ret. Time	Area	Height	Height%	Area%
1	16.386	8203915	357635	83.054	82.299
2	18.218	1764466	72969	16.946	17.701
Total		9968381	430603	100.000	100.000

HPLC conditions [for (*R*)-(–)-5a]: *n*-hexane-*i*-PrOH (95:5, v/v); f=0.9 mL/min; λ=263 nm; Chiralcel OD-H

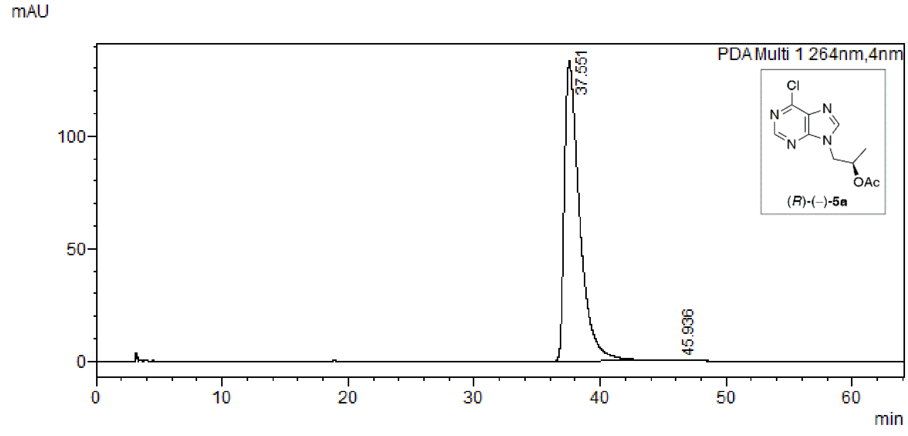

Peak Table

PDA Ch1 264nm

Peak#	Ret. Time	Area	Height	Height%	Area%
1	37.551	11165114	133288	99.763	99.784
2	45.936	24159	316	0.237	0.216
Total		11189273	133604	100.000	100.000

HPLC analysis for the subsequent biocatalytic reaction:

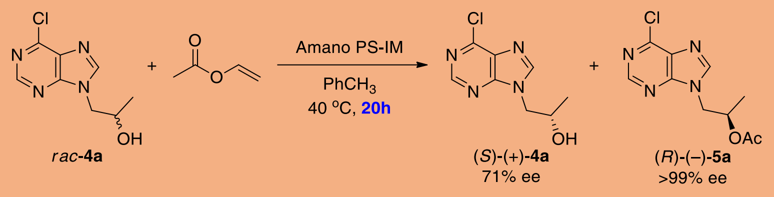

HPLC conditions [for (*S*)-(+)-4a]: *n*-hexane-*i*-PrOH (90:10, v/v); f=1.0 mL/min; λ=264 nm; Chiralcel OJ-H

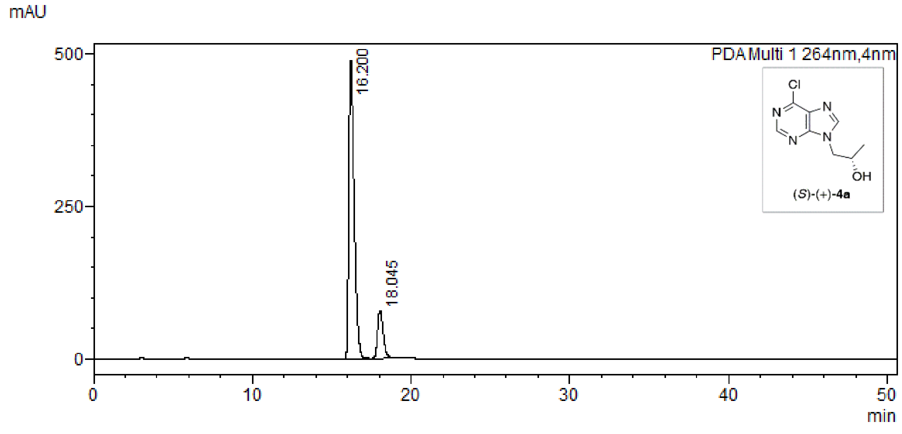

Peak Table

PDA Ch1 264nm

Peak#	Ret. Time	Area	Height	Height%	Area%
1	16.200	11381646	488309	86.167	85.649
2	18.045	1907089	78392	13.833	14.351
Total		13288735	566700	100.000	100.000

HPLC conditions [for (*R*)-(–)-5a]: *n*-hexane-*i*-PrOH (95:5, v/v); f=0.9 mL/min; λ=263 nm; Chiralcel OD-H

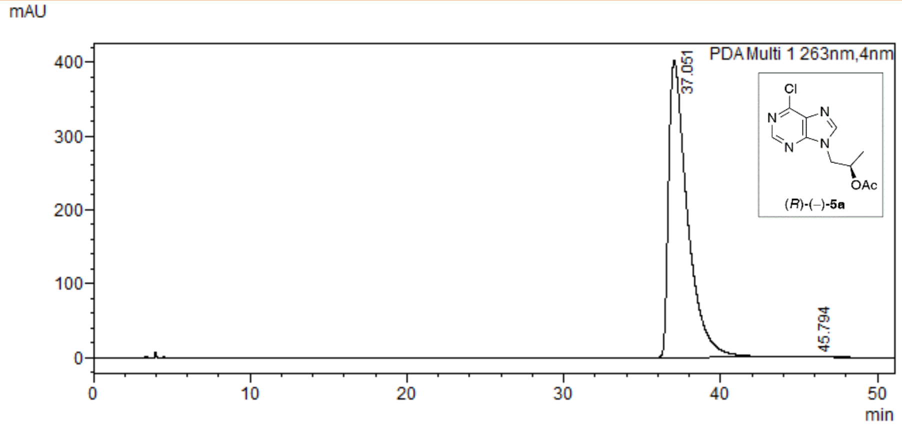

Peak Table

PDA Ch1 263nm

Peak#	Ret. Time	Area	Height	Height%	Area%
1	37.051	33475595	401552	99.831	99.910
2	45.794	30259	679	0.169	0.090
Total		33505854	402230	100.000	100.000

HPLC analysis for the subsequent biocatalytic reaction:

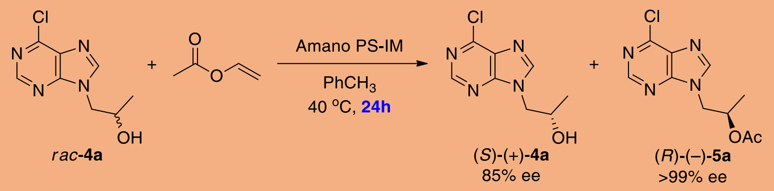

HPLC conditions [for (*S*)-(+)-4a]: *n*-hexane-*i*-PrOH (90:10, v/v); f=1.0 mL/min; λ=264 nm; Chiralcel OJ-H

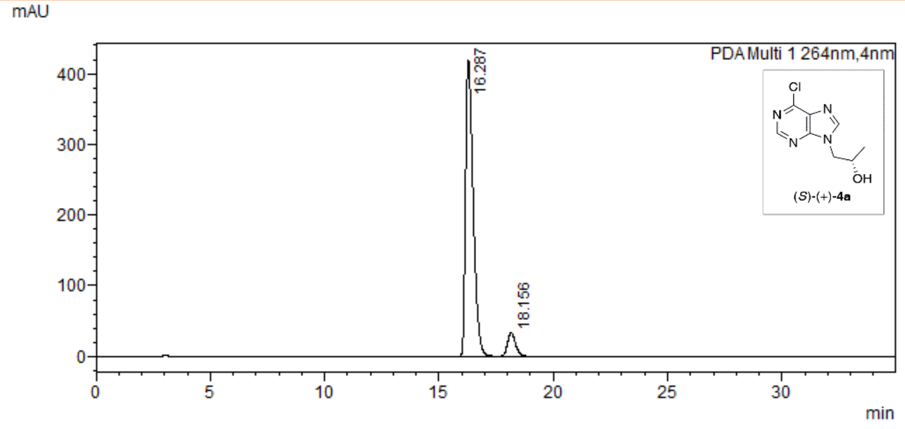

Peak Table

PDA Ch1 264nm

Peak#	Ret. Time	Area	Height	Height%	Area%
1	16.287	9705225	418725	92.552	92.270
2	18.156	813058	33698	7.448	7.730
Total		10518283	452423	100.000	100.000

HPLC conditions [for (*R*)-(–)-5a]: *n*-hexane-*i*-PrOH (95:5, v/v); f=0.9 mL/min; λ=263 nm; Chiralcel OD-H

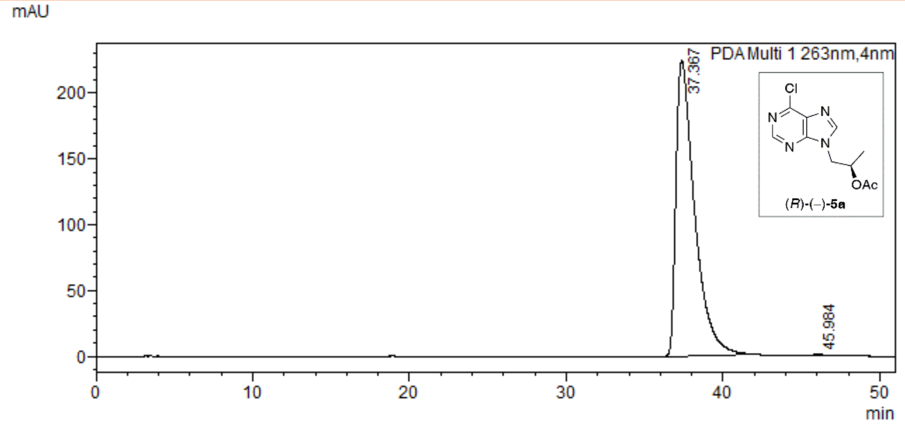

Peak Table

PDA Ch1 263nm

Peak#	Ret. Time	Area	Height	Height%	Area%
1	37.367	18640554	224735	99.739	99.804
2	45.984	36621	587	0.261	0.196
Total		18677176	225322	100.000	100.000

HPLC analysis for the subsequent biocatalytic reaction:

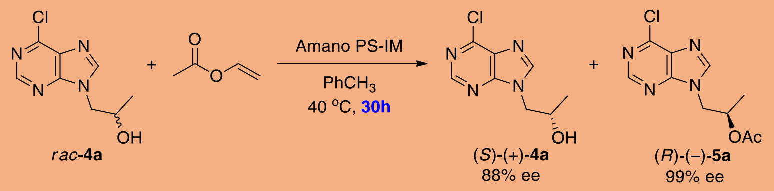

HPLC conditions [for (*S*)-(+)-4a]: *n*-hexane-*i*-PrOH (90:10, v/v); f=1.0 mL/min; λ=264 nm; Chiralcel OJ-H

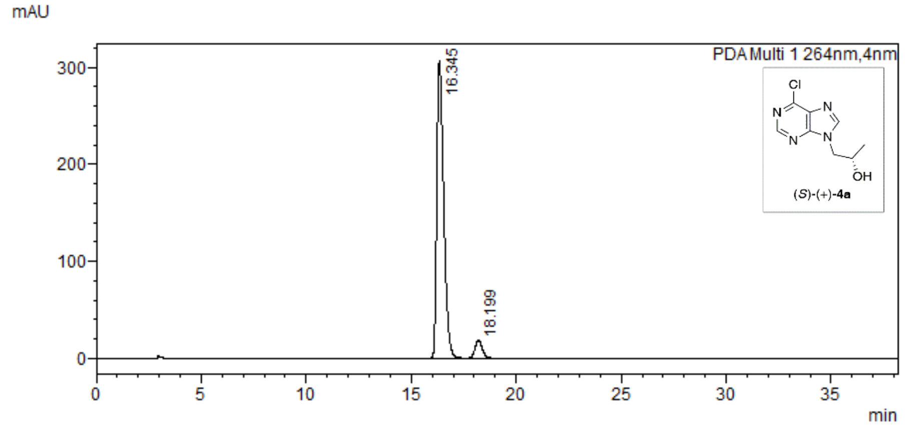

Peak Table

PDA Ch1 264nm

Peak#	Ret. Time	Area	Height	Height%	Area%
1	16.345	7011193	306610	94.297	94.028
2	18.199	445326	18542	5.703	5.972
Total		7456518	325152	100.000	100.000

HPLC conditions [for (*R*)-(–)-5a]: *n*-hexane-*i*-PrOH (95:5, v/v); f=0.9 mL/min; λ=263 nm; Chiralcel OD-H

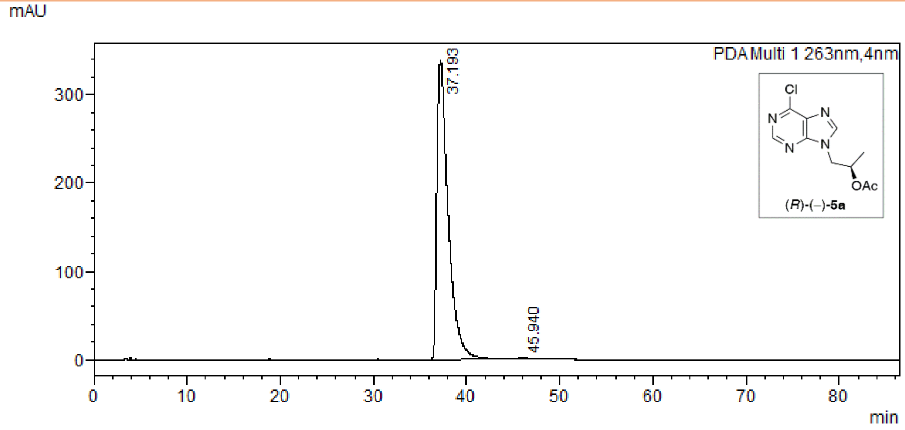

Peak Table

PDA Ch1 263nm

Peak#	Ret. Time	Area	Height	Height%	Area%
1	37.193	28323661	338531	99.549	99.481
2	45.940	147777	1535	0.451	0.519
Total		28471439	340066	100.000	100.000

HPLC analysis for the subsequent biocatalytic reaction:

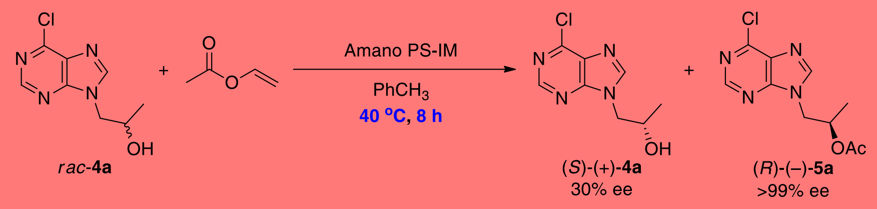

HPLC conditions [for (*S*)-(+)-4a]: *n*-hexane-*i*-PrOH (90:10, v/v); f=1.0 mL/min; λ=264 nm; Chiralcel OJ-H

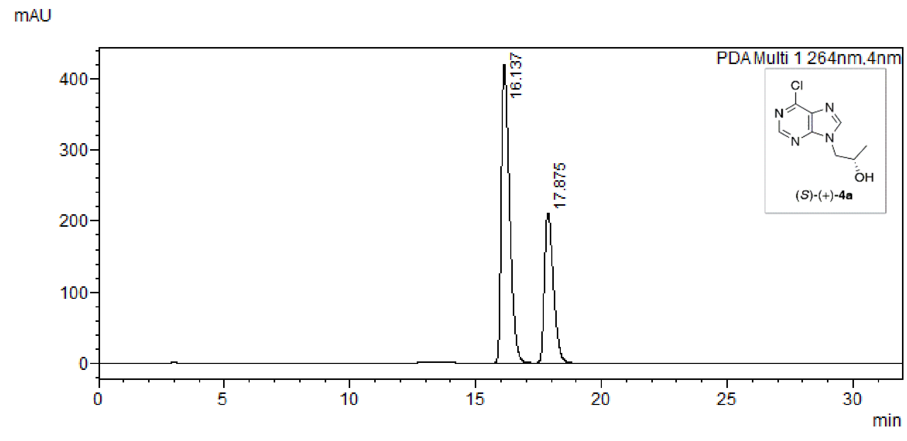

Peak Table

PDA Ch1 264nm

Peak#	Ret. Time	Area	Height	Height%	Area%
1	16.137	9602256	419505	66.494	64.784
2	17.875	5219805	211390	33.506	35.216
Total		14822061	630895	100.000	100.000

HPLC conditions [for (*R*)-(–)-5a]: *n*-hexane-*i*-PrOH (95:5, v/v); f=0.9 mL/min; λ=263 nm; Chiralcel OD-H

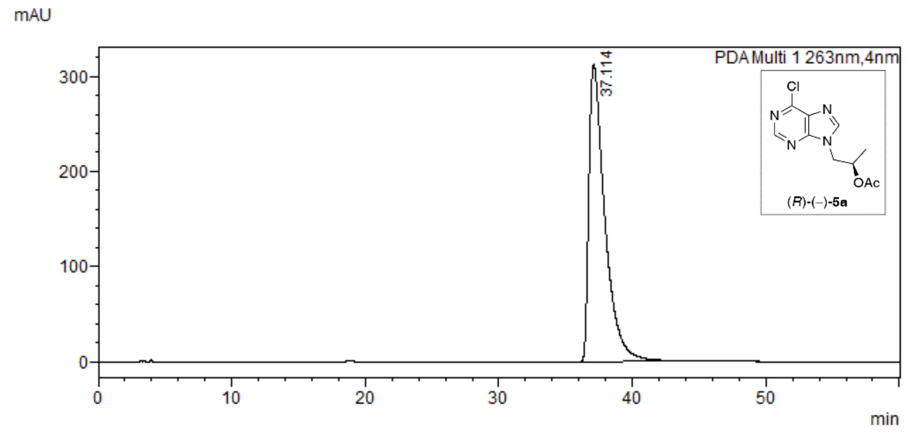

Peak Table

PDA Ch1 263nm

Peak#	Ret. Time	Area	Height	Height%	Area%
1	37.114	25904261	312163	100.000	100.000
Total		25904261	312163	100.000	100.000

HPLC analysis for the subsequent biocatalytic reaction:

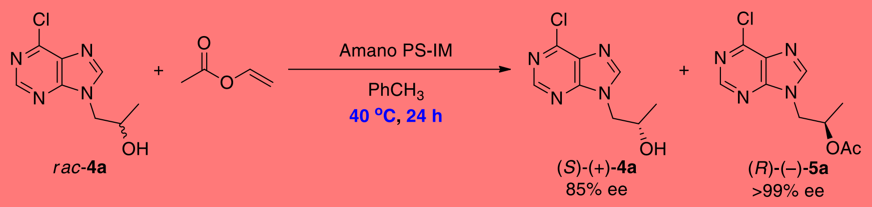

HPLC conditions [for (*S*)-(+)-4a]: *n*-hexane-*i*-PrOH (90:10, v/v); f=1.0 mL/min; λ=264 nm; Chiralcel OJ-H

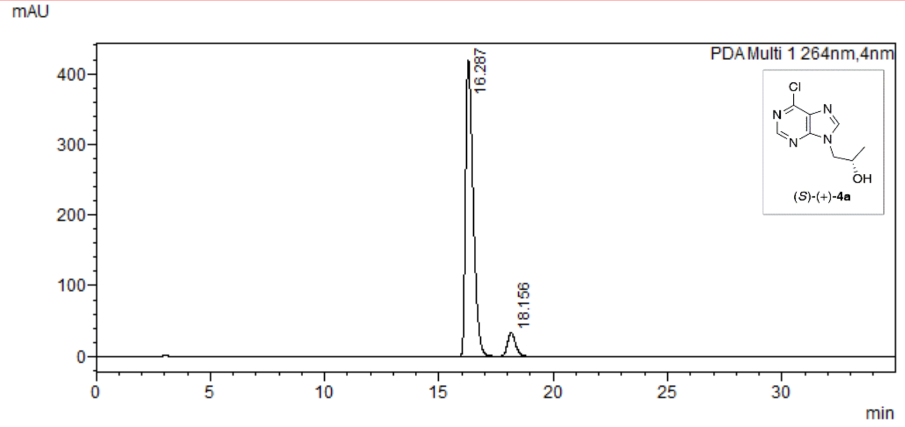

Peak Table

PDA Ch1 264nm

Peak#	Ret. Time	Area	Height	Height%	Area%
1	16.287	9705225	418725	92.552	92.270
2	18.156	813058	33698	7.448	7.730
Total		10518283	452423	100.000	100.000

HPLC conditions [for (*R*)-(–)-5a]: *n*-hexane-*i*-PrOH (95:5, v/v); f=0.9 mL/min; λ=263 nm; Chiralcel OD-H

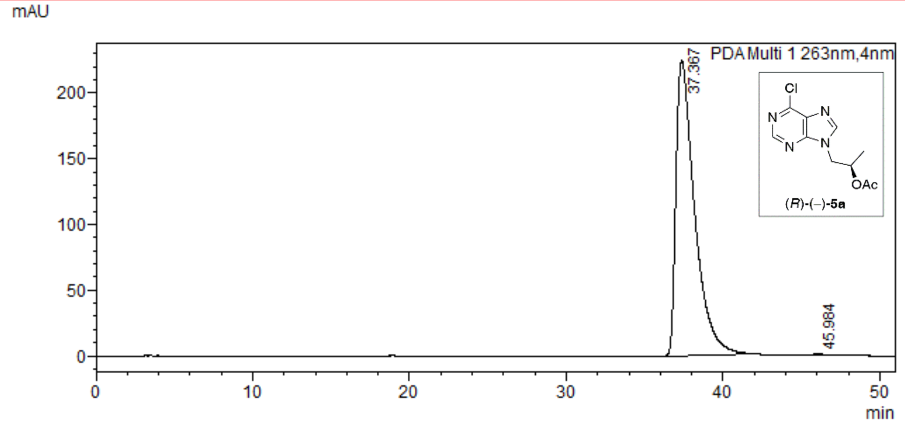

Peak Table

PDA Ch1 263nm

Peak#	Ret. Time	Area	Height	Height%	Area%
1	37.367	18640554	224735	99.739	99.804
2	45.984	36621	587	0.261	0.196
Total		18677176	225322	100.000	100.000

HPLC analysis for the subsequent biocatalytic reaction:

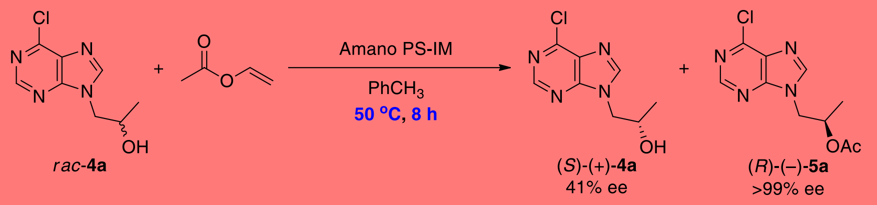

HPLC conditions [for (*S*)-(+)-4a]: *n*-hexane-*i*-PrOH (90:10, v/v); f=1.0 mL/min; λ=264 nm; Chiralcel OJ-H

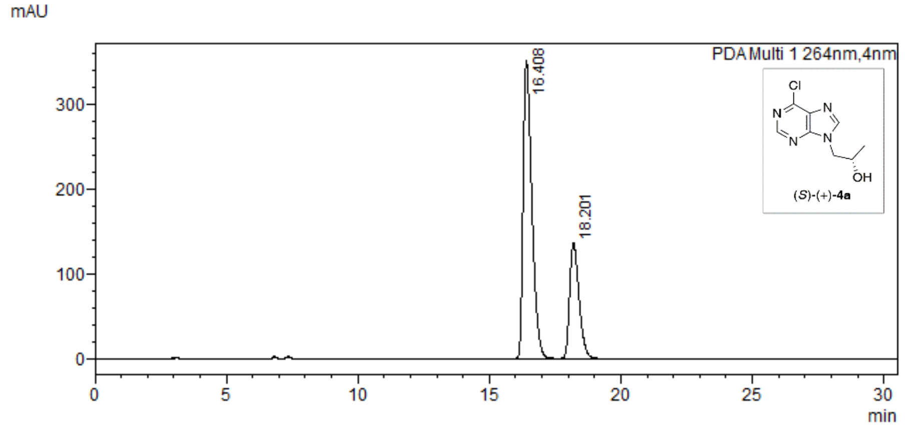

Peak Table

PDA Ch1 264nm

Peak#	Ret. Time	Area	Height	Height%	Area%
1	16.408	8175762	351679	72.116	70.743
2	18.201	3381176	135979	27.884	29.257
Total		11556938	487658	100.000	100.000

HPLC conditions [for (*R*)-(–)-5a]: *n*-hexane-*i*-PrOH (95:5, v/v); f=0.9 mL/min; λ=263 nm; Chiralcel OD-H

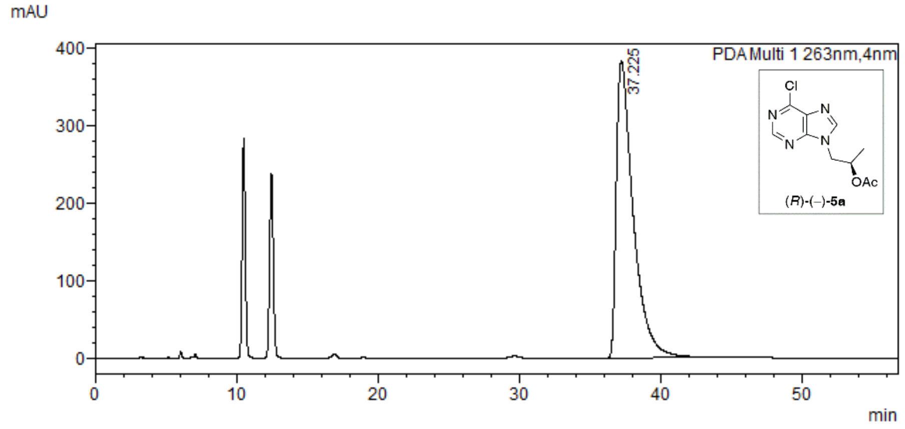

Peak Table

PDA Ch1 263nm

Peak#	Ret. Time	Area	Height	Height%	Area%
1	37.225	31883708	383597	100.000	100.000
Total		31883708	383597	100.000	100.000

HPLC analysis for the subsequent biocatalytic reaction:

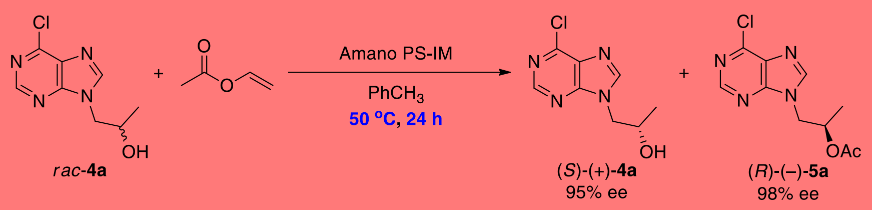

HPLC conditions [for (*S*)-(+)-4a]: *n*-hexane-*i*-PrOH (90:10, v/v); f=1.0 mL/min; λ=264 nm; Chiralcel OJ-H

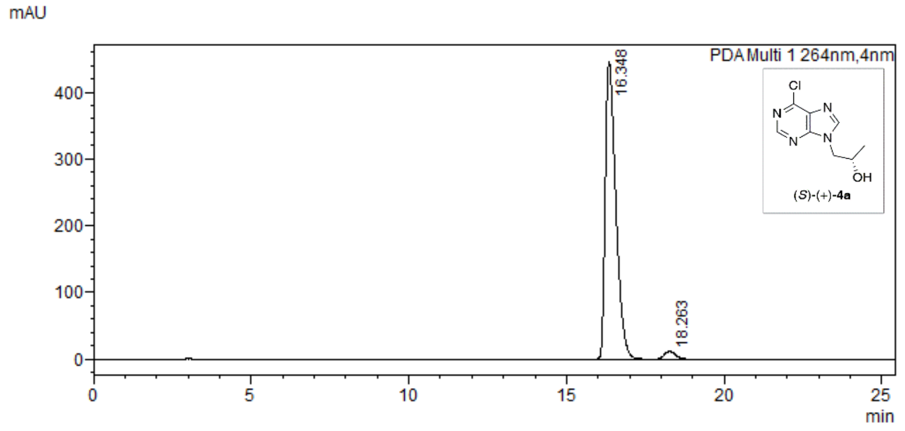

Peak Table

PDA Ch1 264nm

Peak#	Ret. Time	Area	Height	Height%	Area%
1	16.348	10411082	444954	97.456	97.388
2	18.263	279255	11616	2.544	2.612
Total		10690337	456570	100.000	100.000

HPLC conditions [for (*R*)-(–)-5a]: *n*-hexane-*i*-PrOH (95:5, v/v); f=0.9 mL/min; λ=263 nm; Chiralcel OD-H

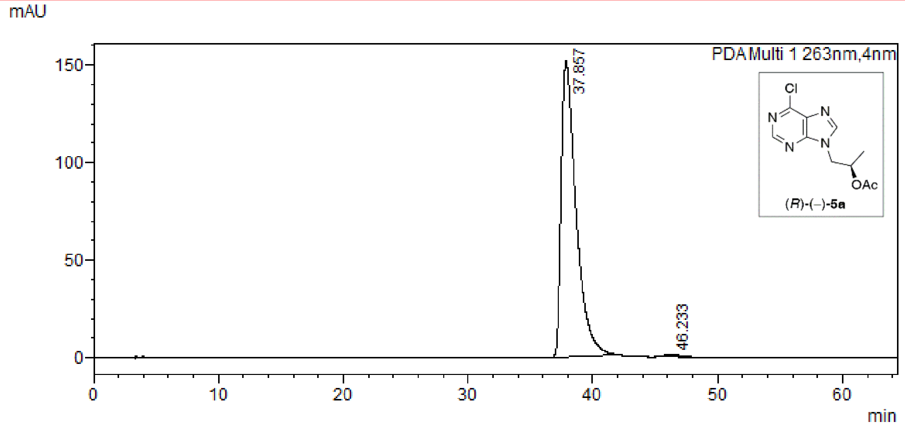

Peak Table

PDA Ch1 263nm

Peak#	Ret. Time	Area	Height	Height%	Area%
1	37.857	12610809	151685	99.187	99.080
2	46.233	117088	1243	0.813	0.920
Total		12727898	152928	100.000	100.000

HPLC analysis for the subsequent biocatalytic reaction:

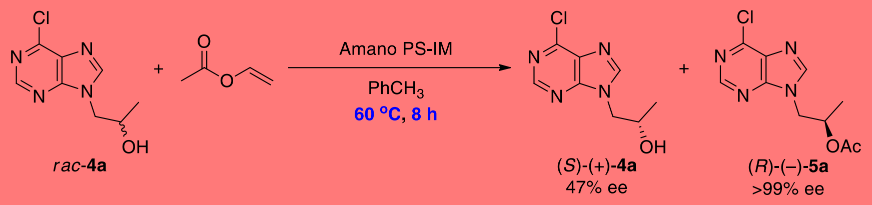

HPLC conditions [for (*S*)-(+)-4a]: *n*-hexane-*i*-PrOH (90:10, v/v); f=1.0 mL/min; λ=264 nm; Chiralcel OJ-H

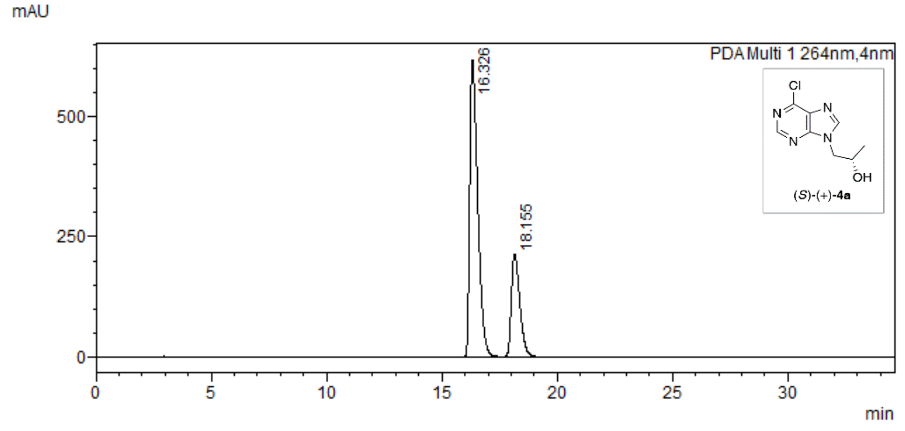

Peak Table

PDA Ch1 264nm

Peak#	Ret. Time	Area	Height	Height%	Area%
1	16.326	14919539	616807	74.270	73.342
2	18.155	5422897	213686	25.730	26.658
Total		20342436	830493	100.000	100.000

HPLC conditions [for (*R*)-(–)-5a]: *n*-hexane-*i*-PrOH (95:5, v/v); f=0.9 mL/min; λ=263 nm; Chiralcel OD-H

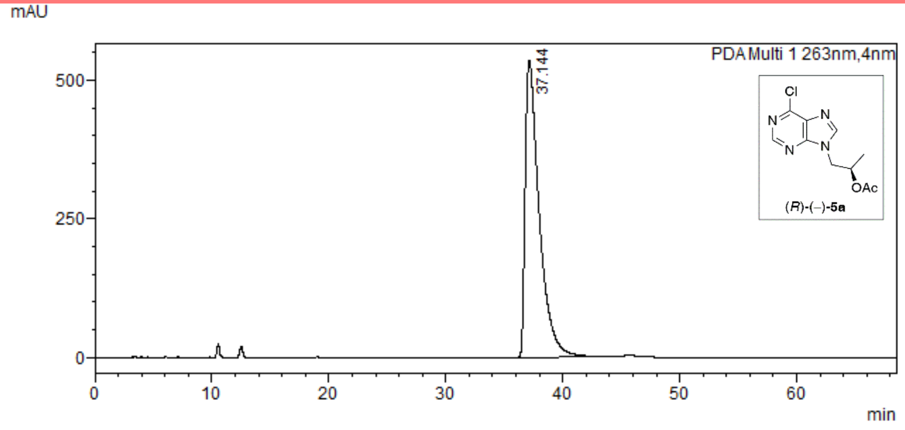

Peak Table

PDA Ch1 263nm

Peak#	Ret. Time	Area	Height	Height%	Area%
1	37.144	44813860	535160	100.000	100.000
Total		44813860	535160	100.000	100.000

HPLC analysis for the subsequent biocatalytic reaction:

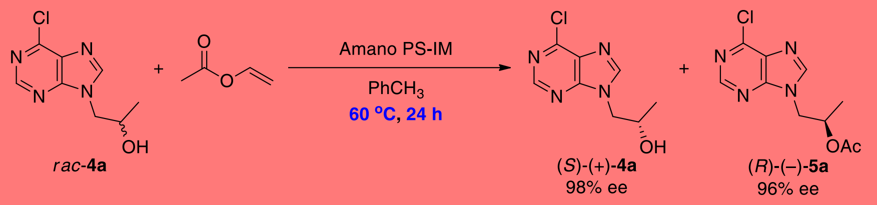

HPLC conditions [for (*S*)-(+)-4a]: *n*-hexane-*i*-PrOH (90:10, v/v); f=1.0 mL/min; λ=264 nm; Chiralcel OJ-H

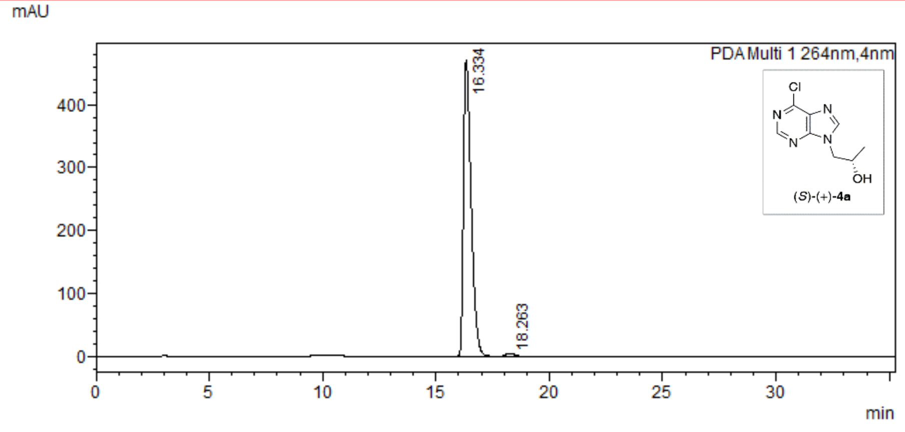

Peak Table

PDA Ch1 264nm

Peak#	Ret. Time	Area	Height	Height%	Area%
1	16.334	11078598	470119	99.030	99.020
2	18.263	109669	4604	0.970	0.980
Total		11188267	474723	100.000	100.000

HPLC conditions [for (*R*)-(–)-5a]: *n*-hexane-*i*-PrOH (95:5, v/v); f=0.9 mL/min; λ=263 nm; Chiralcel OD-H

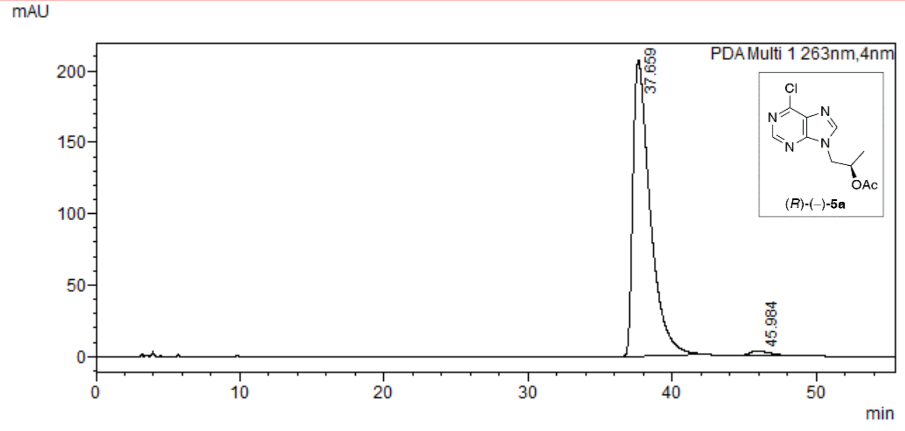

Peak Table

PDA Ch1 263nm

Peak#	Ret. Time	Area	Height	Height%	Area%
1	37.659	17292952	206994	98.493	98.238
2	45.984	310193	3167	1.507	1.762
Total		17603145	210160	100.000	100.000

HPLC analysis for the subsequent biocatalytic reaction:

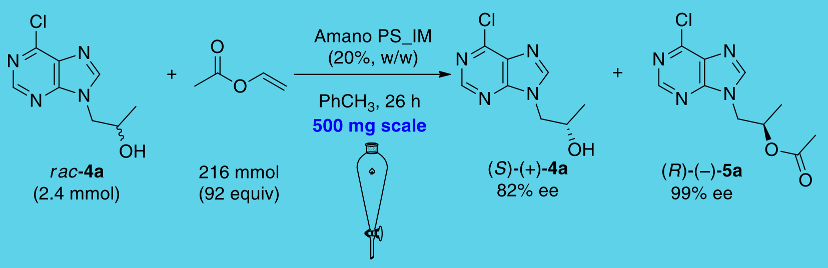

HPLC conditions [for (*S*)-(+)-4a]: *n*-hexane-*i*-PrOH (90:10, v/v); f=1.0 mL/min; λ=264 nm; Chiralcel OJ-H

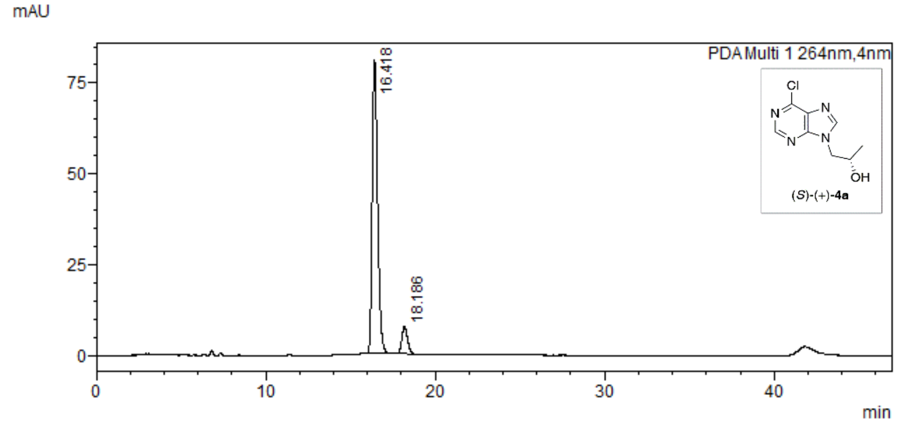

Peak Table

PDA Ch1 264nm

Peak#	Ret. Time	Area	Height	Height%	Area%
1	16.418	1780324	80774	91.410	90.803
2	18.186	180325	7590	8.590	9.197
Total		1960649	88364	100.000	100.000

HPLC conditions [for (*R*)-(–)-5a]: *n*-hexane-*i*-PrOH (95:5, v/v); f=0.9 mL/min; λ=263 nm; Chiralcel OD-H

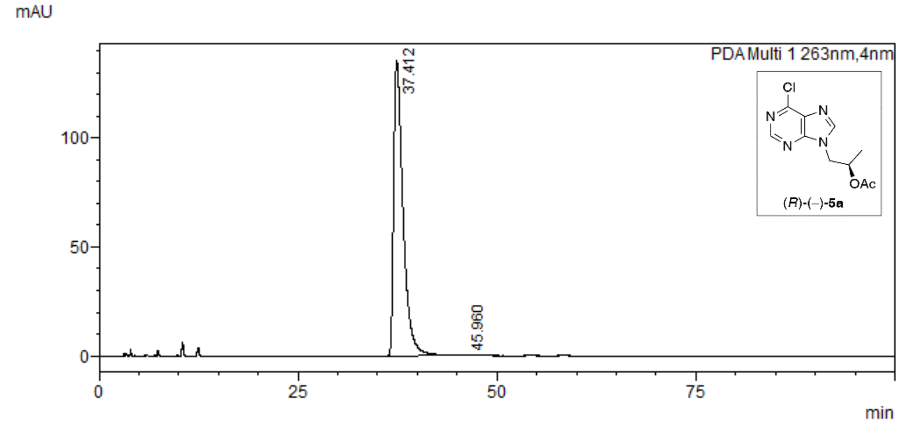

Peak Table

PDA Ch1 263nm

Peak#	Ret. Time	Area	Height	Height%	Area%
1	37.412	11110746	135345	99.713	99.703
2	45.960	33065	389	0.287	0.297
Total		11143811	135734	100.000	100.000

HPLC analysis for the subsequent biocatalytic reaction:

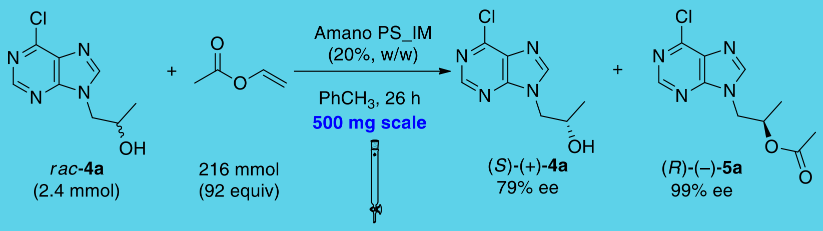

HPLC conditions [for (*S*)-(+)-4a]: *n*-hexane-*i*-PrOH (90:10, v/v); f=1.0 mL/min; λ=264 nm; Chiralcel OJ-H

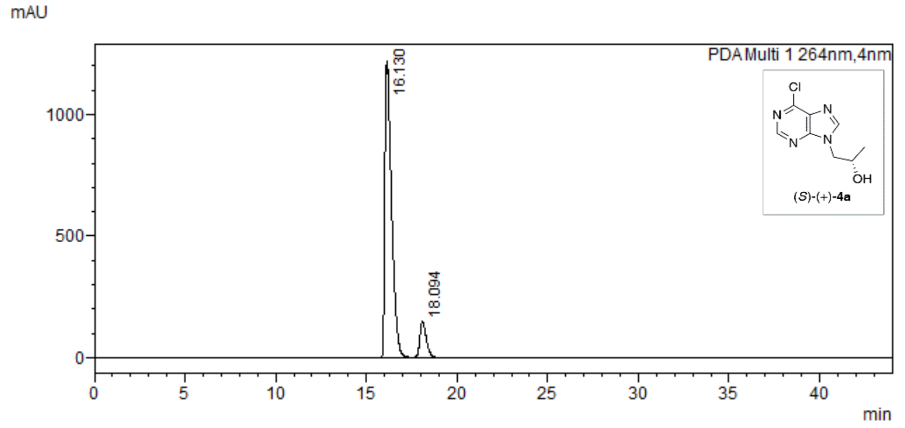

Peak Table

PDA Ch1 264nm

Peak#	Ret. Time	Area	Height	Height%	Area%
1	16.130	31586289	1218399	89.178	89.589
2	18.094	3670536	147857	10.822	10.411
Total		35256825	1366256	100.000	100.000

HPLC conditions [for (*R*)-(–)-5a]: *n*-hexane-*i*-PrOH (95:5, v/v); f=0.9 mL/min; λ=263 nm; Chiralcel OD-H

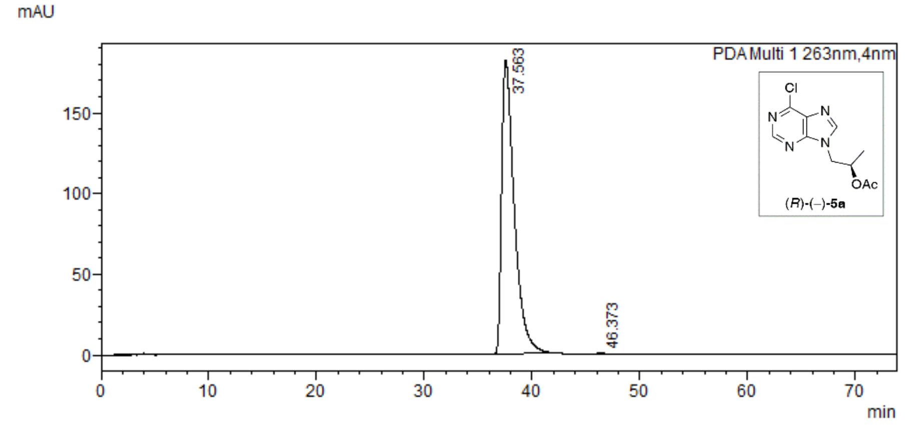

Peak Table

PDA Ch1 263nm

Peak#	Ret. Time	Area	Height	Height%	Area%
1	37.563	14895559	181982	99.675	99.624
2	46.373	56233	594	0.325	0.376
Total		14951792	182576	100.000	100.000

HPLC analysis for the subsequent biocatalytic reaction:

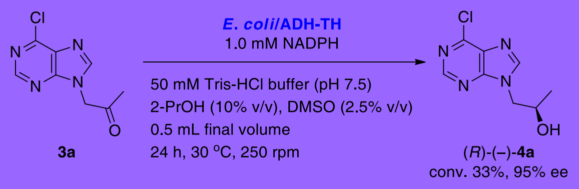

HPLC conditions [for (*R*)-(–)-4a]: *n*-hexane-*i*-PrOH (90:10, v/v); f=1.0 mL/min; λ=264 nm; Chiralcel OJ-H

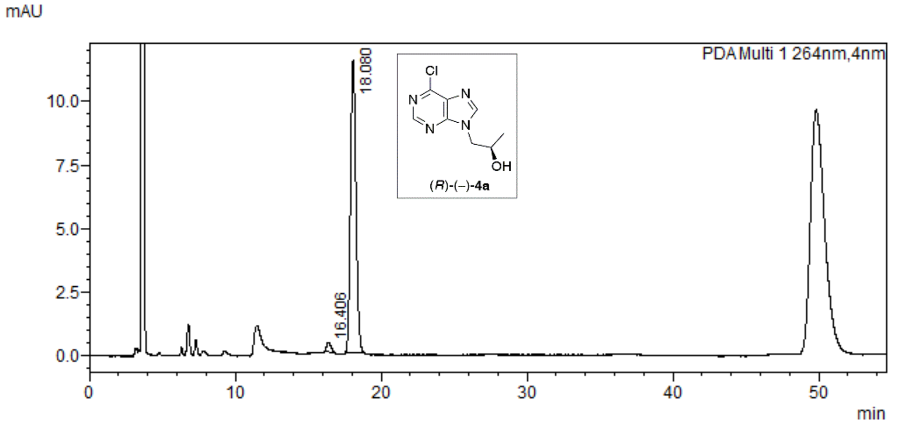

Peak Table

PDA Ch1 264nm

Peak#	Ret. Time	Area	Height	Height%	Area%
1	16.406	7513	367	3.090	2.418
2	18.080	303252	11517	96.910	97.582
Total		310765	11884	100.000	100.000

HPLC analysis for the subsequent biocatalytic reaction:

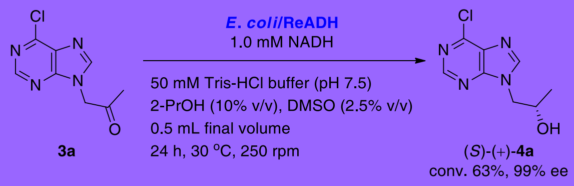

HPLC conditions [for (*S*)-(+)-4a]: *n*-hexane-*i*-PrOH (90:10, v/v); f=1.0 mL/min; λ=264 nm; Chiralcel OJ-H

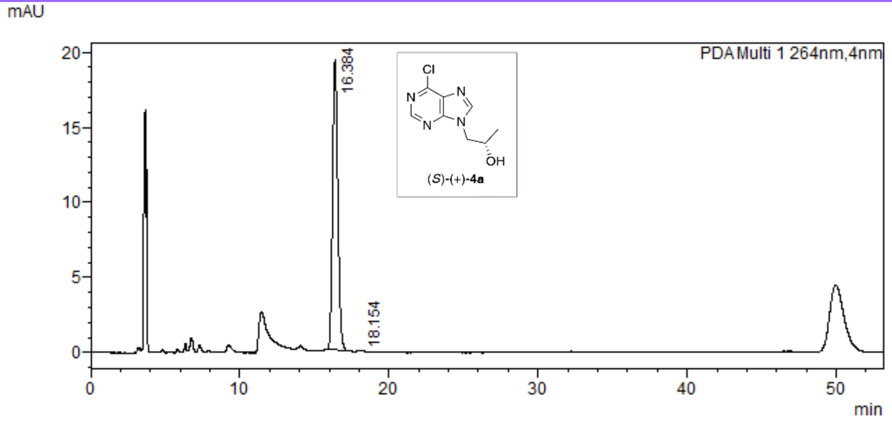

Peak Table

PDA Ch1 264nm

Peak#	Ret. Time	Area	Height	Height%	Area%
1	16.384	470872	19282	99.667	99.736
2	18.154	1245	64	0.333	0.264
Total		472117	19346	100.000	100.000

HPLC analysis for the subsequent biocatalytic reaction:

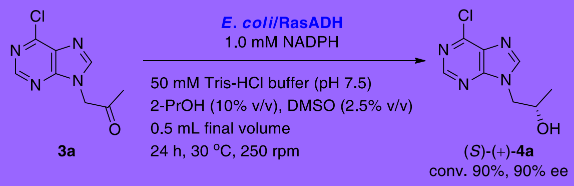

HPLC conditions [for (*S*)-(+)-4a]: *n*-hexane-*i*-PrOH (90:10, v/v); f=1.0 mL/min; λ=264 nm; Chiralcel OJ-H

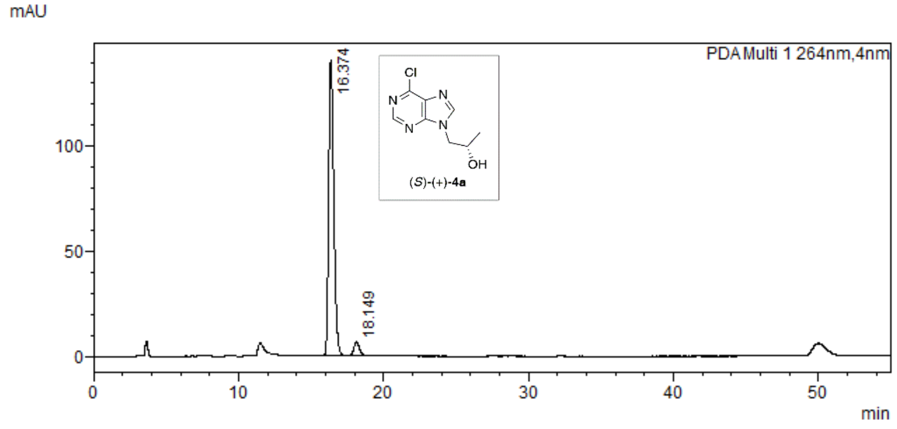

Peak Table

PDA Ch1 264nm

Peak#	Ret. Time	Area	Height	Height%	Area%
1	16.374	3450231	140581	95.482	95.149
2	18.149	175901	6652	4.518	4.851
Total		3626133	147232	100.000	100.000

HPLC analysis for the subsequent biocatalytic reaction:

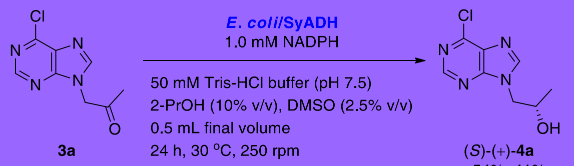

HPLC conditions [for (*S*)-(+)-4a]: *n*-hexane-*i*-PrOH (90:10, v/v); f=1.0 mL/min; λ=264 nm; Chiralcel OJ-H

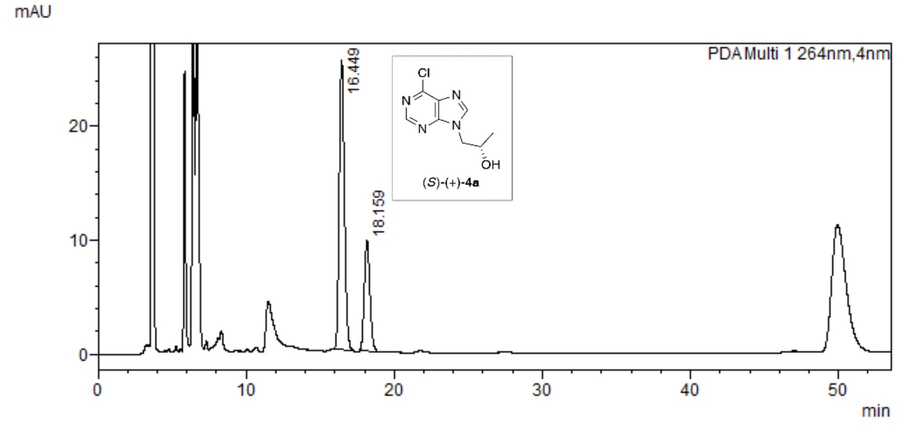

Peak Table

PDA Ch1 264nm

Peak#	Ret. Time	Area	Height	Height%	Area%
1	16.449	614515	25336	72.335	70.600
2	18.159	255902	9690	27.665	29.400
Total		870417	35025	100.000	100.000

HPLC analysis for the subsequent biocatalytic reaction:

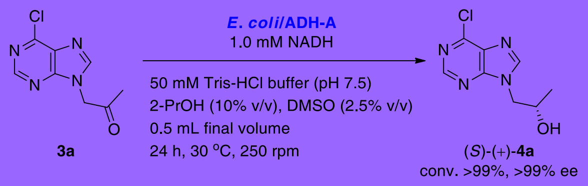

HPLC conditions [for (*S*)-(+)-4a]: *n*-hexane-*i*-PrOH (90:10, v/v); f=1.0 mL/min; λ=264 nm; Chiralcel OJ-H

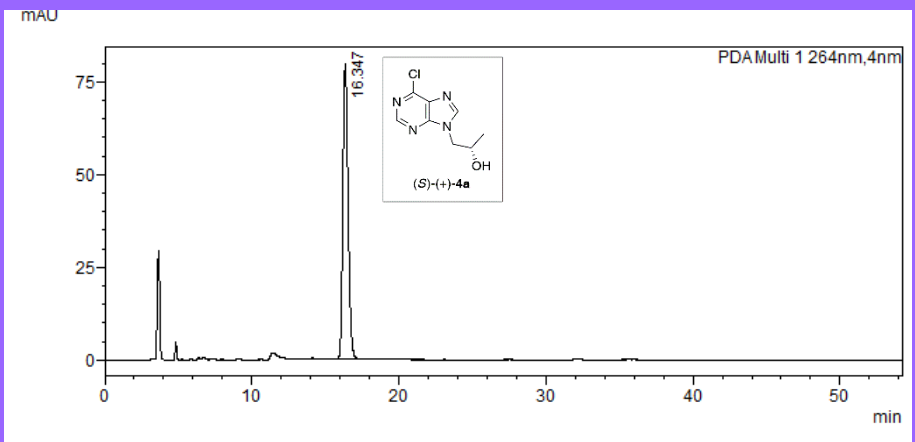

Peak Table

PDA Ch1 264nm

Peak#	Ret. Time	Area	Height	Height%	Area%
1	16.347	1946612	79532	100.000	100.000
Total		1946612	79532	100.000	100.000

HPLC analysis for the subsequent biocatalytic reaction:

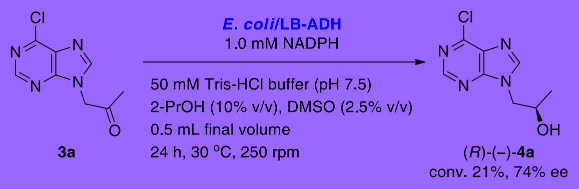

HPLC conditions [for (*R*)-(–)-4a]: *n*-hexane-*i*-PrOH (90:10, v/v); f=1.0 mL/min; λ=264 nm; Chiralcel OJ-H

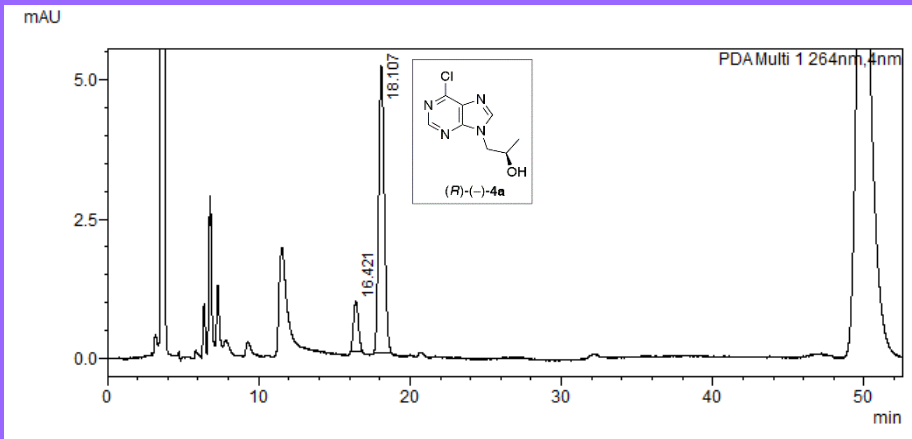

Peak Table

PDA Ch1 264nm

Peak#	Ret. Time	Area	Height	Height%	Area%
1	16.421	20098	902	14.919	13.067
2	18.107	133710	5142	85.081	86.933
Total		153807	6043	100.000	100.000

HPLC analysis for the subsequent biocatalytic reaction:

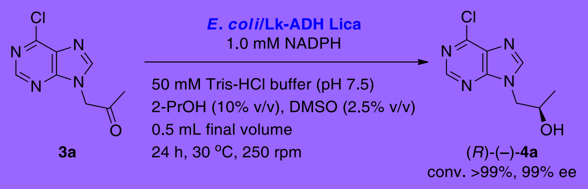

HPLC conditions [for (*R*)-(–)-4a]: *n*-hexane-*i*-PrOH (90:10, v/v); f=1.0 mL/min; λ=264 nm; Chiralcel OJ-H

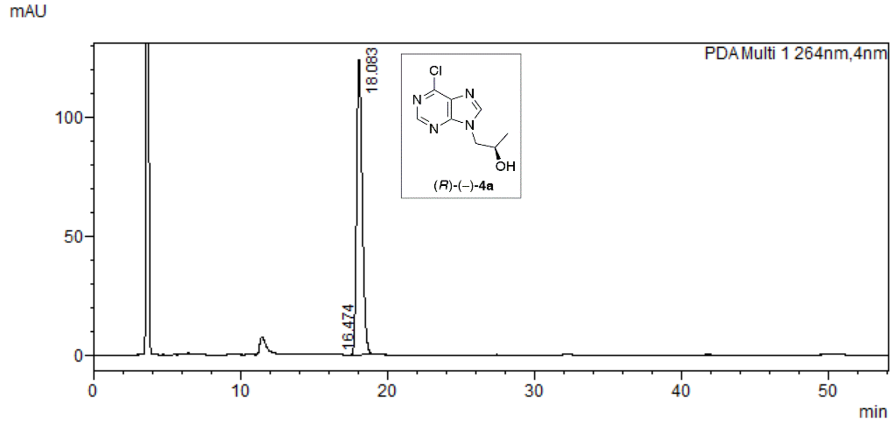

Peak Table

PDA Ch1 264nm

Peak#	Ret. Time	Area	Height	Height%	Area%
1	16.474	10461	482	0.388	0.317
2	18.083	3290532	123724	99.612	99.683
Total		3300993	124206	100.000	100.000

HPLC analysis for the subsequent biocatalytic reaction:

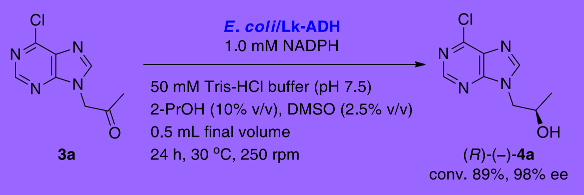

HPLC conditions [for (*R*)-(–)-4a]: *n*-hexane-*i*-PrOH (90:10, v/v); f=1.0 mL/min; λ=264 nm; Chiralcel OJ-H

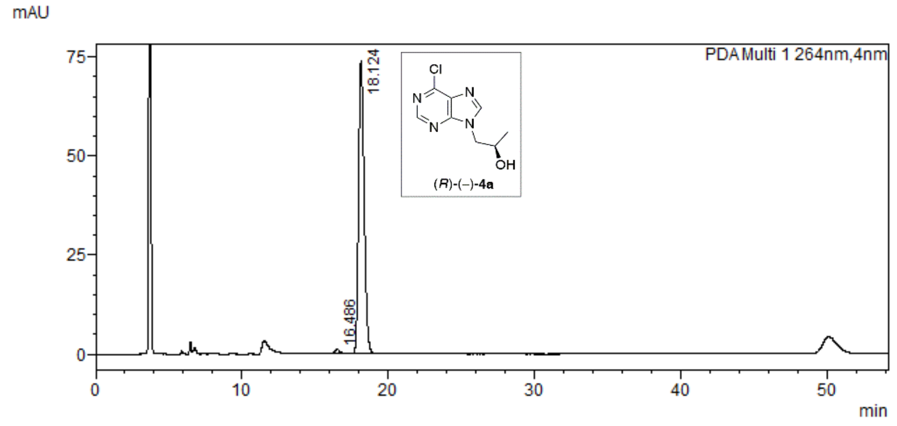

Peak Table

PDA Ch1 264nm

Peak#	Ret. Time	Area	Height	Height%	Area%
1	16.486	22956	1048	1.400	1.163
2	18.124	1951249	73790	98.600	98.837
Total		1974205	74838	100.000	100.000

HPLC analysis for the subsequent biocatalytic reaction:

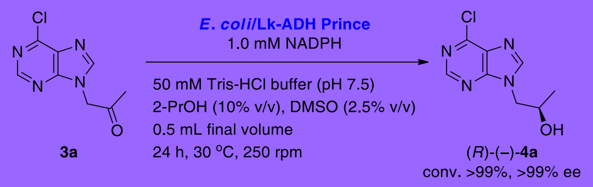

HPLC conditions [for (*R*)-(–)-4a]: *n*-hexane-*i*-PrOH (90:10, v/v); f=1.0 mL/min; λ=264 nm; Chiralcel OJ-H

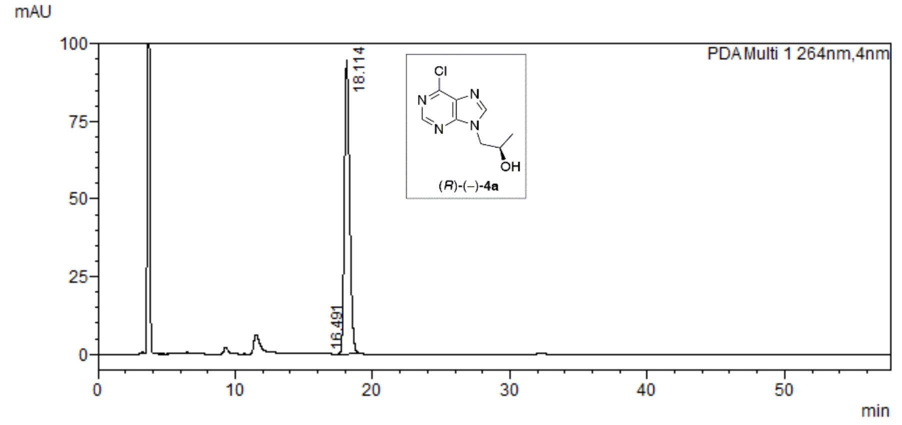

Peak Table

PDA Ch1 264nm

Peak#	Ret. Time	Area	Height	Height%	Area%
1	16.491	5781	295	0.311	0.231
2	18.114	2500019	94453	99.689	99.769
Total		2505800	94748	100.000	100.000

HPLC analysis for the subsequent biocatalytic reaction:

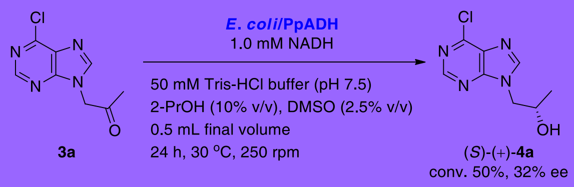

HPLC conditions [for (*S*)-(+)-4a]: *n*-hexane-*i*-PrOH (90:10, v/v); f=1.0 mL/min; λ=264 nm; Chiralcel OJ-H

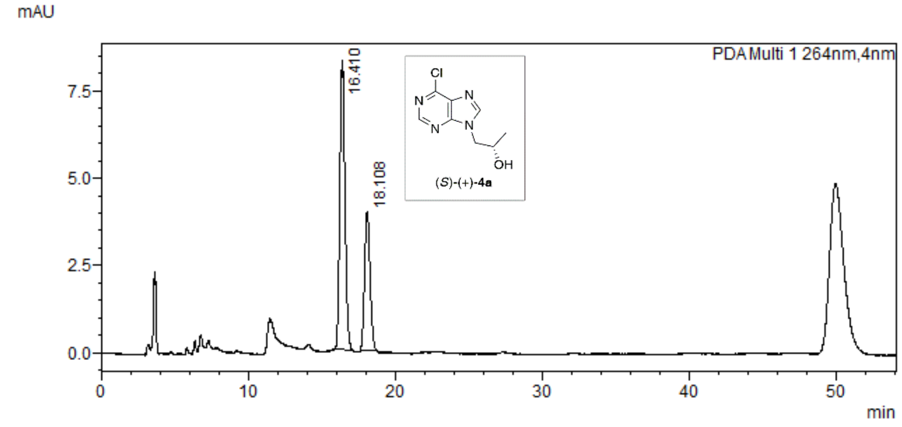

Peak Table

PDA Ch1 264nm

Peak#	Ret. Time	Area	Height	Height%	Area%
1	16.410	201175	8263	67.560	66.206
2	18.108	102688	3968	32.440	33.794
Total		303863	12231	100.000	100.000

HPLC analysis for the subsequent biocatalytic reaction:

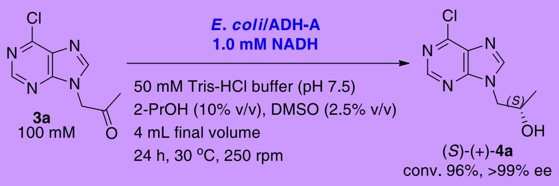

HPLC conditions [for (*S*)-(+)-4a]: *n*-hexane-*i*-PrOH (90:10, v/v); f=1.0 mL/min; λ=264 nm; Chiralcel OJ-H

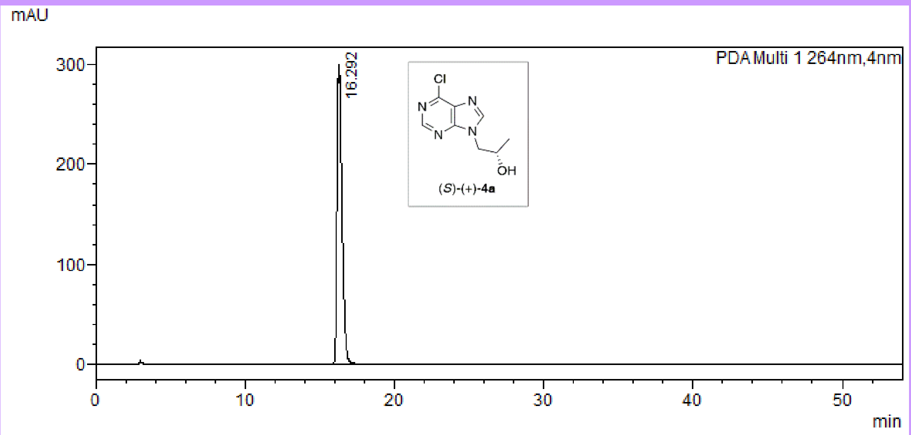

Peak Table

PDA Ch1 264nm

Peak#	Ret. Time	Area	Height	Height%	Area%
1	16.292	6820391	300084	100.000	100.000
Total		6820391	300084	100.000	100.000

HPLC analysis for the subsequent biocatalytic reaction:

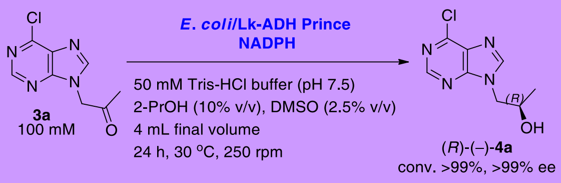

HPLC conditions [for (*R*)-(–)-4a]: *n*-hexane-*i*-PrOH (90:10, v/v); f=1.0 mL/min; λ=264 nm; Chiralcel OJ-H

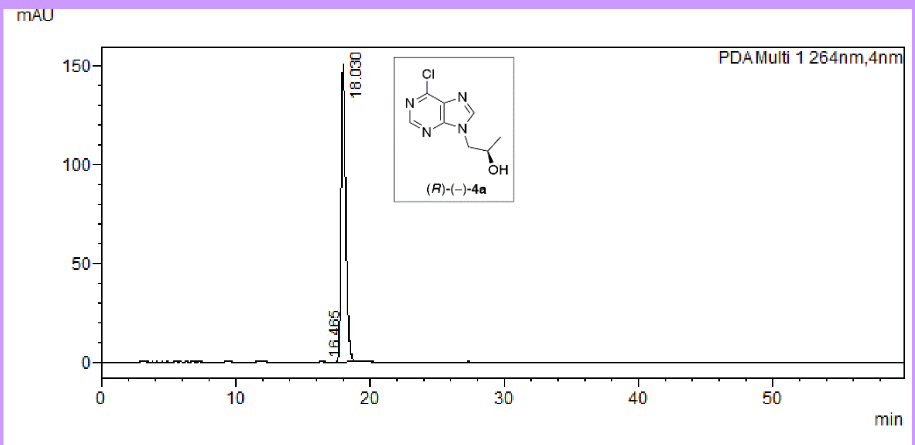

Peak Table

PDA Ch1 264nm

Peak#	Ret. Time	Area	Height	Height%	Area%
1	16.465	3481	221	0.147	0.095
2	18.030	3656845	150479	99.853	99.905
Total		3660325	150700	100.000	100.000

HPLC analysis for the subsequent biocatalytic reaction:

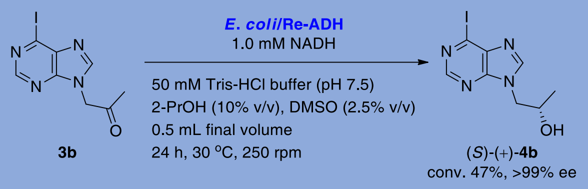

HPLC conditions [for (*S*)-(+)-4b]: *n*-hexane-*i*-PrOH (90:10, v/v); f=1.0 mL/min; λ=272 nm; Chiralcel OJ-H

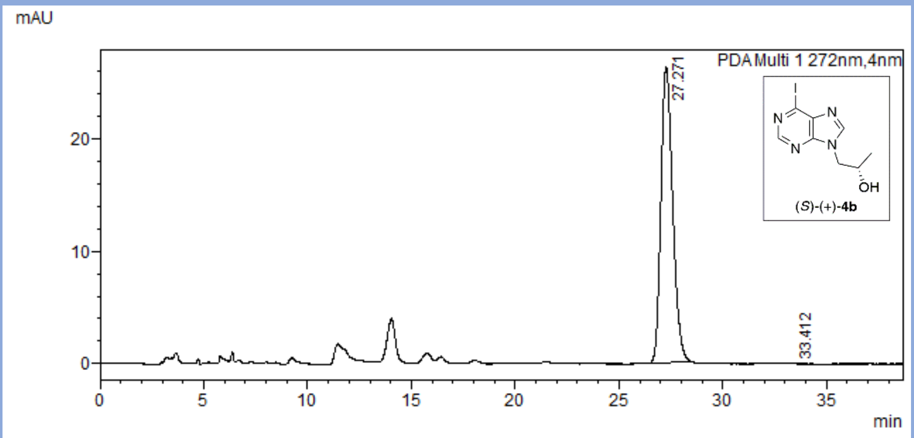

Peak Table

PDA Ch1 272nm

Peak#	Ret. Time	Area	Height	Height%	Area%
1	27.271	1035649	26228	99.746	99.777
2	33.412	2316	67	0.254	0.223
Total		1037965	26295	100.000	100.000

HPLC analysis for the subsequent biocatalytic reaction:

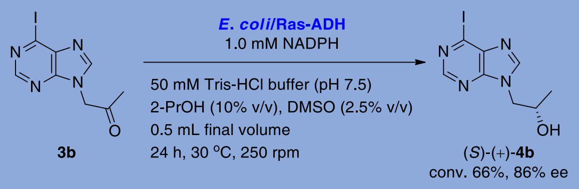

HPLC conditions [for (*S*)-(+)-4b]: *n*-hexane-*i*-PrOH (90:10, v/v); f=1.0 mL/min; λ=272 nm; Chiralcel OJ-H

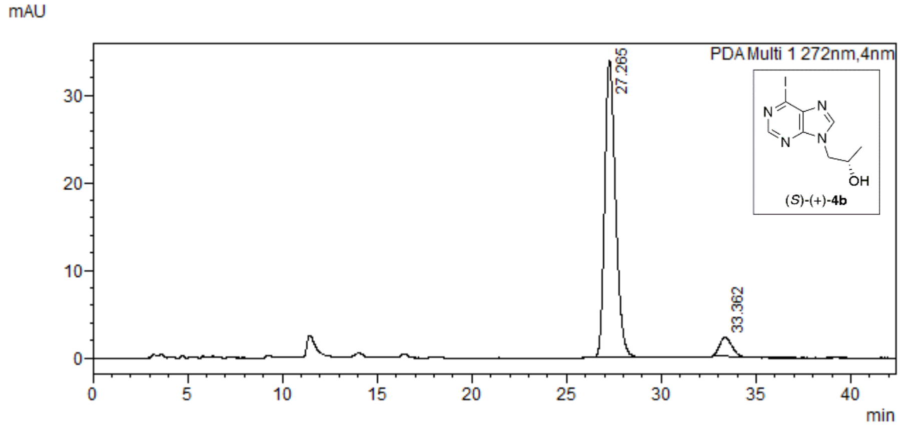

Peak Table

PDA Ch1 272nm

Peak#	Ret. Time	Area	Height	Height%	Area%
1	27.265	1344148	33861	93.882	93.219
2	33.362	97770	2207	6.118	6.781
Total		1441919	36068	100.000	100.000

HPLC analysis for the subsequent biocatalytic reaction:

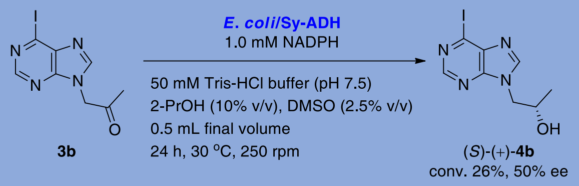

HPLC conditions [for (*S*)-(+)-4b]: *n*-hexane-*i*-PrOH (90:10, v/v); f=1.0 mL/min; λ=272 nm; Chiralcel OJ-H

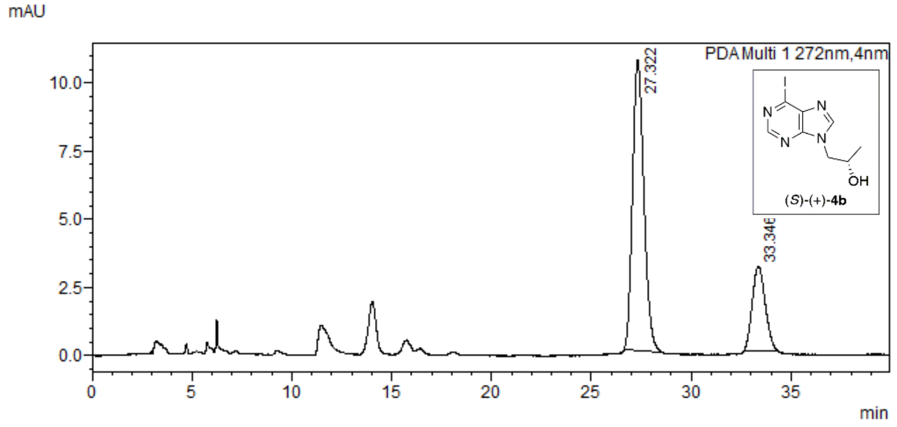

Peak Table

PDA Ch1 272nm

Peak#	Ret. Time	Area	Height	Height%	Area%
1	27.322	422088	10651	77.358	74.891
2	33.346	141515	3117	22.642	25.109
Total		563603	13768	100.000	100.000

HPLC analysis for the subsequent biocatalytic reaction:

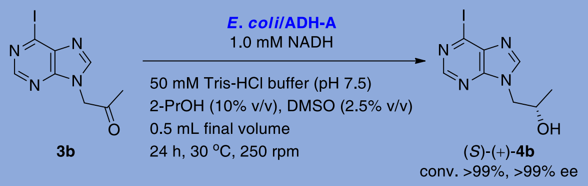

HPLC conditions [for (*S*)-(+)-4b]: *n*-hexane-*i*-PrOH (90:10, v/v); f=1.0 mL/min; λ=272 nm; Chiralcel OJ-H

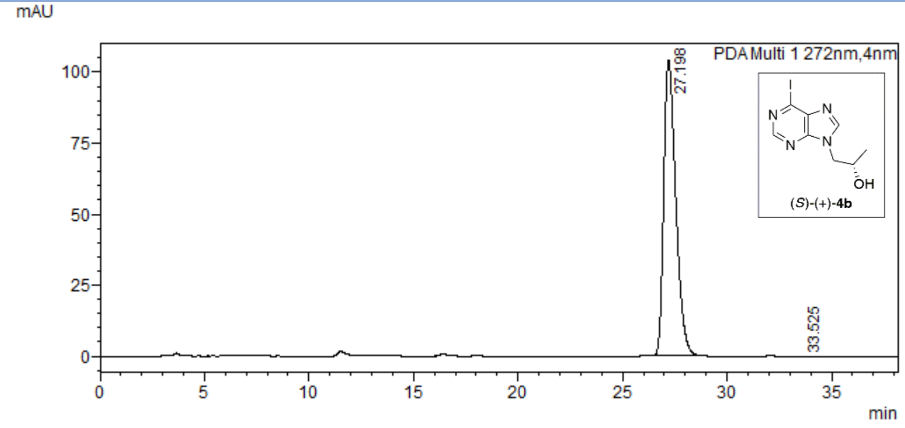

Peak Table

PDA Ch1 272nm

Peak#	Ret. Time	Area	Height	Height%	Area%
1	27.198	4162642	103814	99.911	99.922
2	33.525	3257	93	0.089	0.078
Total		4165899	103906	100.000	100.000

HPLC analysis for the subsequent biocatalytic reaction:

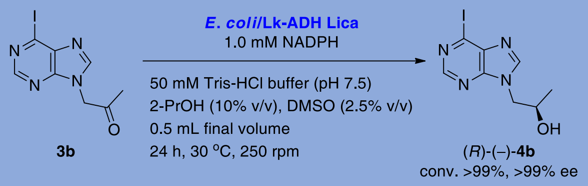

HPLC conditions [for (*R*)-(–)-4b]: *n*-hexane-*i*-PrOH (90:10, v/v); f=1.0 mL/min; λ=272 nm; Chiralcel OJ-H

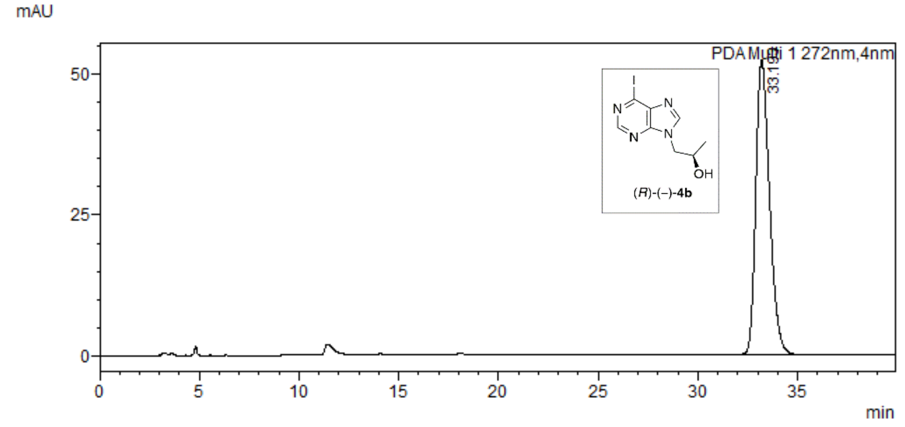

Peak Table

PDA Ch1 272nm

Peak#	Ret. Time	Area	Height	Height%	Area%
1	33.193	2497958	52270	100.000	100.000
Total		2497958	52270	100.000	100.000

HPLC analysis for the subsequent biocatalytic reaction:

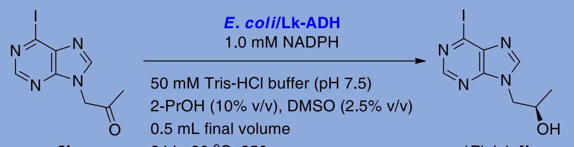

3b

(*R*)-(–)-**4b**
conv. 55%, 87% ee

HPLC conditions [for (*R*)-(–)-4b]: *n*-hexane-*i*-PrOH (90:10, v/v); f=1.0 mL/min; λ=272 nm; Chiralcel OJ-H

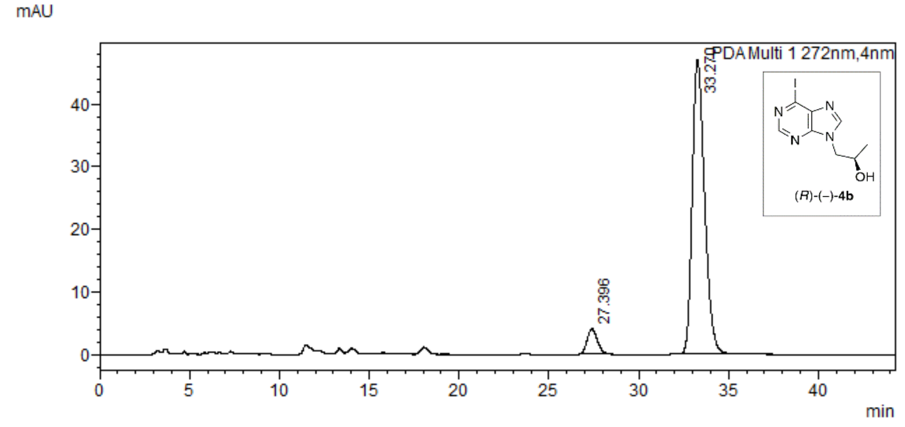

Peak Table

PDA Ch1 272nm

Peak#	Ret. Time	Area	Height	Height%	Area%
1	27.396	154997	4005	7.879	6.509
2	33.270	2226280	46832	92.121	93.491
Total		2381278	50837	100.000	100.000

HPLC analysis for the subsequent biocatalytic reaction:

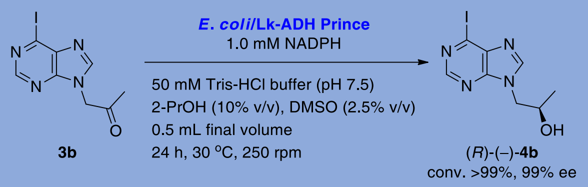

HPLC conditions [for (*R*)-(–)-4b]: *n*-hexane-*i*-PrOH (90:10, v/v); f=1.0 mL/min; λ=272 nm; Chiralcel OJ-H

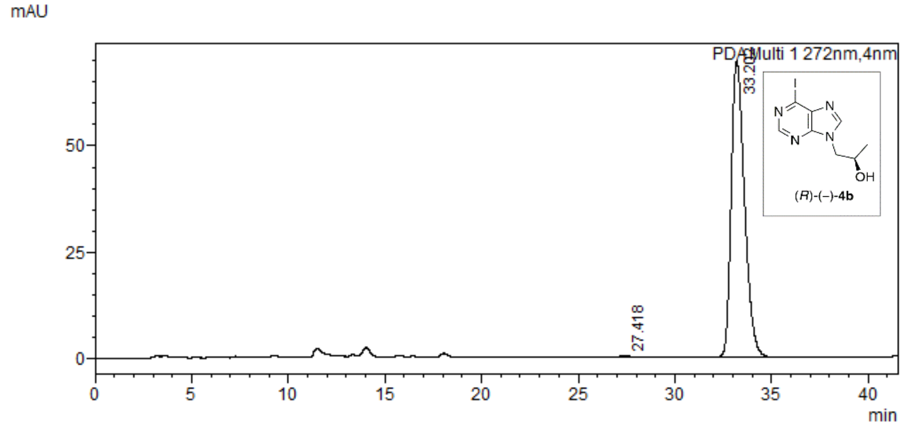

Peak Table

PDA Ch1 272nm

Peak#	Ret. Time	Area	Height	Height%	Area%
1	27.418	16261	447	0.636	0.486
2	33.202	3329257	69778	99.364	99.514
Total		3345518	70225	100.000	100.000

HPLC analysis for the subsequent biocatalytic reaction:

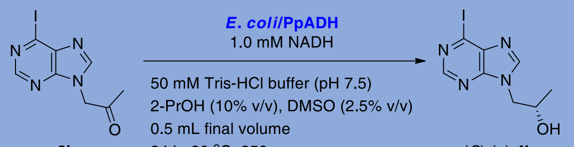

3b → (*S*)-(+)-**4b**

conv. 11%, 89% ee

HPLC conditions [for (*S*)-(+)-4b]: *n*-hexane-*i*-PrOH (90:10, v/v); f=1.0 mL/min; λ=272 nm; Chiralcel OJ-H

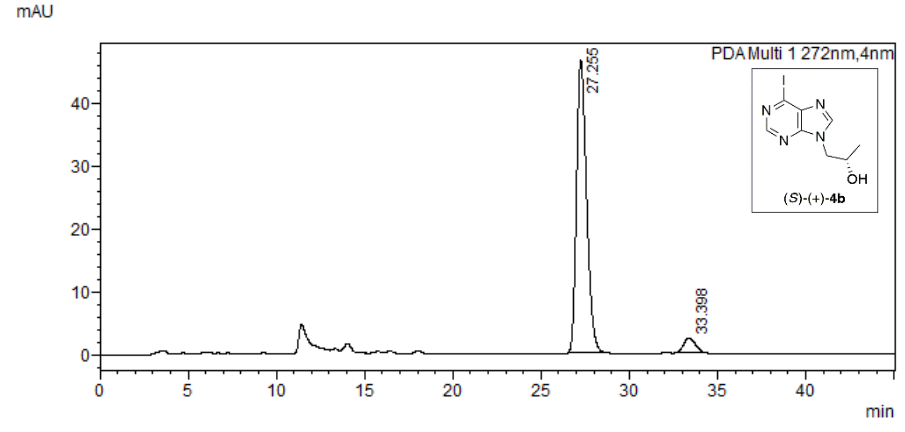

Peak Table

PDA Ch1 272nm

Peak#	Ret. Time	Area	Height	Height%	Area%
1	27.255	1849590	46479	95.190	94.644
2	33.398	104679	2349	4.810	5.356
Total		1954269	48827	100.000	100.000

HPLC of *rac*-7 on Chiralcel OJ-H at 30 °C

Conditions: *n*-hexane-2-PrOH:DEA (95:5:0.1), v/v); f=0.8 mL/min; λ=261 nm; *p*=3.4 MPa

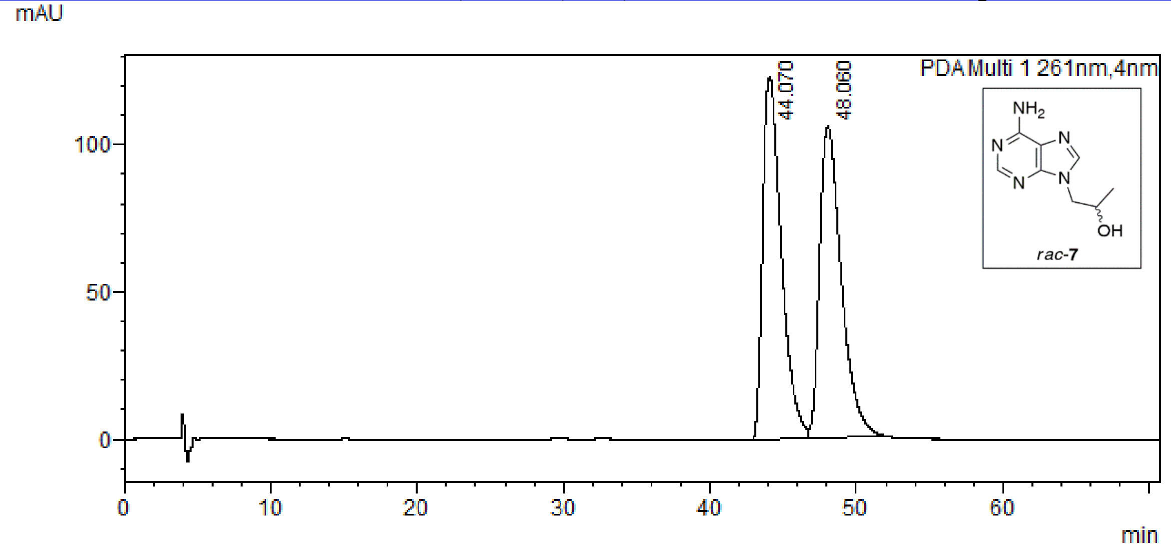

Peak Table

PDA Ch1 261nm

Peak#	Ret. Time	Area	Height	Height%	Area%
1	44.070	10947892	122935	53.758	50.024
2	48.060	10937594	105749	46.242	49.976
Total		21885486	228683	100.000	100.000

HPLC of (*R*)-(–)-7 on Chiralcel OJ-H at 30 °C

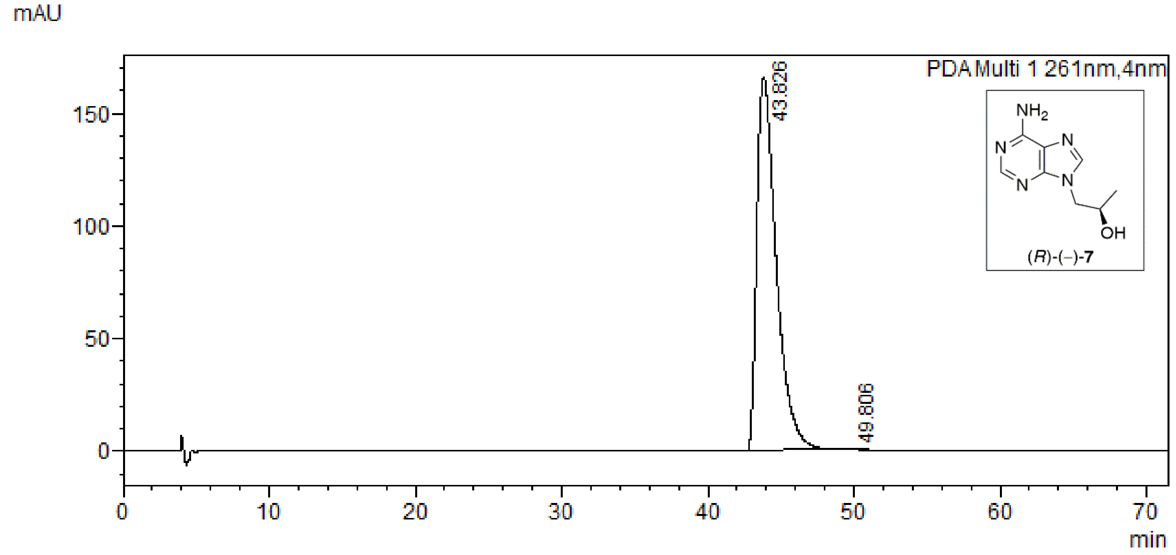

Peak Table

PDA Ch1 261nm

Peak#	Ret. Time	Area	Height	Height%	Area%
1	43.826	15268468	165451	99.711	99.724
2	49.806	42307	480	0.289	0.276
Total		15310775	165931	100.000	100.000

HPLC of *rac*-10 on Chiralpak AD-H at 30 °C

Conditions: *n*-hexane-2-PrOH (85:15, v/v); f=0.8 mL/min; λ=260 nm; *p*=3.8 MPa

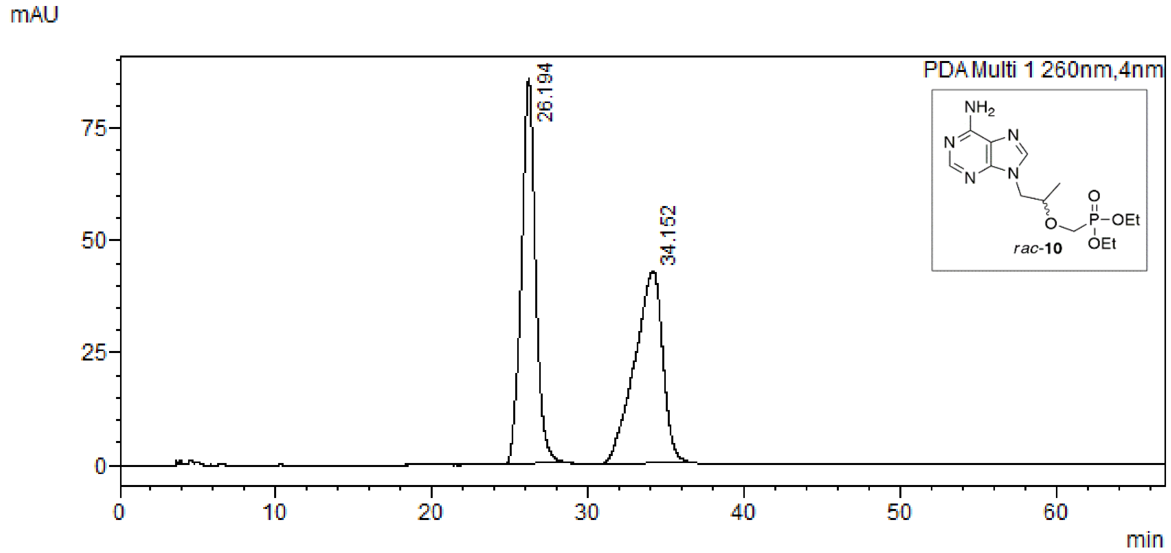

Peak Table

PDA Ch1 260nm

Peak#	Ret. Time	Area	Height	Height%	Area%
1	26.194	5384744	85557	66.655	50.043
2	34.152	5375575	42800	33.345	49.957
Total		10760319	128357	100.000	100.000

HPLC of (*R*)-(–)-10 on Chiralpak AD-H at 30 °C

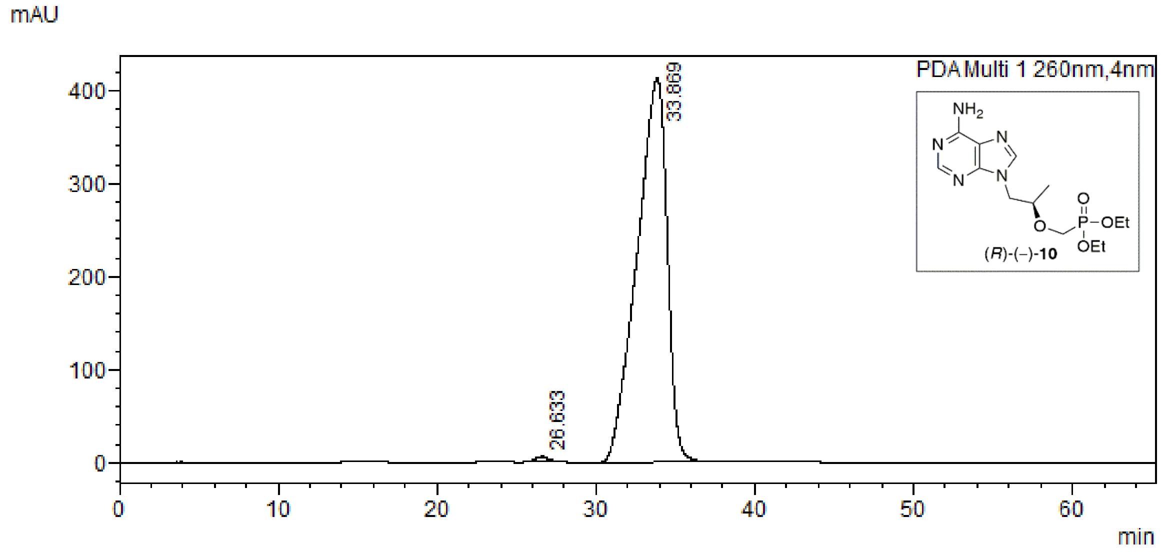

Peak Table

PDA Ch1 260nm

Peak#	Ret. Time	Area	Height	Height%	Area%
1	26.633	355023	5966	1.427	0.648
2	33.869	54416895	412132	98.573	99.352
Total		54771918	418098	100.000	100.000

12. Spectral data (copies of NMR, IR and FTMS spectra)

6-Iodopurine (2)

^{1}H NMR spectrum of **2** (500 MHz, DMSO-d_6)

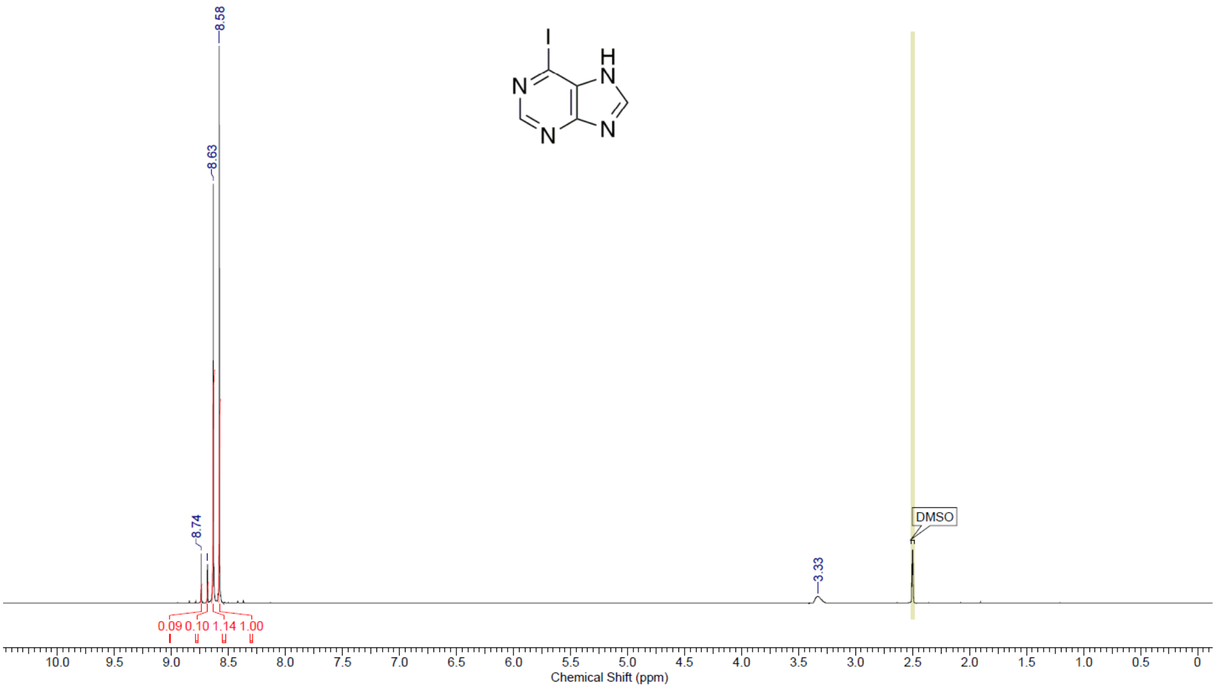

^{13}C{^{1}H} NMR spectrum of **2** (126 MHz, DMSO-d_6)

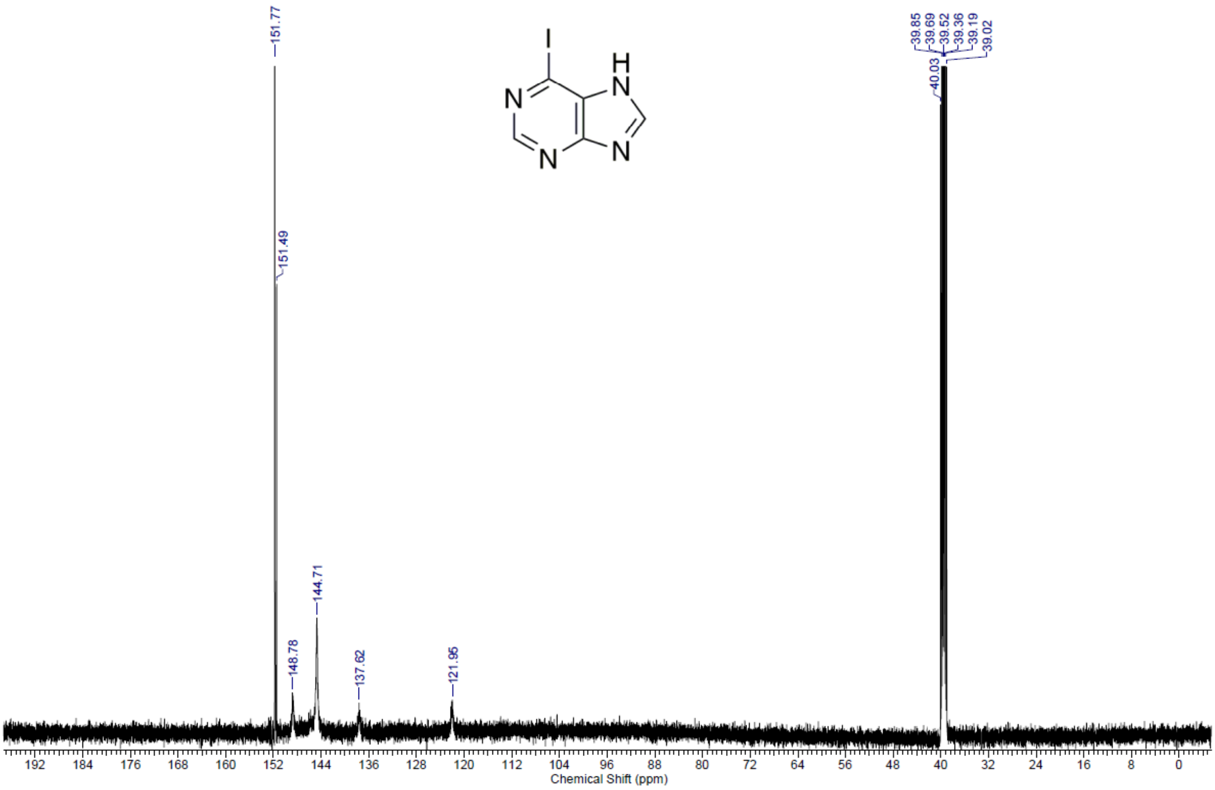

IR spectrum of **2** (Nujol)

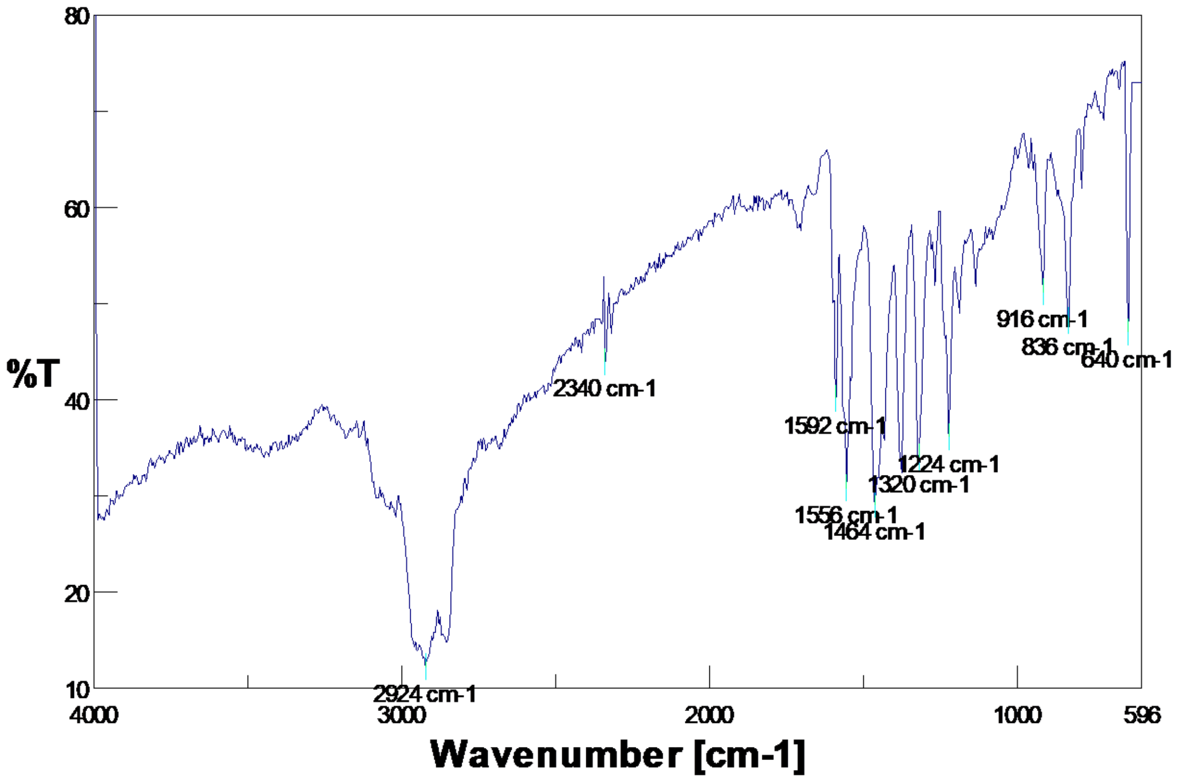

FTMS spectrum of **2** (ESI-TOF)

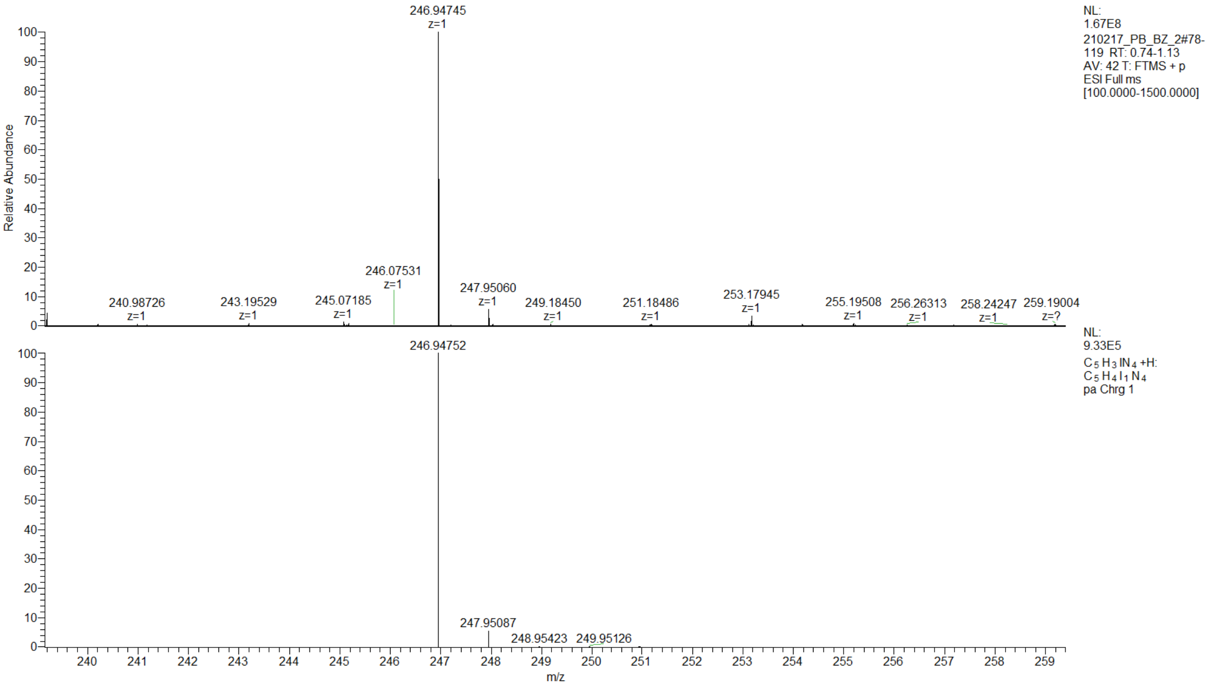

1-(6-Chloro-9H-purin-9-yl)propan-2-one (3a)

^{1}H NMR spectrum of **3a** (500 MHz, $CDCl_3$)

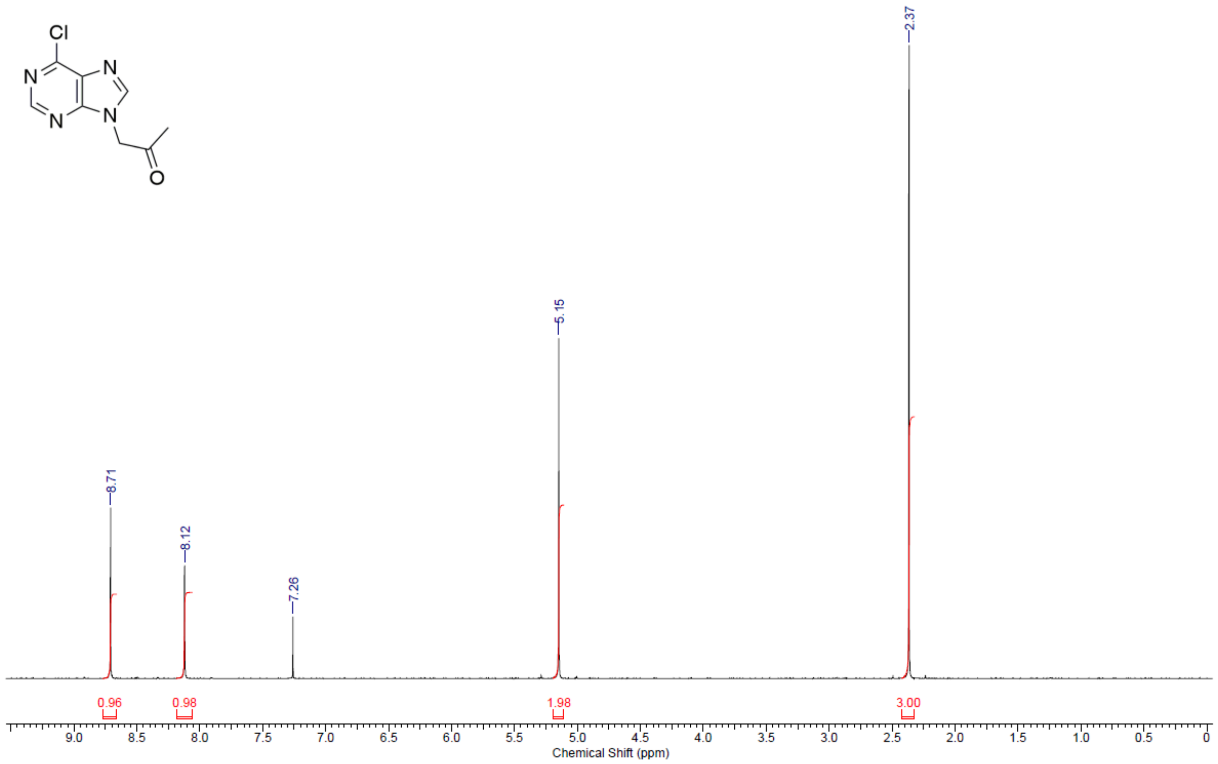

^{13}C{^{1}H} NMR spectrum of **3a** (126 MHz, $CDCl_3$)

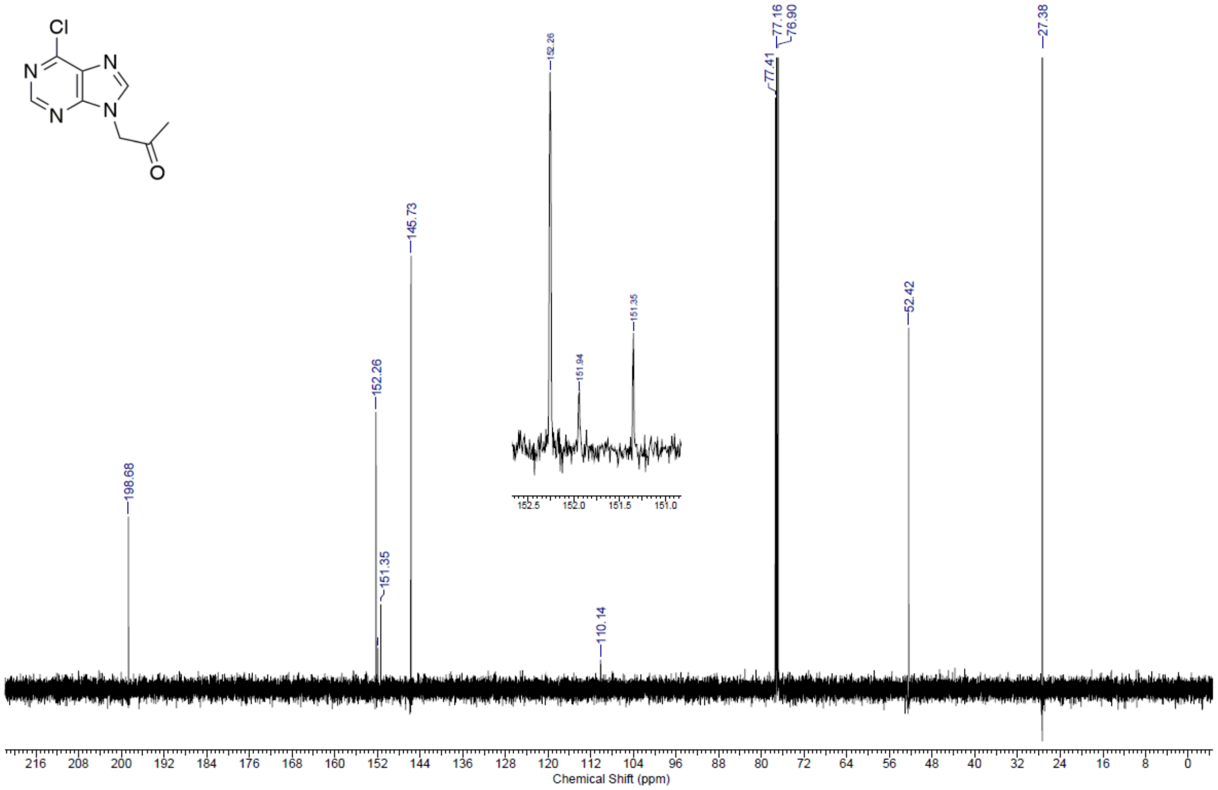

IR spectrum of **3a** (Nujol)

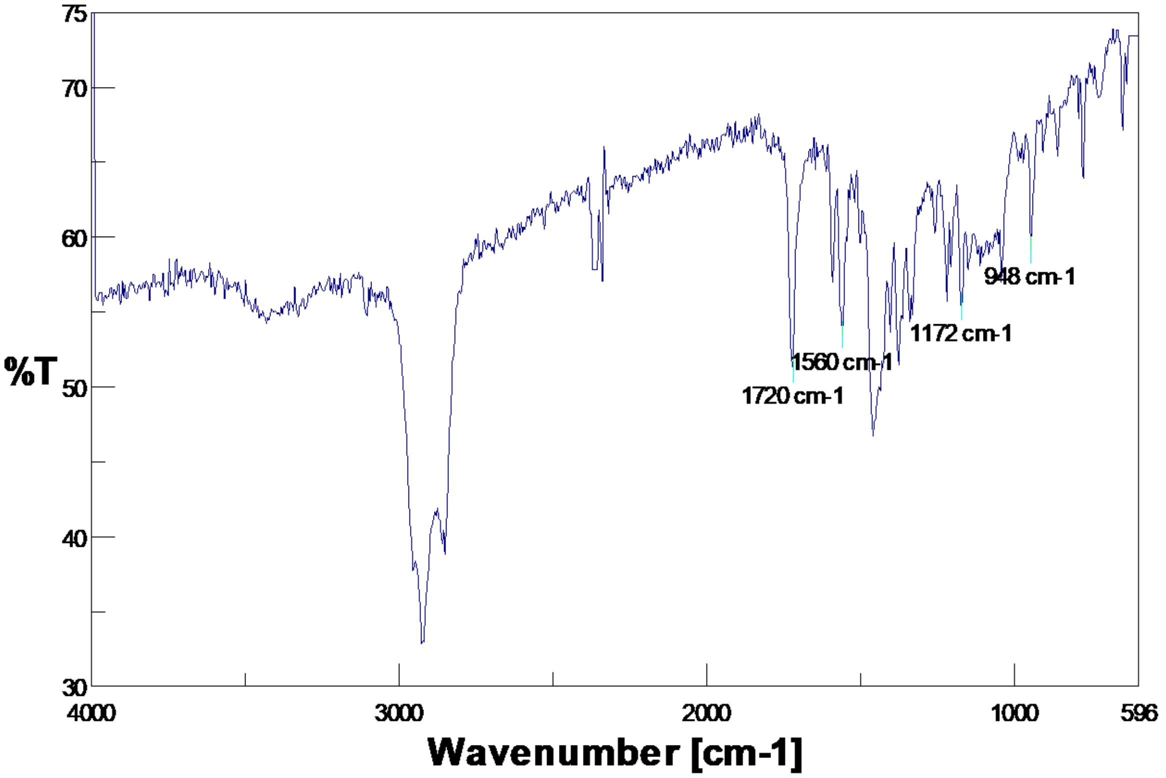

FTMS spectrum of **3a** (ESI-TOF)

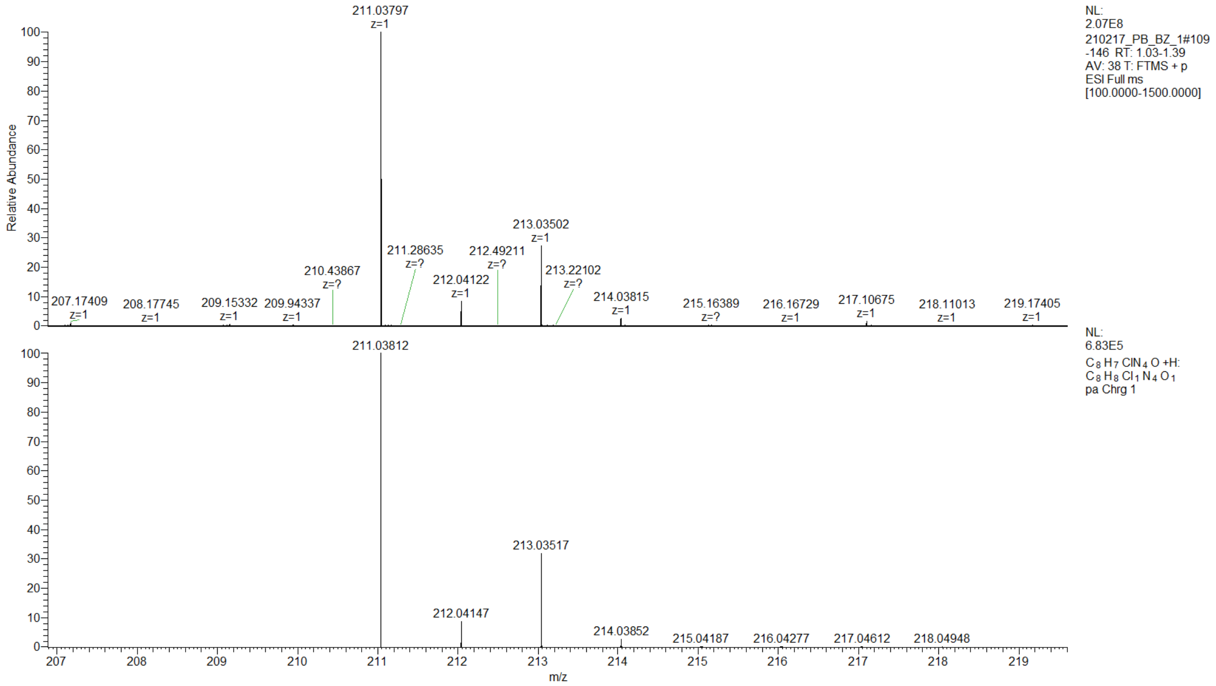

1-(6-Iodo-9H-purin-9-yl)propan-2-one (3b)

^{1}H NMR spectrum of **3b** (500 MHz, $CDCl_3$)

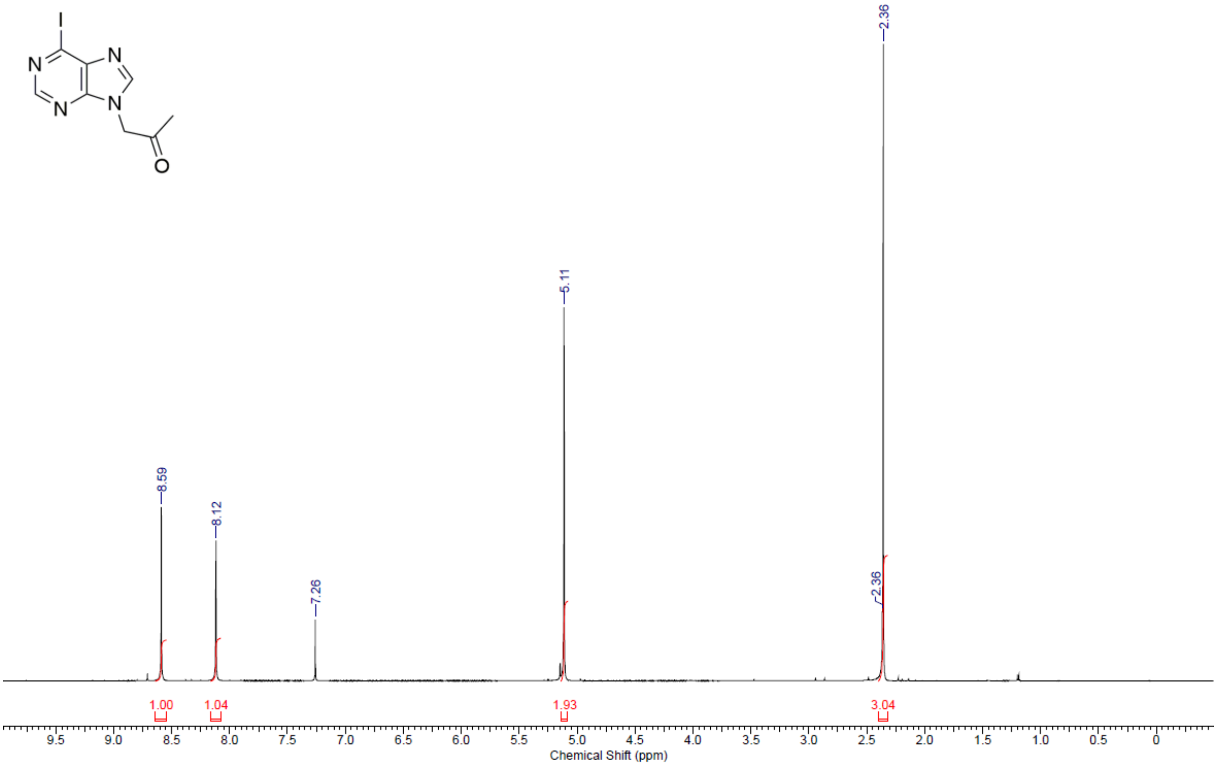

^{13}C{^{1}H} NMR spectrum of **3b** (126 MHz, $CDCl_3$)

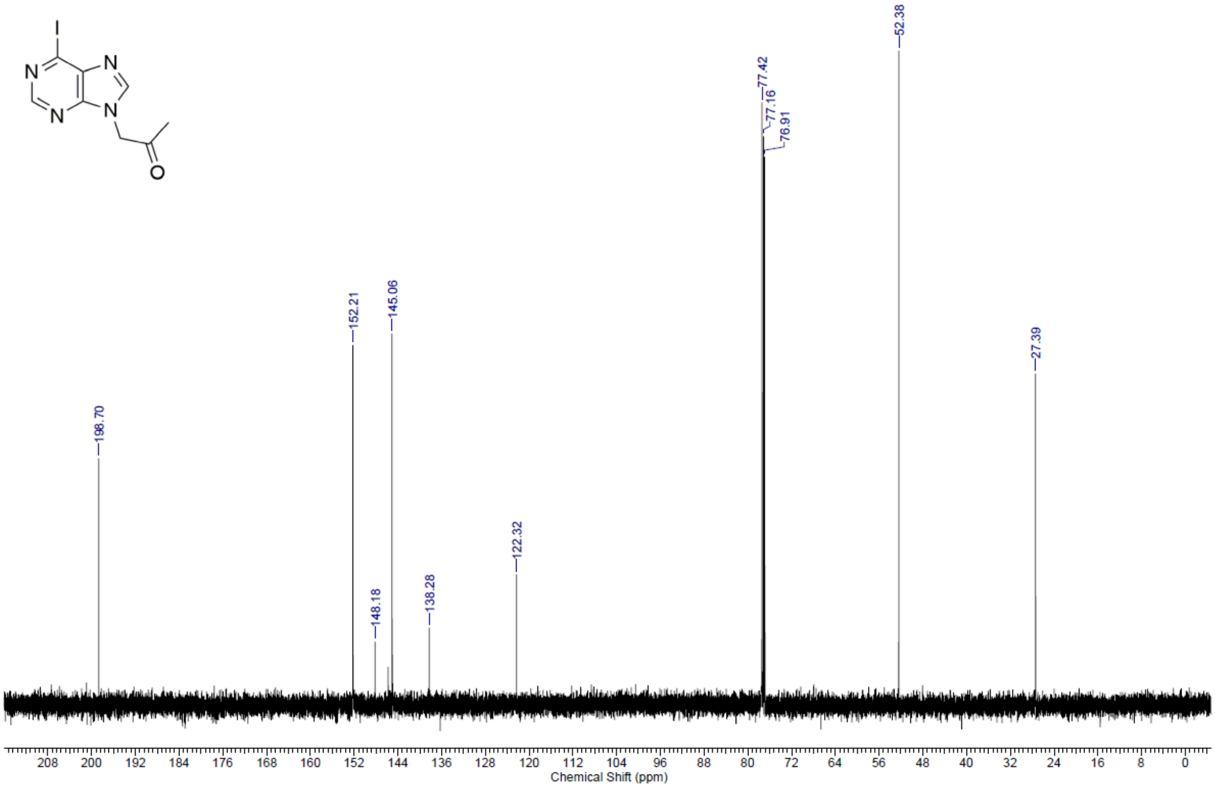

IR spectrum of **3b** (Nujol)

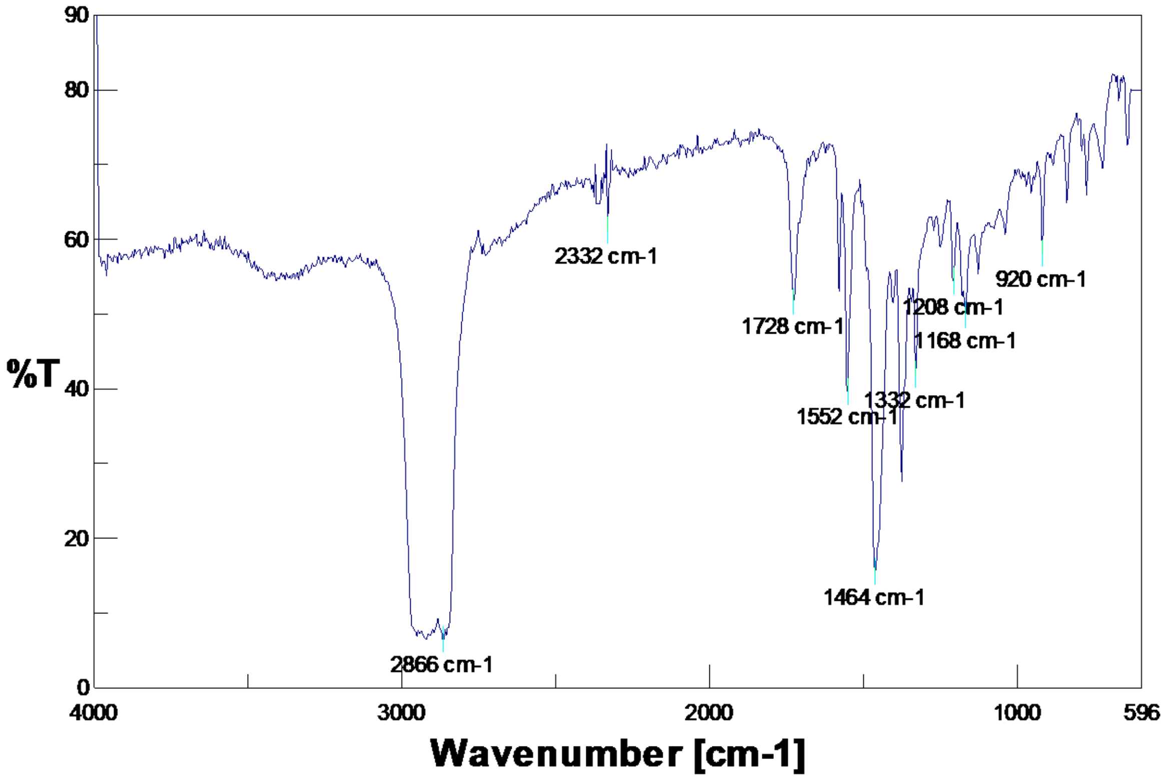

FTMS spectrum of **3b** (ESI-TOF)

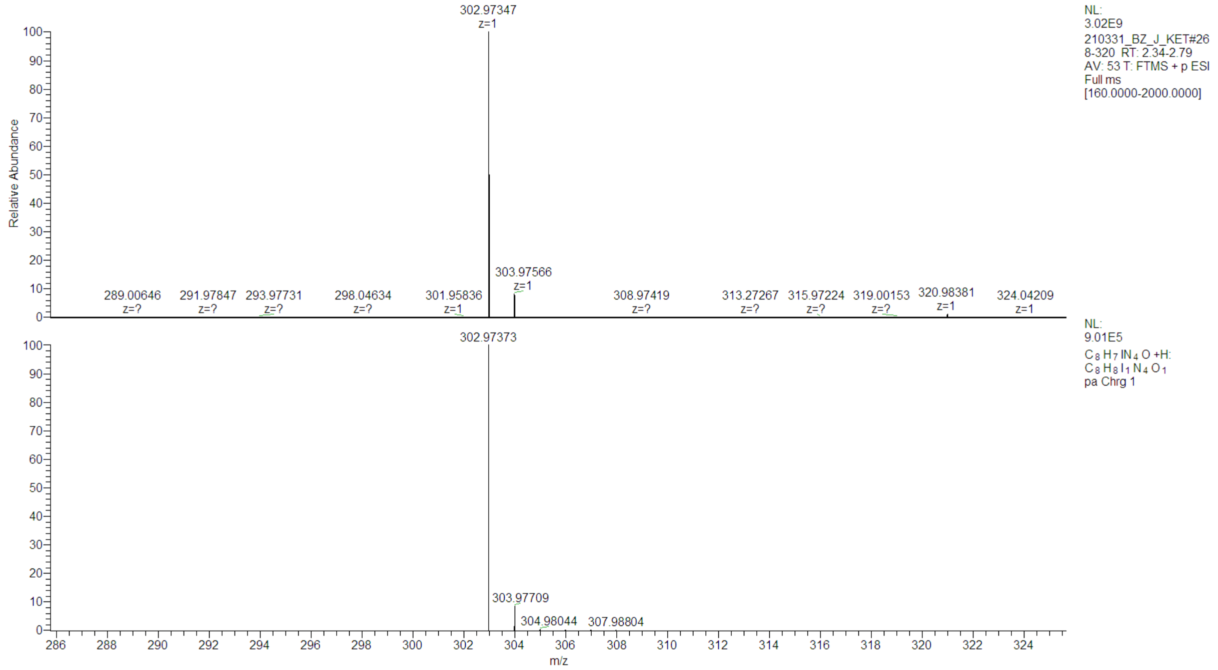

1-(6-Chloro-9H-purin-9-yl)propan-2-ol (rac-4a)

^{1}H NMR spectrum of *rac*-**4a** (500 MHz, $CDCl_3$)

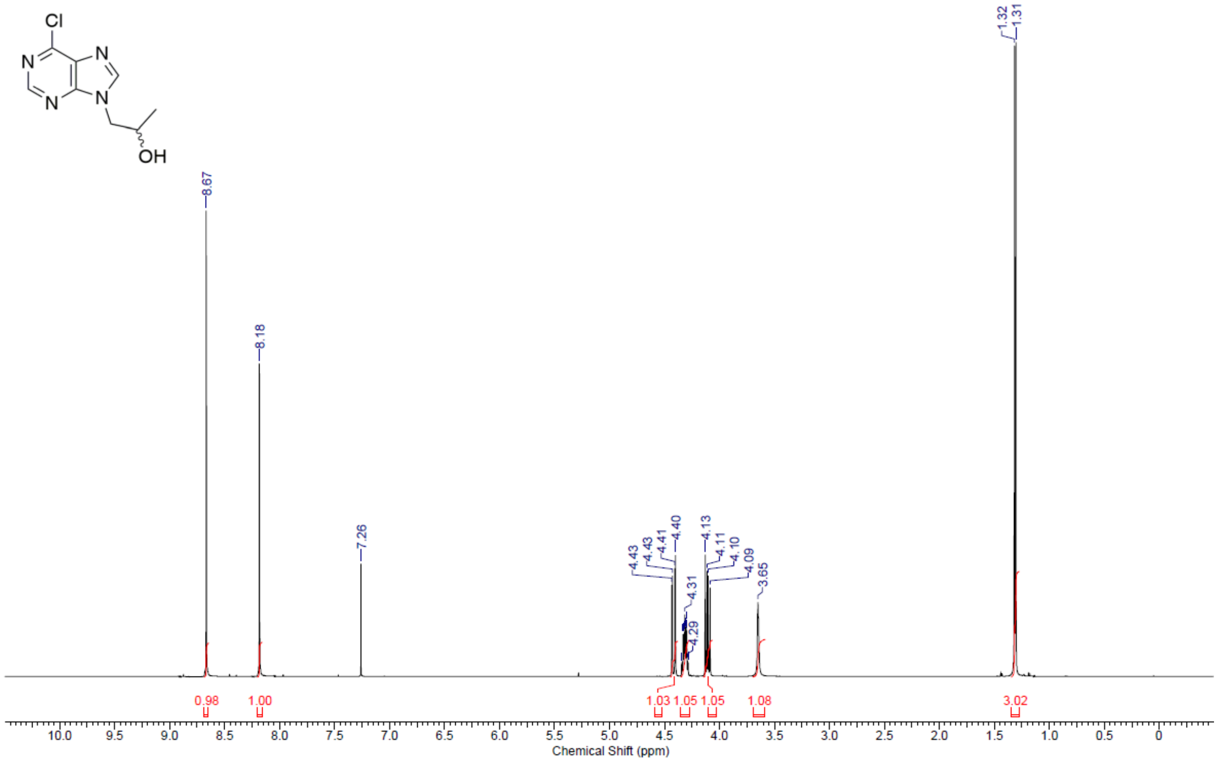

^{13}C{^{1}H} NMR spectrum of *rac*-**4a** (126 MHz, $CDCl_3$)

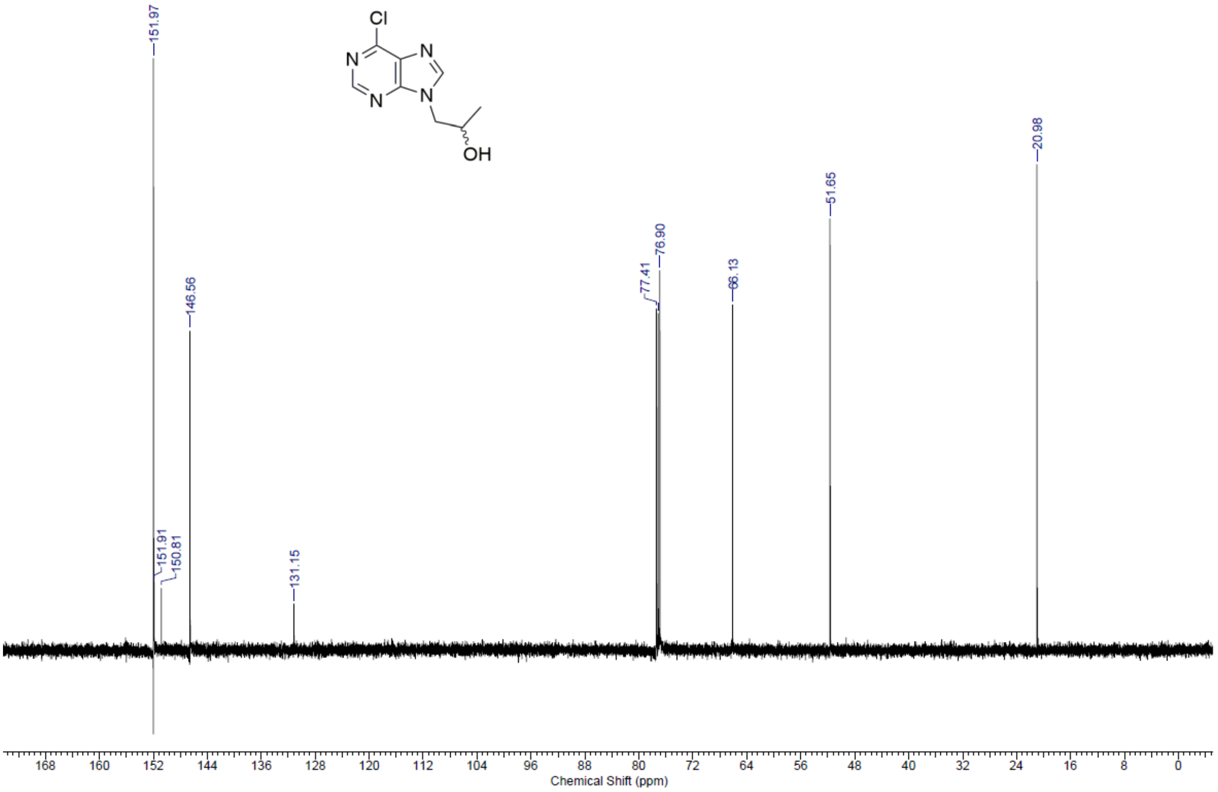

IR spectrum of *rac*-**4a** (Nujol)

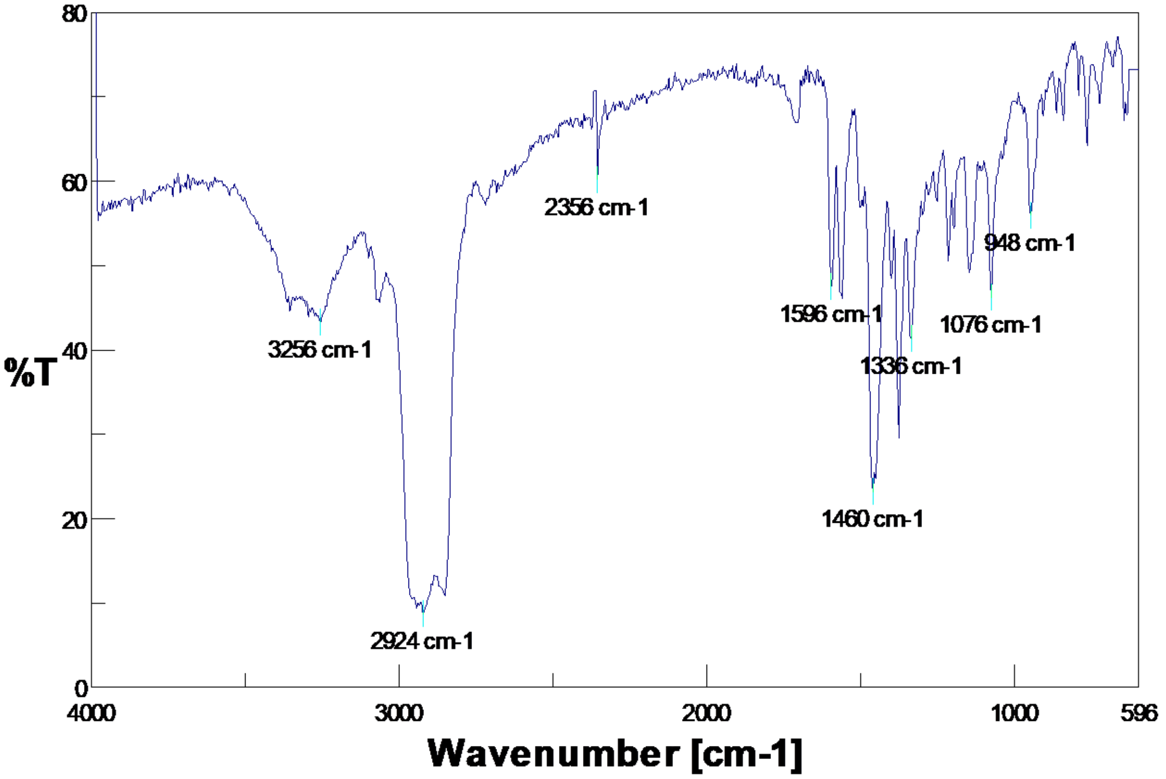

FTMS spectrum of *rac*-**4a** (ESI-TOF)

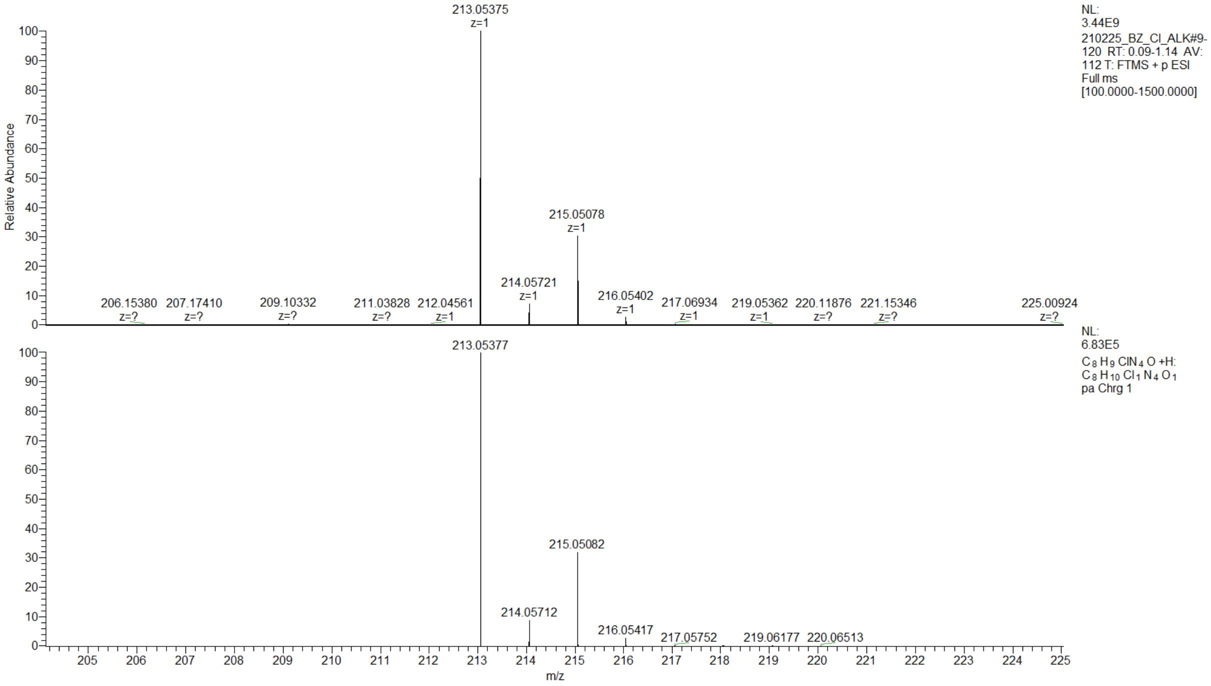

1-(6-Iodo-9H-purin-9-yl)propan-2-ol (rac-4b)

^{1}H NMR spectrum of *rac*-**4b** (500 MHz, $CDCl_3$)

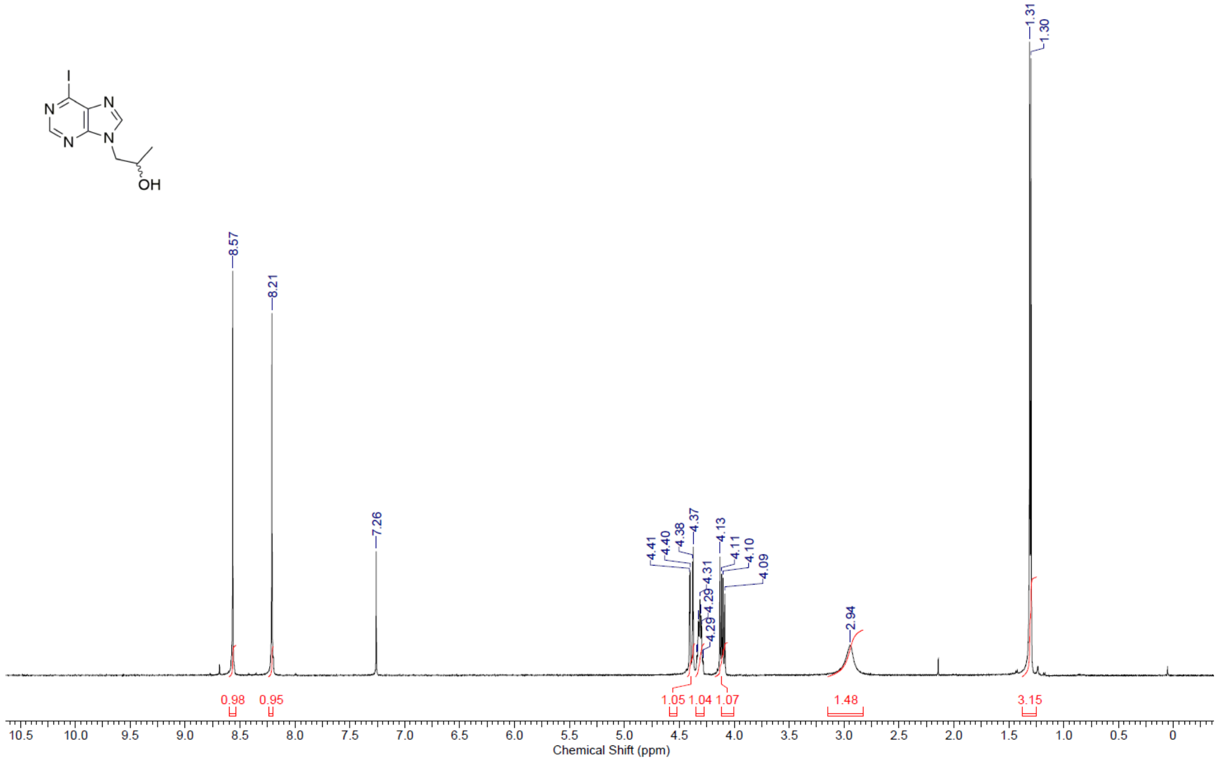

^{13}C{^{1}H} NMR spectrum of *rac*-**4b** (126 MHz, $CDCl_3$)

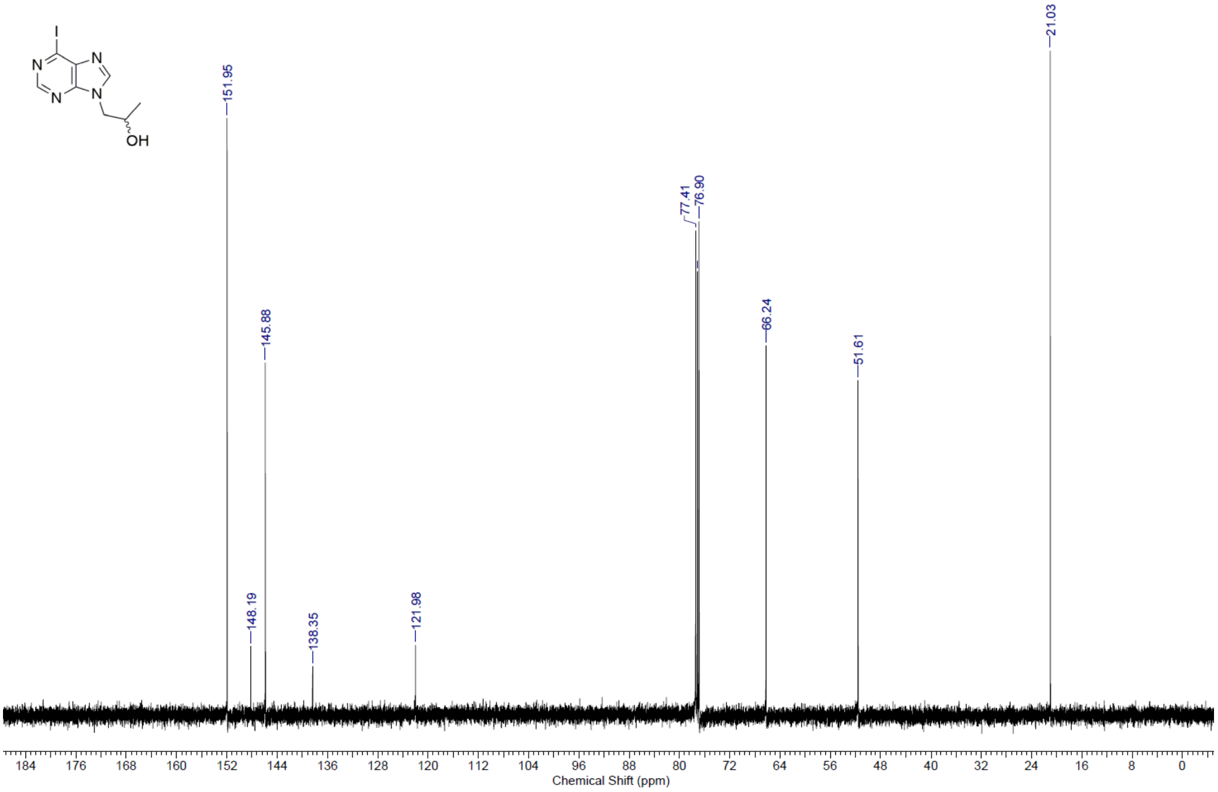

IR spectrum of *rac*-**4b** (Nujol)

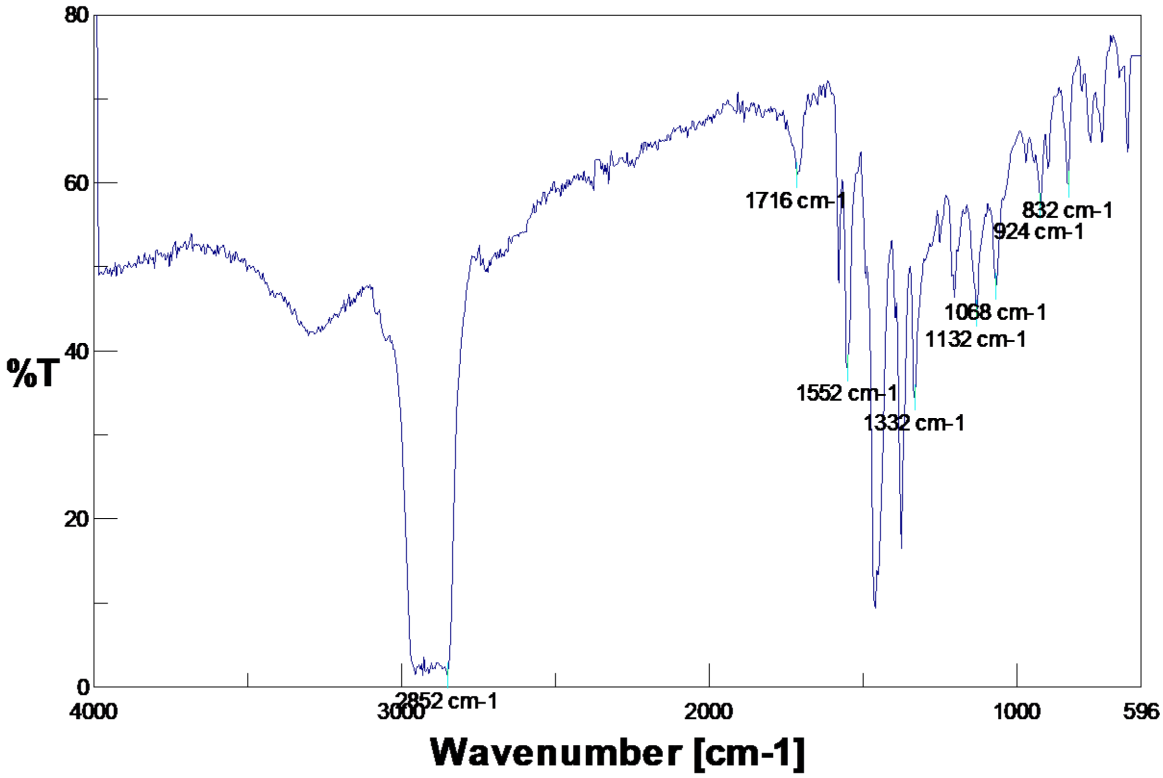

FTMS spectrum of *rac*-**4b** (ESI-TOF)

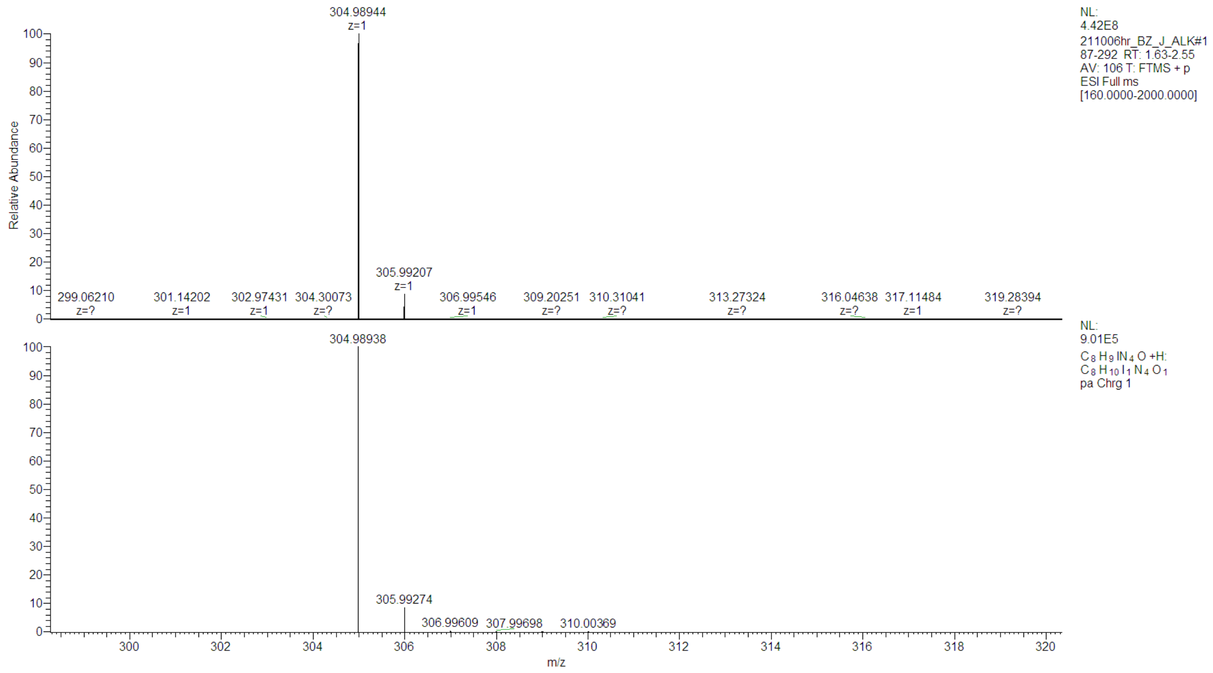

1-(6-Chloro-9H-purin-9-yl)propan-2-yl acetate (rac-5a)

^{1}H NMR spectrum of *rac*-**5a** (500 MHz, $CDCl_3$)

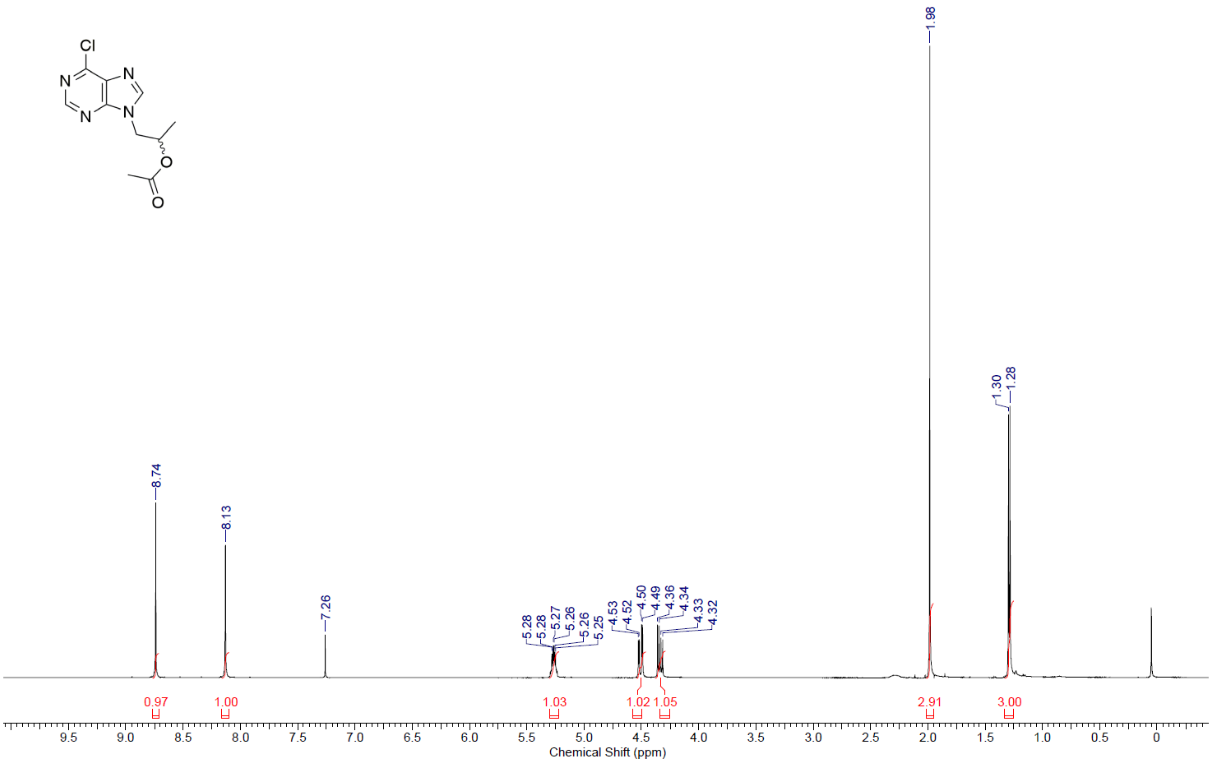

^{13}C{^{1}H} NMR spectrum of *rac*-**5a** (126 MHz, $CDCl_3$)

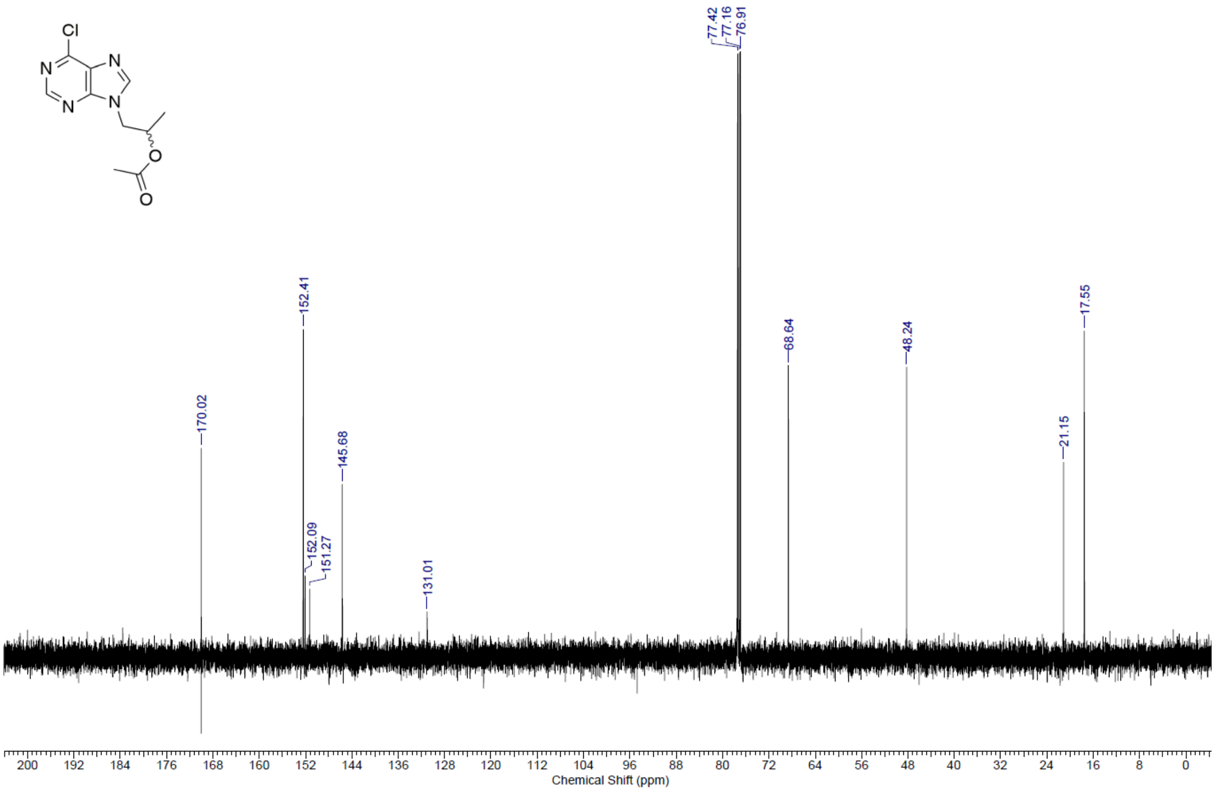

IR spectrum of *rac*-**5a** (Nujol)

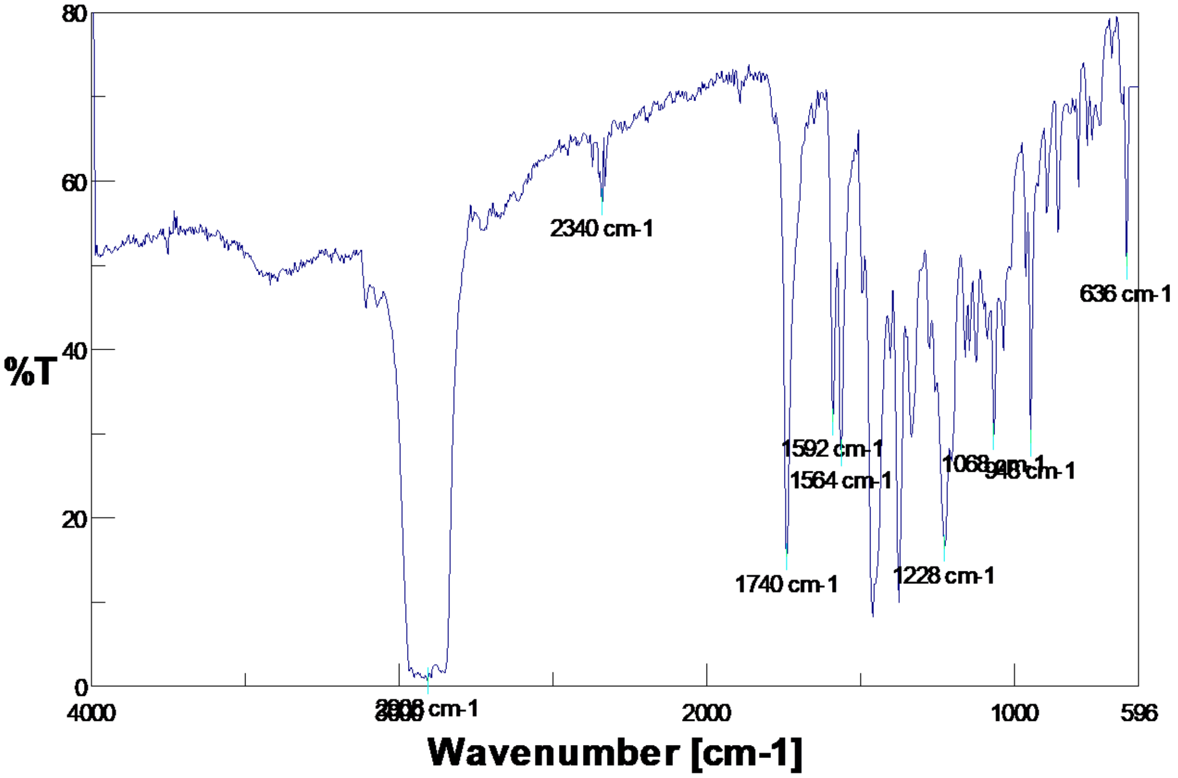

FTMS spectrum of *rac*-**5a** (ESI-TOF)

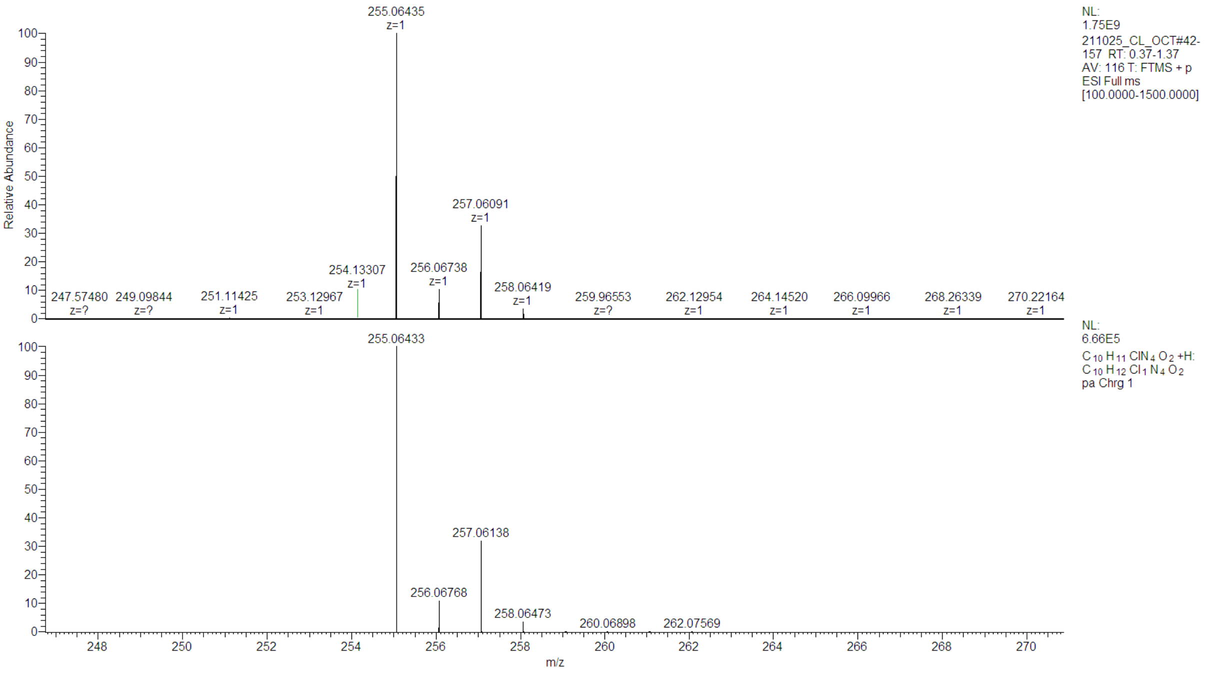

1-(6-Iodo-9H-purin-9-yl)propan-2-yl acetate (rac-5b)

^{1}H NMR spectrum of *rac*-**5b** (500 MHz, $CDCl_3$)

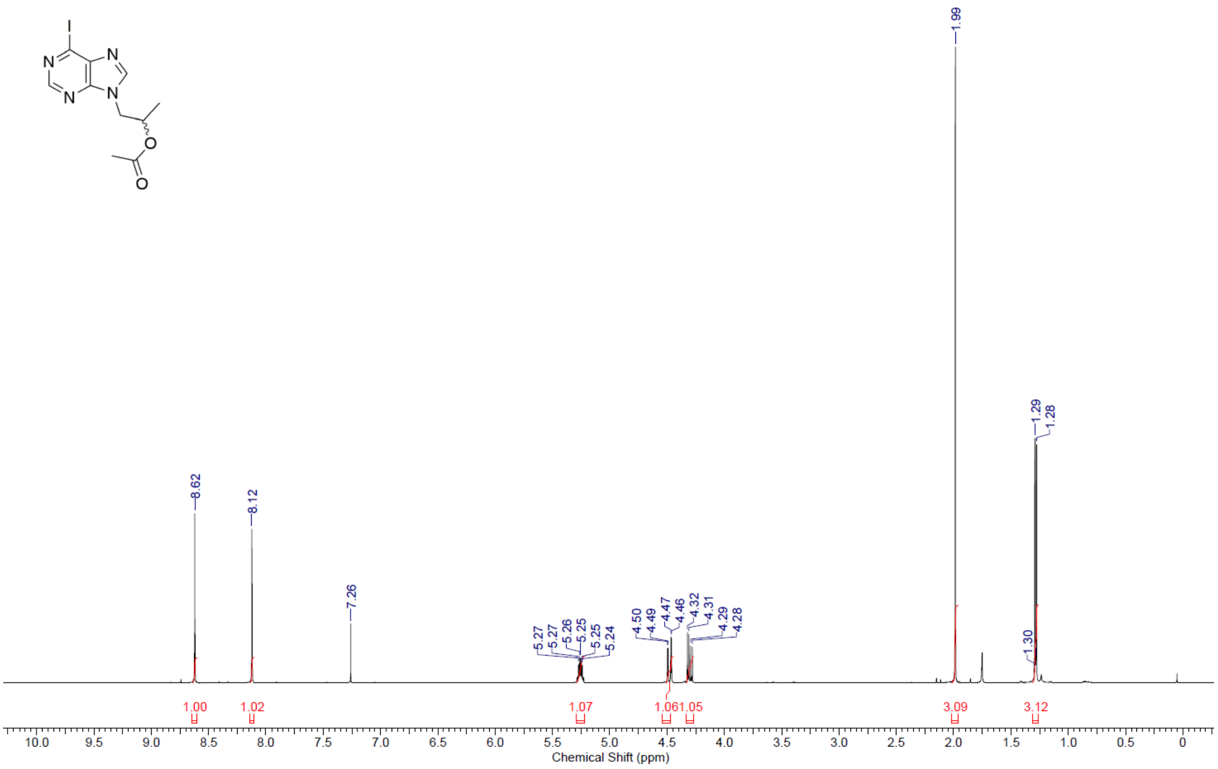

^{13}C{^{1}H} NMR spectrum of *rac*-**5b** (126 MHz, $CDCl_3$)

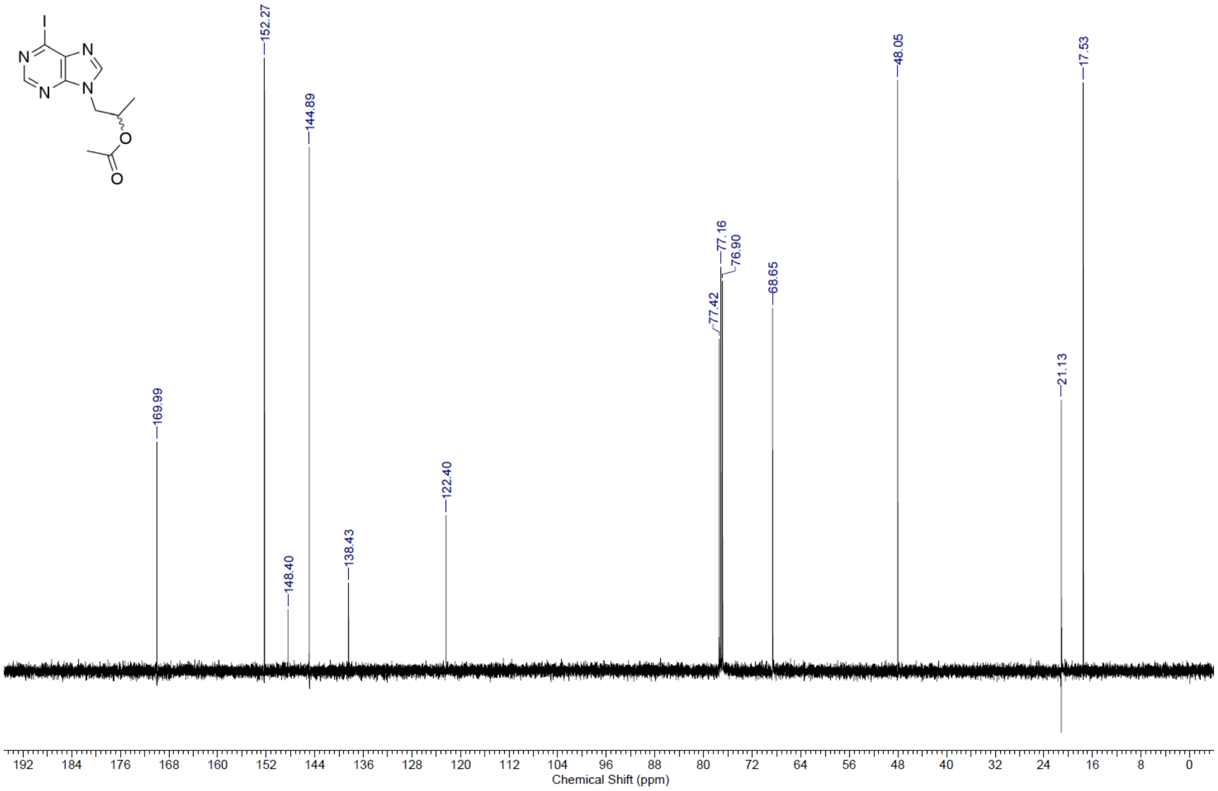

IR spectrum of *rac*-**5b** (Nujol)

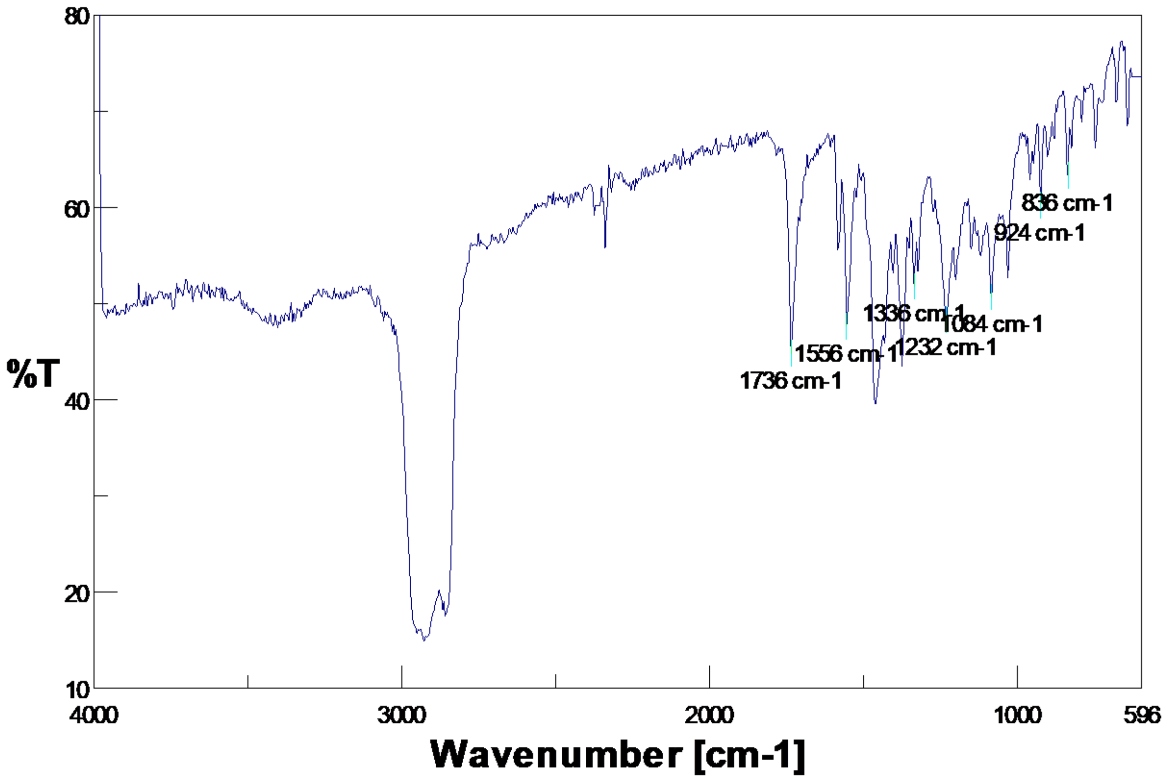

FTMS spectrum of *rac*-**5b** (ESI-TOF)

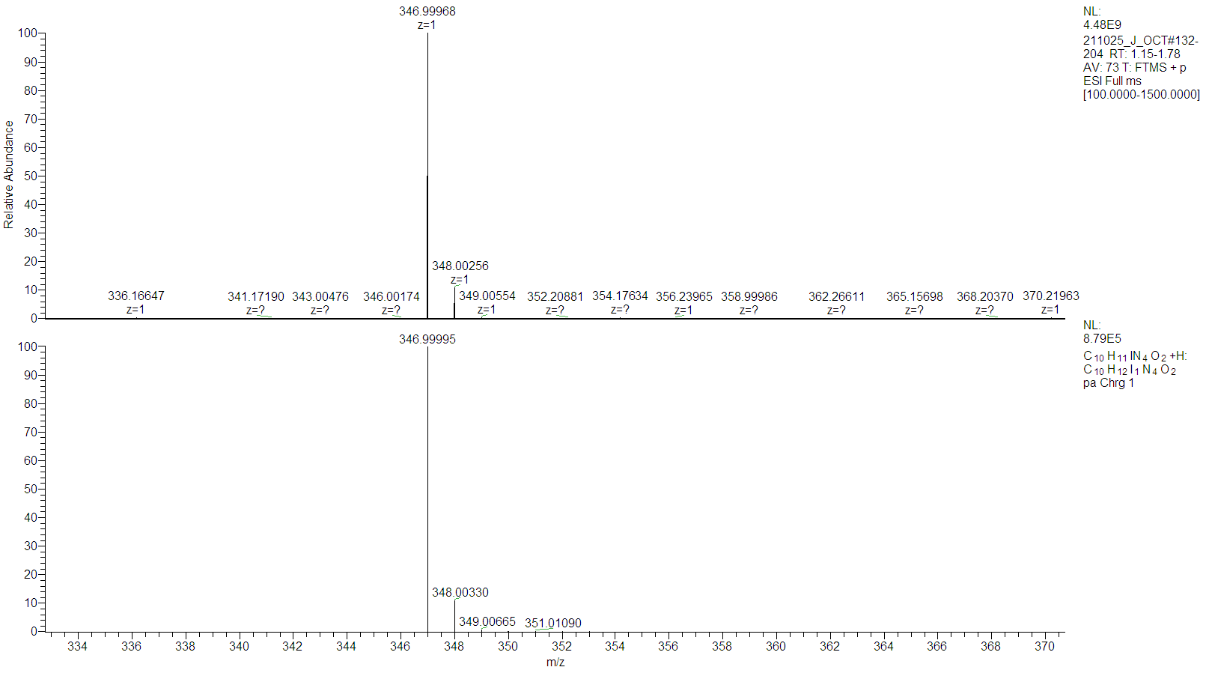

6-Chloro-9-{2-[(trimethylsilyl)oxy]propyl}-9H-purine (rac-6a)

^{1}H NMR spectrum of *rac*-**6a** (500 MHz, $CDCl_3$)

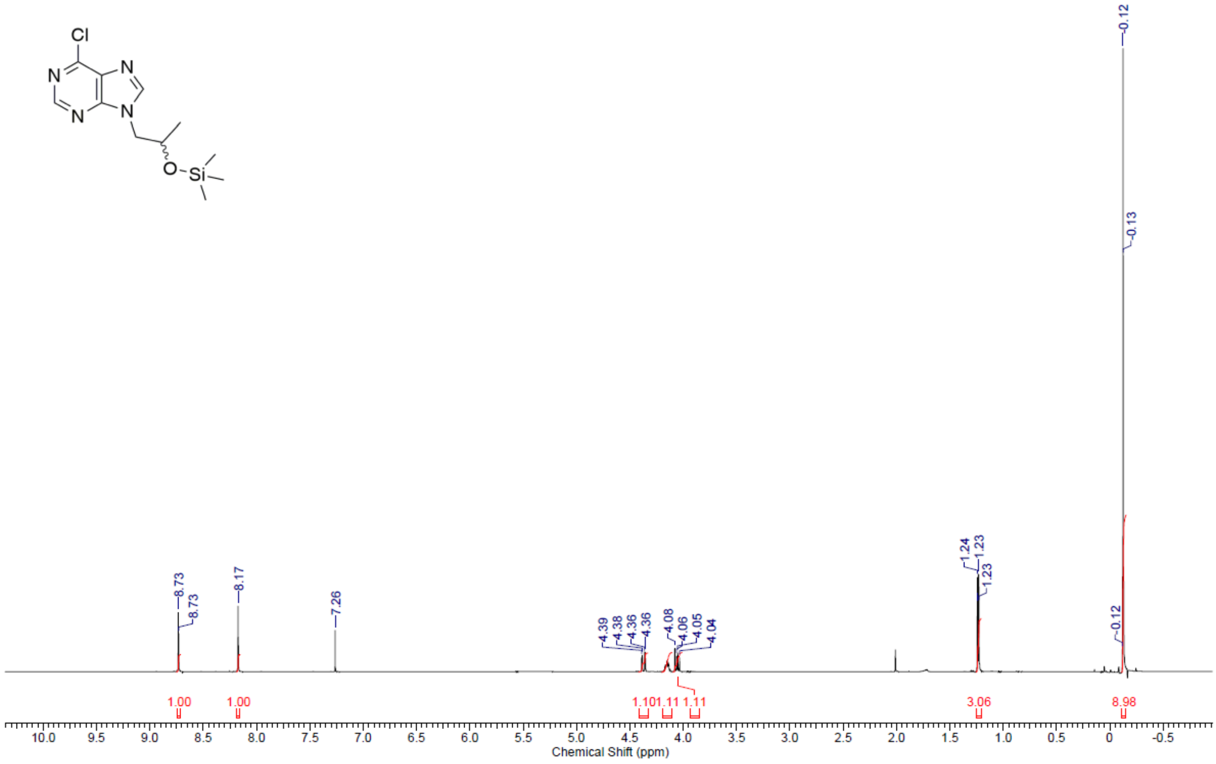

^{13}C{^{1}H} NMR spectrum of *rac*-**6a** (126 MHz, $CDCl_3$)

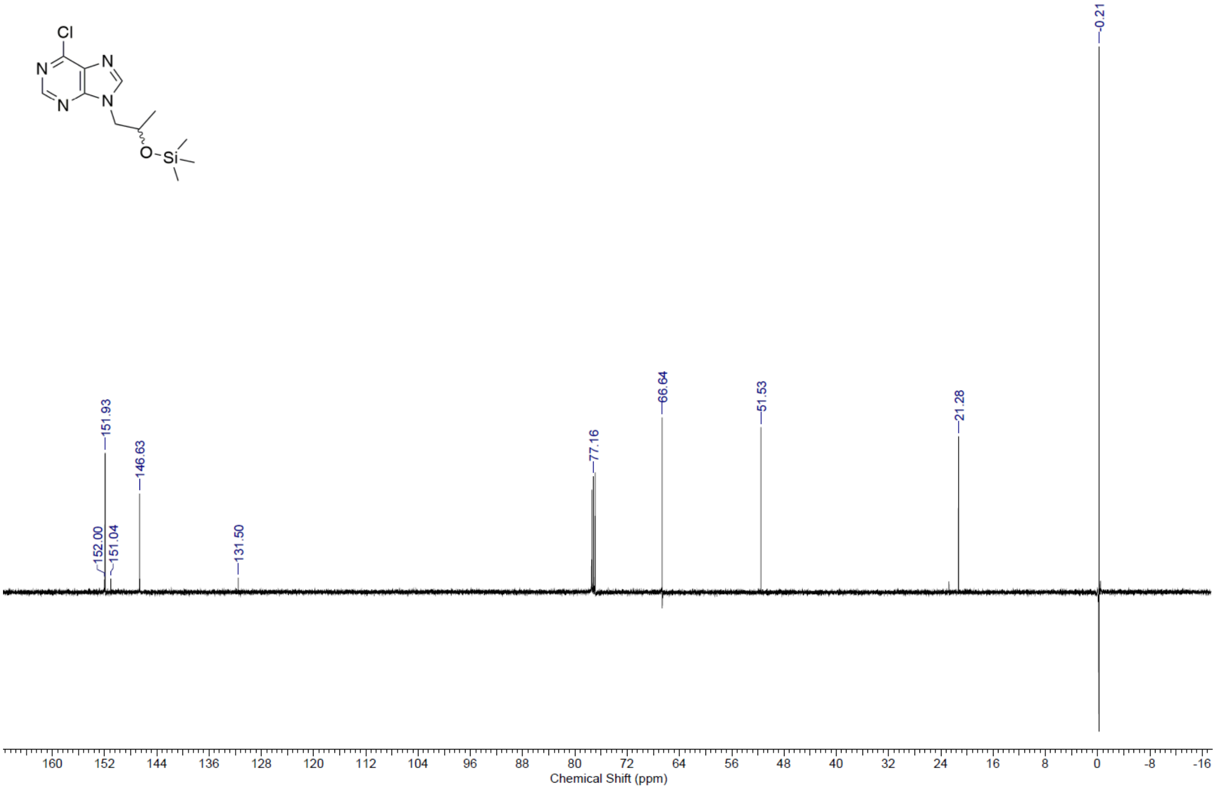

IR spectrum of *rac*-**6a** (Nujol)

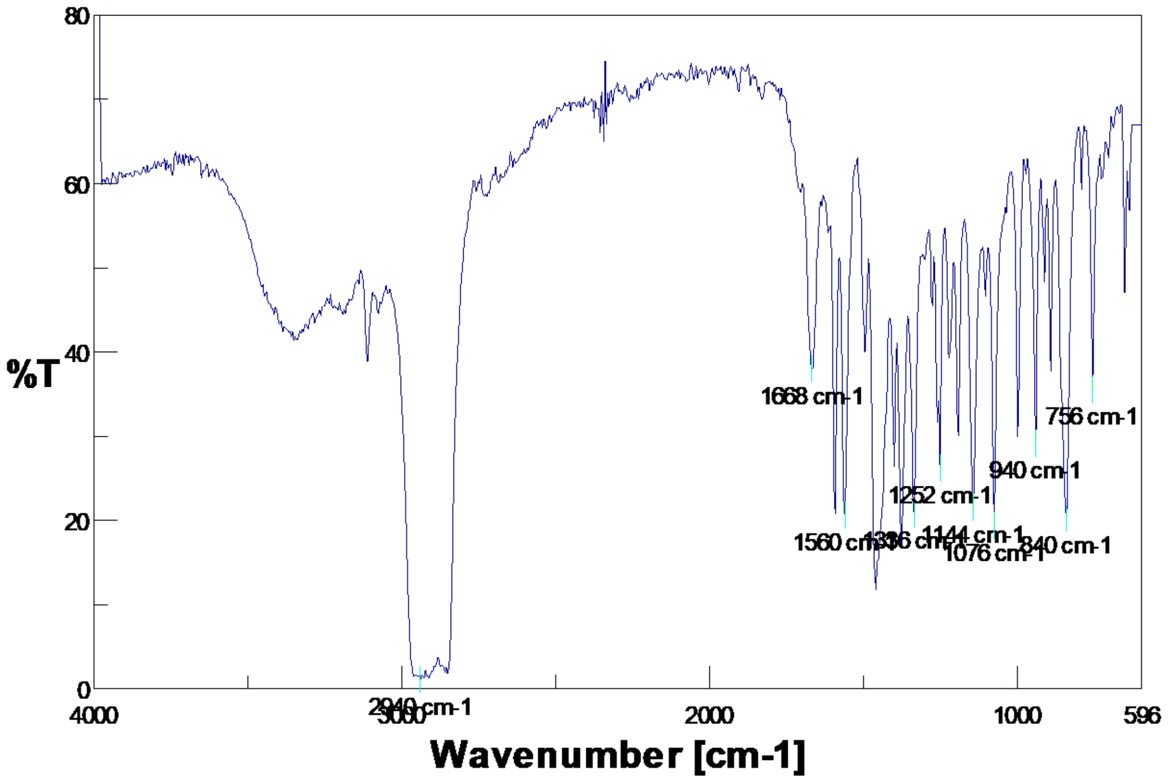

FTMS spectrum of *rac*-**6a** (ESI-TOF)

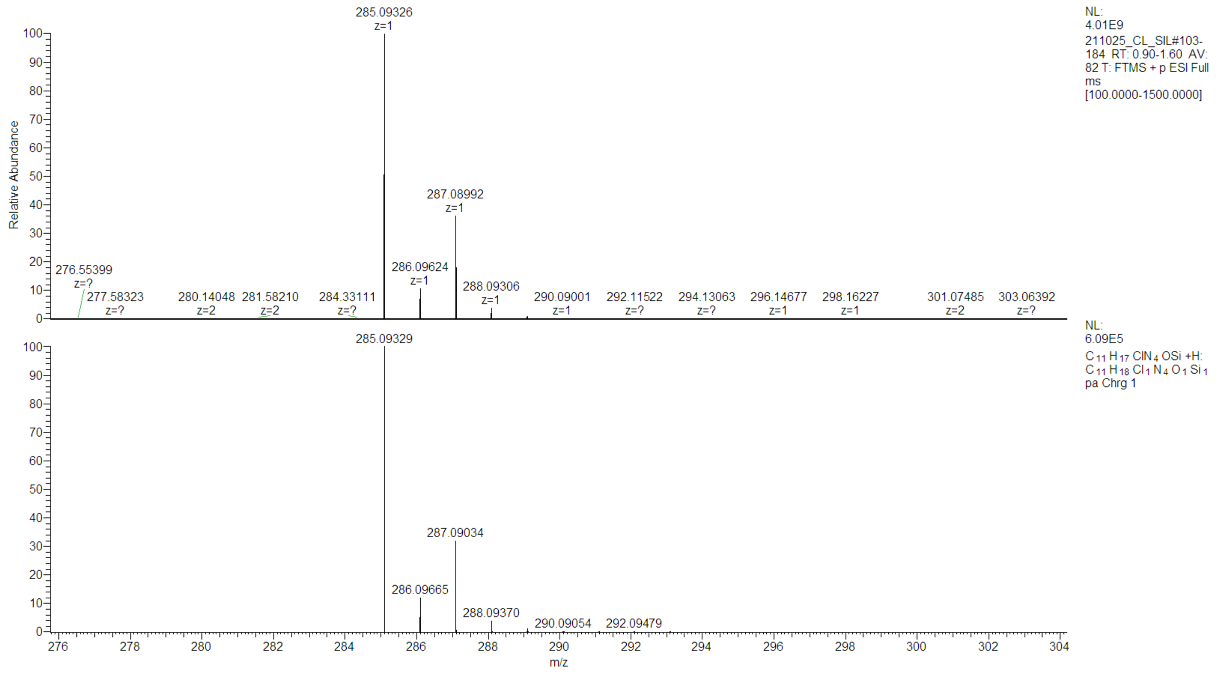

6-Iodo-9-{2-[(trimethylsilyl)oxy]propyl}-9H-purine (rac-6b)

^{1}H NMR spectrum of *rac*-**6b** (500 MHz, $CDCl_3$)

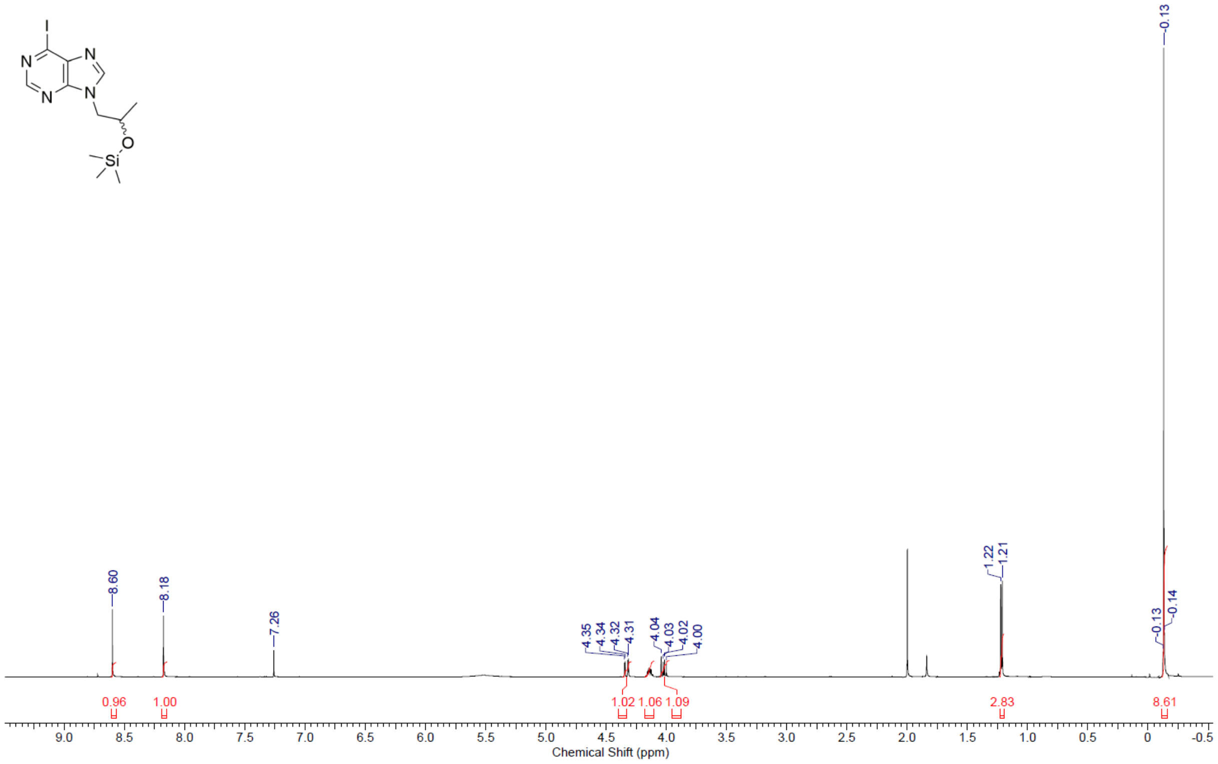

^{13}C{^{1}H} NMR spectrum of *rac*-**6b** (126 MHz, $CDCl_3$)

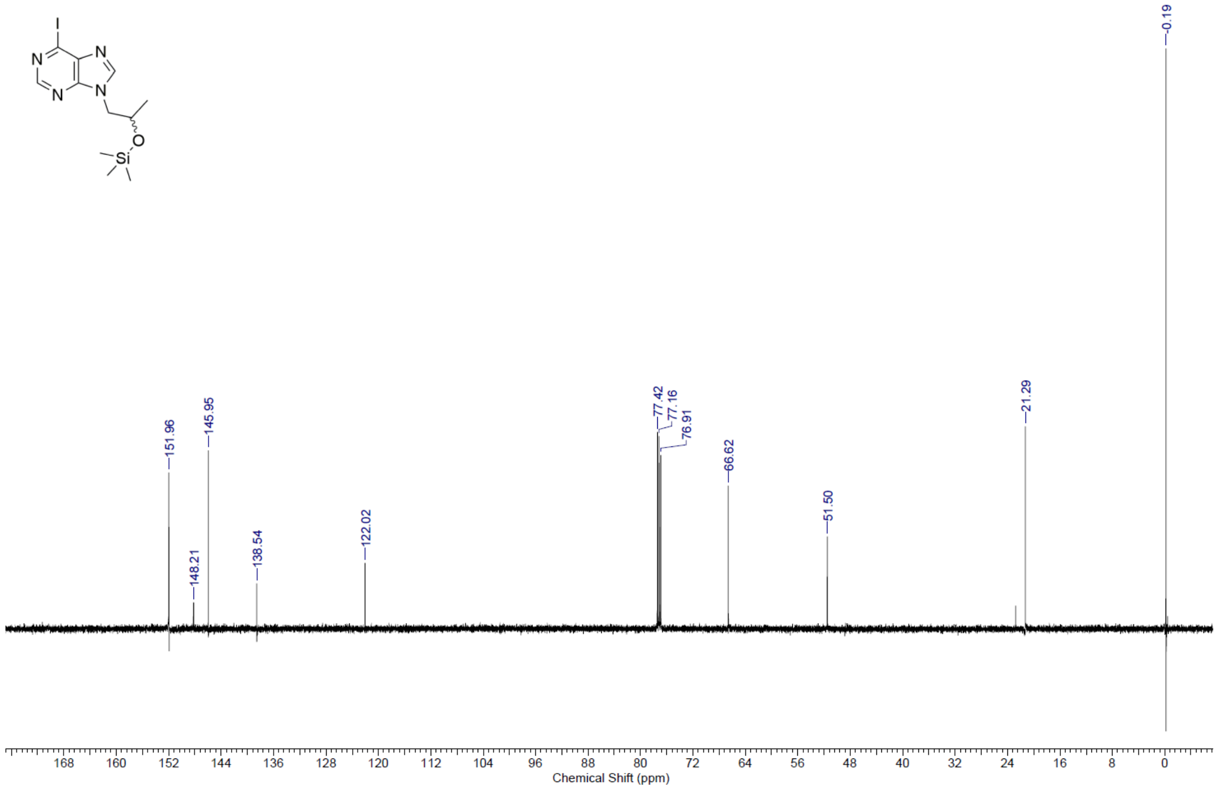

IR spectrum of *rac*-**6b** (Nujol)

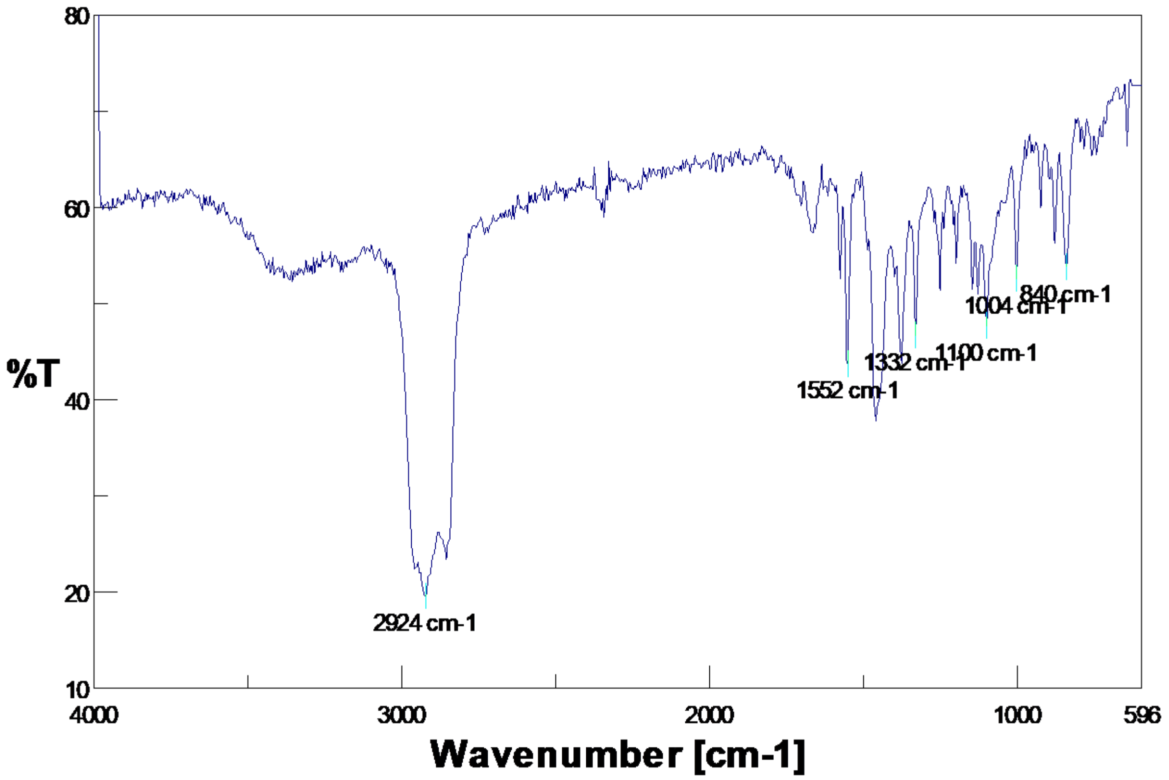

FTMS spectrum of *rac*-**6b** (ESI-TOF)

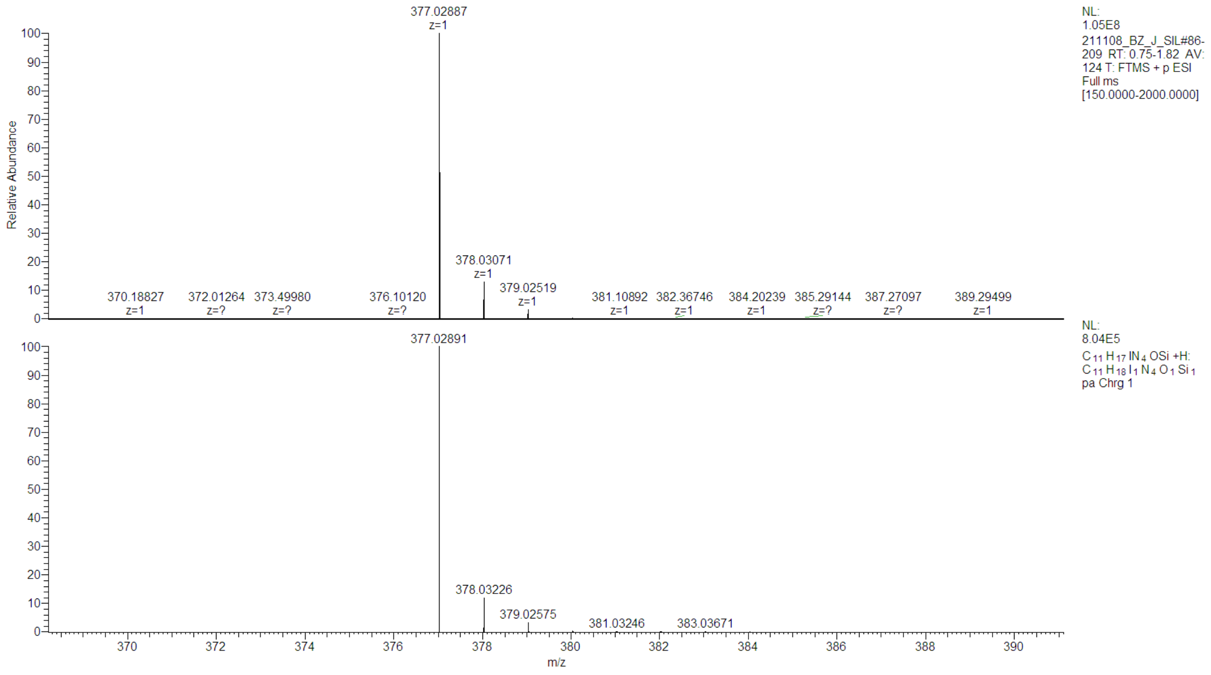

(2R)-1-(6-Amino-9H-purin-9-yl)propan-2-ol [(R)-(–)-7]

^{1}H NMR spectrum of (*R*)-(–)-**7** (500 MHz, DMSO-d_6)

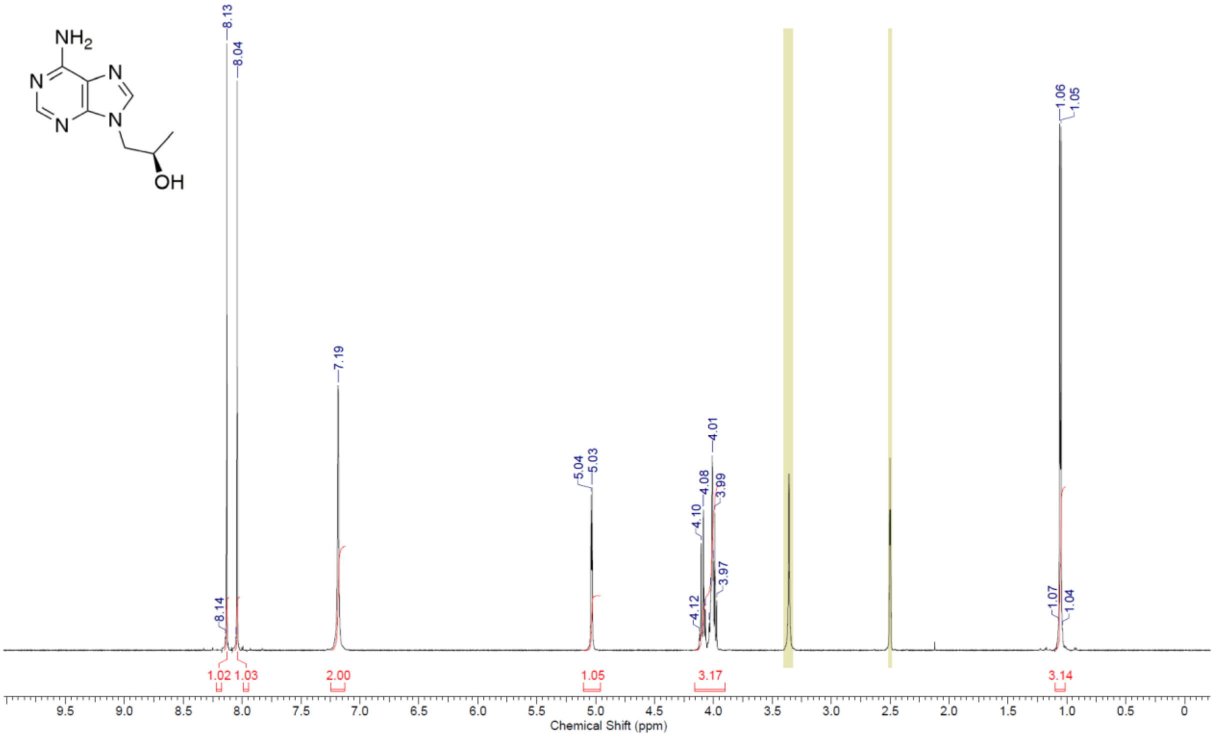

^{13}C{^{1}H} NMR spectrum of (*R*)-(–)-**7** (126 MHz, DMSO-d_6)

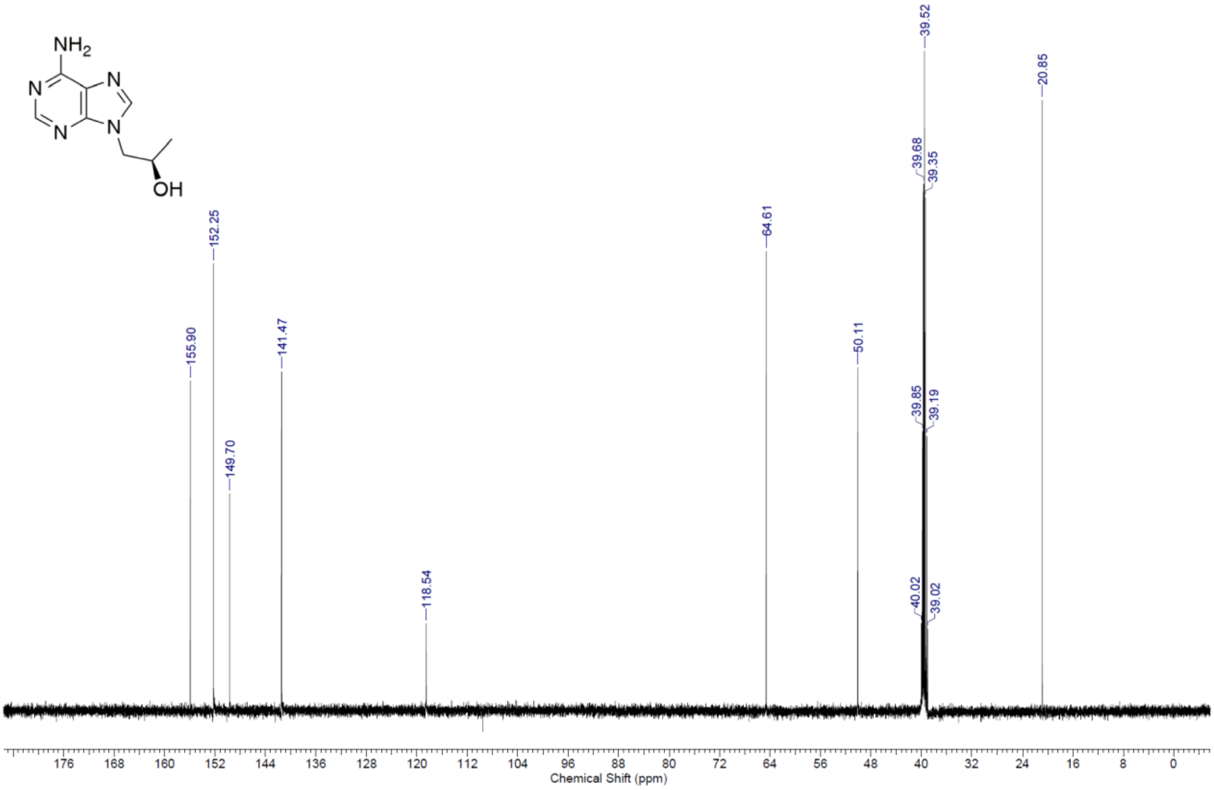

IR spectrum of (*R*)-(–)-**7** (Nujol)

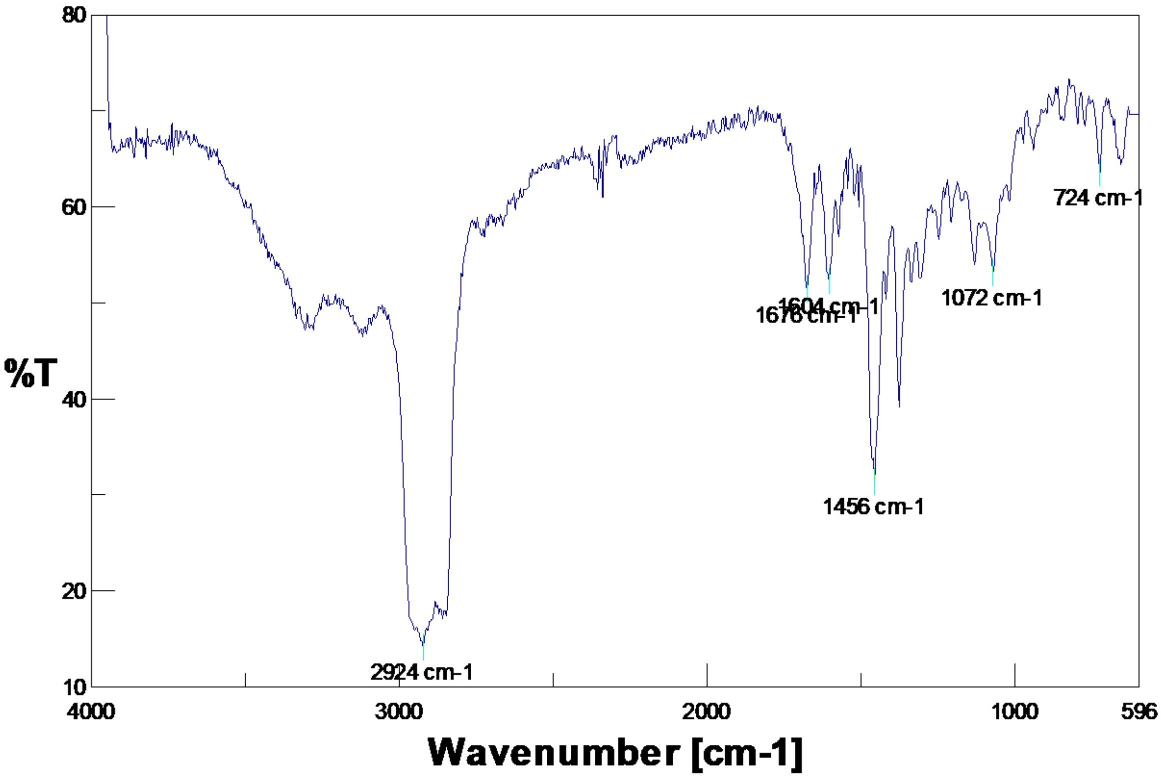

FTMS spectrum of (*R*)-(–)-**7** (ESI-TOF)

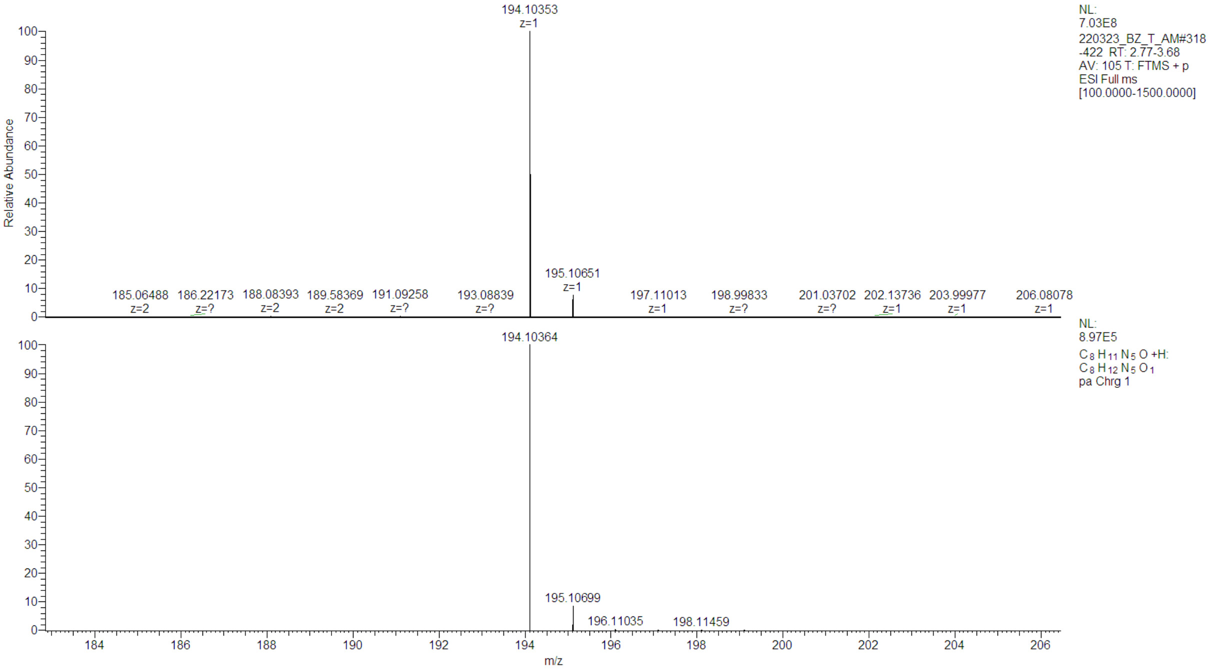

(Diethoxyphosphoryl)methyl 4-methylbenzenesulfonate (9)

^{1}H NMR spectrum of **9** (500 MHz, DMSO-d_6)

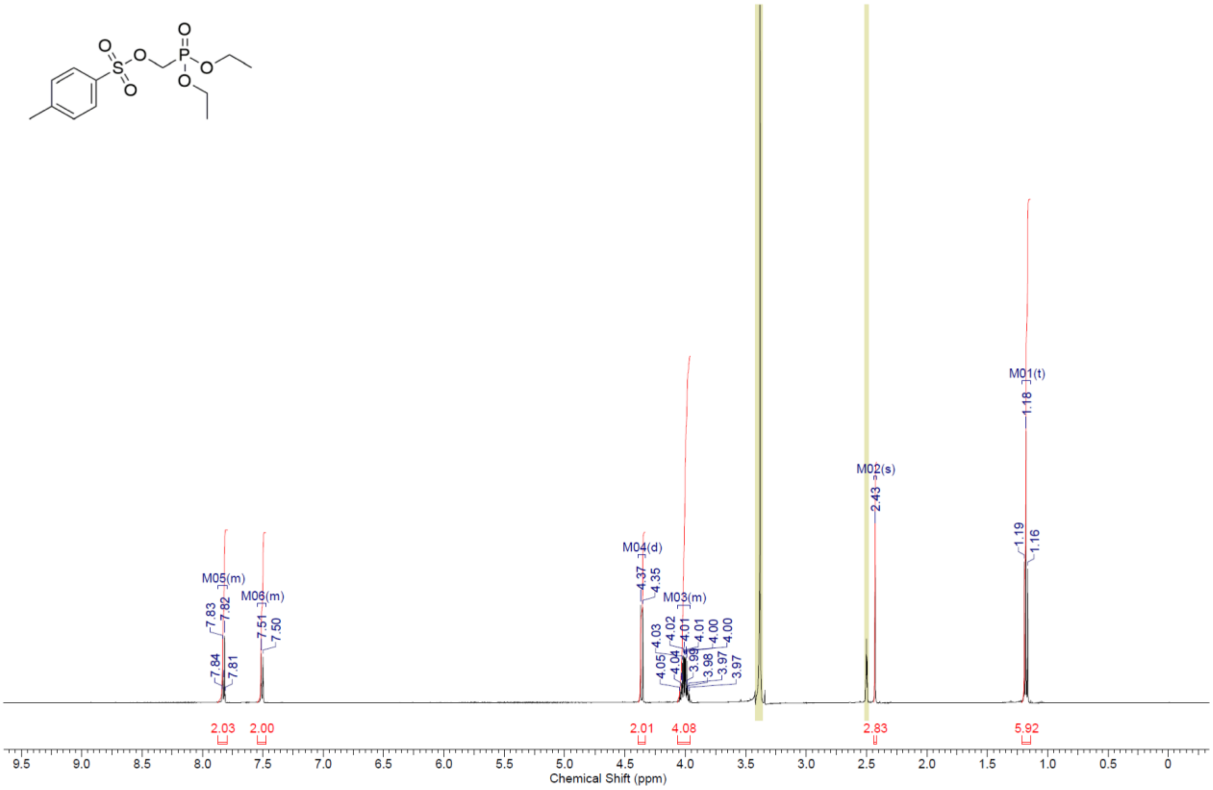

^{13}C{^{1}H} NMR spectrum of **9** (126 MHz, DMSO-d_6)

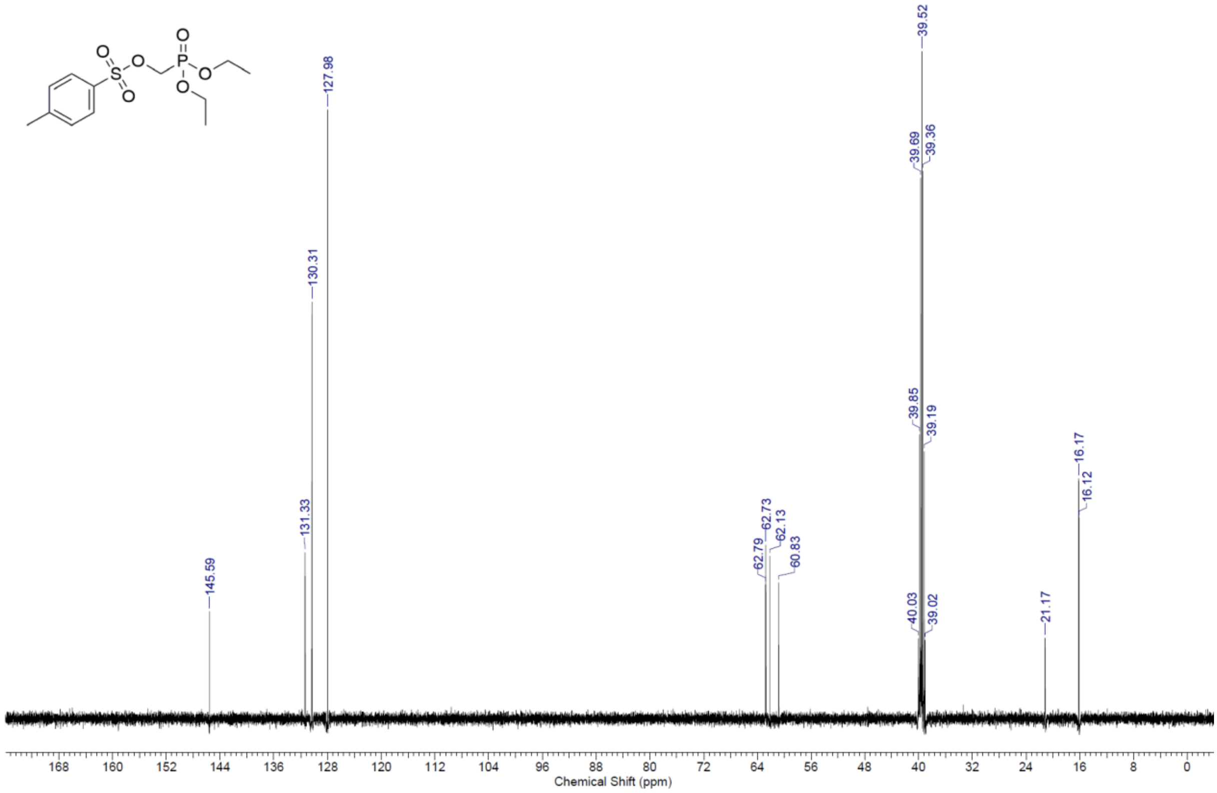

$^{31}P\{^{1}H\}$ spectrum of **9** (202 MHz, DMSO-d_6)

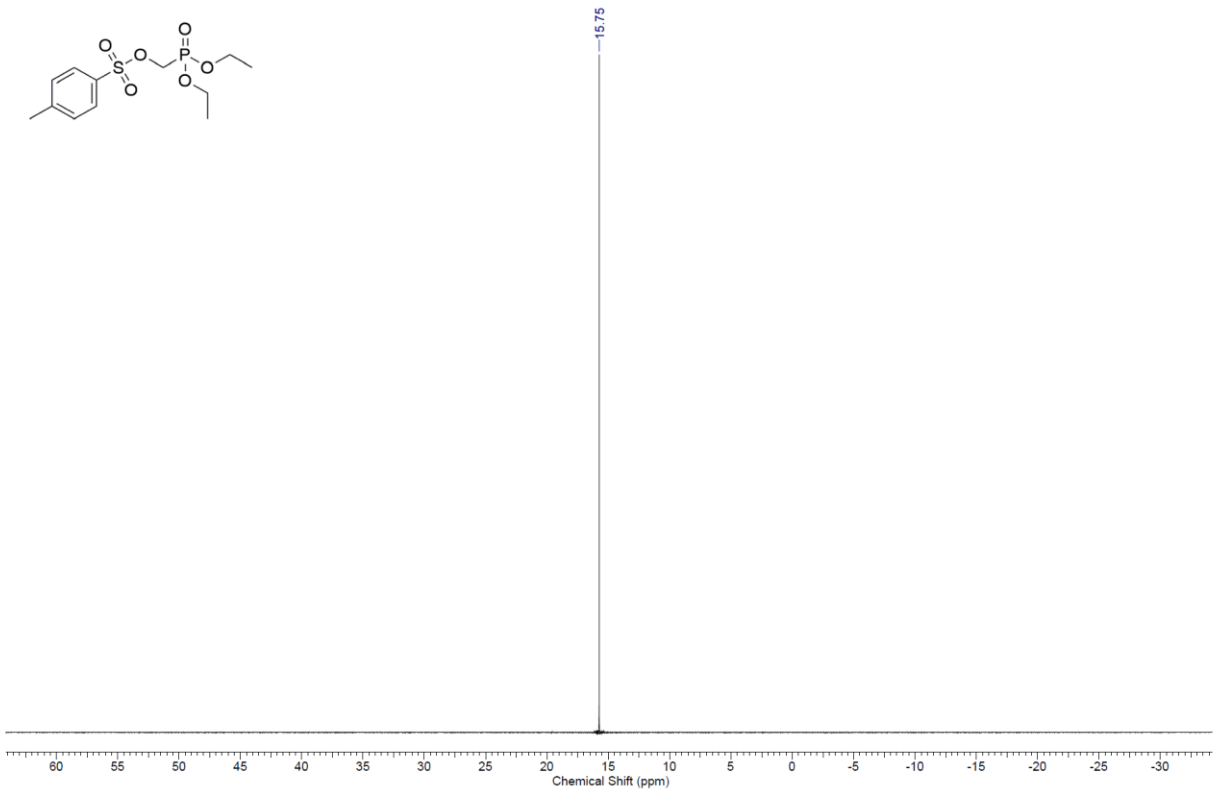

IR spectrum of **9** (Nujol)

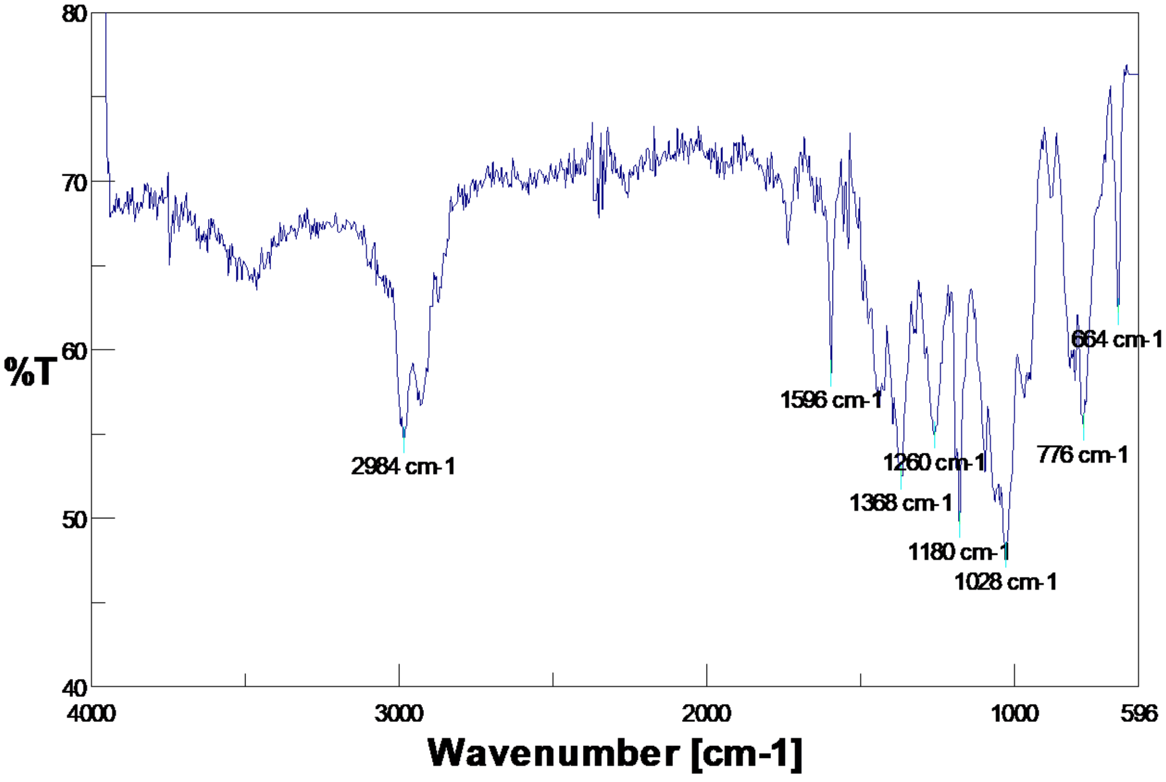

FTMS spectrum of **9** (ESI-TOF)

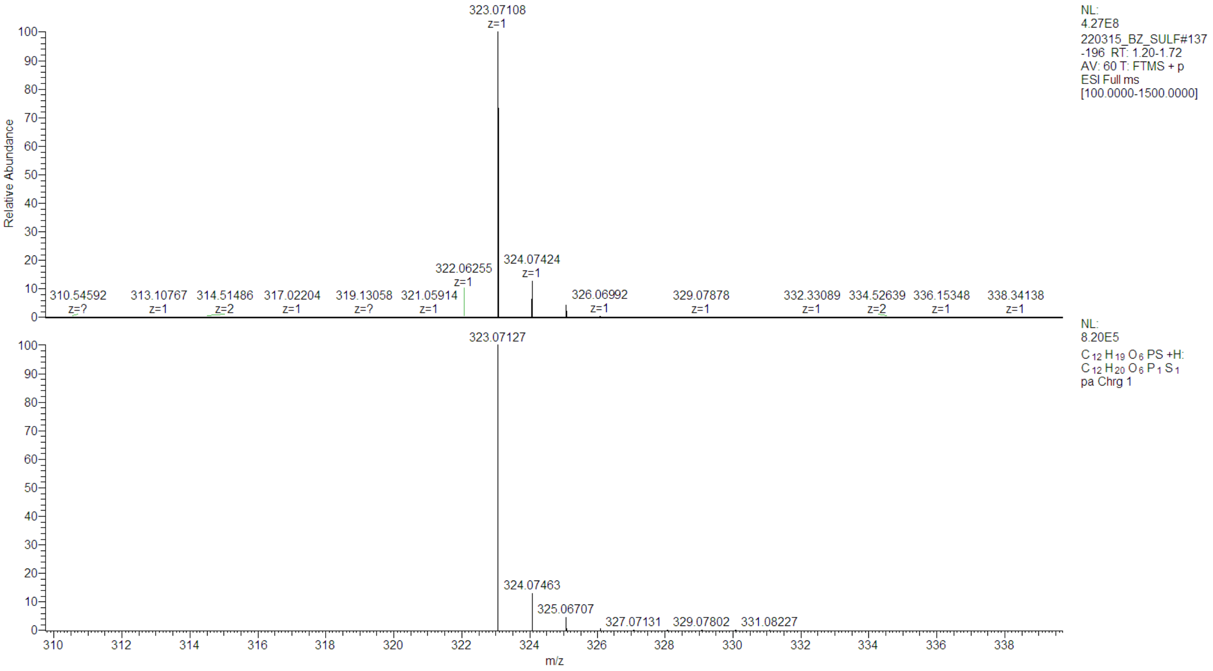

Diethyl ({[(2R)-1-(6-amino-9H-purin-9-yl)propan-2-yl]oxy}methyl)phosphonate [(R)-(–)-10]

^{1}H NMR spectrum of (*R*)-(–)-**10** (500 MHz, DMSO-d_6)

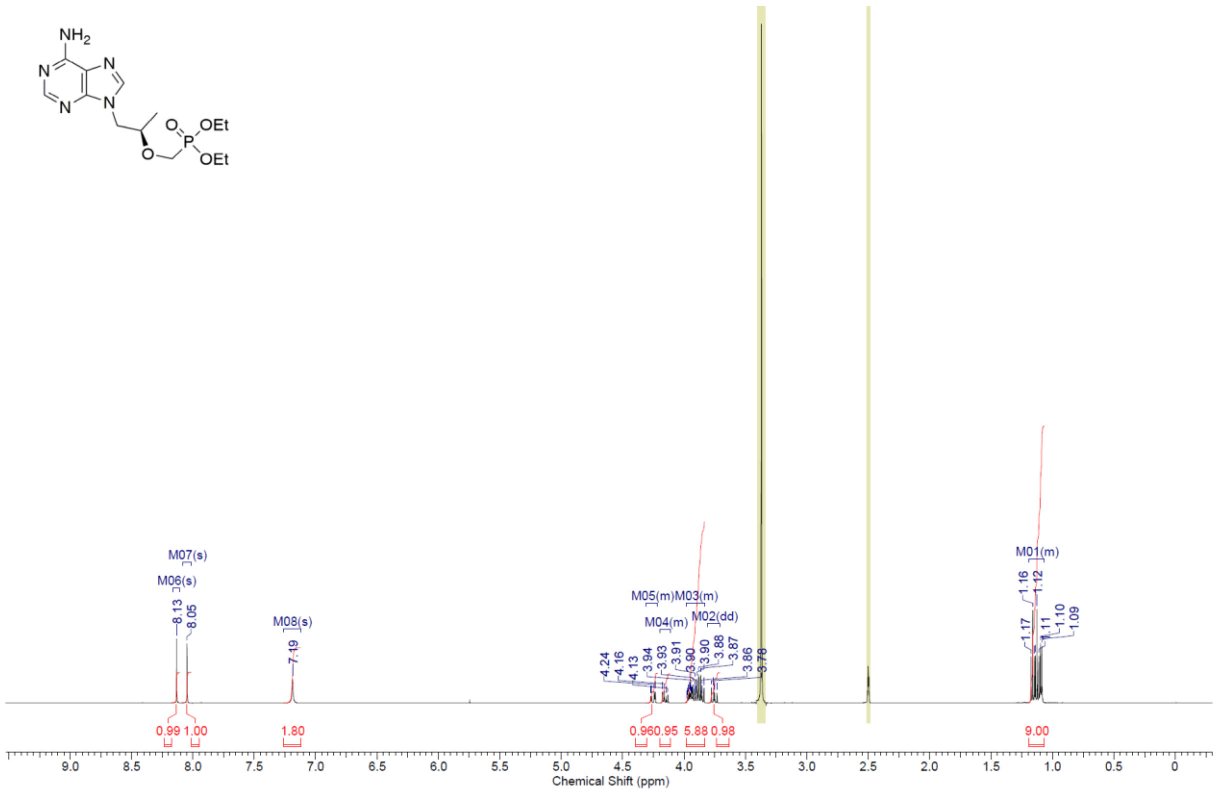

^{13}C{^{1}H} NMR spectrum of (*R*)-(–)-**10** (126 MHz, DMSO-d_6)

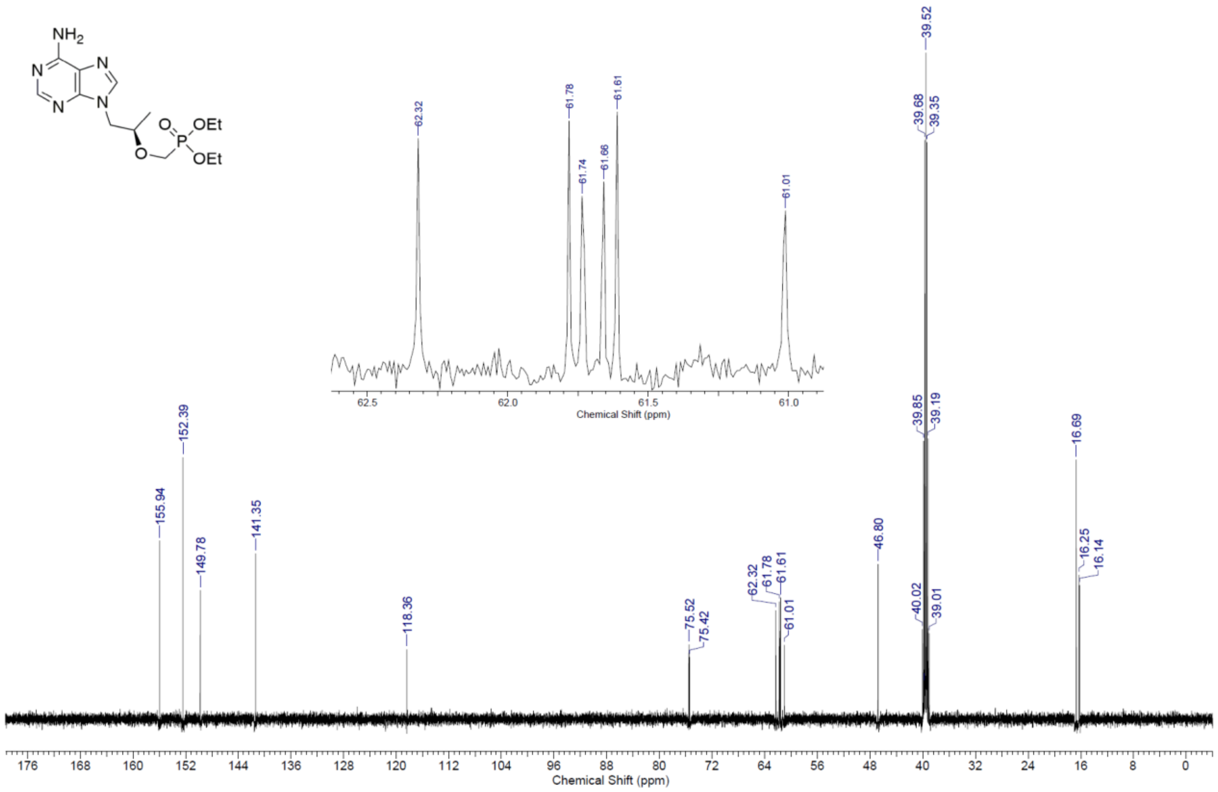

$^{31}P\{^{1}H\}$ spectrum of (*R*)-(–)-**10** (202 MHz, DMSO-*d*6)

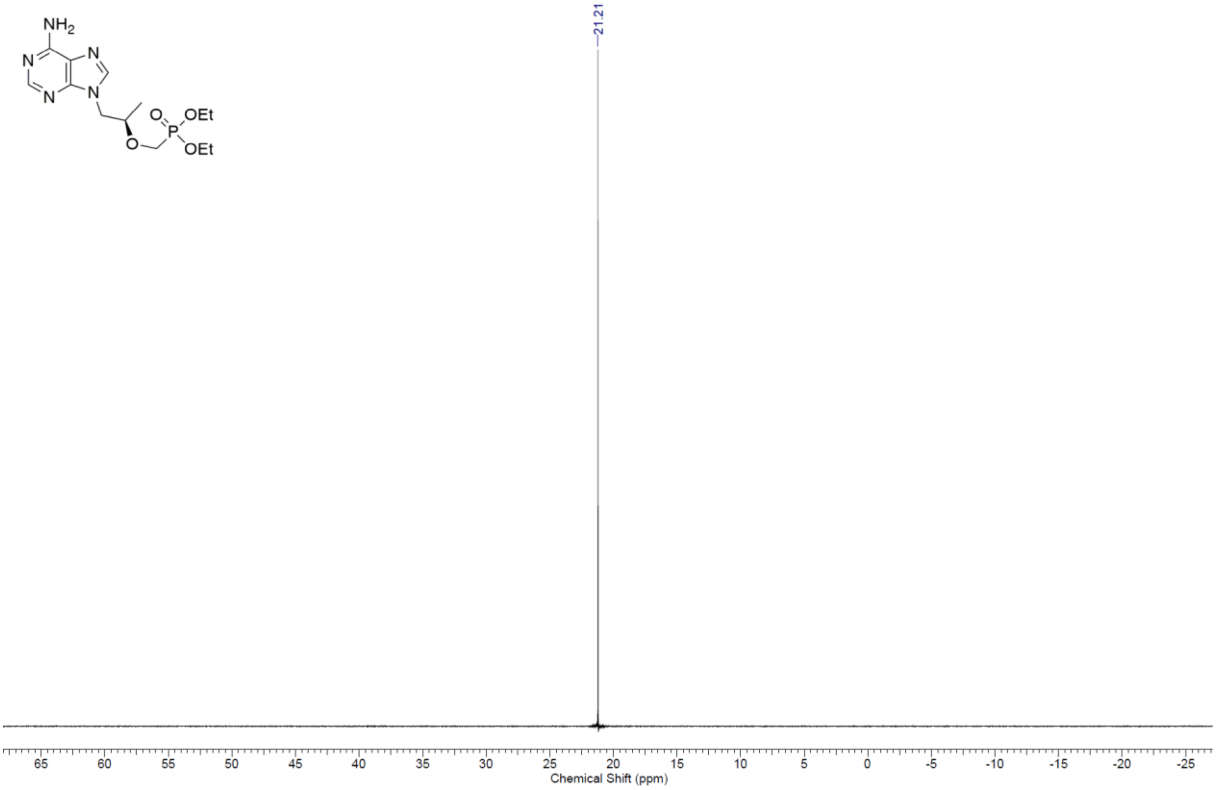

IR spectrum of (*R*)-(–)-**10** (Nujol)

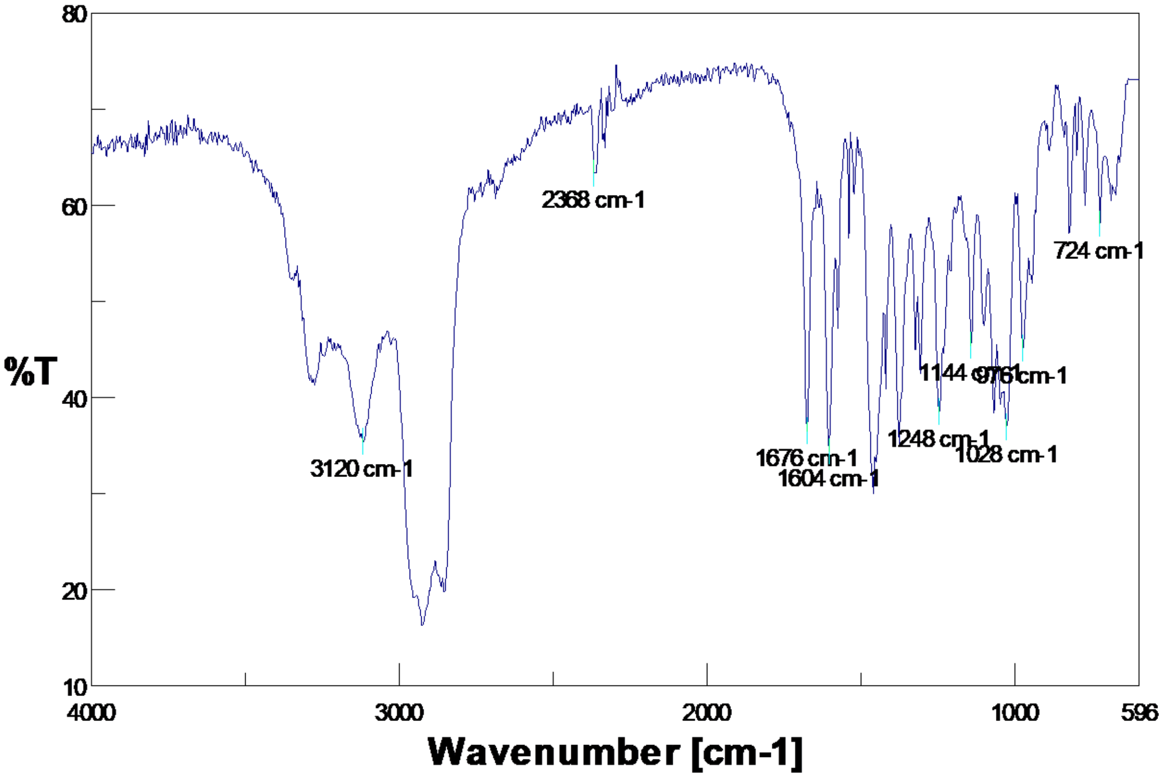

FTMS spectrum of (*R*)-(–)-**10** (ESI-TOF)

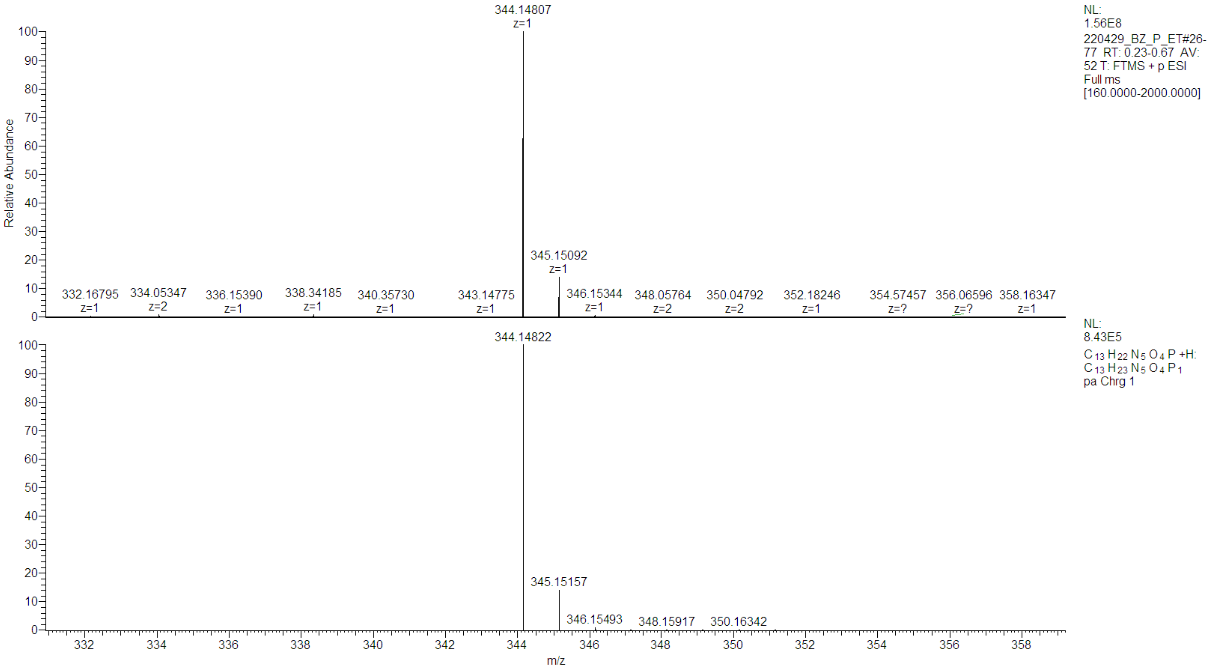

({[(2R)-1-(6-Amino-9H-purin-9-yl)propan-2-yl]oxy}methyl)phosphonic acid [(R)-(–)-11, tenofovir]

^{1}H NMR spectrum of (*R*)-(–)-**11** (500 MHz, D_2O)

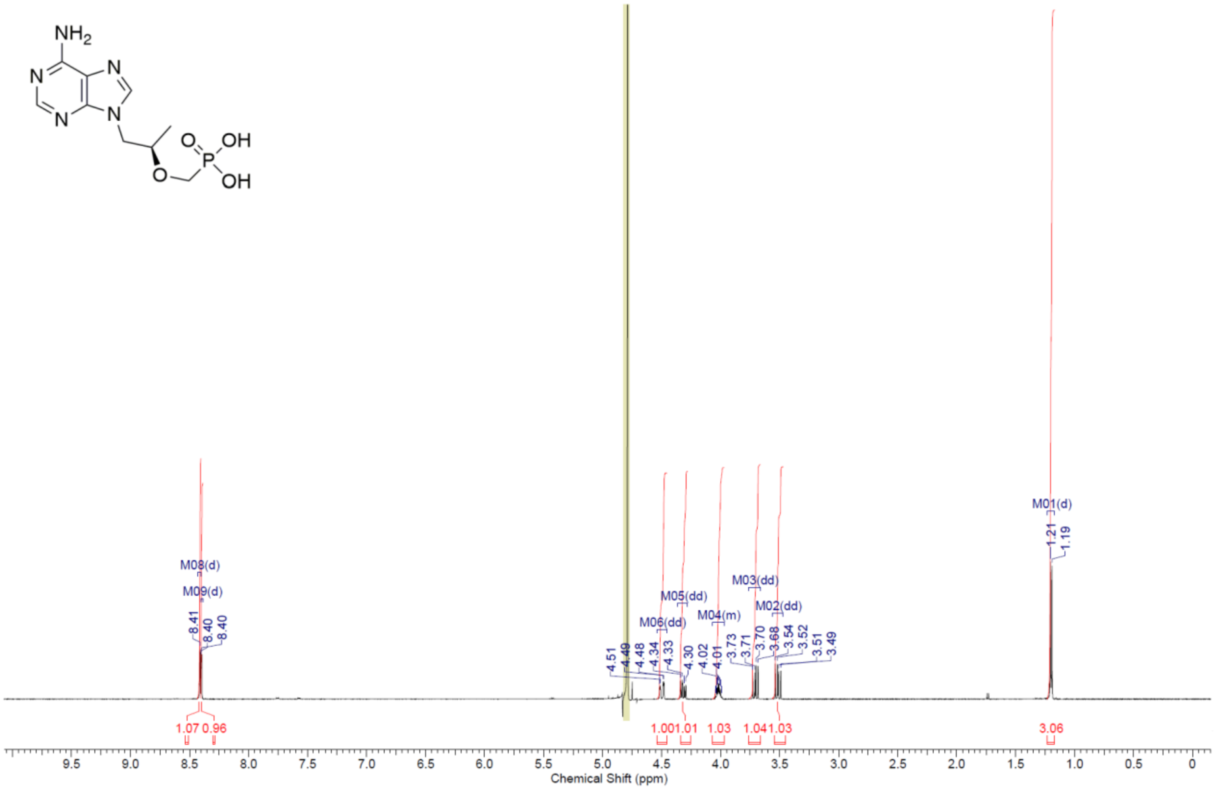

^{13}C{^{1}H} NMR spectrum of (*R*)-(–)-**11** (126 MHz, DMSO-d_6)

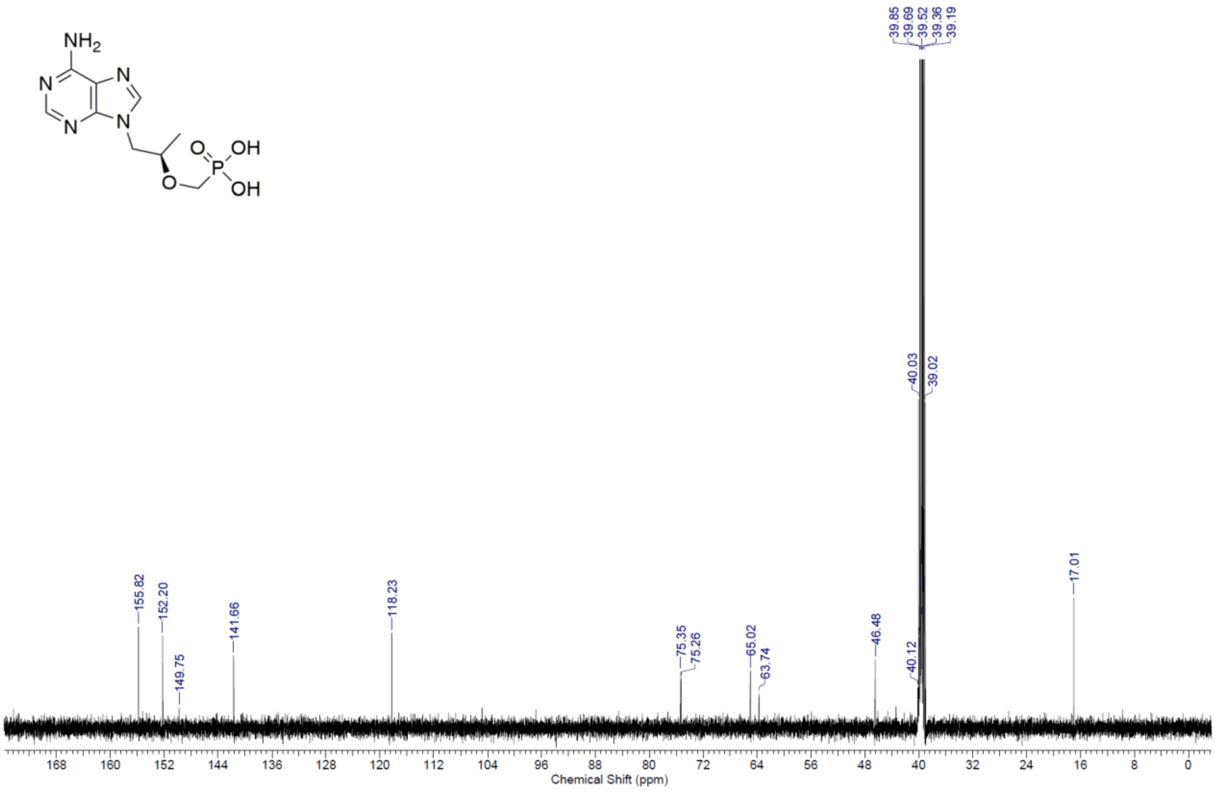

$^{31}P\{^{1}H\}$ spectrum of (*R*)-(–)-**11** (202 MHz, DMSO-d_6)

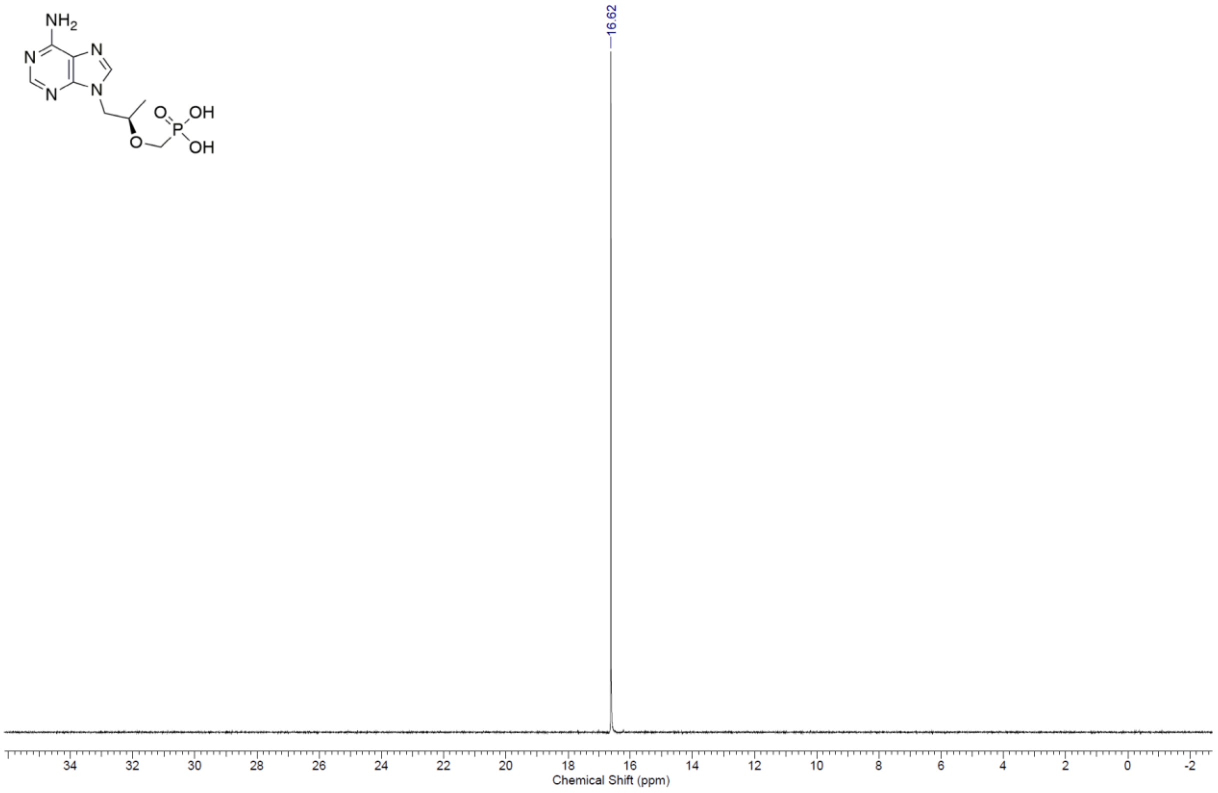

IR spectrum of (*R*)-(–)-**11** (Nujol)

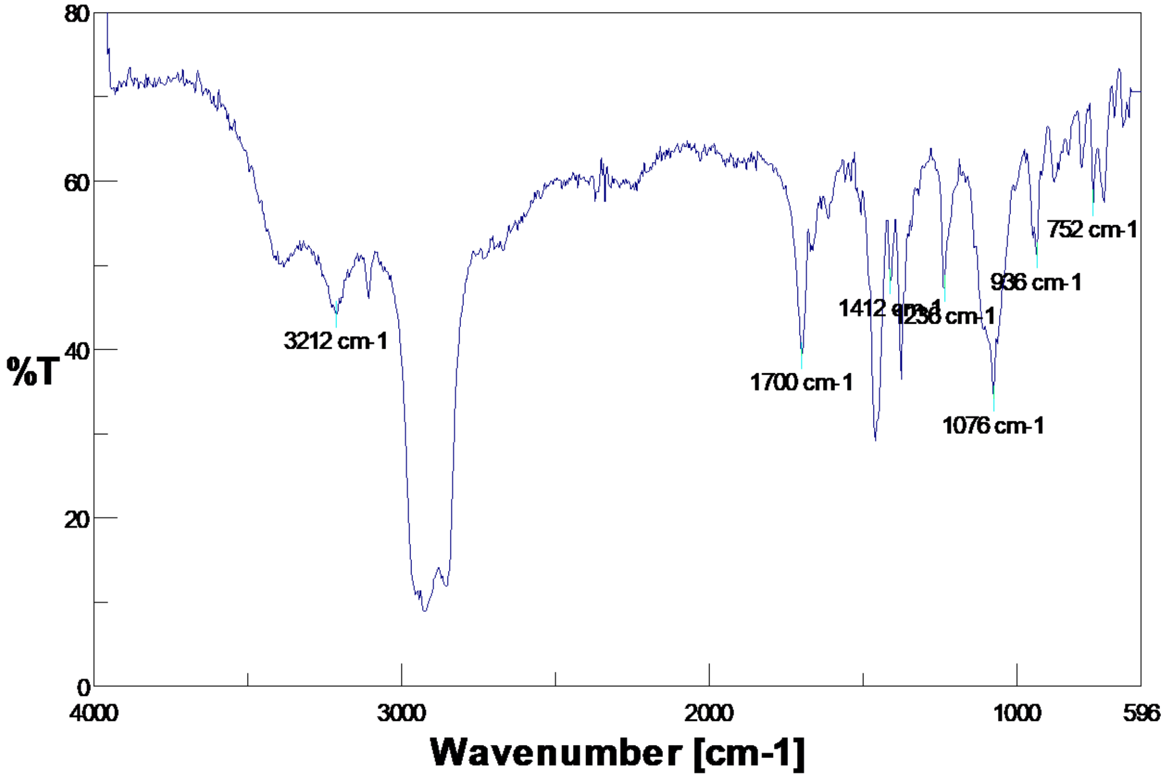

FTMS spectrum of (*R*)-(–)-**11** (ESI-TOF)

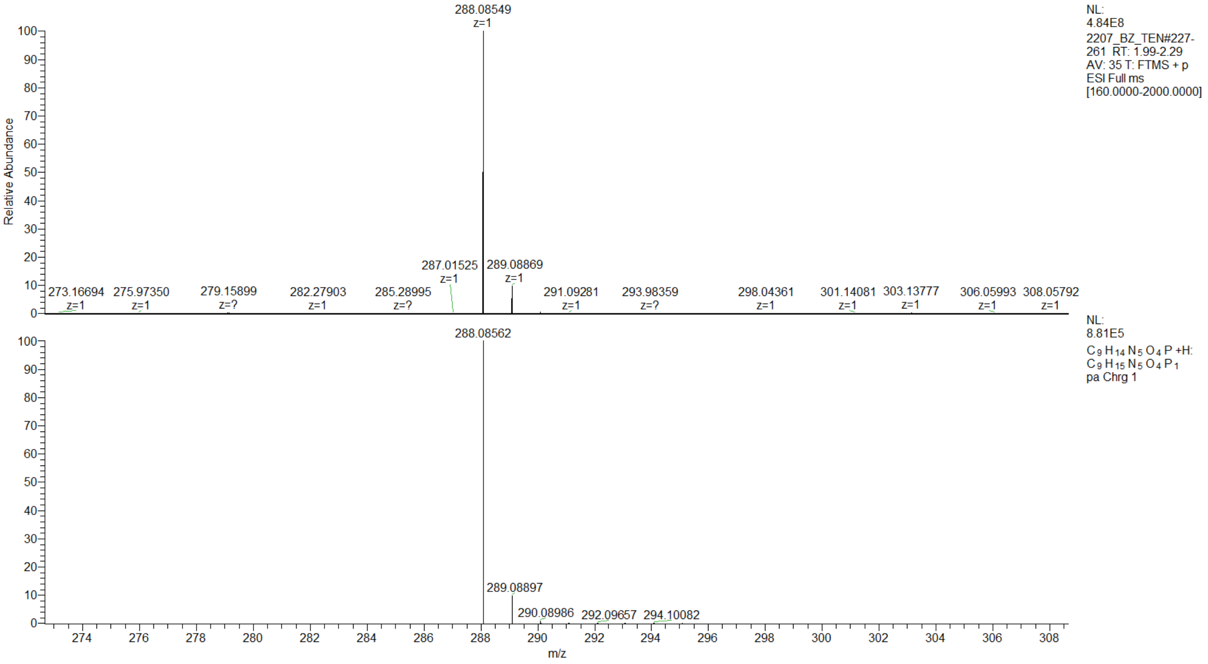

13. References

[1] G.B. Elion, G.H. Hitchings, Studies on Condensed Pyrimidine Systems. XVII. Some Halogenopurines, J. Am. Chem. Soc. 78(14) (2002) 3508–3510.

[2] K.X. Huang, M.S. Xie, Q.Y. Zhang, H.Y. Niu, G.R. Qu, H.M. Guo, Synthesis of Chiral Six-Membered Carbocyclic Purine Nucleosides via Organocatalytic Enantioselective [3 + 3] Annulation, Org. Lett. 20(17) (2018) 5398–5401.

[3] Q. Zhang, G. Cheng, Y.-Z. Huang, G.-R. Qu, H.-Y. Niu, H.-M. Guo, Regioselective N9 alkylation of purine rings assisted by β-cyclodextrin, Tetrahedron 68(38) (2012) 7822–7826.

[4] P. Pospisil, B.D. Pilger, S. Marveggio, P. Schelling, C. Wurth, L. Scapozza, G. Folkers, M. Pongracic, M. Mintas, S.R. Malic, Synthesis, Kinetics, and Molecular Docking of Novel 9-(2-Hydroxypropyl)purine Nucleoside Analogs as Ligands of Herpesviral Thymidine Kinases, Helv. Chim. Acta 85(10) (2002) 3237–3250.

[5] Q. Zhang, B.W. Ma, Q.Q. Wang, X.X. Wang, X. Hu, M.S. Xie, G.R. Qu, H.M. Guo, The synthesis of tenofovir and its analogues via asymmetric transfer hydrogenation, Org. Lett. 16(7) (2014) 2014–2017.

[6] K. Kondo, T. Sato, K. Takemoto, Reaction of Nucleic Acid Bases with Epoxides and Lactone, Chem. Lett. 2(9) (1973) 967–968.

[7] A. Holý, M. Masojídková, Synthesis of Enantiomeric N-(2-Phosphonomethoxypropyl) Derivatives of Purine and Pyrimidine Bases. I. The Stepwise Approach, Collect. Czech. Chem. Commun. 60(7) (1995) 1196–1212.

[8] Chen, C.S.; Fujimoto, Y.; Girdaukas, G.; Sih, C.J. Quantitative analyses of biochemical kinetic resolutions of enantiomers, *J. Am. Chem. Soc.* **2002**, *104*, 7294–7299.

[9] Trott, O.; Olson, A.J. AutoDock Vina: improving the speed and accuracy of docking with a new scoring function, efficient optimization, and multithreading. *J. Comput. Chem.* **2010**, *31*, 455–461.

[10] Wang, J.; Wolf, R.M.; Caldwell, J.W.; Kollman, P.A.; Case, D.A. Development and testing of a general amber force field. *J. Comput. Chem.* **2004**, *25*, 1157–1174.

[11] Karabec, M.; Łyskowski, A.; Tauber, K. C.; Steinkellner, G.; Kroutil, W.; Grogan, G.; Gruber, K., Structural insights into substrate specificity and solvent tolerance in alcohol dehydrogenase ADH-‘A' from *Rhodococcus ruber* DSM 44541. *Chem. Commun.* **2010**, *46*, 6314–6316.

[12] Noey, E. L.; Tibrewal, N.; Jiménez-Osés, G.; Osuna, S.; Park, J.; Bond, C. M.; Cascio, D.; Liang, J.; Zhang, X.; Huisman, G. W.; Tang, Y.; Houk, K. N., Origins of stereoselectivity in evolved ketoreductases. *Proceed. Nat. Acad. Sci.* **2015**, *112*, E7065–E7072.

[13] Pettersen, E.F.; Goddard, T.D.; Huang, C.C.; Couch, G.S.; Greenblatt, D.M.; Meng, E.C.; Ferrin, T.E. UCSF Chimera--a visualization system for exploratory research and analysis. *J. Comput. Chem.* **2004**, *25*, 1605−1612.
