# Supplementary material for: Chemoenzymatic Synthesis of Tenofovir
Source: J Org Chem. 2023 Jul 19;88(15):11045–55. doi: 10.1021/acs.joc.3c01005 (PMC10407936; doi:10.1021/acs.joc.3c01005)
Supplement: Supplementary file 2 — jo3c01005_si_002.zip [file jo3c01005_si_002.zip › FID for Publication/Additional Information.docx]

^1^H NMR and ^13^C NMR spectra were recorded on a Spectrometr Varian NMR System 500 MHz (Varian, Inc., Palo Alto, CA, USA)]; chemical shifts (*δ*) are given in parts per million (ppm) on the delta scale related to the solvent peak used as reference value; signal multiplicity assignment: s, singlet; d, doublet; t, triplet; q, quartet; m, multiplet; coupling constant (*J*) are given in hertz (Hz); all samples were recorded as solutions in fully deuterated chloroform (CDCl_3_), dimethylsulfoxide (DMSO-*d_6_*), and deuterium oxide (D_2_O), respectively. All NMR reports for Supporting Information were created by ACD/NMR Processor Academic Edition 12.0. (Freeware software provided by ACD/Labs, USA & Canada).
